# Correction to “Photoelectrochemical Synthesis of Benzo[*b*]phosphole Oxides via Sequential P–H/C–H Bond Functionalizations”

**DOI:** 10.1021/acscatal.5c01983

**Published:** 2025-04-07

**Authors:** Nayan Saha, Burkhard König

We noticed technical errors in the Supporting Information provided for the article. In some cases, the copied chemical structures overlap with the displayed NMR spectra. We are providing a corrected version of the Supporting Information and include a repository link to all primary NMR data.

## Supporting Information Available

Experimental details and characterization data which includes NMR spectra of all of the products, spectroscopic and cyclic voltammetric details, and corresponding figures (PDF)

