# Supplementary material for: Correction to “Photoelectrochemical Synthesis of Benzo[b]phosphole Oxides via Sequential P–H/C–H Bond Functionalizations”
Source: ACS Catal. 2025 Apr 7;15(8):6544. doi: 10.1021/acscatal.5c01983 (PMC12012827; doi:10.1021/acscatal.5c01983)

Supporting Information

Photoelectrochemical Synthesis of Benzo[*b*]phosphole Oxides via Sequential P-H/C-H Bond Functionalizations

Nayan Saha[a] and Burkhard König*[a]

[a]Institute of Organic Chemistry, Faculty of Chemistry and Pharmacy, University of Regensburg, D-93053 Regensburg, Germany.

E-mail address: burkhard.koenig@ur.de

Primary research data of this study (FIDs of proton, carbon and phosphorous NMRs) are available at:

https://radar4chem.radar-service.eu/radar/en/dataset/ycx77xj3ue717uxc

TABLE OF CONTENTS

S.No.	Content	Page No.
1	General Information	S3
2	Materials	S4
3	Reaction Setup	S4
4	Spectrum of the Light Used for the Photochemical Reaction	S8
5	Calculations of Excited State Potential ($E^*_{1/2}$) of **PC$_1$** Using Rehm and Weller Equation	S8
6	Optimization of the Photoelectrochemical Annulation of Secondary Phosphine Oxides with Internal Alkynes	S10
7	General Procedure for the Photoelectrochemical Annulation of Secondary Phosphine Oxides with Internal Alkynes	S15
8	Procedure for the Photoelectrochemical Dehydrogenative Annulation in 2.0 mmol Scale	S16
9	Cyclic Voltammetry Studies	S16
10	Spectroelectrochemistry Studies	S21
11	UV-Visible Spectroscopic Studies	S22
12	Fluorescence Studies	S26
13	Electron Paramagnetic Resonance (EPR) Analysis	S27
14	Radical Inhibition Experiments	S29
15	Light On-Off and Electricity On-Off Experiment	S31
16	Reusability of the Photocatalyst and Electrodes (one pot batch scale up)	S34
17	Detection of H_2 gas Evolution	S35
18	Kinetic Isotopic Effect Experiment	S36
19	Photophysical Properties of some Benzo[*b*]Phosphole Oxide Products	S38
20	Intermolecular Competition Experiments	S43
21	Characterization Data of the Products	S45
22	References	S75
23	^{1}H, and ^{13}C spectra of **PC$_1$**	S76
24	^{1}H, ^{13}C, ^{31}P, and ^{19}F spectra of the Products	S77

1. General Information:

All the photoelectrochemical reactions were conducted using a well-designed setup comprising of IKA ElectraSyn 2.0 and blue light irradiation from a Kessil® PR160-456 nm lamp. Commercially available reaction vials and electrodes (graphite and platinum foil respectively (0.8 cm × 0.2 cm × 5.2 cm)) were purchased from IKA. Ambient temperature throughout the course of the reaction was maintained by an external cooling device. A distance of 3 cm was maintained in between the vial and the Kessil light source.

UV-Visible experiments were conducted on Agilent Cary 4000 UV-Vis spectrophotometer using dimethylformamide (DMF). The emission spectra were recorded on Horiba FluoroMax-4 spectrofluorometer using dimethylformamide (DMF). Cyclic voltammetry (CV) and Chronoamperometry measurements was carried out using a three-electrode potentiostat galvanostat PGSTAT302N from Metrohm Autolab. A glassy carbon working electrode (disk, diameter: 3mm), a coiled platinum wire counter electrode, a silver wire as pseudo reference electrode and ferrocene as the internal reference were employed for the CV studies. Spectroelectrochemistry (SEC) was carried out using Cary 8454 Online UV-Vis spectrophotometer. Chromatographic purification of products was accomplished by column chromatography on Merck silica gel 60 M (0.040-0.063 mm, 230-440 mesh). For thin layer chromatography (TLC) analysis throughout this work, ALUGRAM Xtra SIL G UV254 Ref. 818333 pre-coated TLC plates (silica gel 60 GF_{254}, 0.25 mm) were employed, using UV light (254 nm, 365 nm) as the visualizing agent. Organic solutions were concentrated under reduced pressure using Buchi rotary evaporator. The products obtained were characterised by using ^{1}H NMR, ^{13}C NMR, ^{31}P NMR, ^{19}F NMR, and HRMS. NMR spectra were recorded at 400 MHz using Bruker Avance 400 spectrometer for ^{1}H, 101 MHz for ^{13}C, 162 MHz for ^{31}P, and 376 MHz for ^{19}F. The chemical shift (δ) for ^{1}H, ^{13}C, ^{31}P, and ^{19}F are given in ppm relative to internal standard/residual signals of the solvents (tetramethylsilane @ 0 ppm and $CDCl_3$ @ 7.26 ppm in ^{1}H NMR and $CDCl_3$ @ 77.00 ppm in ^{13}C NMR). Coupling constants (*J*) are given in hertz (Hz). The following abbreviations are followed to indicate the multiplicity: s, singlet; d, doublet; t, triplet; q, quartet; sept, septet; m, multiplet; dd, doublet of doublets; ddd, doublet of doublet of doublets; dddd, doublet of doublet of doublets of doublets; td, triplet of doublets; qd, quartet of doublets. High-resolution mass spectra (HRMS) were obtained from the High-Resolution Mass Spectrometry unit on MicroTOF Focus with electrospray ionization using Agilent Q-TOF 6540 UHD instrument at the Central Analytical Laboratory of the University of Regensburg. A 7890B Gas Chromatography (GC) System from Agilent Technologies was used for quantitative yield determination. GC-MS measurements were performed on a 7890A GC system from Agilent Technologies with an Agilent 5975 MSD Detector. H_2 detection via headspace GC-TCD was performed using the Inficon 300 MicroGC instrument. The ^{1}H NMR spectra of some compounds contain residual solvent peaks from ethyl acetate and DMF (< 0.5%) which were unable to remove even after multiple attempts as the compounds being highly polar, tend to trap them.

2. Materials:

Synthesis grade solvents were used as purchased. Secondary phosphine oxides, alkyl aryl phosphinates, internal alkynes and their derivatives were purchased from Sigma-Aldrich, Merck, BLDpharm, Alfa Aesar, TCI and Acros. All the other commercial grade reagents and solvents were purchased from Sigma-Aldrich at the highest commercial quality and used without further purification, unless otherwise stated. **PC_1** is prepared according to the reported method[1]. Few Secondary phosphine oxides were prepared according to the precedented literature procedures[2].

For **PC_1**:

^{1}H NMR (400 MHz, $CDCl_3$) δ 8.22 (dt, *J* = 7.8, 0.9 Hz, 2H), 7.74 – 7.67 (m, 8H), 7.49 (ddd, *J* = 8.0, 6.6, 1.6 Hz, 2H), 7.35 – 7.31 (m, 2H), 7.24 – 7.20 (m, 4H), 7.13 – 7.04 (m, 8H), 6.85 – 6.79 (m, 4H), 6.63 (ddd, *J* = 8.4, 7.3, 1.2 Hz, 2H) ppm.

^{13}C{^{1}H} NMR (101 MHz, $CDCl_3$) δ 145.2, 144.6, 140.0, 138.2, 137.0, 127.0, 125.8, 125.0, 124.8, 124.6, 123.9, 122.4, 121.9, 121.4, 121.0, 120.4, 119.7, 116.4, 111.6, 110.0, 109.5, 109.4 ppm.

3. Reaction Setup:

The photoelectrochemical reaction setups are detailed in Figure S1-S4. To perform electrolysis, ElectraSyn vial of 5 mL capacity was used. An IKA Graphite SK-50 electrode as the anode and an IKA Platinum foil electrode as the cathode were inserted into the ElectraSyn vial cap. This vial cap was then fitted tightly to the reaction vial consisting of reaction mixture and a magnetic stirring bead. The complete setup was connected to the vial holder of ElectraSyn 2.0 and placed at a distance of 3 cm under irradiation with a Kessil® PR160-456 nm lamp equipped with an external cooling device for maintaining the ambient temperature throughout the course of the reaction (Fig. S2-S4). Temperature range was recorded between 25 – 32°C with an average of 28°C.

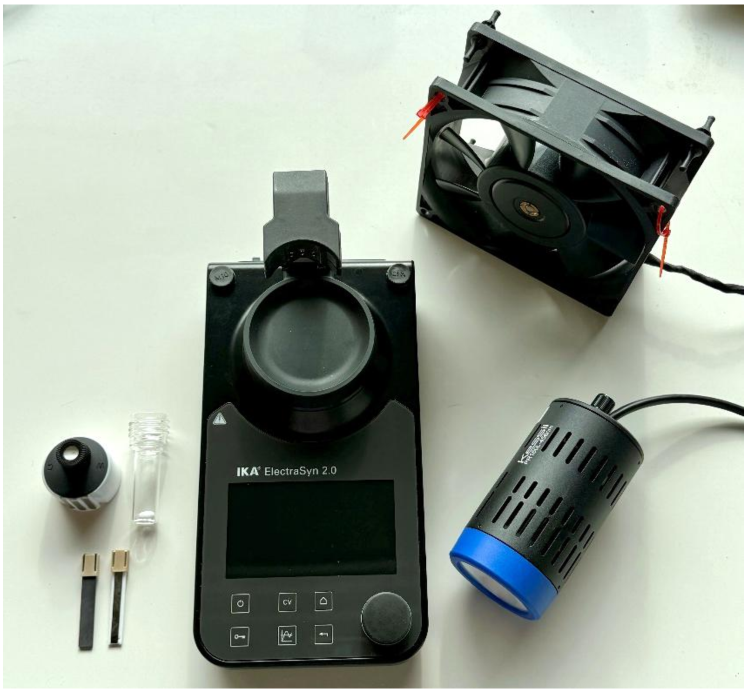

Figure S1: ***<u>Photoelectrochemical reaction kit</u>***: IKA ElectraSyn 2.0, reaction vial with magnetic stir bar, electrodes, cap, external cooling device and Kessil® PR160-456 nm lamp.

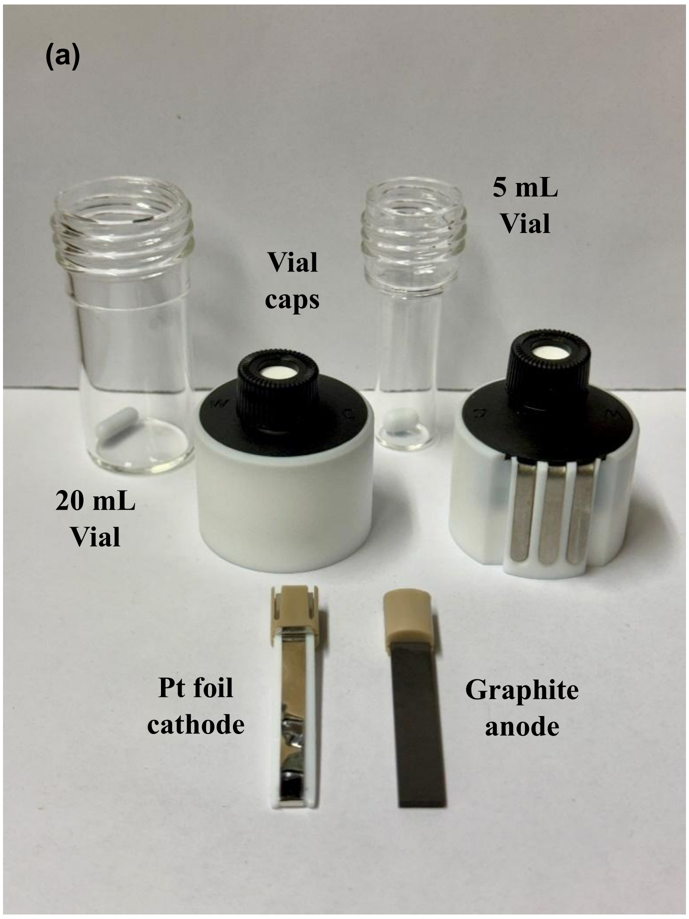

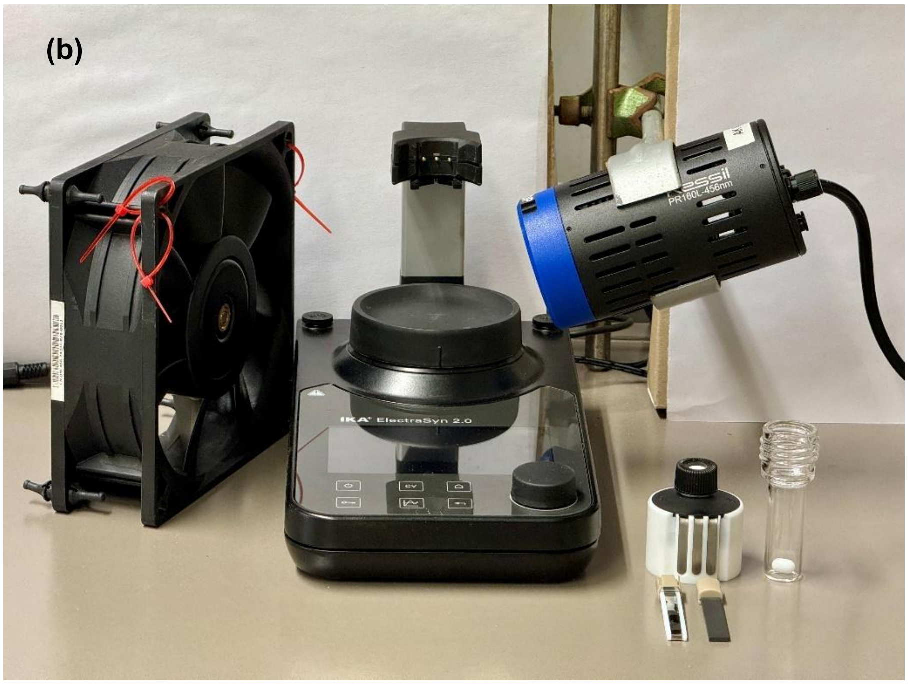

Figure S2: ***<u>Photoelectrochemical reaction setup</u>***: **(a)** ElectraSyn 2.0. reaction vials, vial holders, electrodes and caps; **(b)** The complete setup equipped with external cooling fan.

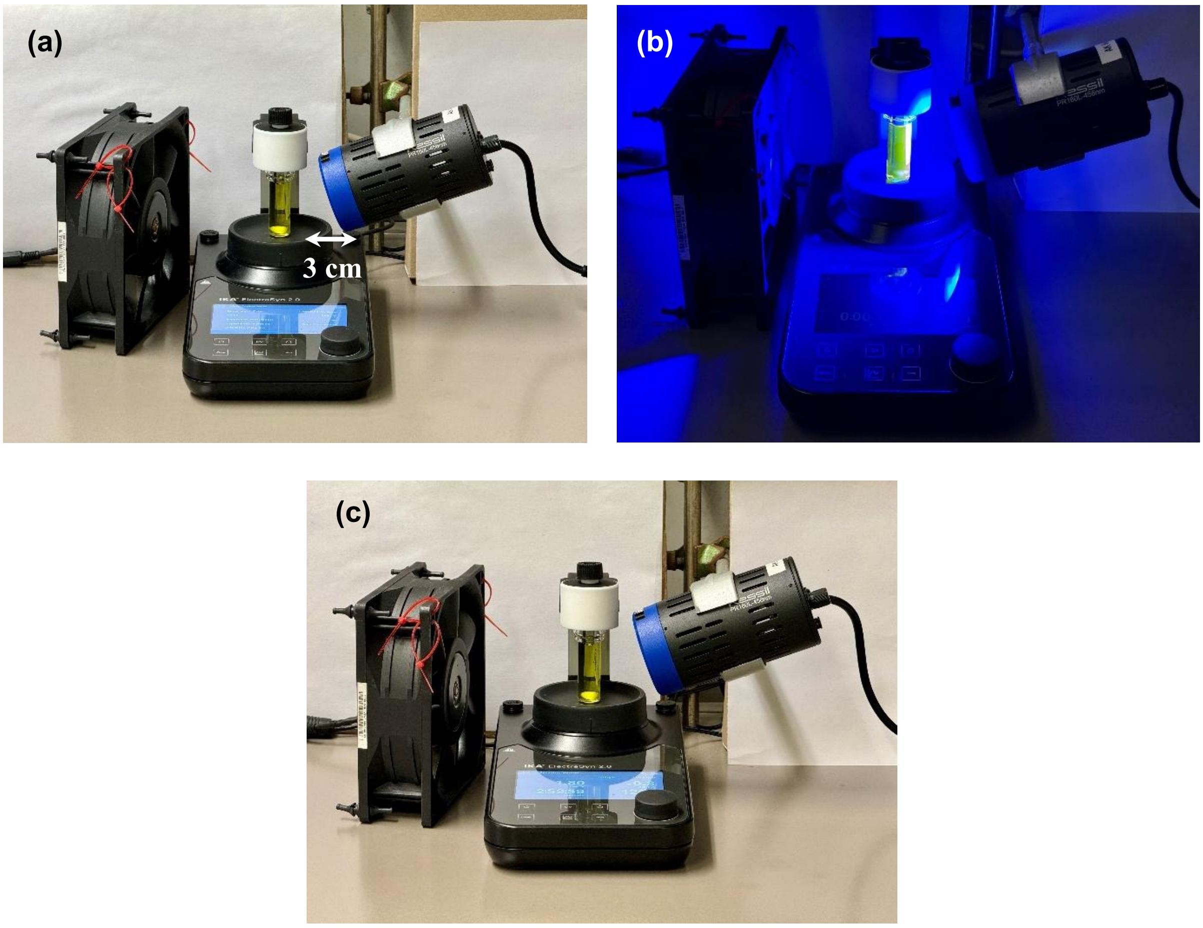

Figure S3: ***Photoelectrochemical reaction setup***: **(a)** Before excitation, **(b)** During 456 nm Kessil lamp excitation, **(c)** After completion of the reaction.

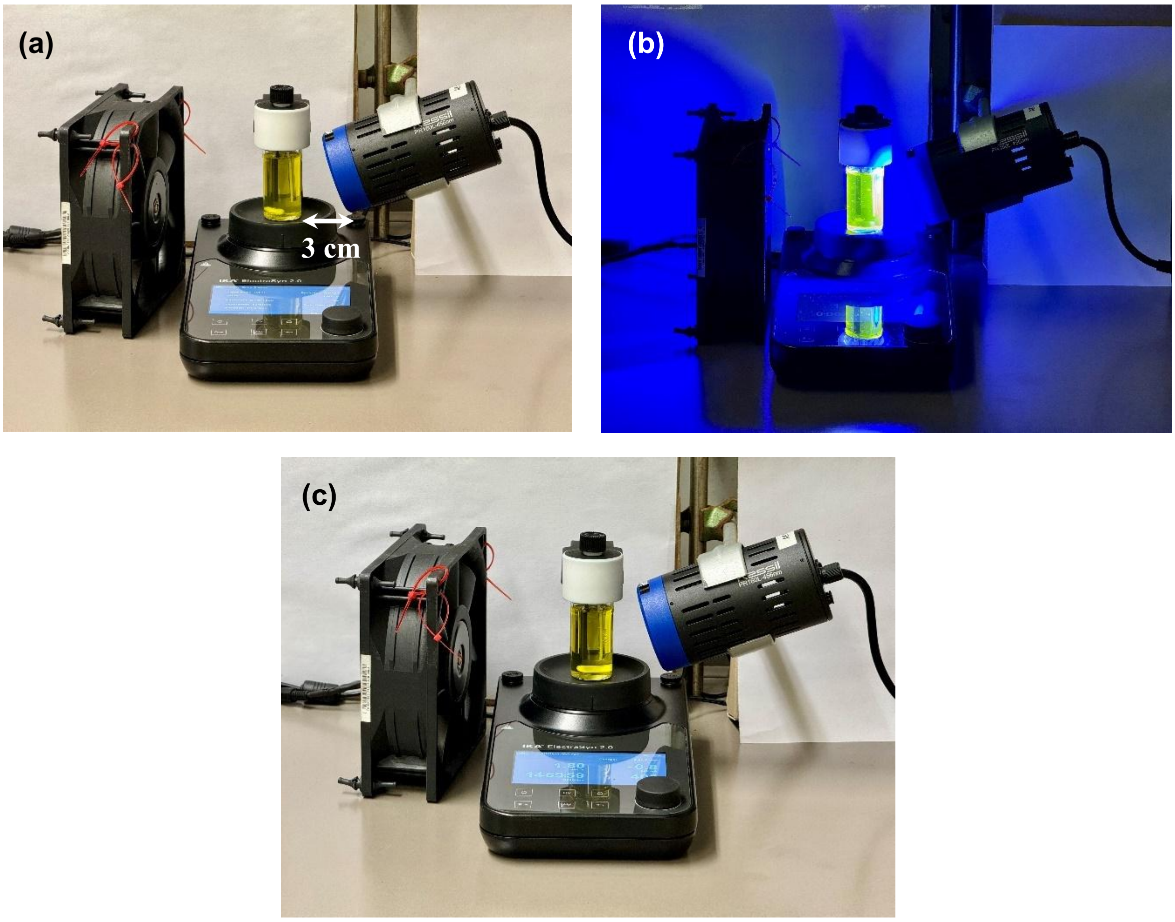

Figure S4: ***Photoelectrochemical reaction setup for scale-up***: **(a)** Before excitation, **(b)** During 456 nm Kessil lamp excitation, **(c)** After completion of the reaction.

4. Spectrum of the Light Used for the Photochemical Reaction:

The emission spectra of the 456 nm Kessil light was measured by a FieldMaxII-TO Laser Power Meter (Measurement range of 1 nW to 10 kW) and PM10 TOP Assy High Sensitivity Sensor (Measurement range of 5 mW to 10 W).

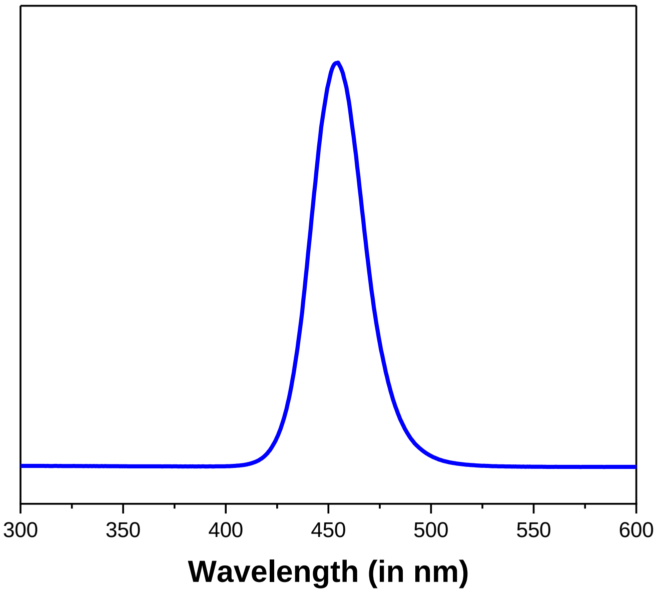

Figure S5: Emission spectra of 456 nm Kessil light source.

The light source was placed in front of the Power Meter connected via a fiber and emission was recorded with the sensor. Finally, the data obtained was plotted as shown in Fig S5.

5. Calculations of Excited State Potential ($E^*_{1/2}$) of PC_1 Using Rehm and Weller Equation:

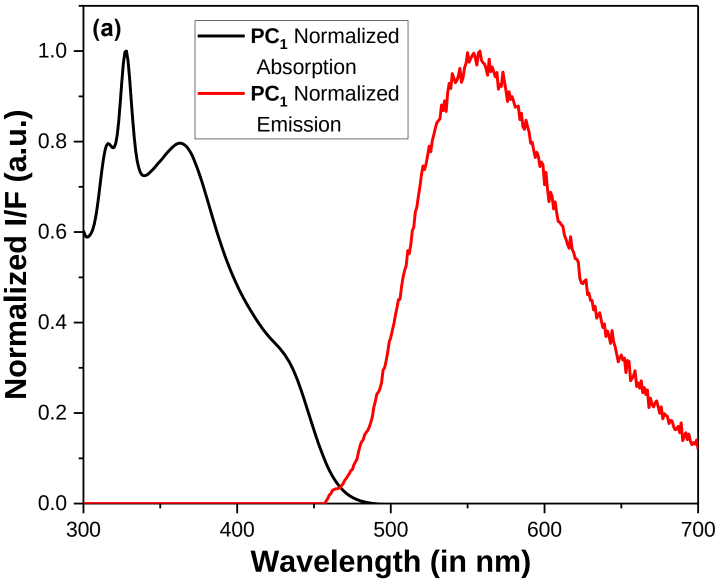

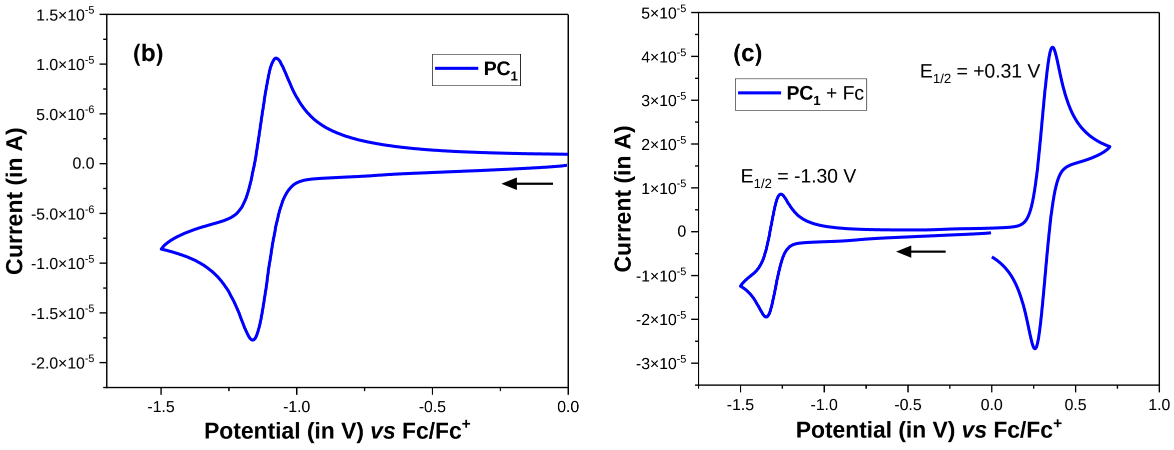

Figure S6: Electrochemical and photophysical properties of **PC_1**: (a) Normalized absorption and emission spectrum of **PC_1**, (b) CV of **PC_1** (reduction range), (c) CV of **PC_1** along with ferrocene.

The Rehm-Weller equation for the calculation of the excited state potential is given by equation[3]:

$$\mathbf{E^*_{1/2} = E_{1/2} + E_{00}}$$

Where, $\mathbf{E^*_{1/2}}$ = excited-state redox potential, $\mathbf{E_{1/2}}$ = ground-state redox potential (known by experiment) and $\mathbf{E_{00}}$ = mean photon energy of the emission spectra in eV (by the exciting wavelength 456 nm and 400 nm).

E (*vs.* SCE) = **E** (*vs.* Fc/Fc^+) + 0.38 V.

For **PC_1**,

$\mathbf{E_{1/2}}$ = -1.23 V (from CV *vs.* SCE)

$\mathbf{E_{00}}$ = 2.72 eV (for 456 nm)

$\mathbf{E_{00}}$ = 2.95 eV (for 420 nm)

$\mathbf{E_{00}}$ = 2.83 eV (for (420+456)/2=438 nm).

Therefore, $E^*_{1/2}$ comes out to be **+1.49 V** (for 456 nm) and $E^*_{1/2}$ comes out to be **+1.72 V** (for 420 nm). $E^*_{1/2}$ comes out to be **+1.6 V** (for 438 nm). Note: We have considered the highest absorption wavelength (456 nm) of **PC_1** and lowest emission wavelength (420 nm) of the blue LED (456 nm) light used.

If we consider, E_{00} from the intersection of absorption spectra and emission spectra,

E_{00} = 2.66 eV (for 466 nm)

$E^*_{1/2}$ = **+1.43 V** (for 466 nm)

6. Optimization of the Photoelectrochemical Annulation of Secondary Phosphine Oxides with Internal Alkynes :

Initially, we electrolyzed an electrolytic solution ($TBAPF_6$ as an electrolyte in MeCN) containing **1a**, **2a**, and **PC_1** in an undivided electrochemical cell upon continuous blue light (456 nm) irradiation from Kessil lamp equipped with an external cooling fan.

Table S1: Optimization of the photoelectrochemical annulation with different solvents and electrodes' combination.[a]

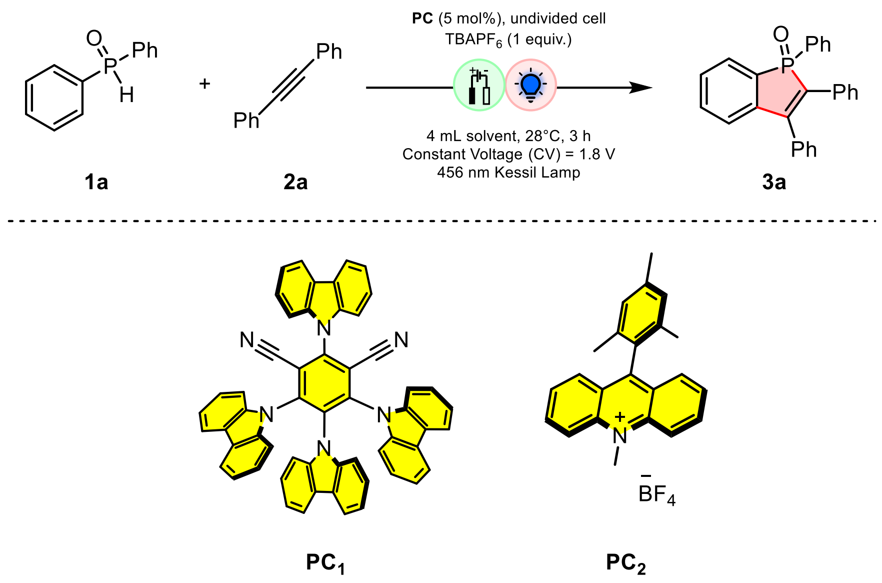

Entry	Electrodes (+ | -)	Solvent	Yield (%)[b]
1	Graphite | Graphite	CH_3CN	27
2	Graphite | Graphite	DMF	42
3	Graphite | Graphite	$CH_3CN:H_2O$ = 4:1 (v/v)	25
4	Graphite | Graphite	CH_3CN:MeOH = 4:1 (v/v)	21
5	Graphite | Graphite	DMF:H_2O = 4:1 (v/v)	39
6	Graphite | Stainless Steel	DMF	45
7	Graphite | Ni foam	DMF	51
8	Graphite | Pt	CH_3CN	20

9	Graphite | Pt	DMF:H_2O = 4:1 (v/v)	34
10	Graphite | Pt	DMF:MeOH = 4:1 (v/v)	18
11	Graphite | Pt	CH_3CN:H_2O = 4:1 (v/v)	19
12	Graphite | Pt	DMA	68
**13**	**Graphite | Pt**	**DMF**	**93**
14[c]	Graphite | Pt	DMF	10
15[d]	Graphite | Pt	DMF	14
16[e]	Graphite | Pt	DMF	8
17[f]	Graphite | Pt	DMF	5
18[g]	Graphite | Pt	DMF	92

[a]Standard reaction condition unless otherwise specified: **1a** (0.4 mmol), **2a** (0.2 mmol), **PC_1** (5 mol%), $TBAPF_6$ as electrolyte (1.0 equiv.), solvent (4 mL), light irradiation from 456 nm Kessil lamp, electrolysis by using constant voltage (CV) of +1.8 V in an undivided cell equipped with anode (+) and cathode (-) for 3 h, [b]Yield determined by gas chromatography with reference to benzophenone as internal standard. [c]using **PC_2** instead of **PC_1**. [d]no electricity. [e]in dark. [f]no **PC_1**. [g]under Ar atmosphere.

The electrolysis of this mixture by using graphite as an anode and the same as cathode for 3 h resulted only 27% (Table S1, entry 1) of the desired benzo[*b*]phosphole oxide **3a**. On replacing the solvent from MeCN to DMF produced 42% of **3a** (Table S1, entry 2). Solvent mixtures like MeCN-H_2O (4:1 v/v), MeCN-MeOH (4:1 v/v), and DMF-H_2O (4:1 v/v) furnished 25, 21 and 39% of **3a** respectively, which might be due to poor solubility of the starting materials (Table S1, entries 3-5). However, the attempt to replace the electrode combination from graphite/graphite to graphite/stainless steel, graphite/nickel foam was found to be quite successful with significant enhancement of the yield was observed (Table S1, entries 6-7). On using graphite/platinum foil as the electrode combination, we obtained still lower yields using MeCN, DMF-H_2O (4:1 v/v), DMF-MeOH (4:1 v/v), and MeCN-H_2O (4:1 v/v) solvent combinations (Table S1, entries 8-11). We envisioned sudden increment in the yield to 68% (Table S1, entry 12) by using DMA as the solvent with graphite as an anode and Pt foil as cathode. When the reaction was conducted in DMF by applying +1.8 V constant potential for 3 h (2.4 F/mol), to our delight, yield of **3a** was dramatically increased to 93% (Table S1, entry 13). The reaction did not proceed well

with **PC_2** as the photocatalyst (Table S1, entry 14). To our delight, we found this newly developed protocol to be indeed a photoelectrochemical as only 14% formation of the desired product was noticed in the absent of current electricity (Table S1, entry 15). The necessity of continuous light irradiation was demonstrated by conducting the standard electrolysis in dark which resulted in only 8% (Table S1, entry 16) of the desired product. We were delighted to observe only 5% product formation in absence of **PC_1** (Table S1, entry 17). Next, we moved on to checking different electrolytes, electrochemical conditions, effect of incorporating bases, amount of photocatalyst needed for the reaction and progress of the reaction with time. We observed no significant variation in the yield of **3a** when the reaction was carried out under argon atmosphere (Table S1, entry 18).

Table S2: Optimization of the photoelectrochemical annulation with different electrolytes.[a]

Entry	Electrolyte	Yield (%)[b]
1	$^{n}Bu_4BF_4$	72
**2**	**$^{n}Bu_4PF_6$**	**93**
3	$^{n}Bu_4ClO_4$	51
4	$^{n}Bu_4Br$	n.d.

[a]Standard reaction condition unless otherwise specified: **1a** (0.4 mmol), **2a** (0.2 mmol), **PC_1** (5 mol%), electrolyte (1.0 equiv.), DMF as solvent (4 mL), light irradiation from 456 nm Kessil lamp, electrolysis by using constant voltage (CV) of +1.8 V in an undivided cell equipped with anode (+) and cathode (-) for 3 h, [b]Yield determined by gas chromatography with reference to benzophenone as internal standard. n.d. = not detected.

Table S3: Optimization of the photoelectrochemical annulation with different electrochemical conditions.[a]

Entry	Electrochemical Condition	Yield (%)[b]
1	+ 1.0 V CV	23
2	+ 1.5 V CV	82
**3**	**+ 1.8 V CV**	**93**

4	+ 2.0 V CV	73
5	+ 2.5 V CV	61
6	3 mA CC	44

[a]Standard reaction condition unless otherwise specified: **1a** (0.4 mmol), **2a** (0.2 mmol), **PC_1** (5 mol%), $TBAPF_6$ as electrolyte (1.0 equiv.), DMF as solvent (4 mL), light irradiation from 456 nm Kessil lamp, electrolysis by using respective electrochemical condition in an undivided cell equipped with anode (+) and cathode (-) for 3 h, [b]Yield determined by gas chromatography with reference to benzophenone as internal standard. CV = constant voltage, CC = constant current.

Table S4: Optimization of the photoelectrochemical annulation with different bases.[a]

Entry	Base	Yield (%)[b]
1	$NaHCO_3$	33
2	K_3PO_4	37
3	Et_3N	23
**4**	**–**	**93**

[a]Standard reaction condition unless otherwise specified: **1a** (0.4 mmol), **2a** (0.2 mmol), **PC_1** (5 mol%), $TBAPF_6$ as electrolyte (1.0 equiv.), DMF as solvent (4 mL), base (1.5 equiv.) light irradiation from 456 nm Kessil lamp, electrolysis by using constant voltage (CV) of +1.8 V in an undivided cell equipped with anode (+) and cathode (-) for 3 h, [b]Yield determined by gas chromatography with reference to benzophenone as internal standard.

Table S5: Optimization of the photoelectrochemical annulation with different catalytic amounts of PC_1.[a]

Entry	PC_1 mol%	Yield (%)[b]
1	2	51

2	3	83
**3**	**5**	**91**
4	7	74
5	10	69

[a]Standard reaction condition unless otherwise specified: **1a** (0.4 mmol), **2a** (0.2 mmol), **PC_1** (x mol%), $TBAPF_6$ as electrolyte (1.0 equiv.), DMF as solvent (4 mL), base (1.5 equiv.) light irradiation from 456 nm Kessil lamp, electrolysis by using constant voltage (CV) of +1.8 V in an undivided cell equipped with anode (+) and cathode (-) for 3 h, [b]Yield determined by gas chromatography with reference to benzophenone as internal standard.

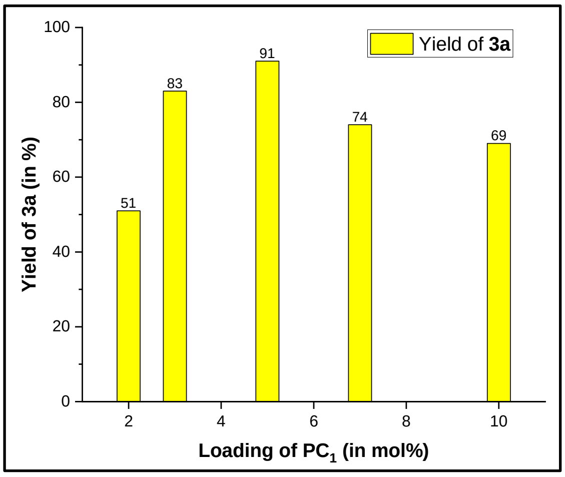

Figure S7: Variation of the yield of **3a** with different catalytic amount loadings of **PC_1**.

Table S6: Optimization of the photoelectrochemical annulation with time.[a]

**Entry**	**Time (min)**	**Yield (%)**[b]
1	20	21
2	40	35
3	60	52
4	80	68

5	100	73
6	120	78
7	140	83
8	160	87
9	180	91
10	200	93

[a]Standard reaction condition unless otherwise specified: **1a** (0.4 mmol), **2a** (0.2 mmol), **PC$_1$** (5 mol%), TBAPF$_6$ as electrolyte (1.0 equiv.), DMF as solvent (4 mL), base (1.5 equiv.) light irradiation from 456 nm Kessil lamp, electrolysis by using constant voltage (CV) of +1.8 V in an undivided cell equipped with anode (+) and cathode (-) with time, [b]Yield determined by gas chromatography with reference to benzophenone as internal standard.

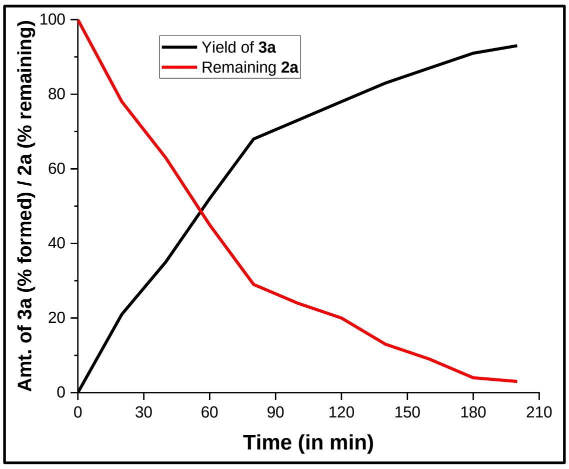

Figure S8: Progress of the yield of **3a** with time.

7. General Procedure for the Photoelectrochemical Annulation of Secondary Phosphine Oxides with Internal Alkynes:

An oven dried ElectraSyn vial (5 mL) equipped with magnetic stirring bar was charged with Secondary Phosphine Oxide **1** (0.4 mmol, 2.0 equiv.), Alkyne **2** (0.2 mmol, 1.0 equiv.), **PC$_1$** (5 mol%, 0.01 mmol), and TBAPF$_6$ (0.2 mmol, 1.0 equiv.). Exact 4 mL of DMF was added to the ElectraSyn vial. An ElectraSyn vial cap equipped with Graphite SK-50 as an anode and IKA Platinum foil as cathode was then fitted tightly to the reaction vial. The whole reaction mixture was then stirred vigorously for 10

minutes to make the mixture homogenous. Without any precaution of excluding air, the complete setup was connected to the vial holder of ElectraSyn 2.0 and placed at a distance of 3 cm under irradiation with a Kessil® PR160-456 nm lamp equipped with an external cooling device for maintaining the ambient temperature throughout the course of the reaction. The reaction mixture was simultaneously electrolysed for 3 h by applying 1.8 V constant potential with 1200 rpm magnetic stirring (Fig. S3). After completion of the reaction, saturated sodium chloride solution (25.0 mL) was added to the reaction mixture and was extracted with ethyl acetate (3 × 25.0 mL), dried using anhydrous sodium sulfate, concentrated in vacuo. The residue was finally purified using silica gel (230-400 mesh) column chromatography using mixture of EtOAc in hexane as eluent. Isolated yield of the mixture of two successive batches was reported.

8. Procedure for the Photoelectrochemical Dehydrogenative Annulation in 2.0 mmol Scale:

An oven dried ElectraSyn vial (20 mL) equipped with magnetic stirring bar was charged with Diphenylphosphine Oxide **1a** (4 mmol, 2.0 equiv.), Diphenylacetylene **2a** (2 mmol, 1.0 equiv.), **PC$_1$** (5 mol%, 0.1 mmol), and $TBAPF_6$ (2 mmol, 1.0 equiv.). Exact 15 mL of DMF was added to the ElectraSyn vial. An ElectraSyn vial cap equipped with Graphite SK-50 as an anode and IKA Platinum foil as cathode was then fitted tightly to the reaction vial. The whole reaction mixture was then stirred vigorously for 10 minutes to make the mixture homogenous. Without any precaution of excluding air, the complete setup was connected to the vial holder of ElectraSyn 2.0 and placed at a distance of 3 cm under irradiation with a Kessil® PR160-456 nm lamp equipped with an external cooling device for maintaining the ambient temperature throughout the course of the reaction. The reaction mixture was simultaneously electrolysed for 15 h by applying 1.8 V constant potential with 1200 rpm magnetic stirring (Fig. S4). After completion of the reaction, saturated sodium chloride solution (60.0 mL) was added to the reaction mixture and was extracted with ethyl acetate (3 × 50.0 mL), dried using anhydrous sodium sulfate, concentrated in vacuo. The residue was finally purified using silica gel (230-400 mesh) column chromatography using mixture of EtOAc in hexane (1:1) as eluent and isolated 79% of **3a** (598 mg).

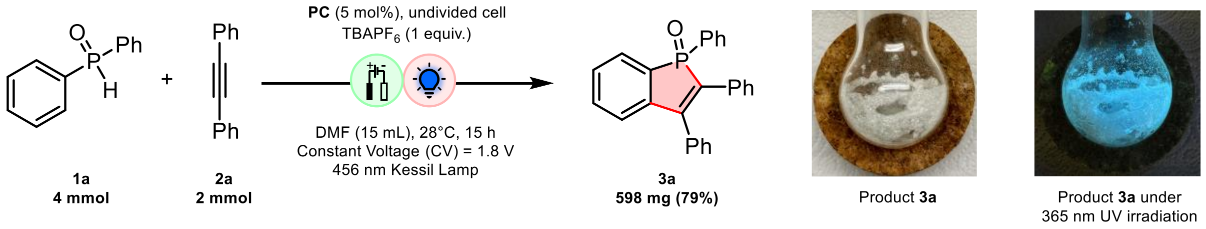

9. Cyclic Voltammetry Studies:

All the Cyclic Voltammetry (CV) experiments were carried out under Argon (Ar) atmosphere. A glassy carbon electrode (3 mm diameter) and a platinum wire were used as working and counter electrodes,

respectively. All the voltammograms were recorded in MeCN at room temperature with 0.1M $^{n}Bu_4NPF_6$ as supporting electrolyte. A silver wire was used as a reference electrode and ferrocene as the internal reference. The potential values were then reported relative to Fc/Fc^+, and SCE (using conversion factor) and voltammetric studies in MeCN were recorded and reported according to IUPAC conventions.

E (*vs.* SCE) = **E** (*vs.* Fc/Fc^+) + 0.38 V.

A. CV of 1a:

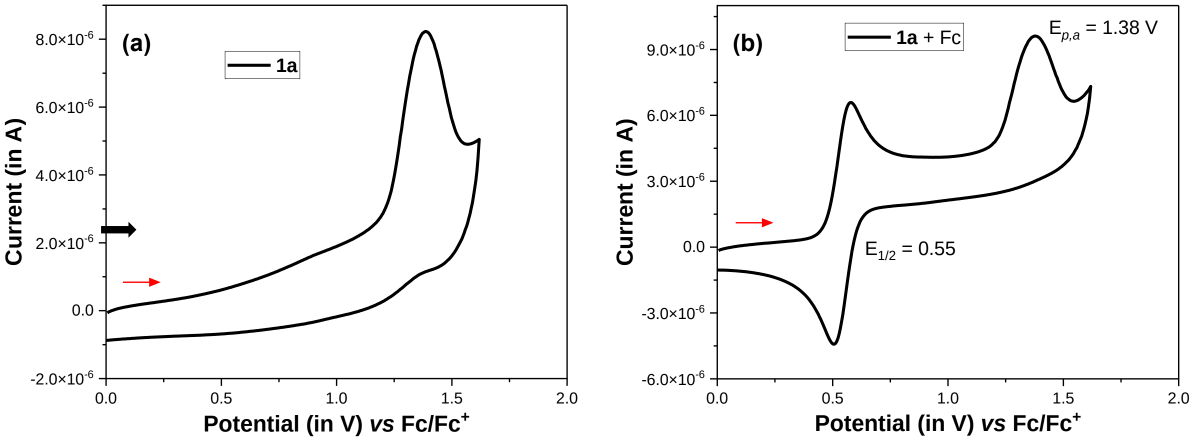

Figure S9: (a) CV of **1a** (oxidation range), **(b)** CV of **1a** with ferrocene.

The cyclic voltammogram of **1a** (using 0.1 M $^{n}Bu_4NPF_6$ as a supporting electrolyte in MeCN) was recorded (initial potential = 0 V, Upper vertex potential = +1.6 V, Lower vertex potential = 0 V, stopping potential = 0 V) with 50 mV/s scan rate (Fig. S9a).

$\mathbf{E_{p,a}}$ = + 1.21 V (*vs.* SCE).

B. CV of 2a:

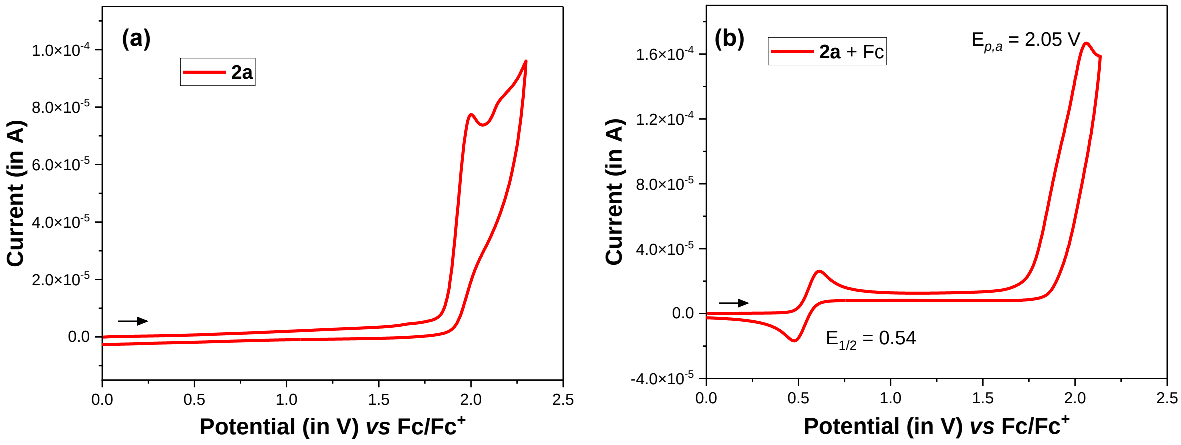

Figure S10: (a) CV of **2a** (oxidation range), **(b)** CV of **2a** with ferrocene.

Next, the cyclic voltammogram of **2a** (using 0.1 M $^{n}Bu_4NPF_6$ as a supporting electrolyte in MeCN) was recorded (initial potential = 0 V, Upper vertex potential = +2.2 V, Lower vertex potential = 0 V, stopping potential = 0 V) with 50 mV/s scan rate (Fig. S10a).

$\mathbf{E}_{p,a}$ = + 1.89 V (*vs.* SCE).

C. CV of $\mathbf{PC_1}$:

Next, the cyclic voltammogram of $\mathbf{PC_1}$ (using 0.1 M $^{n}Bu_4NPF_6$ as a supporting electrolyte in MeCN) was recorded (initial potential = 0 V, Upper vertex potential = 0 V, Lower vertex potential = −1.5 V, stopping potential = 0 V) with 50 mV/s scan rate (Fig. S6b).

D. Stacked CV:

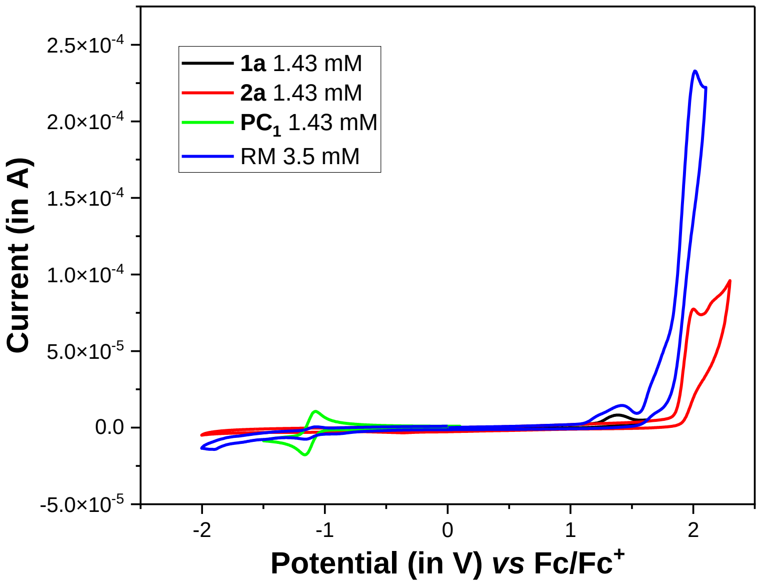

Figure S11: Stacked CV of 1a, 2a, PC_1, and the reaction mixture.

E. CV studies of 1a at different scan rates (oxidation range) and plot of current density vs square of the scan rate (Randles–Ševčík equation):

A solution of **1a** (using 0.1 M $^{n}Bu_4NPF_6$ as a supporting electrolyte in MeCN) was subjected to CV experiment with different scan rates (50 mV/s, 100 mV/s, 200 mV/s, 300 mV/s, 400 mV/s, 500 mV/s). A plot of current density vs square root of scan rate[4] was drawn (Fig. S12).

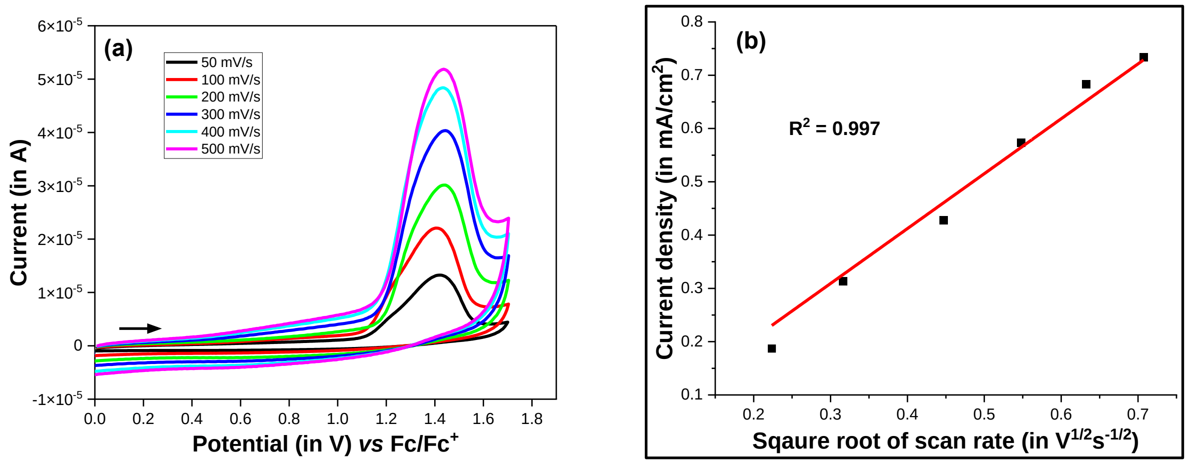

Figure S12: (a) The cyclic voltammogram at different scan rate in case of a solution containing **1a**, (b) The plot of current density vs square root of scan rate.

F. CV studies of 2a at different scan rates (oxidation range) and plot of current density vs square of the scan rate (Randles–Ševčík equation):

A solution of **2a** (using 0.1 M $^{n}Bu_4NPF_6$ as a supporting electrolyte in MeCN) was subjected to CV experiment with different scan rates (50 mV/s, 100 mV/s, 200 mV/s, 300 mV/s, 400 mV/s, 500 mV/s). A plot of current density vs square root of scan rate was drawn (Figure S13).

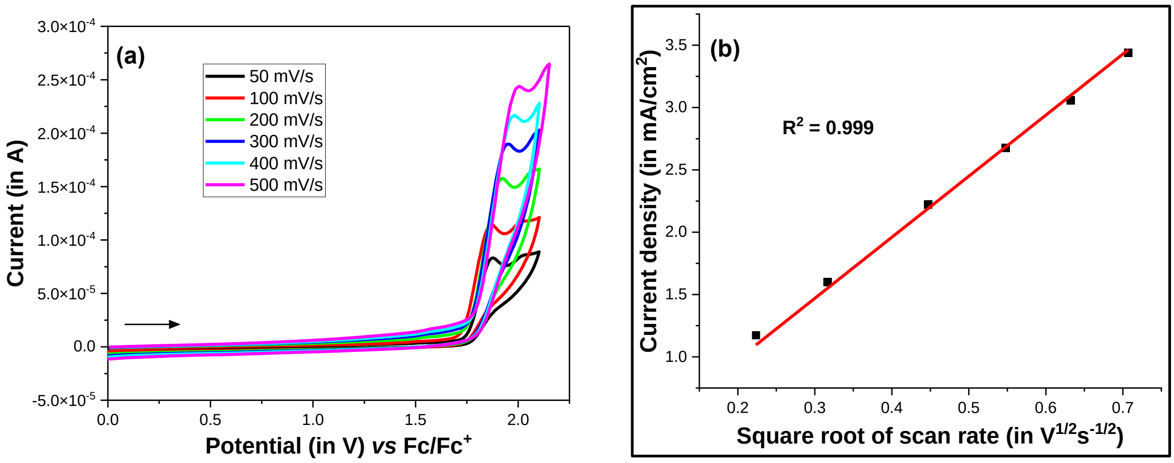

Figure S13: (a) The cyclic voltammogram at different scan rate in case of a solution containing **2a**, (b) The plot of current density vs square root of scan rate.

G. CV studies of PC_1 at different scan rates (reduction range) and plot of current density vs square of the scan rate (Randles–Ševčík equation):

A solution of **PC_1** (using 0.1 M $^{n}Bu_4NPF_6$ as a supporting electrolyte in MeCN) was subjected to CV experiment with different scan rates (50 mV/s, 100 mV/s, 200 mV/s, 300 mV/s, 400 mV/s, 500 mV/s). A plot of current density vs square root of scan rate was drawn (Figure S14).

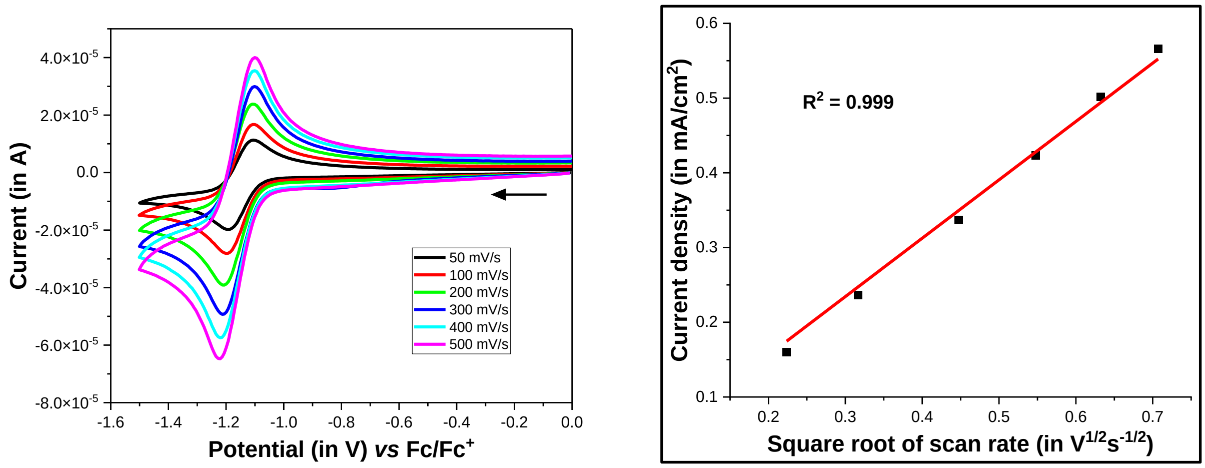

Figure S14: (a) The cyclic voltammogram at different scan rate in case of a solution containing $\mathbf{PC_1}$, (b) The plot of current density vs square root of scan rate (back scan only, considering $\mathbf{PC_1}^{\bullet-}$ to $\mathbf{PC_1}$).

Note: - Cyclic Voltammetry experiment of $\mathbf{PC_1}$ in case of the wave associated with one electron reduction at different scan rates (v) and plotting current density (i/A) as a function of the square root of the scan rate ($v^{1/2}$) revealed the electrochemical process to be diffusion-controlled[4] as a linear dependency of peak current on root of the scan rate ($v^{1/2}$) was observed. The similar pattern was observed in case of the oxidation peak when **1a** and **2a** was subjected to CV experiment.

10. Spectroelectrochemistry Studies:

A stock solution of **PC$_1$** (2.66 mM) was prepared in HPLC-grade MeCN with 0.01 (M) $TBAPF_6$ as supporting electrolyte. Spectroelectrochemistry was performed with a 50 µL aliquot of this solution (initial potential = -0.05 V, Upper vertex potential = 0 V, Lower vertex potential = −1.5 V, stopping potential = 0 V) with 50 mV/s scan rate and the in-situ UV-Vis spectra was recorded every 0.05 V change in potential. **PC$_1$** undergoes reversible 1e$^-$ reduction to form **PC$_1$$^{•-}$** and back to **PC$_1$** which can be seen in the absorption spectra with isosbestic points near 344 nm and 446 nm.

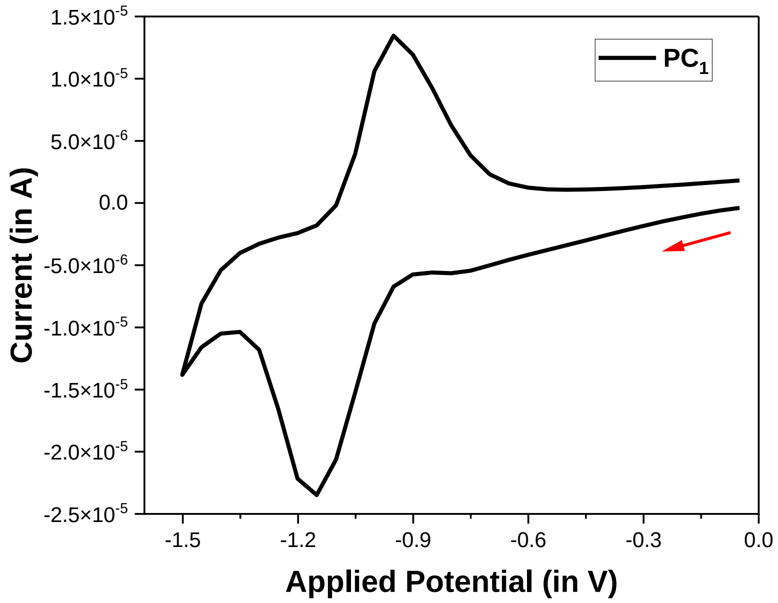

Figure S15: CV of 1e$^-$ reduction of **PC$_1$** coupled with in-situ UV-Vis for spectroelectrochemistry.

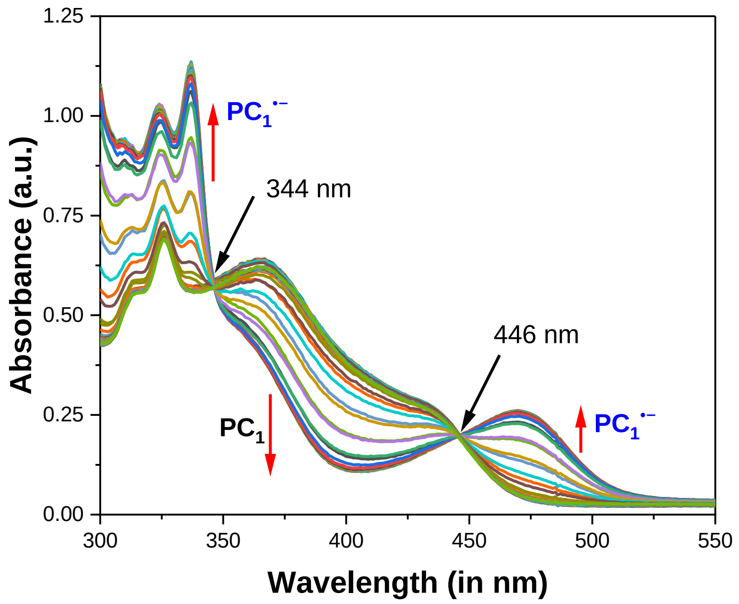

Figure S16: Spectroelectrochemical UV-Vis Absorption spectra of **PC$_1$** to **PC$_1$$^{•-}$** and back to **PC$_1$**.

11. UV-Visible Spectroscopic Studies:

A. Photostability of PC_1:

We initially investigated the photostability of the catalyst. The solution of $\mathbf{PC_1}$ (60µM) was prepared in DMF and the spectra was recorded. Next, the cuvette was irradiated with 456 nm Kessil lamp for 10 s consecutively and the spectra were recorded. From the spectra, it can be concluded that $\mathbf{PC_1}$ is quite stable under irradiation as there are no significant changes in the UV-Vis spectra on irradiation (Fig. S17).

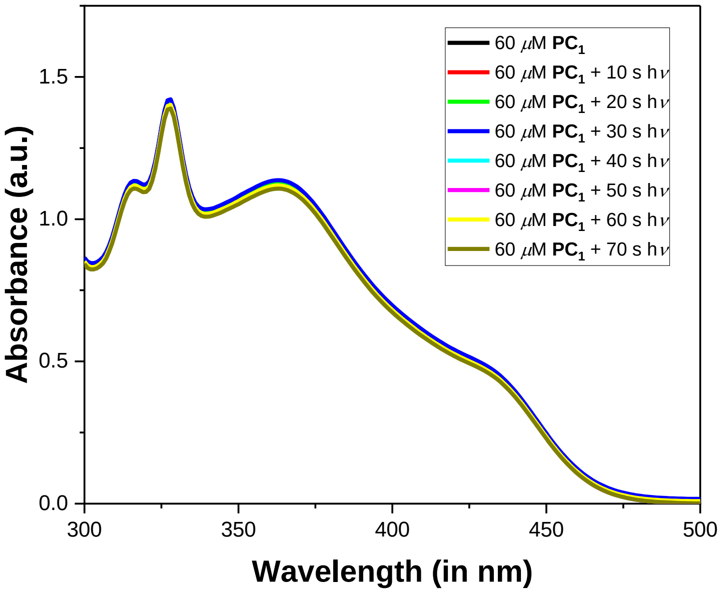

Figure S17: UV-Vis Absorption spectra of $\mathbf{PC_1}$ under consecutive light irradiations.

B. Validation of Lambert-Beer Law:

As we are exciting the $\mathbf{PC_1}$ using visible light of 456 nm wavelength, next, we planned to examine the validation of Lambert-Beer[5] law for $\mathbf{PC_1}$ at $\lambda = 456$ nm. For that purpose, five set of solutions having 33.33 μM, 40.00 μM, 46.67 μM, 53.33 μM and 60 μM concentrations of $\mathbf{PC_1}$, obtained by properly diluting an original stock solution of 0.01 M $\mathbf{PC_1}$ in DMF were introduced individually to a 1 cm path length quartz cuvette equipped with a Teflon® septum. Finally, the absorption spectra of these solutions were analysed (Fig. S18) using a UV-Vis spectrophotometer (Agilent Cary 4000 UV-Vis spectrophotometer).

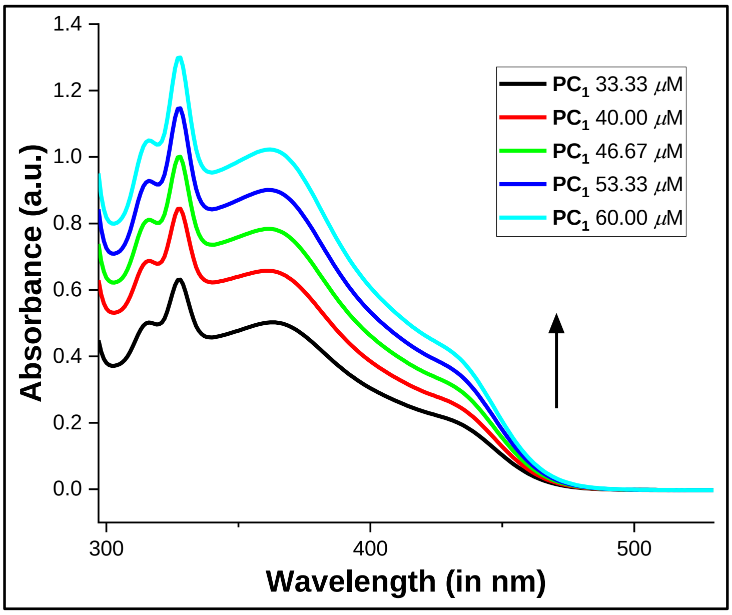

Figure S18: Absorbance *vs* wavelength plot for **PC₁** to satisfy Lambert-Beer law at λ = 456 nm.

From the absorption spectra, a linear correlation at λ = 456 nm was obtained when absorbance (a.u.) *vs* [**PC₁**] was plotted which evidently validates Lambert-Beer law (Fig. S19).

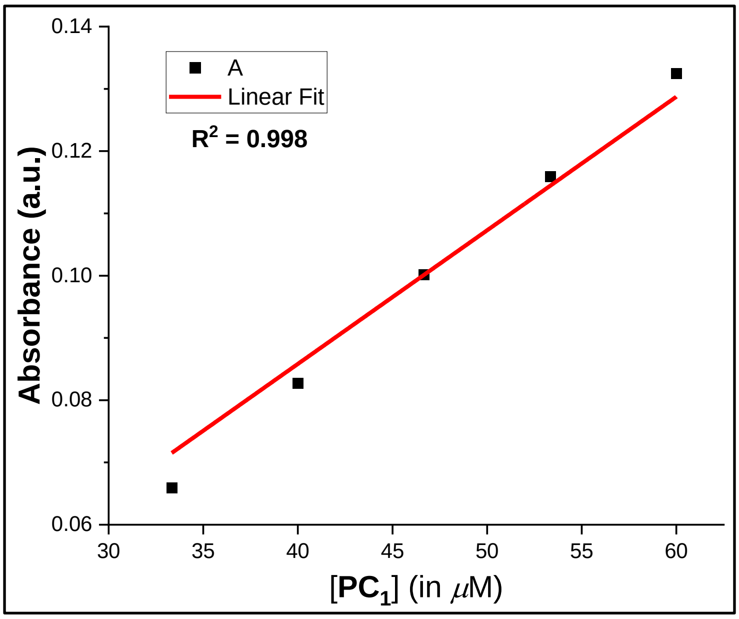

Figure S19: Plot of absorbance vs [**PC₁**].

C. Ground state quenching studies between PC₁ with 1a and 2a:

Initially, the solution of **PC₁** (50 µM) was prepared in DMF, and the spectra was recorded. Then, 1 mM, 2 mM, 3 mM, 4 mM, 5 mM, 6 mM, 7 mM, and 8 mM of **1a** was added sequentially to the 50 µM of PC and the spectra was recorded. No significant decrease in the absorbance spectra was noted (Fig. S20).

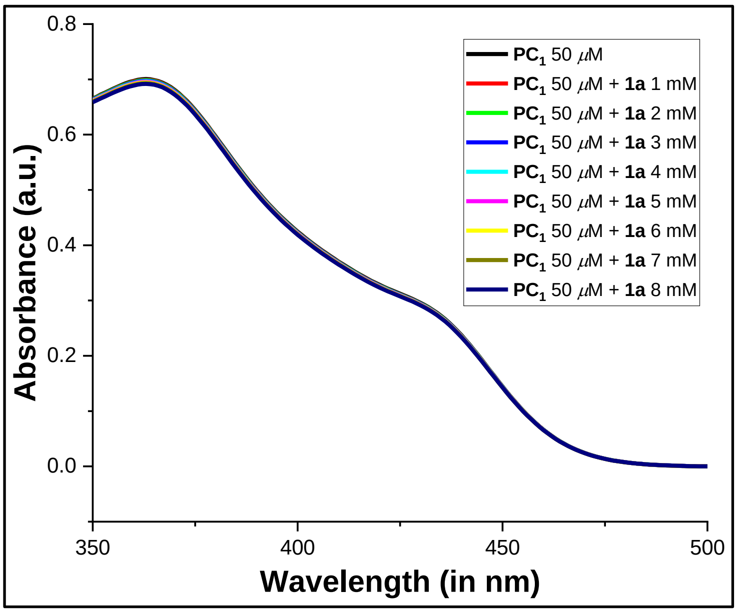

Figure S20: Absorption spectra of **PC**$_1$ with the sequential addition of **1a** in DMF.

Similarly, again the solution of **PC**$_1$ (50 µM) was prepared in DMF and then, 1 mM, 2 mM, 3 mM, 4 mM, 5 mM, 6 mM, 7 mM, and 8 mM of **2a** was added sequentially to the former solution and the spectra was recorded on each addition. In this case also, no significant decrease in the absorbance spectra was noted (Fig. S21).

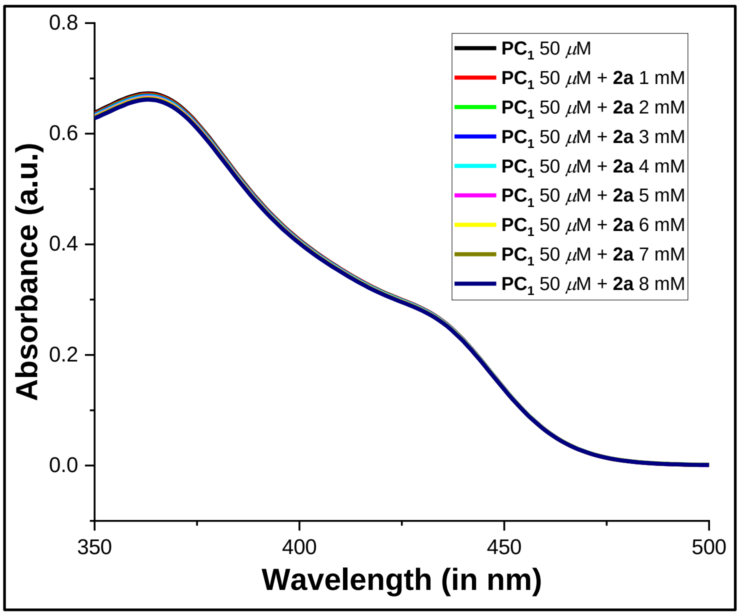

Figure S21: Absorption spectra of **PC**$_1$ with the sequential addition of **2a** in DMF.

D. Stacked UV-Vis spectra:

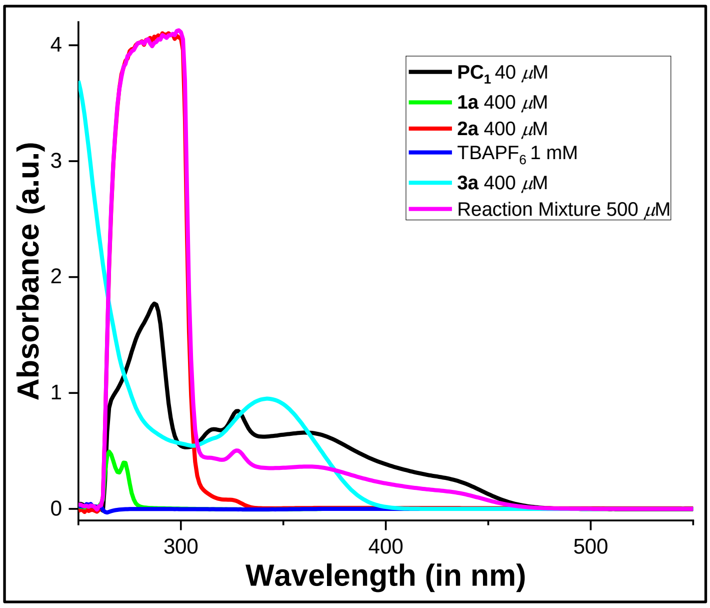

Figure S22: Stacked UV-Vis Absorption spectra of **PC_1**, **1a**, **2a**, $TBAPF_6$, **3a**, and the reaction mixture.

E. UV-Vis spectra of PC_1 and 1a (1:1) with light irradiation :

A solution of **PC_1** (60 μM) was prepared in DMF and then, 60 μM solution of **1a** was added to it and the spectra was recorded. Next, the screw-capped cuvette was irradiated with 456 nm Kessil lamp for 10 min intervals and the spectra were recorded. From the spectra, it can be concluded that **PC_1** oxidizes **1a** in the excited state and forms **$PC_1^{\bullet-}$**. The isosbestic point near 340 nm matches with the spectrum obtained in spectroelectrochemical data which corresponds to formation of **$PC_1^{\bullet-}$**.

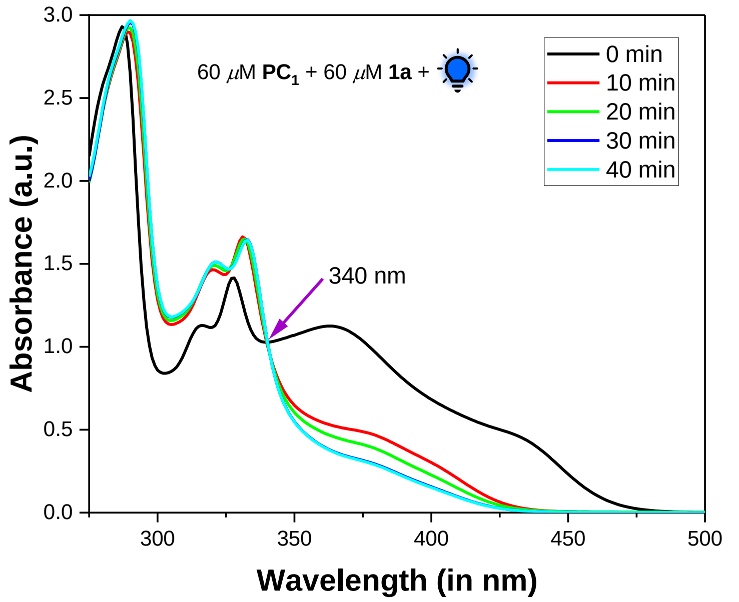

Figure S23: UV-Vis Absorption spectra of 1:1 mixture of **PC_1** and **1a** under consecutive light irradiation.

12. Fluorescence Studies:

To visualize the involvement of light for the photoelectrochemical annulation, the excited state quenching study of **PC_1** i.e., fluorescence quenching experiment was conducted. The components under consideration must satisfy the linear correlation obtained from Stern-Volmer plot[6] for successful quenching:

$$\frac{F_0}{F} = 1 + K_{SV}[\boldsymbol{Q}]$$

Where, F_0 = Fluorescence intensity in absence of quencher, F = Fluorescence intensity in presence of quencher, K_{SV} = Stern-Volmer constant, [**Q**] = concentration of quencher.

At first, the fluorescence emission intensity of a solution containing 1.5 μM **PC_1** (in DMF) in 1 cm path length quartz cuvette equipped with a Teflon® septum was recorded by exciting at 450 nm and data was collected from 465 nm to 700 nm. Then to the solution of 1.5 μM **PC_1** sequential addition of 0.15 mM, 0.30 mM, 0.45 mM, 0.60 mM, 0.75 mM, 0.90 mM, and 1.05 mM **1a** (in DMF) results in a steady decrease in the fluorescence intensity (fluorescence quenching) after each addition (Fig. S24).

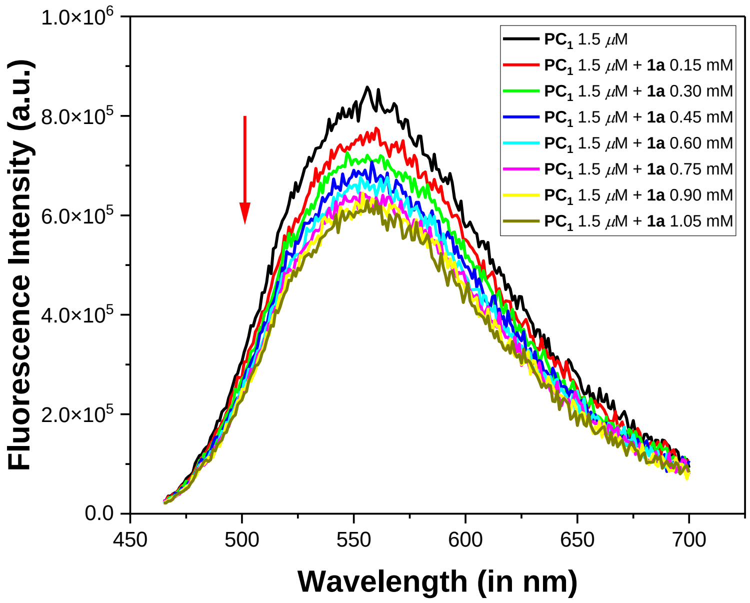

Figure S24: Fluorescence quenching study by sequential addition of **1a** to a solution **PC_1** in DMF.

Similarly, again the fluorescence emission intensity of a solution containing 1.5 μM **PC_1** (in DMF) in 1 cm path length quartz cuvette equipped with a Teflon® septum was recorded by exciting at 450 nm and data was collected from 465 nm to 700 nm. Then to the solution of 1.5 μM **PC_1** sequential addition of 0.15 mM, 0.30 mM, 0.45 mM, 0.60 mM, 0.75 mM, 0.90 mM, and 1.05 mM **2a** (in DMF) results in almost negligible decrease in the fluorescence intensity (fluorescence quenching) after each addition (Fig. S25).

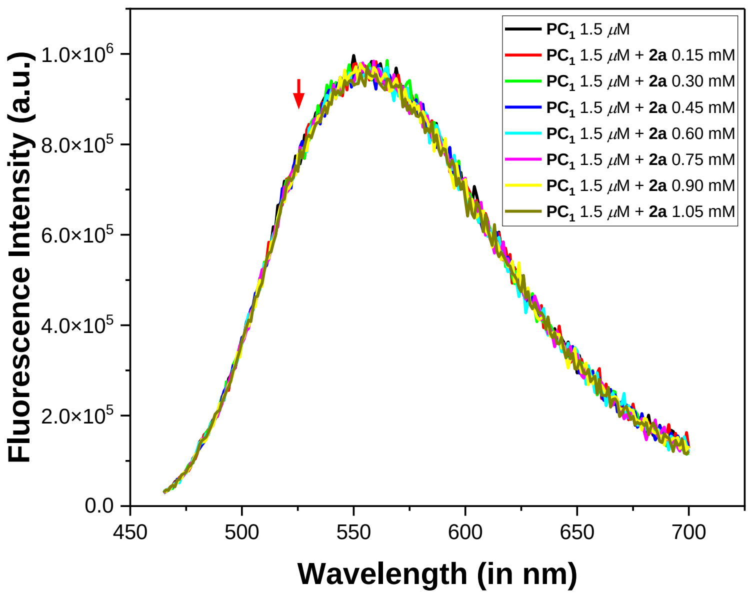

Figure S25: Fluorescence quenching study by sequential addition of **2a** to a solution $\mathbf{PC_1}$ in DMF.

From the data sets obtained, a plot of F_0/F vs [Quencher] was drawn (Fig. S26).

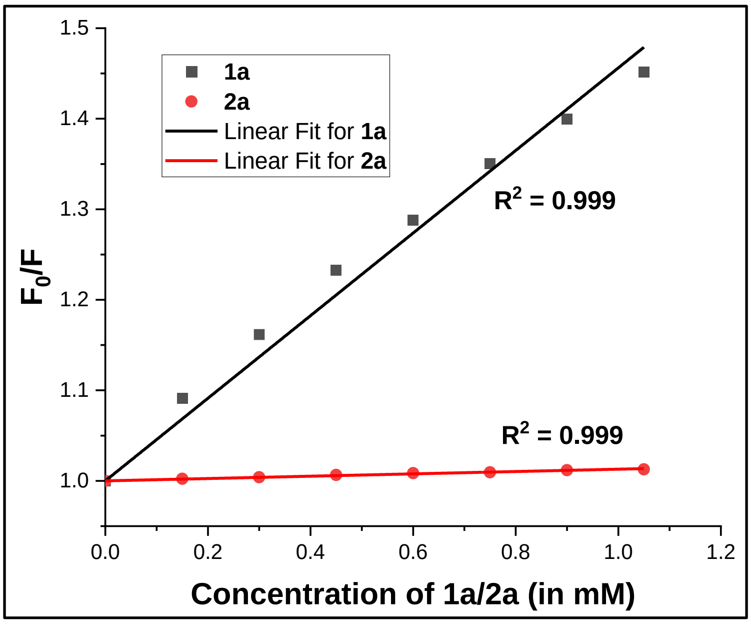

Figure S26: Stern-Volmer plot of F_0/F *vs* [Quencher] in DMF.

It is evident from the Stern-Volmer plot that **1a** is a better quencher for the excited state of $\mathbf{PC_1}$ as compared to that of **2a**.

13. Electron Paramagnetic Resonance (EPR) Analysis:

Continuous wave (CW) EPR spectra were obtained using a Bruker MagnetTech MiniScope MS 400 benchtop EPR spectrometer instrument with X-band of 9.30-9.55 GHz. The spectral data was collected

at room temperature with the following spectrometer settings: microwave power = 0.48 mW, center field = 335.87 mT , sweep width = 70.65 mT, sweep time = 30 s, modulation frequency = 9.5 GHz, modulation amplitude = 0.1 mT, MW attenuation = 20 db.

For all the EPR measurements, the corresponding sample solution was transferred into the EPR tube. After that, the sample tube was inserted in the EPR cavity at room temperature for the recording of the spectra. For experiments in which the sample was irradiated, the sample tube was kept at 4 cm distance from the 456 nm Kessil lamp.

An oven dried 10 mL crimp cap glass vial equipped with magnetic stirring bar was charged with **1a** (80.8 mg, 0.4 mmol), **2a** (35.6 mg, 0.2 mmol) and **PC₁** (8 mg, 5 mol%). Exact 4 mL of DMF was added to the vial and crimped. Next, the vial was placed into a magnetic stirrer and stirred for 10 minutes to ensure homogeneity. Then, from the reaction mixture, 500 μL solution was transferred into the EPR tube and EPR spectra was recorded (Fig S27, black line). Then the tube containing reaction mixture was irradiated for 1h. Instantly after irradiation, the EPR spectra was recorded in a similar fashion as previous (Fig S27, red line). (g value = 2.004)

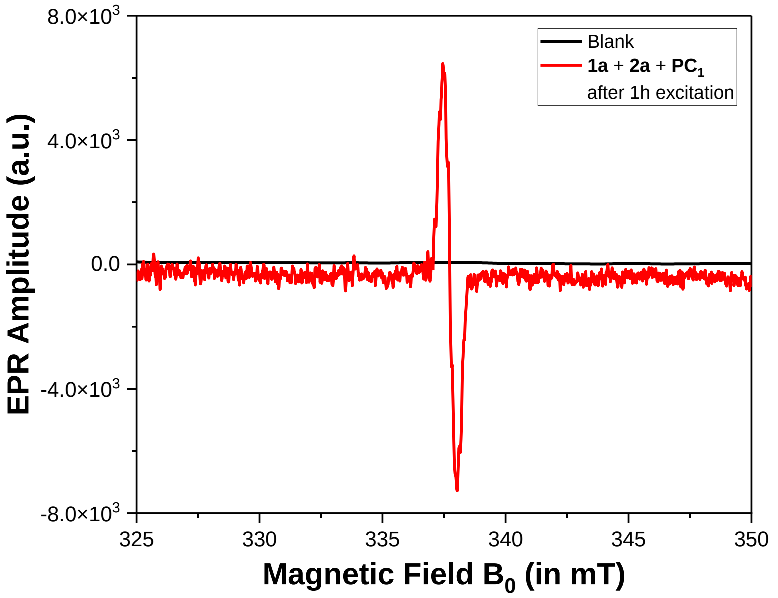

Figure S27: EPR analysis of $PC^{\bullet -}$.

Another oven dried 5 mL crimp cap glass vial equipped with magnetic stirring bar was charged with **1a** (20.2 mg, 0.1 mmol), 5,5-dimethyl-1-pyrroline-*N*-oxide (DMPO, 22.4 µL, 0.2 mmol) and **PC₁** (4 mg, 5 mol%). Exact 2 mL of DMF was added to the vial after removing the air and purging it with Ar and crimped. Next, the vial was placed into a magnetic stirrer and stirred for 10 minutes to ensure homogeneity. Then, from the reaction mixture, 500 μL solution was transferred into the EPR tube and EPR spectra was recorded (Fig S28, black line). Then the tube containing reaction mixture was

irradiated for 1h. Instantly after irradiation, the EPR spectra was recorded in a similar fashion as previous (Fig S28, blue line).

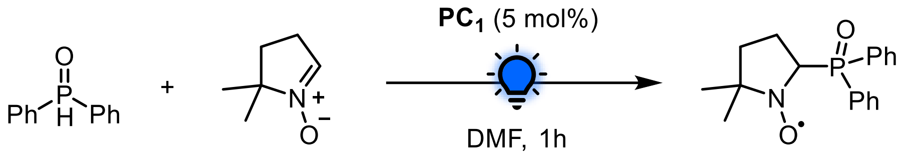

1a (0.1 mmol)   DMPO (2 equiv.)

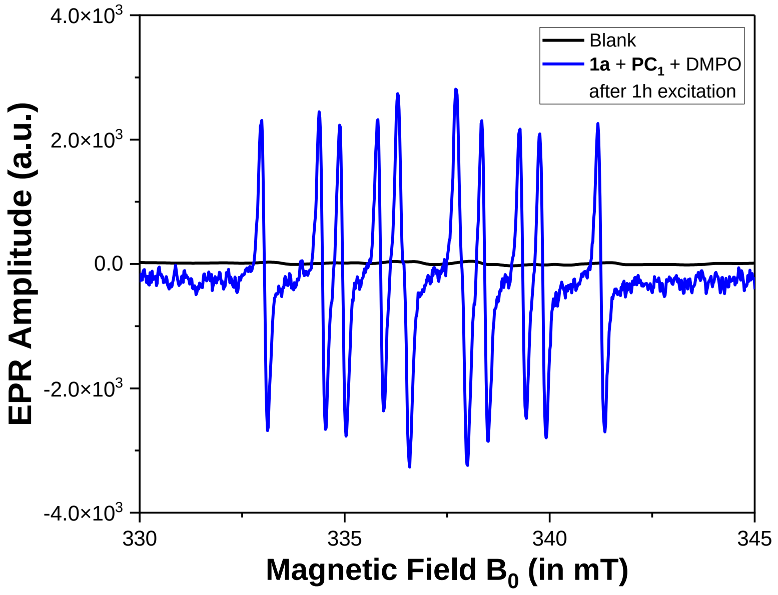

Figure S28: EPR analysis of DMPO trapped phosphinoyl radical.

14. Radical Inhibition Experiments:

An oven dried ElectraSyn vial (5 mL) equipped with magnetic stirring bar was charged with **1a** (0.4 mmol), **2a** (0.2 mmol), **PC₁** (5 mol%, 0.01 mmol), BHT (3.0 equiv., 0.6 mmol) or TEMPO (3.0 equiv., 0.6 mmol) and $TBAPF_6$ (1.0 equiv., 0.2 mmol). Exact 4 mL of degassed DMF was added to the ElectraSyn vial. An ElectraSyn vial cap equipped with Graphite SK-50 as an anode and IKA Pt foil as cathode was then fitted tightly to the reaction vial.

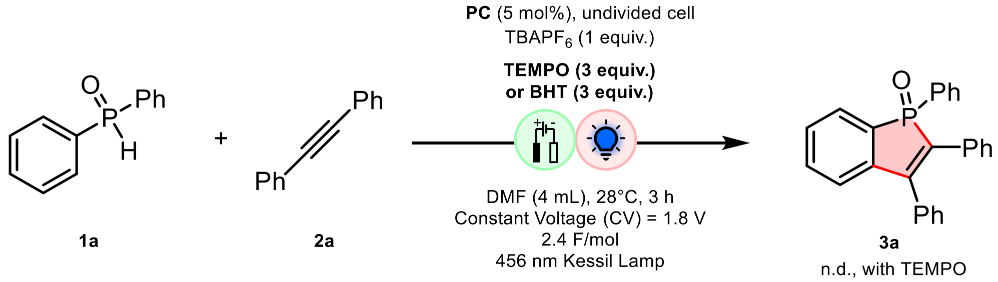

1a   **2a**   **3a**

n.d., with TEMPO
trace, with BHT

The whole reaction mixture was the stirred vigorously for 10 minutes to make the mixture homogenous. After 10 minutes of pre-stirring, vial was purged with Ar gas thrice. Then the complete setup was connected to the vial holder of ElectraSyn 2.0 and placed at a distance of 3 cm under irradiation with a Kessil® PR160-456 nm lamp equipped with an external cooling device for maintaining the ambient temperature throughout the course of the reaction. The reaction mixture was simultaneously electrolysed for 3 h by applying +1.8 V constant potential (2.4 F/mol) with 1200 rpm magnetic stirring (Fig. S3). After completion of the reaction, 100 μL aliquot was taken out from the reaction mixture and diluted with 900 μL MeCN and finally subjected for HRMS analysis. Only trace amounts of product were obtained in both the cases and the adducts were detected in HRMS.

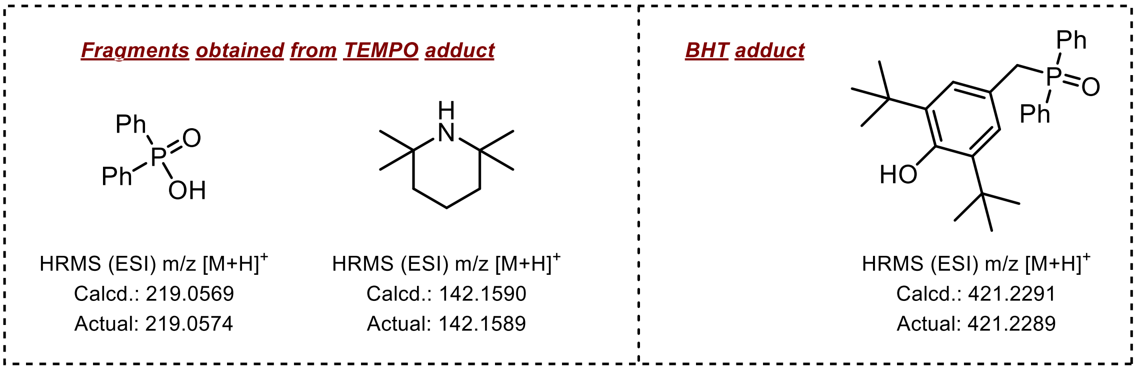

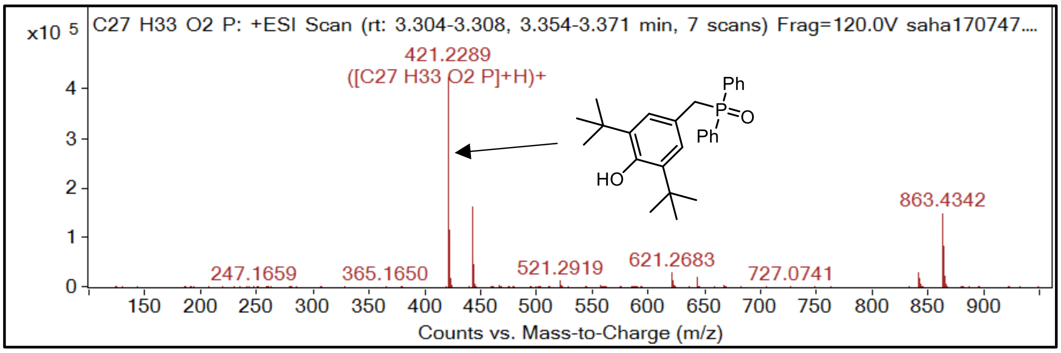

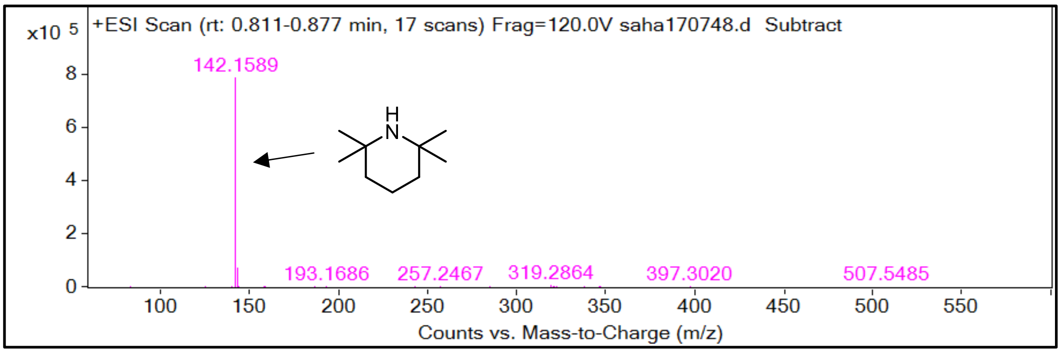

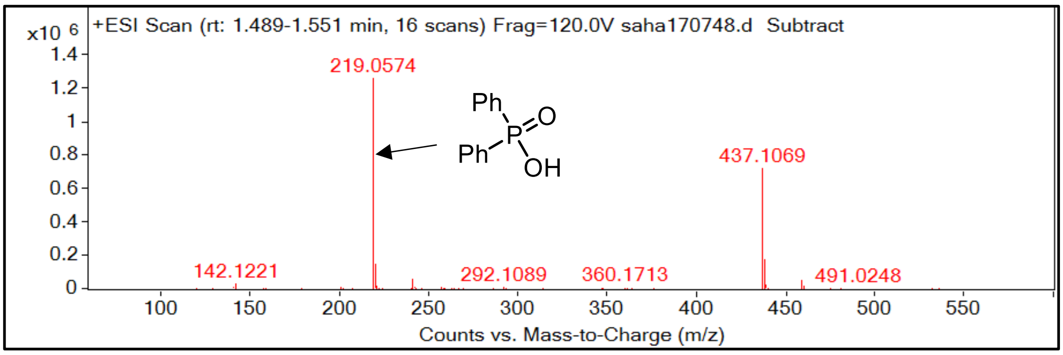

Figure S29: HRMS (ESI-TOF) reports of the adducts and fragments obtained.

15. Light On-Off and Electricity On-Off Experiment:

Initially, an oven dried ElectraSyn vial (5 mL) equipped with magnetic stirring bar was charged with **1a** (0.4 mmol), **2a** (0.2 mmol), **PC$_1$** (5 mol%, 0.01 mmol), and TBAPF$_6$ (1.0 equiv., 0.2 mmol). Exact 4 mL of degassed DMF was added to the ElectraSyn vial. An ElectraSyn vial cap equipped with Graphite SK-50 as an anode and IKA Pt foil as cathode was then fitted tightly to the reaction vial. The whole reaction mixture was the stirred vigorously for 10 minutes to make the mixture homogenous. After 10 minutes of pre-stirring, vial was purged with argon gas thrice. Then the complete setup was connected to the vial holder of ElectraSyn 2.0 and placed at a distance of 3 cm under irradiation with a Kessil® PR160-456 nm lamp equipped with an external cooling device for maintaining the ambient temperature throughout the course of the reaction. The reaction mixture was simultaneously electrolysed at +1.8 V constant potential with 1200 rpm magnetic stirring (Fig. S3). After 15 min irradiation (**light on-electricity on**), an aliquot portion (20 μL) was taken out from the reaction mixture and quenched with 100 μL CH_3CN. The solution was then subjected to GC-FID analysis using benzophenone as internal standard to check the yield of **3a**. After that, the electrolysis was continued in the dark condition (**light off-electricity on**) under continuous stirring for another 15 mins. Again, the analytical solution was prepared, and yield was determined. This process was repeated for 1.5 h and the yield of **3a** was plotted with respect to time (Fig. S30). The nature of the graph indicates that the dehydrogenative annulation process is light dependent as negligible formation of desired product was observed in the absence of the light.

Entry	Time (in min)	Yield of 3a (%)
1	15	31.24
2	30	31.92
3	45	53.07
4	60	53.79
5	75	73.61
6	90	74.03

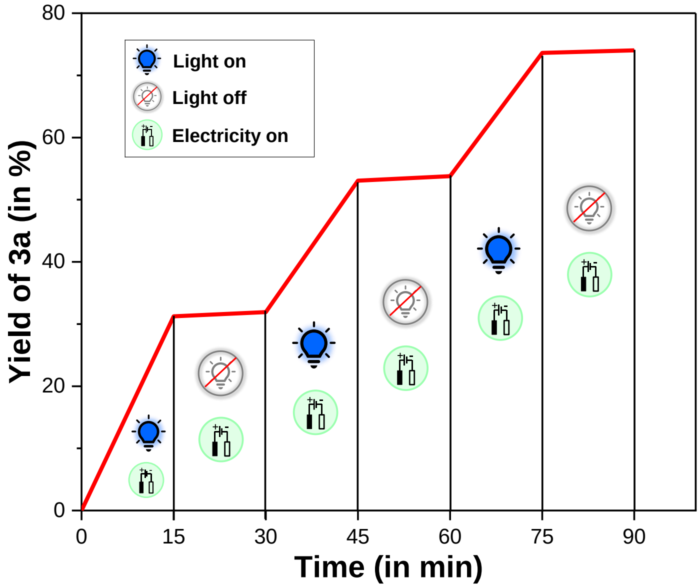

Figure S30: Electricity on during the course of reaction and light on-off.

Next, an oven dried ElectraSyn vial (5 mL) equipped with magnetic stirring bar was charged with **1a** (0.4 mmol), **2a** (0.2 mmol), **PC$_1$** (5 mol%, 0.01 mmol), and TBAPF$_6$ (1.0 equiv., 0.2 mmol). Exact 4 mL of degassed DMF was added to the ElectraSyn vial. An ElectraSyn vial cap equipped with Graphite SK-50 as an anode and IKA Pt foil as cathode was then fitted tightly to the reaction vial. The whole reaction mixture was the stirred vigorously for 10 minutes to make the mixture homogenous. After 10 minutes of pre-stirring, vial was purged with argon gas thrice. Then the complete setup was connected to the vial holder of ElectraSyn 2.0 and placed at a distance of 3 cm under irradiation with a Kessil® PR160-456 nm lamp equipped with an external cooling device for maintaining the ambient temperature throughout the course of the reaction. The reaction mixture was simultaneously electrolysed at +1.8 V constant potential with 1200 rpm magnetic stirring (Fig. S3). After 15 min irradiation (**electricity on-light on**), an aliquot portion (20 μL) was taken out from the reaction mixture and quenched with 100 μL CH_3CN. The solution was then subjected to GC-FID analysis using benzophenone as internal standard to check the yield of **3a**. After that, the light irradiation was continued, and electricity was turned off (**electricity off- light on**) under continuous stirring for another 15 mins. Again, the analytical solution was prepared, and yield was determined. This process was repeated for 1.5 h and the yield of **3a** was plotted with respect to time (Fig. S31). The nature of the graph indicates that the

dehydrogenative annulation process is also dependent on electricity as negligible formation of desired product was observed in the absence of the light.

Entry	Time (in min)	Yield of 2a (%)
1	15	33.34
2	30	37.92
3	45	53.07
4	60	59.79
5	75	73.61
6	90	77.03

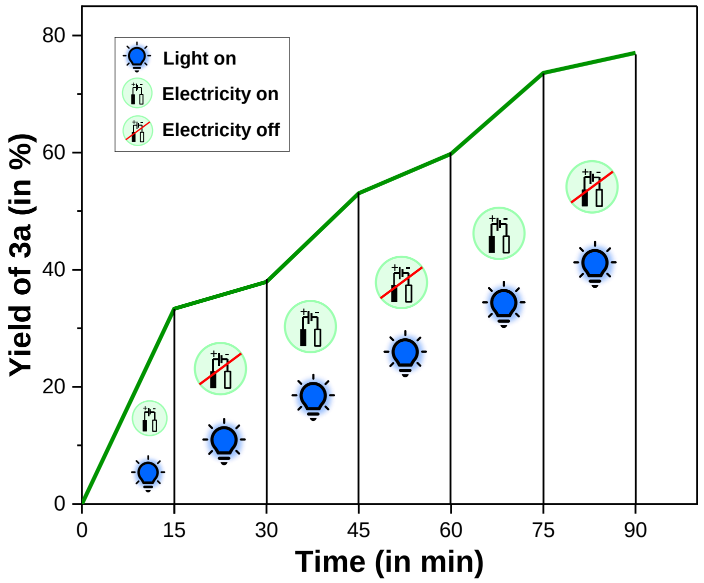

Figure S31: Light on during the course of reaction and electricity on-off.

16. Reusability of the Photocatalyst and the Electrodes (one pot batch scale up):

Following the General Procedure for Photoelectrochemical Annulation, three consecutive cycles were observed for single loading of **PC_1**. After completion of each cycle, another 0.4 mmol of the **1a**, 0.2 mmol of **2a** and required amount of electrolyte (~ 20 mol%, to adjust the resistance of the solution, so that the photoelectrochemical process doesn't stop) was introduced into the same reaction vial. GC yields of the desired product **3a** were reported for the three consecutive cycles.

Cycle	Yield of 2a (%)
1	91
2	85
3	82

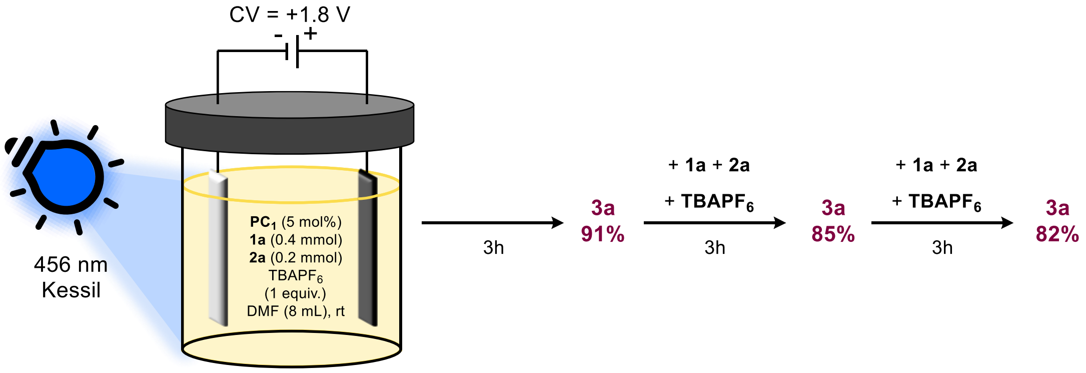

Figure S32: One-pot sequential batch scale up and sustainability of the photocatalyst.

17. Detection of H_2 gas evolution (Headspace GC-TCD analysis):

Initially, an oven dried ElectraSyn vial (5 mL) equipped with magnetic stirring bar was charged with **1a** (0.4 mmol), **2a** (0.2 mmol), **PC$_1$** (5 mol%, 0.01 mmol), and TBAPF$_6$ (1.0 equiv., 0.2 mmol). Exact 4 mL of degassed DMF was added to the ElectraSyn vial. An ElectraSyn vial cap equipped with Graphite SK-50 as an anode and IKA Pt foil as cathode was then fitted tightly to the reaction vial covered with teflon film. The whole reaction mixture was the stirred vigorously for 10 minutes to make the mixture homogenous. After 10 minutes of pre-stirring, vial was purged with argon gas thrice and sealed properly with parafilm. Then the complete setup was connected to the vial holder of ElectraSyn 2.0 and placed at a distance of 3 cm under irradiation with a Kessil® PR160-456 nm lamp equipped with an external cooling device for maintaining the ambient temperature throughout the course of the reaction. The reaction mixture was simultaneously electrolysed at +1.8 V constant potential with 1200 rpm magnetic stirring (Figure. S3). After completion of the reaction, 1 mL gas volume of the headspace was carefully taken in a gas syringe and analyzed in GC-TCD to detect H_2 gas. (Peak at 0.479 min)

For the standard H_2 peak observation and comparison, 0.2 mmol of sodium borohydride was taken in an oven dried crimp-cap vial, sealed and evacuated, followed by purging with Ar gas. 15 μL of distilled water (excess) was then added quickly using a Hamilton microsyringe avoiding any loss of H_2 gas evolved from the instant hydrolysis of $NaBH_4$. 1 mL gas volume of the headspace was then carefully taken in a gas syringe and analyzed in GC-TCD to detect H_2 gas. (Peak at 0.478 min)

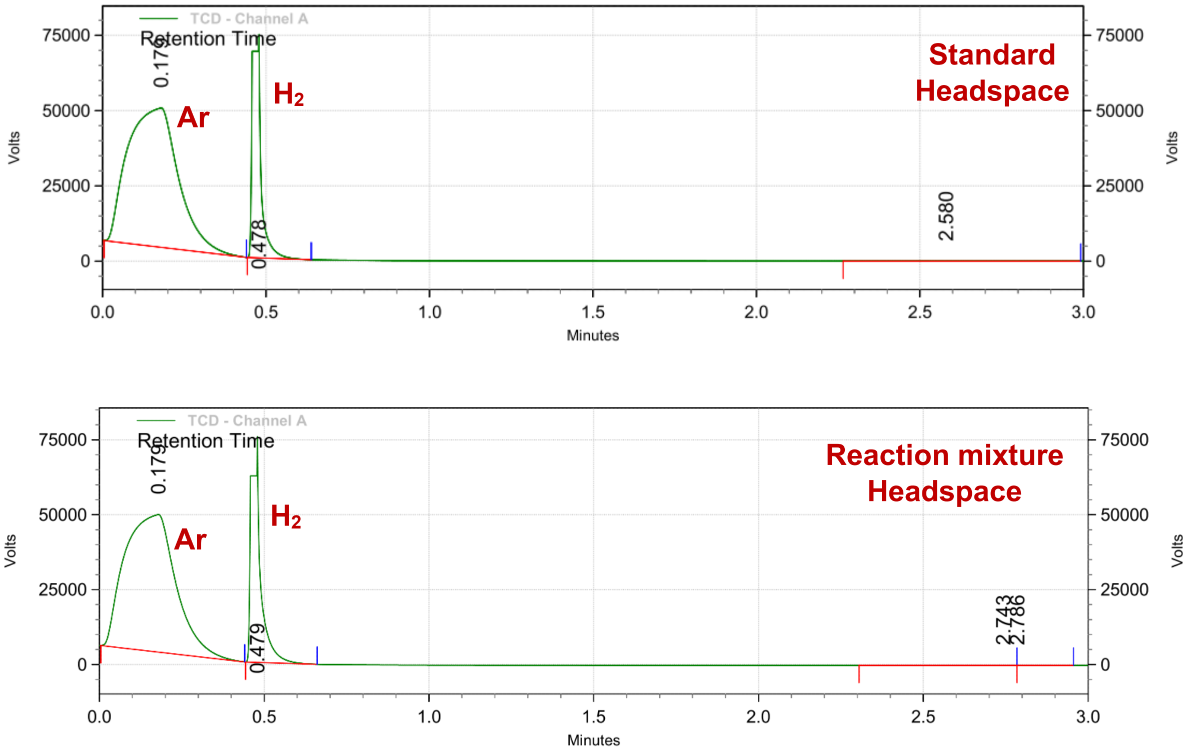

Figure S33: Headspace GC-TCD chromatograms for the standard and reaction mixture.

18. Kinetic Isotopic Effect Experiment:

In order to get information about the rate determining step of the photoelectrochemical protocol, we performed the reaction with diphenylphosphine oxide (1a) and diphenyldeuterophosphine oxide (1a-d_1) separately according to the general procedure, for following the initial rates of the two reactions.

For the preparation of diphenyldeuterophosphine oxide, chlorodiphenylphosphine (1g, 4.5 mmol) was taken in a 10 mL oven dried crimp-cap vial, dissolved in 3 mL of acetonitrile-d_3 (CD_3CN) and cooled to 0°C followed by addition of excess amount of D_2O (2 mL) under argon atmosphere and crimped. The contents were warmed to 60°C for 8 h. After cooling down to room temperature, the mixture was extracted with DCM (3 × 5 mL) and saturated $NaHCO_3$ solution in D_2O (1 mL). The combined organic phases were dried over anhydrous Na_2SO_4 and concentrated in vacuo. The residue was purified by column chromatography using ethyl acetate in hexane (1:1) as eluent to furnish 1a-d_1 as a white solid.

The percentage of D-incorporation was found to be **83.55%**.

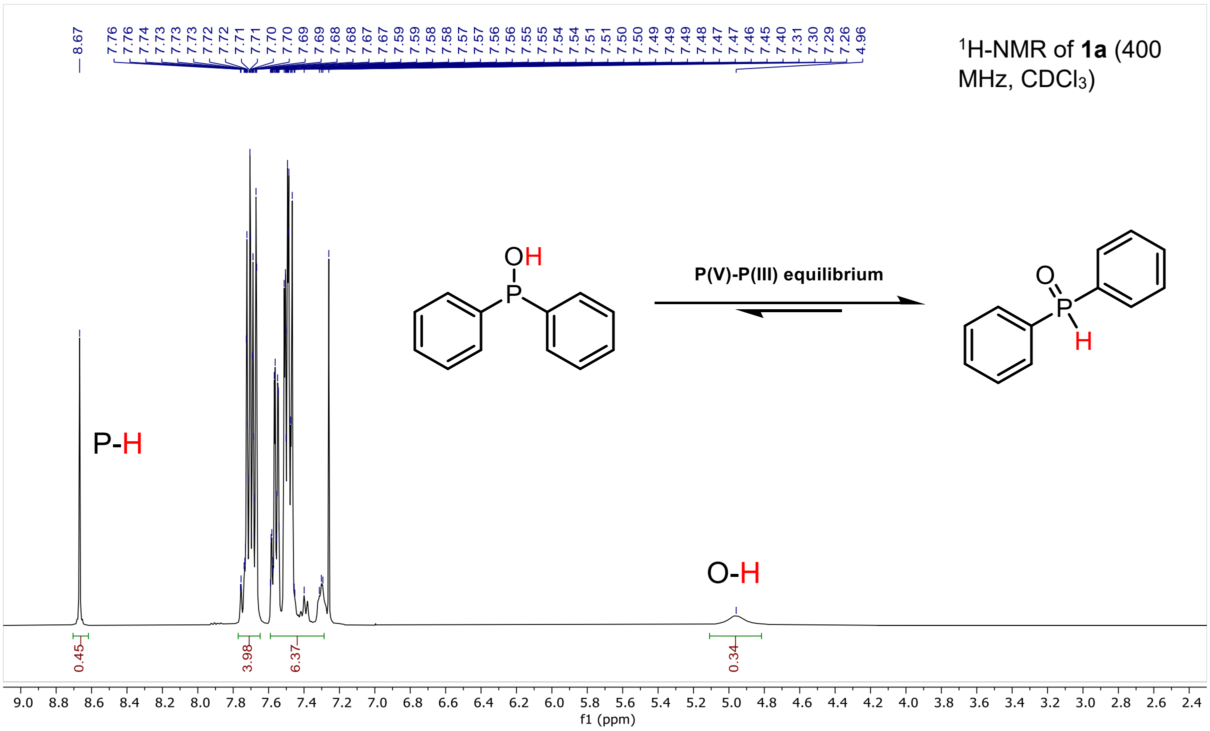

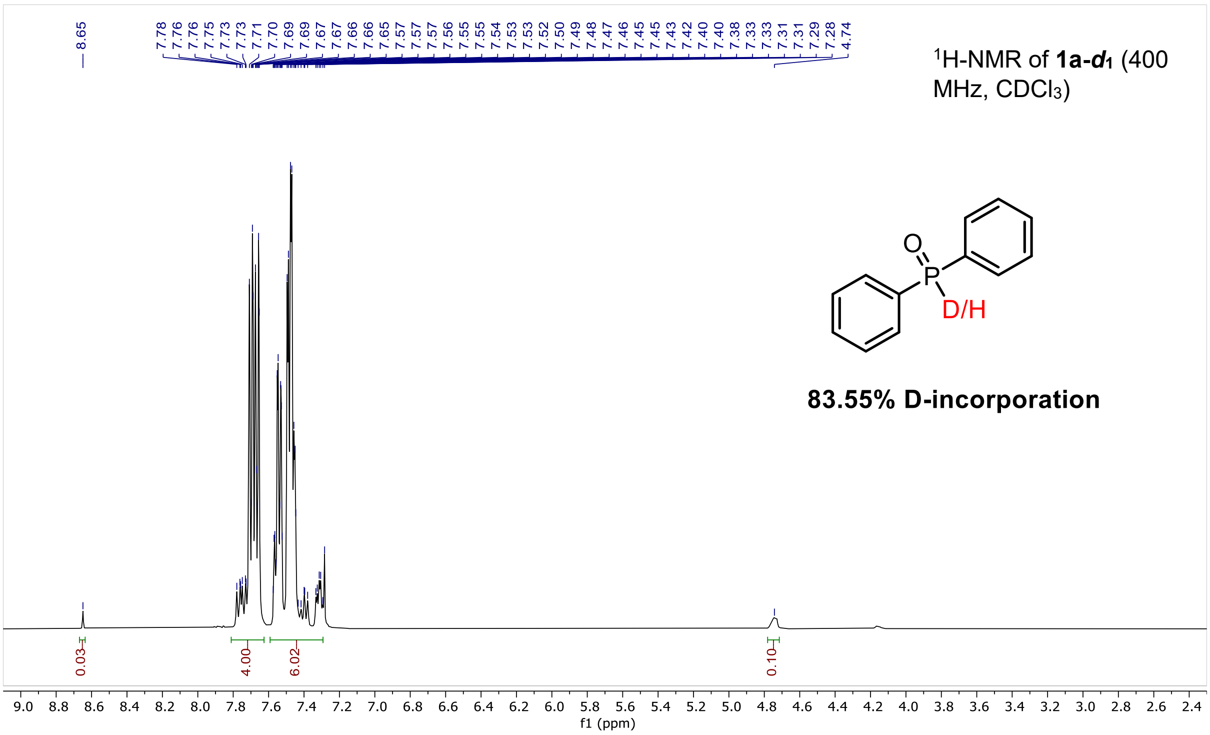

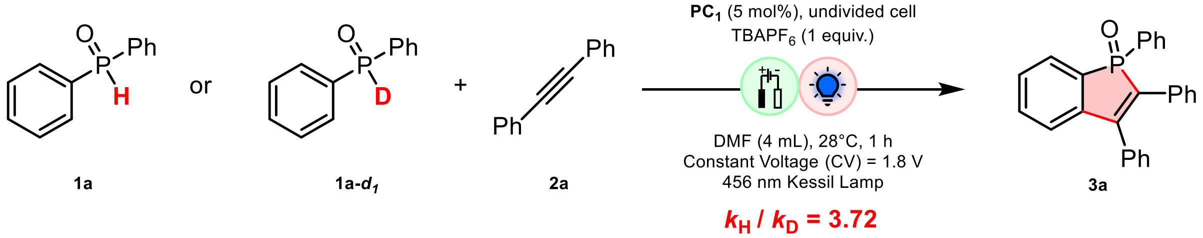

Time (in min)	Yield of 3a (%)	
	Using **1a**	Using **1a-*d*₁**
0	0	0
15	17	5
30	26	7
45	39	10
60	51	14

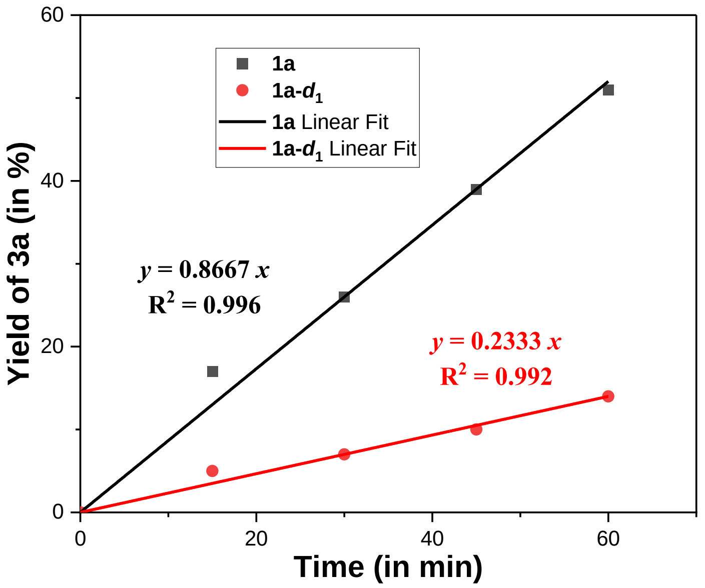

Figure S34: Kinetic isotopic effect experiment plots.

The ratio of k_H/k_D has been found to be 3.72 which suggests that the breaking of P-H bond is involved in the rate-determining step of the reaction indicating Primary Kinetic Isotopic effect.

19. Photophysical Properties of some Benzo[*b*]phosphole Oxide Products:

The Absorption and Emission spectra are recorded by dissolving some of the products in HPLC-grade CH_3CN.

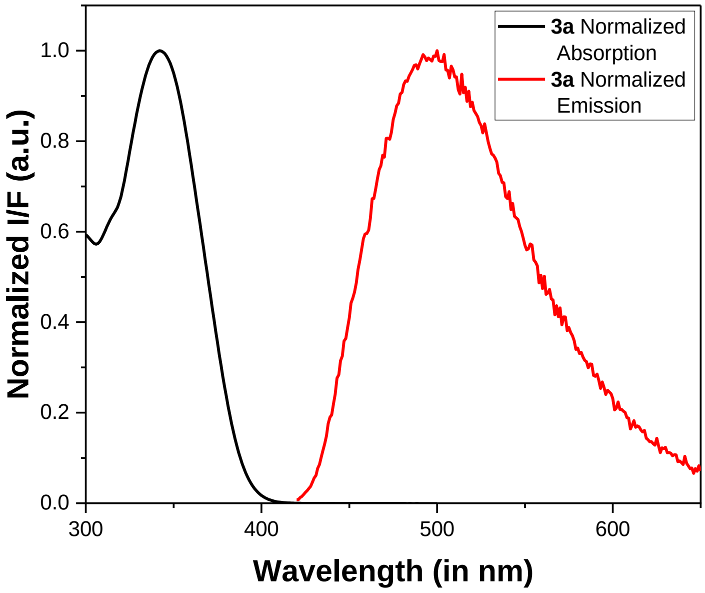

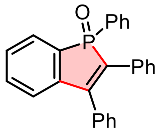

3a

Figure S35: Normalized Absorption and Emission spectra of **3a** in CH_3CN. (Excitation λ = 350 nm)

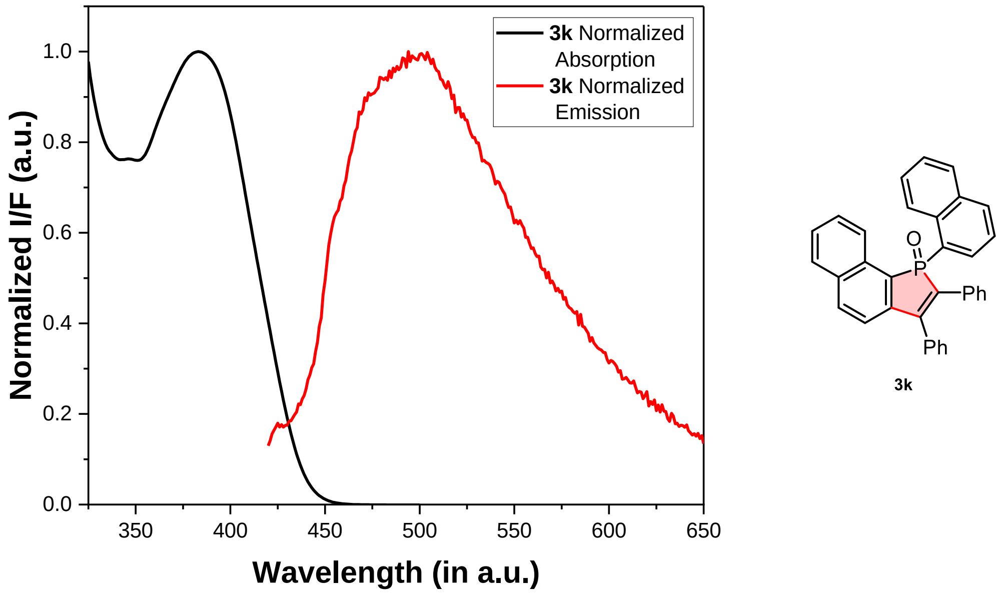

Figure S36: Normalized Absorption and Emission spectra of **3k** in CH_3CN. (Excitation λ = 400 nm)

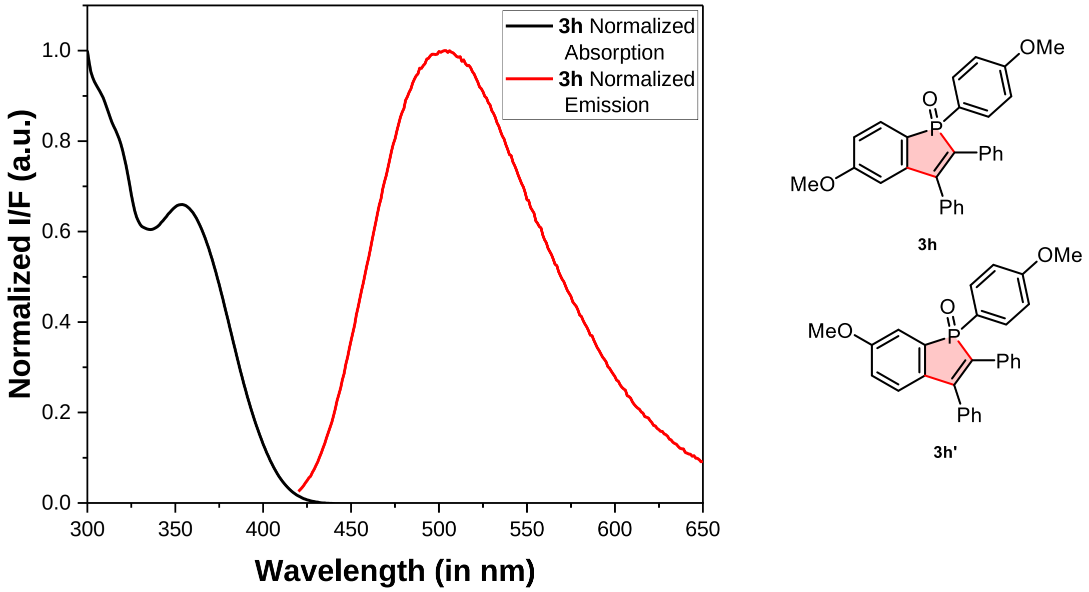

Figure S37: Normalized Absorption and Emission spectra of **3h**, **3h'** in CH_3CN. (Excitation λ = 400 nm)

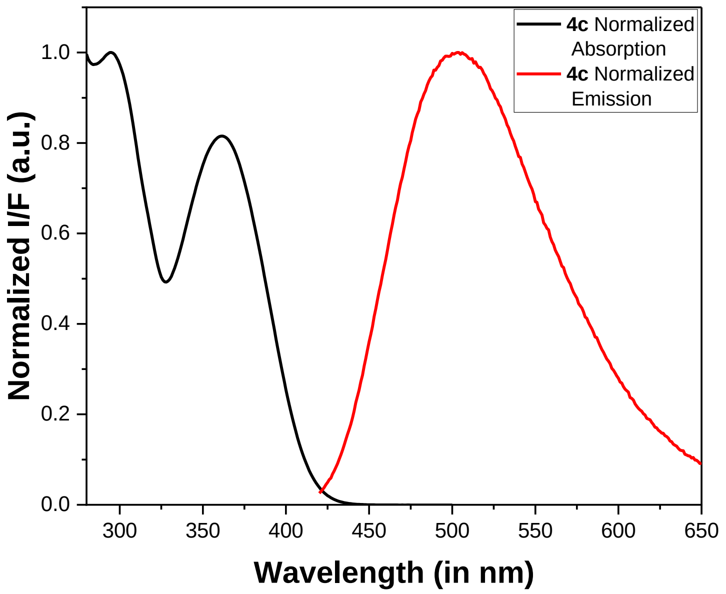

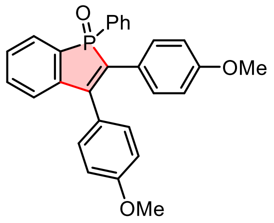

4c

Figure S38: Normalized Absorption and Emission spectra of **4c** in CH_3CN. (Excitation λ = 400 nm)

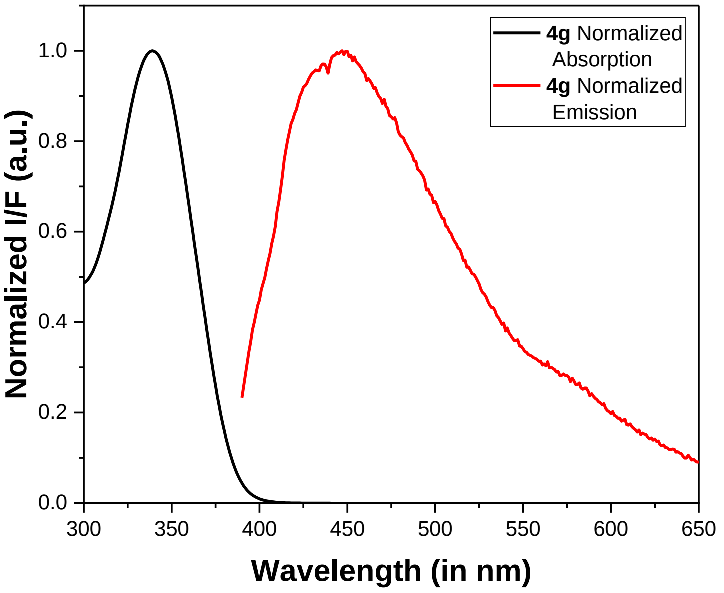

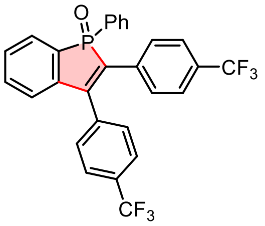

4g

Figure S39: Normalized Absorption and Emission spectra of **4g** in CH_3CN. (Excitation λ = 375 nm)

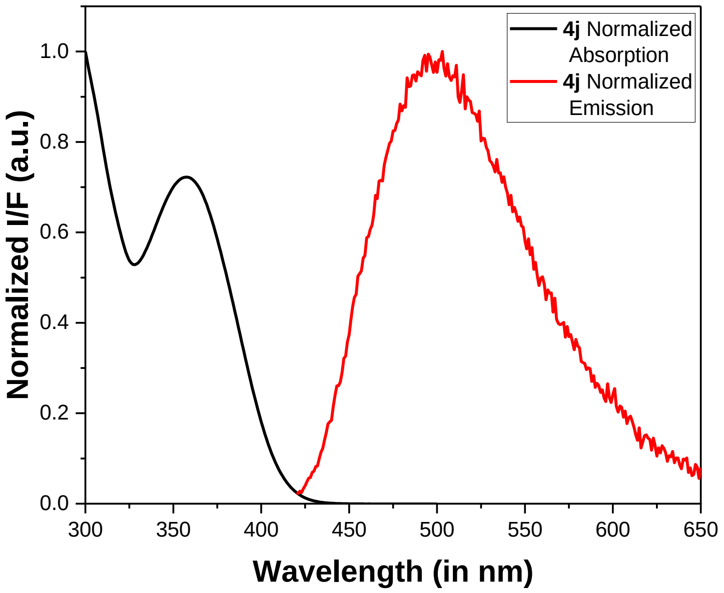

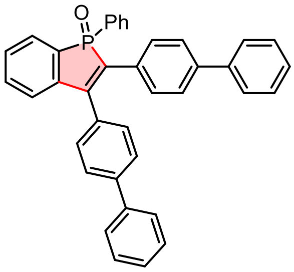

4j

Figure S40: Normalized Absorption and Emission spectra of **4j** in CH_3CN. (Excitation λ = 400 nm)

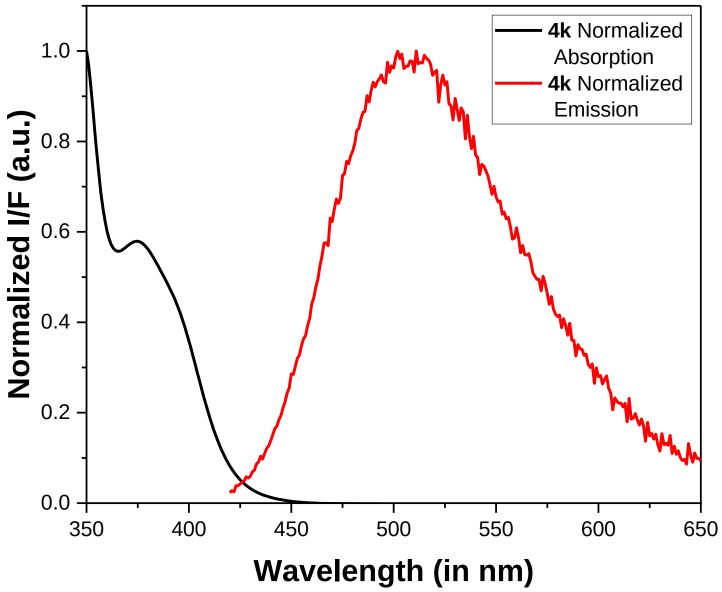

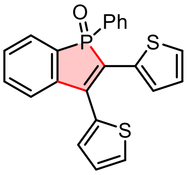

4k

Figure S41: Normalized Absorption and Emission spectra of **4k** in CH_3CN. (Excitation λ = 400 nm)

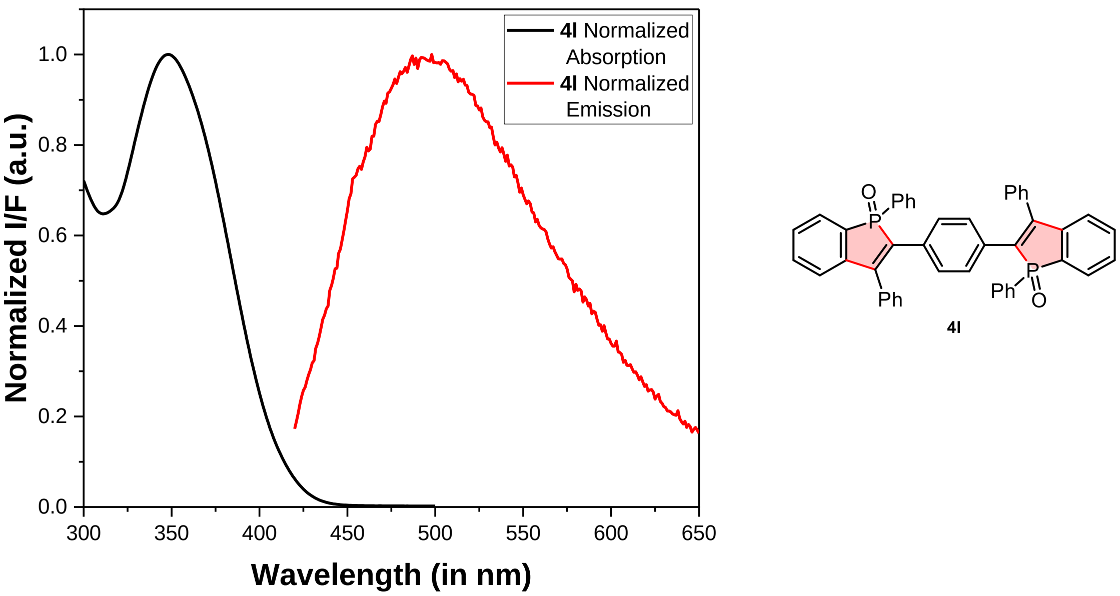

Figure S42: Normalized Absorption and Emission spectra of **4l** in CH_3CN. (Excitation λ = 400 nm)

20. Intermolecular Competition Experiments:

Following the General Procedure, intermolecular competition experiments were conducted by using (a) different diphenyl acetylenes with electron-rich and deficient functional groups; (b) aromatic substituted and aliphatic substituted alkynes; (c) diphenylphosphine oxide and ethyl phenylphosphine oxide. The ratios of the products obtained was determined from ^{31}P NMR spectra of the crude mixtures.

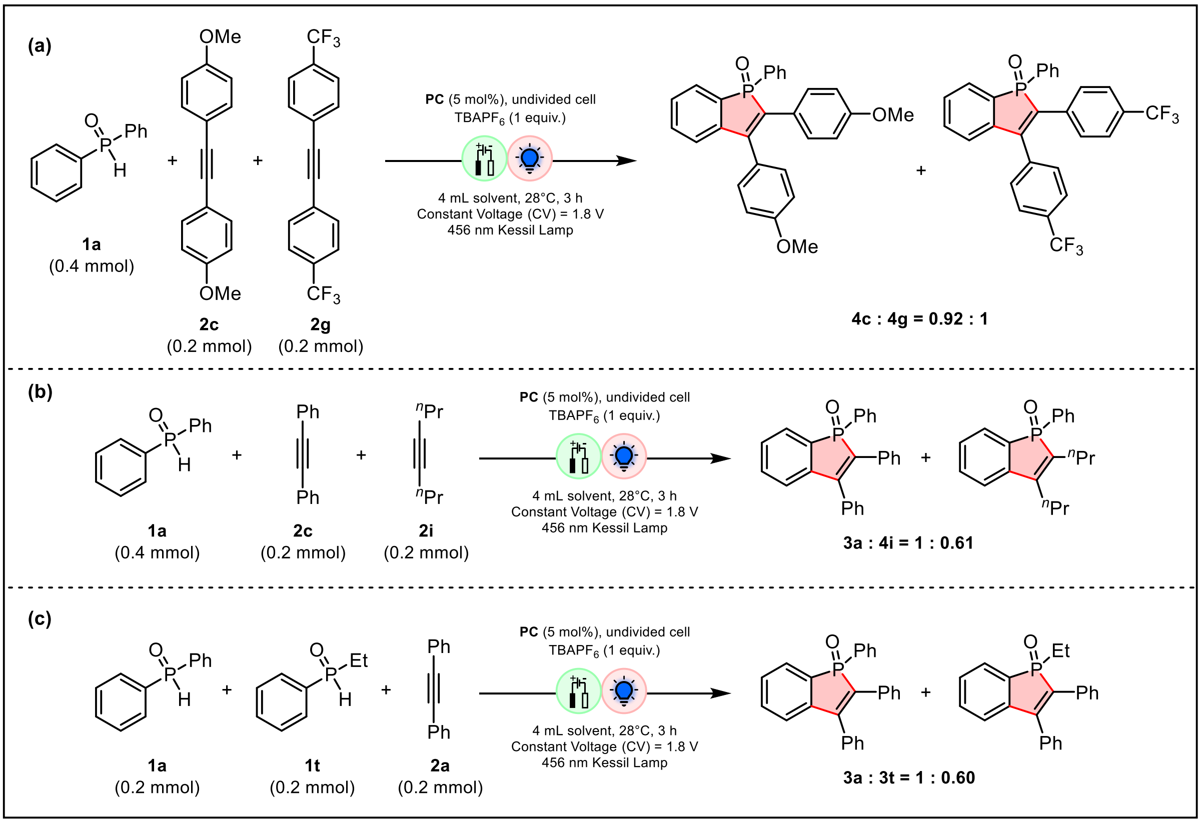

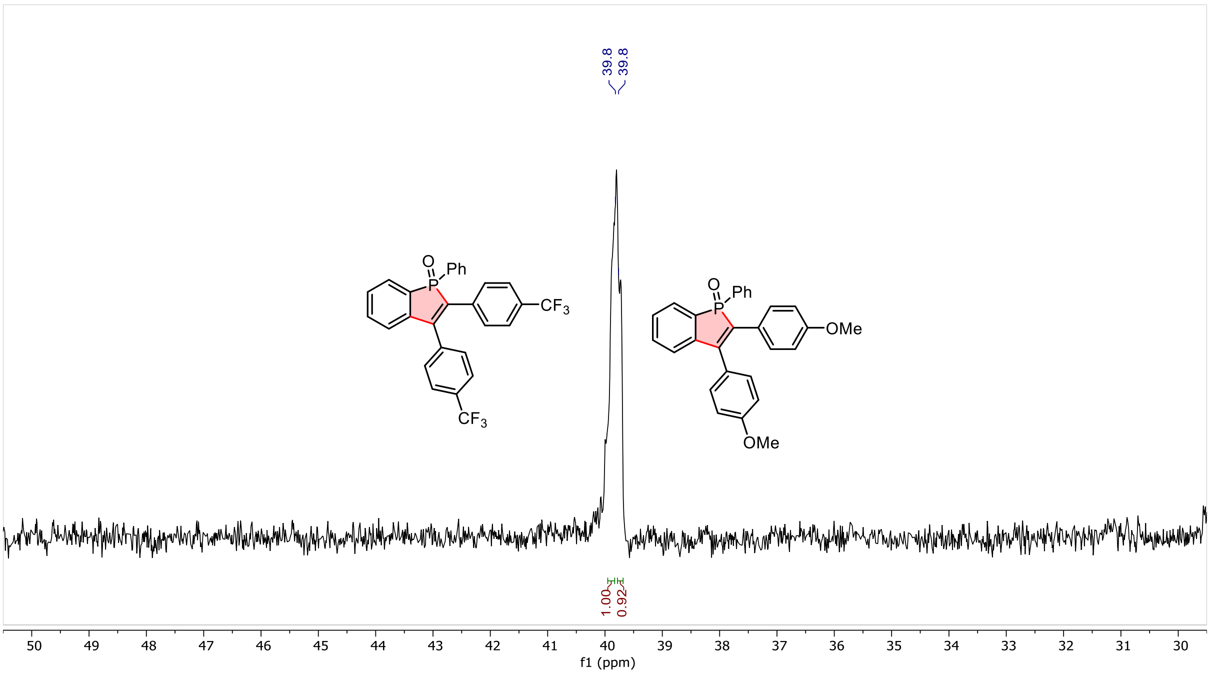

Figure S43: ^{31}P NMR for intermolecular competition experiment (a).

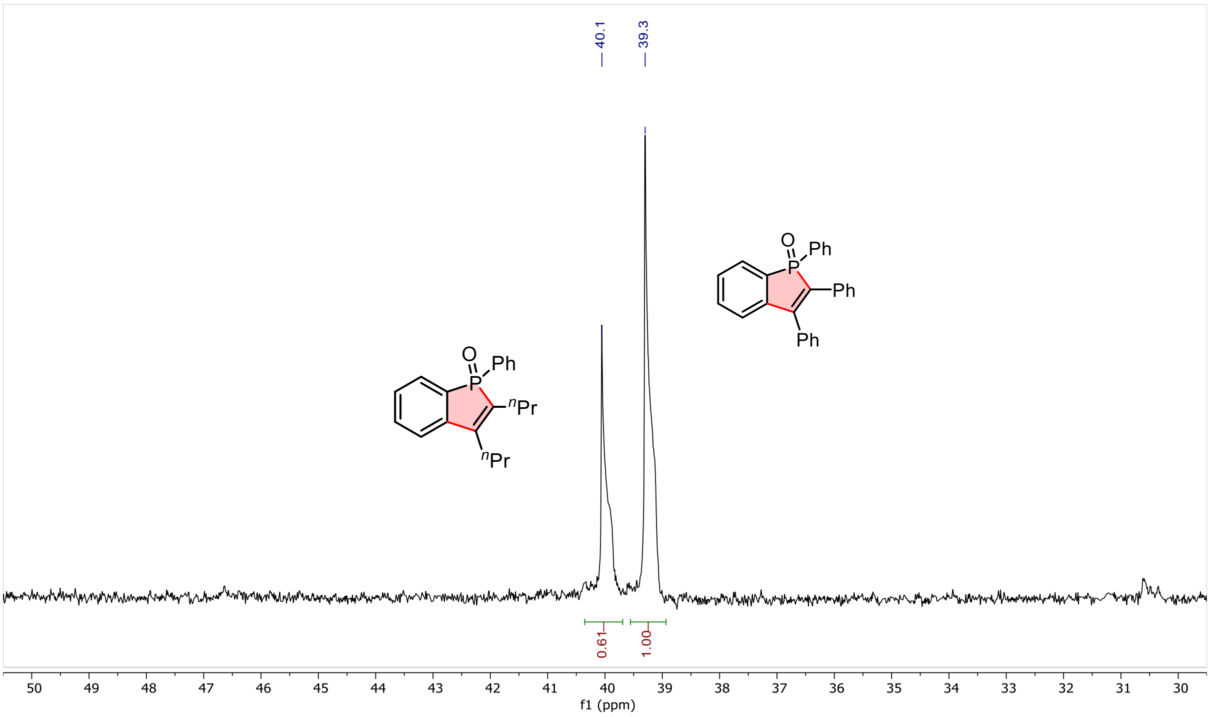

Figure S44: ^{31}P NMR for intermolecular competition experiment (b).

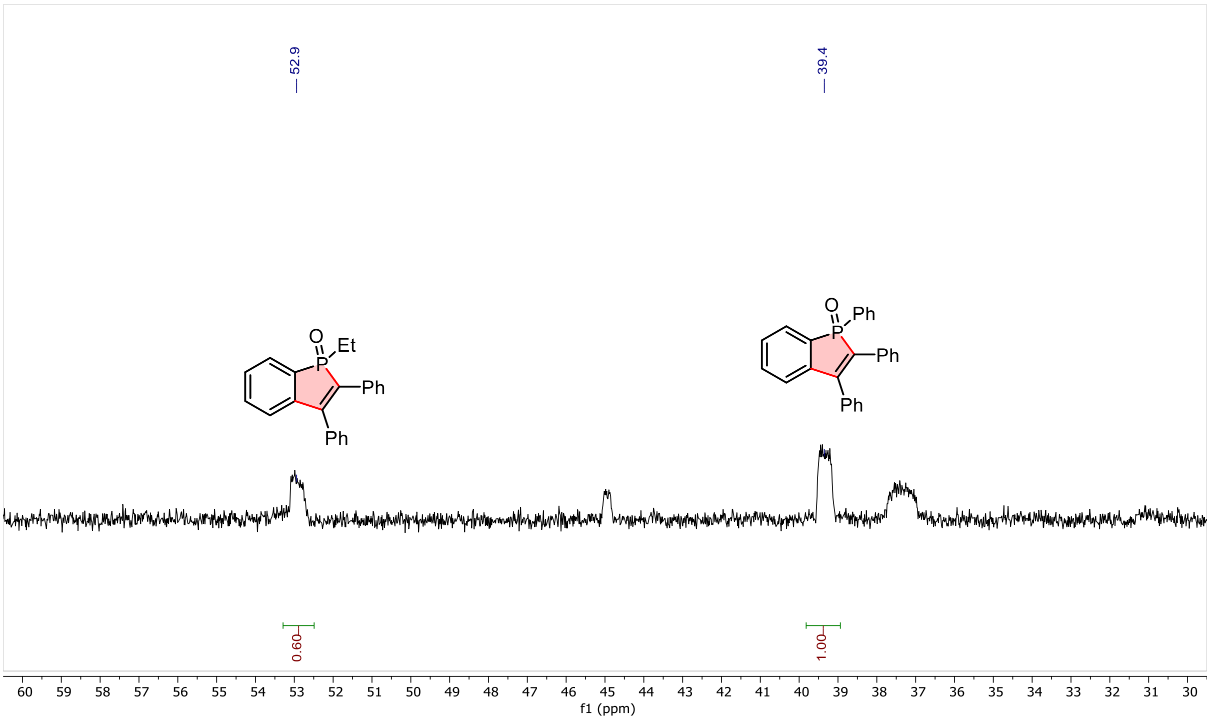

Figure S45: ^{31}P NMR for intermolecular competition experiment (c).

21. Characterization Data of the Products:

1,2,3-triphenylphosphindole 1-oxide (3a)[7]

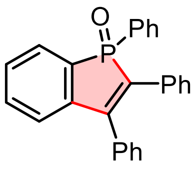

3a

According to the General Procedure for the Photoelectrochemical Annulation, diphenylphosphine oxide (80.87 mg, 0.4 mmol, 2.0 equiv.), diphenylacetylene (35.64 mg, 0.2 mmol, 1.0 equiv.), **PC_1** (8 mg, 5 mol%, 0.01 equiv.), $TBAPF_6$ (77.49 mg, 0.2 mmol, 1.0 equiv.) were dissolved in DMF for the reaction. After completion of the reaction followed by work up and silica gel column chromatography with EtOAc : Hexane (1:1) as eluent, afforded benzophosphole oxide **3a** (65.8 mg, 87%) as a white solid.

^{1}H NMR (400 MHz, $CDCl_3$) δ 7.69 (ddd, J = 12.6, 8.3, 1.4 Hz, 2H), 7.62 (ddd, J = 9.4, 7.3, 1.5 Hz, 1H), 7.40 – 7.20 (m, 10H), 7.18 – 7.09 (m, 3H), 7.02 – 6.95 (m, 3H) ppm.

^{13}C{^{1}H} NMR (101 MHz, $CDCl_3$) δ 149.96 (d, J = 21.4 Hz), 143.61 (d, J = 27.0 Hz), 132.82 (d, J = 2.0 Hz), 132.52 (d, J = 9.9 Hz), 132.09 (d, J = 2.9 Hz), 130.83 (d, J = 10.7 Hz), 129.99 (d, J = 39.8 Hz), 128.64 (d, J = 11.2 Hz), 123.95 (d, J = 10.8 Hz) ppm.

^{31}P NMR (162 MHz, $CDCl_3$) δ 39.2 ppm.

HRMS (ESI) m/z calcd. for $C_{26}H_{20}OP^+$ $(M+H)^+$: 379.1246, found : 379.1246.

5-methyl-2,3-diphenyl-1-(*p*-tolyl)phosphindole 1-oxide : 6-methyl-2,3-diphenyl-1-(*p*-tolyl)phosphindole 1-oxide (3b : 3b' = 2.5 : 1)[7] – regioisomeric mixture inseparable in column chromatography.

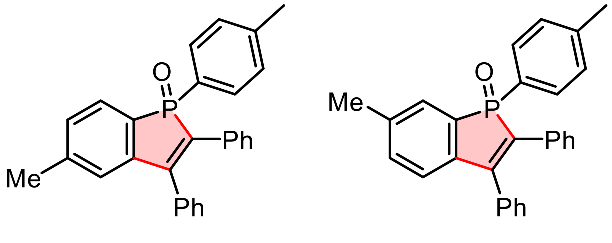

According to the General Procedure for the Photoelectrochemical Annulation, di-*p*-tolylphosphine oxide (92.09 mg, 0.4 mmol, 2.0 equiv.), diphenylacetylene (35.64 mg, 0.2 mmol, 1.0 equiv.), **PC_1** (8 mg, 5 mol%, 0.01 equiv.), $TBAPF_6$ (77.49 mg, 0.2 mmol, 1.0 equiv.) were dissolved in DMF for the reaction. After completion of the reaction followed by work up and silica gel column chromatography with EtOAc : Hexane (2:3) as eluent, afforded benzophosphole oxides **3b, 3b'** (74.7 mg, 92%) as a pale-yellow solid.

^{1}H NMR (400 MHz, $CDCl_3$) δ 7.75 – 7.49 (m, 6H), 7.49 – 7.36 (m, 6H), 7.36 – 7.29 (m, 4H), 7.24 – 7.13 (m, 11H), 7.13 – 7.02 (m, 6H), 7.01 – 6.96 (m, 1H), 2.66 – 2.21 (m, 12H) ppm.

^{13}C{^{1}H} NMR (101 MHz, $CDCl_3$) δ 149.8 (d, J = 21.1 Hz), 144.1 (d, J = 27.1 Hz), 143.5 (d, J = 2.1 Hz), 142.6 (d, J = 2.6 Hz), 135.3, 134.5 (d, J = 15.0 Hz), 133.2, 132.9 (d, J = 10.1 Hz), 131.0 (d, J =

11.0 Hz), 129.7 (d, J = 4.6 Hz), 129.6 (d, J = 2.2 Hz), 129.6, 129.1 (d, J = 5.2 Hz), 129.0 (d, J = 3.6 Hz), 128.9, 128.9 (d, J = 2.6 Hz), 128.5 (d, J = 3.6 Hz), 128.2, 127.7 (d, J = 8.4 Hz), 127.3, 126.2, 124.9 (d, J = 11.3 Hz), 123.9, 21.8 (d, J = 29.6 Hz), 21.3 ppm.

^{31}P NMR (162 MHz, CDCl$_3$) δ 39.7, 39.3 ppm.

HRMS (ESI) m/z calcd. for $C_{28}H_{24}OP^+$ $(M+H)^+$: 407.1559, found : 407.1556.

7-methyl-2,3-diphenyl-1-(*o*-tolyl)phosphindole 1-oxide : 4-methyl-2,3-diphenyl-1-(*o*-tolyl)phosphindole 1-oxide (3c : 3c' = 1 : 2.5)[7] – regioisomeric mixture inseparable in column chromatography.

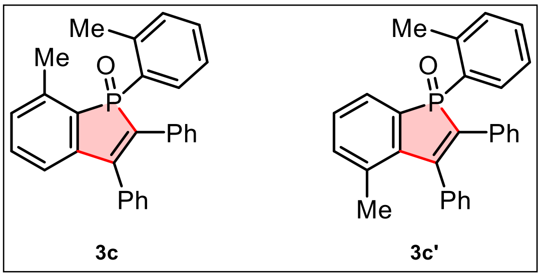

According to the General Procedure for the Photoelectrochemical Annulation, di-*o*-tolylphosphine oxide (92.09 mg, 0.4 mmol, 2.0 equiv.), diphenylacetylene (35.64 mg, 0.2 mmol, 1.0 equiv.), **PC$_1$** (8 mg, 5 mol%, 0.01 equiv.), TBAPF$_6$ (77.49 mg, 0.2 mmol, 1.0 equiv.) were dissolved in DMF for the reaction. After completion of the reaction followed by work up and silica gel column chromatography with EtOAc : Hexane (2:3) as eluent, afforded benzophosphole oxides **3c, 3c'** (63.4 mg, 78%) as an off-white solid.

^{1}H NMR (400 MHz, CDCl$_3$) δ 8.3 (ddd, J = 13.5, 7.5, 1.8 Hz, 0.4H), 8.2 (ddd, J = 13.5, 7.7, 1.5 Hz, 1H), 7.9 (q, J = 7.4 Hz, 0.4H), 7.5 (ddd, J = 10.5, 7.0, 1.5 Hz, 1.3H), 7.4 – 7.3 (m, 9H), 7.2 – 7.2 (m, 3H), 7.2 (dd, J = 7.7, 6.2 Hz, 2H), 7.2 – 7.1 (m, 6.3H), 7.1 – 7.0 (m, 5H), 2.3 (s, 1.46H), 2.3 (s, 3.12H), 2.2 (s, 2.1H), 1.7 (s, 3H) ppm.

^{13}C{^{1}H} NMR (101 MHz, CDCl$_3$) δ 152.4 (d, J = 21.4 Hz), 150.2 (d, J = 21.9 Hz), 144.6 (d, J = 27.3 Hz), 143.5, 141.2 (d, J = 10.8 Hz), 141.0 (d, J = 10.6 Hz), 140.7, 140.6, 140.5, 137.6 (d, J = 15.1 Hz), 137.3 (d, J = 1.7 Hz), 135.7 (d, J = 10.5 Hz), 134.9 (d, J = 2.5 Hz), 134.6 (d, J = 15.0 Hz), 134.3 (d, J = 9.5 Hz), 133.8, 133.7 (d, J = 6.8 Hz), 133.2 (d, J = 5.2 Hz), 133.0, 133.0 (d, J = 3.3 Hz), 132.9, 132.3 (d, J = 2.8 Hz), 132.2, 132.2 (d, J = 3.4 Hz), 131.8 (d, J = 5.9 Hz), 131.5 (d, J = 11.3 Hz), 130.7 (d, J = 9.3 Hz), 130.0, 129.1 (d, J = 10.9 Hz), 128.6, 128.2 (d, J = 4.0 Hz), 128.1, 128.0, 128.0, 127.7, 127.5, 127.1 (d, J = 6.7 Hz), 127.0 (d, J = 3.5 Hz), 126.3 (d, J = 11.6 Hz), 126.1 (d, J = 11.8 Hz), 125.3 (d, J = 6.0 Hz), 122.0 (d, J = 10.8 Hz), 21.1 (d, J = 65.5 Hz), 20.3 (d, J = 4.3 Hz), 19.9 (d, J = 4.1 Hz), 19.3 (d, J = 4.4 Hz) ppm.

^{31}P NMR (162 MHz, CDCl$_3$) δ 37.5, 37.3 ppm.

HRMS (ESI) m/z calcd. for $C_{28}H_{24}OP^+$ $(M+H)^+$: 407.1559, found : 407.1559.

1-(3,5-dimethylphenyl)-4,6-dimethyl-2,3-diphenylphosphindole 1-oxide (3d)[11]

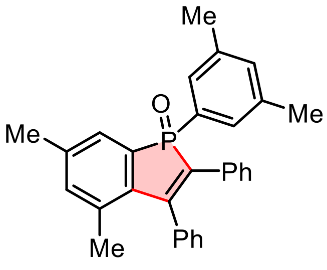

3d

According to the General Procedure for the Photoelectrochemical Annulation, bis(3,5-dimethylphenyl)phosphine oxide (131.4 mg, 0.4 mmol, 2.0 equiv.), diphenylacetylene (35.64 mg, 0.2 mmol, 1.0 equiv.), **PC_1** (8 mg, 5 mol%, 0.01 equiv.), $TBAPF_6$ (77.49 mg, 0.2 mmol, 1.0 equiv.) were dissolved in DMF for the reaction. After completion of the reaction followed by work up and silica gel column chromatography with EtOAc : Hexane (2:3) as eluent, afforded benzophosphole oxide **3d** (76.5 mg, 88%) as a white gum.

^{1}H NMR (400 MHz, $CDCl_3$) δ 7.4 – 7.3 (m, 5H), 7.3 – 7.1 (m, 4H), 7.1 – 7.0 (m, 6H), 2.3 (d, J = 4.0 Hz, 12H) ppm.

^{13}C{^{1}H} NMR (101 MHz, $CDCl_3$) δ 142.6, 139.2, 138.5 (d, J = 13.0 Hz), 137.9 (d, J = 2.5 Hz), 137.8 (d, J = 5.8 Hz), 137.7, 135.1 (d, J = 11.4 Hz), 134.0 (d, J = 3.0 Hz), 133.5, 133.0, 131.2, 130.3, 130.1 (d, J = 4.6 Hz), 130.0 (d, J = 9.5 Hz), 129.2, 129.1 (d, J = 5.5 Hz), 128.7 (d, J = 9.6 Hz), 128.5 (d, J = 10.3 Hz), 128.1 (d, J = 7.5 Hz), 127.9 (d, J = 11.4 Hz), 127.6, 127.3, 21.3 (d, J = 4.6 Hz) ppm.

^{31}P NMR (162 MHz, $CDCl_3$) δ 39.1 ppm.

HRMS (ESI) m/z calcd. for $C_{30}H_{28}OP^+$ $(M+H)^+$: 435.1872, found : 435.1869.

5-(tert-butyl)-1-(4-(tert-butyl)phenyl)-2,3-diphenylphosphindole 1-oxide : 6-(tert-butyl)-1-(4-(tert-butyl)phenyl)-2,3-diphenylphosphindole 1-oxide (3e : 3e' = 1 : 0.8) – regioisomeric mixture inseparable in column chromatography.

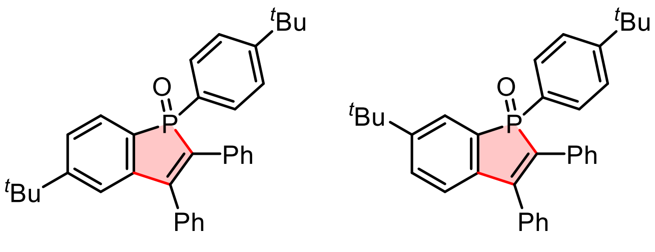

3e **3e'**

According to the General Procedure for the Photoelectrochemical Annulation, bis(4-(tert-butyl)phenyl)phosphine oxide (125.76 mg, 0.4 mmol, 2.0 equiv.), diphenylacetylene (35.64 mg, 0.2 mmol, 1.0 equiv.), **PC_1** (8 mg, 5 mol%, 0.01 equiv.), $TBAPF_6$ (77.49 mg, 0.2 mmol, 1.0 equiv.) were dissolved in DMF for the reaction. After completion of the reaction followed by work up and silica gel column chromatography with EtOAc : Hexane (2:3) as eluent, afforded benzophosphole oxides **3e, 3e'** (84.4 mg, 86%) as a pale-yellow gum.

^{1}H NMR (400 MHz, $CDCl_3$) δ 7.8 (dd, J = 10.6, 1.9 Hz, 1H), 7.7 (ddt, J = 12.2, 6.6, 1.2 Hz, 4H), 7.7 – 7.6 (m, 2H), 7.6 – 7.5 (m, 2H), 7.5 – 7.5 (m, 2H), 7.5 – 7.4 (m, 9H), 7.4 – 7.3 (m, 6H), 7.3 (ddt, J = 7.4, 6.3, 2.5 Hz, 5H), 7.2 – 7.1 (m, 6H), 1.3 (d, J = 9.1 Hz, 18H), 1.3 (d, J = 8.6 Hz, 16H) ppm.

$^{13}C\{^1H\}$ NMR (101 MHz, CDCl$_3$) δ 156.7 (d, *J* = 2.1 Hz), 155.5, 155.5 (d, *J* = 2.7 Hz), 155.2 (d, *J* = 2.8 Hz), 152.7 (d, *J* = 9.8 Hz), 150.3 (d, *J* = 21.3 Hz), 149.8 (d, *J* = 21.7 Hz), 143.7 (d, *J* = 27.2 Hz), 141.1 (d, *J* = 27.3 Hz), 134.8 (d, *J* = 26.9 Hz), 134.5 (d, *J* = 1.9 Hz), 134.3 (d, *J* = 13.6 Hz), 133.6 (d, *J* = 68.9 Hz), 133.1 (d, *J* = 3.4 Hz), 133.0 (d, *J* = 3.5 Hz), 132.6, 132.0 (d, *J* = 10.3 Hz), 131.6, 131.5, 130.9 (d, *J* = 10.9 Hz), 130.0, 129.7 (d, *J* = 2.1 Hz), 129.6, 129.2 (d, *J* = 3.9 Hz), 129.1 (d, *J* = 3.9 Hz), 129.0, 128.9 (d, *J* = 10.6 Hz), 128.6 (d, *J* = 6.3 Hz), 128.4, 128.2 (d, *J* = 6.1 Hz), 128.0, 127.6 (d, *J* = 3.5 Hz), 127.2 (d, *J* = 3.6 Hz), 126.2 (d, *J* = 4.7 Hz), 126.2 (d, *J* = 2.4 Hz), 126.1, 126.0 (d, *J* = 3.1 Hz), 125.9 (d, *J* = 2.9 Hz), 125.4 (d, *J* = 12.5 Hz), 123.7 (d, *J* = 11.5 Hz), 123.3, 121.3 (d, *J* = 11.1 Hz), 35.2 (d, *J* = 19.8 Hz), 35.0, 31.2 (d, *J* = 9.9 Hz), 31.1 (d, *J* = 2.8 Hz) ppm.

^{31}P NMR (162 MHz, CDCl$_3$) δ 40.6, 39.8 ppm.

HRMS (ESI) m/z calcd. for $C_{34}H_{36}OP^+$ $(M+H)^+$: 491.2498, found : 491.2502.

5-chloro-1-(4-chlorophenyl)-2,3-diphenylphosphindole 1-oxide : 6-chloro-1-(4-chlorophenyl)-2,3-diphenylphosphindole 1-oxide (3f : 3f' = 1 : 0.6) – regioisomeric mixture separable in column chromatography.

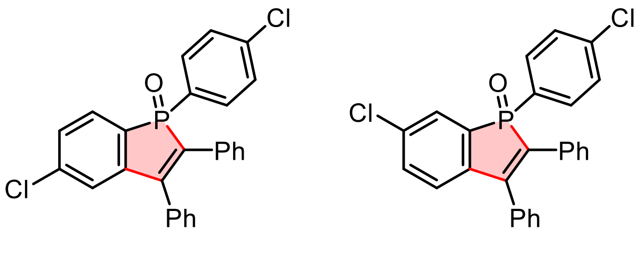

3f 3f'

According to the General Procedure for the Photoelectrochemical Annulation, bis(4-chlorophenyl)phosphine oxide (108.43 mg, 0.4 mmol, 2.0 equiv.), diphenylacetylene (35.64 mg, 0.2 mmol, 1.0 equiv.), **PC$_1$** (8 mg, 5 mol%, 0.01 equiv.), TBAPF$_6$ (77.49 mg, 0.2 mmol, 1.0 equiv.) were dissolved in DMF for the reaction. After completion of the reaction followed by work up and silica gel column chromatography with EtOAc : Hexane (1:1) as eluent, afforded benzophosphole oxides **3f** (45.8 mg)**, 3f'** (27.5 mg) as pale-yellow solid and pale-yellow gum (Overall yield 82%).

➢ For **3f**:

^1H NMR (400 MHz, CDCl$_3$) δ 7.74 – 7.64 (m, 2H), 7.64 (dd, *J* = 9.9, 2.0 Hz, 1H), 7.47 – 7.36 (m, 6H), 7.32 – 7.28 (m, 2H), 7.22 – 7.19 (m, 2H), 7.19 – 7.04 (m, 4H) ppm.

$^{13}C\{^1H\}$ NMR (101 MHz, CDCl$_3$) δ 149.5 (d, *J* = 20.9 Hz), 141.9 (d, *J* = 27.2 Hz), 140.0, 139.2 (d, *J* = 3.7 Hz), 138.2, 135.7 (d, *J* = 14.0 Hz), 134.4 (d, *J* = 14.1 Hz), 133.6 (d, *J* = 15.4 Hz), 133.4 (d, *J* = 21.8 Hz), 133.0 (d, *J* = 1.9 Hz), 132.3 (d, *J* = 11.5 Hz), 132.1 (d, *J* = 10.0 Hz), 129.5 (d, *J* = 13.1 Hz), 129.2 (d, *J* = 9.0 Hz), 129.0 (d, *J* = 10.9 Hz), 128.9, 128.5, 128.2 (d, *J* = 12.3 Hz), 127.1, 125.8, 125.3 (d, *J* = 11.9 Hz), 124.6 (d, *J* = 21.0 Hz) ppm.

^{31}P NMR (162 MHz, CDCl$_3$) δ 37.9 ppm.

HRMS (ESI) m/z calcd. for $C_{26}H_{18}Cl_2OP^+$ $(M+H)^+$: 447.0467, found : 447.0463.

- For **3f'**:

^{1}H NMR (400 MHz, CDCl$_3$) δ 7.71 – 7.57 (m, 2H), 7.57 – 7.52 (m, 1H), 7.48 – 7.27 (m, 6H), 7.24 – 7.18 (m, 2H), 7.18 – 7.11 (m, 3H), 7.11 – 6.89 (m, 3H) ppm.

^{13}C{^{1}H} NMR (101 MHz, CDCl$_3$) δ 149.1, 143.8 (d, *J* = 9.6 Hz), 139.9, 138.9 (d, *J* = 34.6 Hz), 133.7 (d, *J* = 10.3 Hz), 133.4 (d, *J* = 15.0 Hz), 132.7, 132.3 (d, *J* = 11.4 Hz), 132.1, 130.4, 130.1 (d, *J* = 10.5 Hz), 129.9 (d, *J* = 4.4 Hz), 129.4 (d, *J* = 13.0 Hz), 129.3, 129.2 (d, *J* = 9.0 Hz), 129.0 (d, *J* = 4.3 Hz), 128.9 (d, *J* = 5.6 Hz), 128.8 (d, *J* = 12.5 Hz), 128.5 (d, *J* = 10.5 Hz), 128.3 (d, *J* = 5.9 Hz), 128.1, 127.4, 124.6 (d, *J* = 11.7 Hz) ppm.

^{31}P NMR (162 MHz, CDCl$_3$) δ 37.7 ppm.

HRMS (ESI) m/z calcd. for $C_{26}H_{18}Cl_2OP^+$ $(M+H)^+$: 447.0467, found : 447.0468.

5-fluoro-1-(4-fluorophenyl)-2,3-diphenylphosphindole 1-oxide : 6-fluoro-1-(4-fluorophenyl)-2,3-diphenylphosphindole 1-oxide (3g : 3g' = 1 : 0.54)[8] – regioisomeric mixture inseparable in column chromatography.

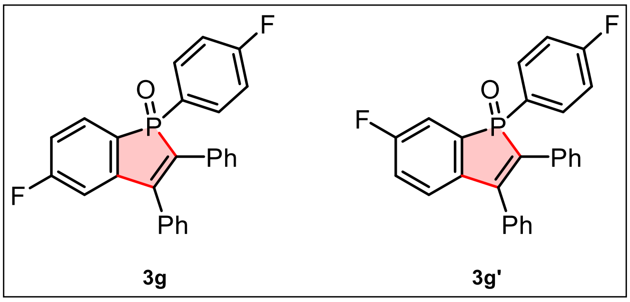

According to the General Procedure for the Photoelectrochemical Annulation, bis(4-fluorophenyl)phosphine oxide (95.27 mg, 0.4 mmol, 2.0 equiv.), diphenylacetylene (35.64 mg, 0.2 mmol, 1.0 equiv.), **PC$_1$** (8 mg, 5 mol%, 0.01 equiv.), TBAPF$_6$ (77.49 mg, 0.2 mmol, 1.0 equiv.) were dissolved in DMF for the reaction. After completion of the reaction followed by work up and silica gel column chromatography with EtOAc : Hexane (2:3) as eluent, afforded benzophosphole oxides **3g, 3g'** (61.4 mg, 74%) as a yellow gum.

^{1}H NMR (400 MHz, CDCl$_3$) δ 7.82 – 7.72 (m, 3H), 7.69 (ddd, *J* = 9.3, 8.0, 5.4 Hz, 1H), 7.47 – 7.38 (m, 5H), 7.34 – 7.28 (m, 3H), 7.25 – 7.17 (m, 4H), 7.09 (ddtd, *J* = 10.6, 6.3, 4.3, 2.0 Hz, 9H), 6.93 (dt, *J* = 9.4, 2.3 Hz, 1H) ppm.

^{13}C{^{1}H} NMR (101 MHz, CDCl$_3$) δ 167.6 (d, *J* = 2.2 Hz), 166.8, 166.7 (d, *J* = 3.5 Hz), 165.0 (d, *J* = 2.3 Hz), 164.7 (d, *J* = 15.2 Hz), 164.2 (d, *J* = 3.4 Hz), 162.1 (d, *J* = 15.3 Hz), 149.5 (d, *J* = 21.8 Hz), 148.7 (d, *J* = 2.6 Hz), 148.5 (d, *J* = 2.7 Hz), 147.1 (d, *J* = 8.8 Hz), 146.8 (d, *J* = 8.7 Hz), 139.4 (d, *J* =

27.2 Hz), 136.5, 135.0 (d, J = 7.0 Hz), 134.1 (d, J = 16.4 Hz), 133.8 (d, J = 15.3 Hz), 133.5 (d, J = 8.7 Hz), 133.4 (d, J = 3.0 Hz), 133.4, 133.2, 132.3 (d, J = 9.9 Hz), 132.2 (d, J = 9.8 Hz), 131.1 (d, J = 9.4 Hz), 131.0 (d, J = 9.4 Hz), 129.4, 129.2 (d, J = 8.0 Hz), 129.0 (d, J = 8.6 Hz), 128.9 (d, J = 5.8 Hz), 128.9 (d, J = 3.0 Hz), 128.5, 128.4 (d, J = 9.5 Hz), 128.1, 127.6 (d, J = 3.4 Hz), 127.5, 126.5 (d, J = 3.2 Hz), 126.0, 125.9 (d, J = 5.1 Hz), 125.7 (d, J = 4.0 Hz), 125.4, 124.7 (d, J = 3.4 Hz), 124.4, 119.6 (d, J = 21.6 Hz), 116.9, 116.8 (d, J = 8.8 Hz), 116.6 (d, J = 6.0 Hz), 116.5 (d, J = 7.9 Hz), 116.3 (d, J = 7.8 Hz), 116.0 (d, J = 12.0 Hz), 115.8 (d, J = 11.8 Hz), 112.3 (d, J = 12.2 Hz), 112.1 (d, J = 12.3 Hz) ppm.

^{31}P NMR (162 MHz, CDCl$_3$) δ 37.8 (d, J = 5.6 Hz), 37.5 (d, J = 5.6 Hz) ppm.

^{19}F NMR (376 MHz, CDCl$_3$) δ -105.0 – -106.1 (m), -106.2 – -111.2 (m) ppm.

HRMS (ESI) m/z calcd. for $C_{26}H_{18}F_2OP^+$ $(M+H)^+$: 415.1058, found : 415.1058.

5-methoxy-1-(4-methoxyphenyl)-2,3-diphenylphosphindole 1-oxide : 6-methoxy-1-(4-methoxyphenyl)-2,3-diphenylphosphindole 1-oxide (3h : 3h' = 1 : 0.6)[7] – regioisomeric mixture inseparable in column chromatography.

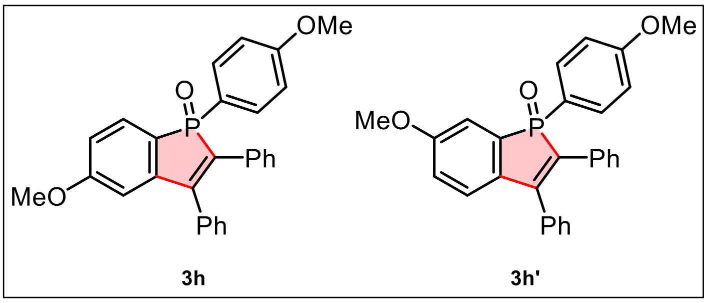

According to the General Procedure for the Photoelectrochemical Annulation, bis(4-methoxyphenyl)phosphine oxide (104.89 mg, 0.4 mmol, 2.0 equiv.), diphenylacetylene (35.64 mg, 0.2 mmol, 1.0 equiv.), **PC$_1$** (8 mg, 5 mol%, 0.01 equiv.), TBAPF$_6$ (77.49 mg, 0.2 mmol, 1.0 equiv.) were dissolved in DMF for the reaction. After completion of the reaction followed by work up and silica gel column chromatography with EtOAc : Hexane (3:2) as eluent, afforded benzophosphole oxides **3h, 3h'** (78.1 mg, 89%) as a yellow solid.

^{1}H NMR (400 MHz, CDCl$_3$) δ 7.73 – 7.58 (m, 4H), 7.38 (ddt, J = 6.5, 5.0, 2.4 Hz, 4H), 7.29 (ddd, J = 6.8, 4.4, 2.1 Hz, 3H), 7.20 (dddd, J = 9.8, 6.2, 3.3, 1.9 Hz, 4H), 7.12 – 7.04 (m, 6H), 6.93 – 6.80 (m, 5H), 6.72 (t, J = 2.3 Hz, 1H), 3.78 – 3.74 (m, 9H) ppm.

^{13}C{^{1}H} NMR (101 MHz, CDCl$_3$) δ 163.7 (d, J = 2.1 Hz), 162.8 (d, J = 3.0 Hz), 162.7 (d, J = 3.0 Hz), 160.7 (d, J = 13.4 Hz), 149.8 (d, J = 21.2 Hz), 148.9 (d, J = 20.9 Hz), 146.2 (d, J = 28.7 Hz), 136.4 (d, J = 46.4 Hz), 135.8 (d, J = 22.2 Hz), 134.8 (d, J = 32.1 Hz), 134.4 (d, J = 17.0 Hz), 134.0 (d, J = 25.8 Hz), 133.1 (d, J = 9.4 Hz), 133.0 (d, J = 3.4 Hz), 132.9 (d, J = 3.1 Hz), 132.8 (d, J = 3.1 Hz), 132.7, 131.7 (d, J = 12.2 Hz), 130.5 (d, J = 11.1 Hz), 130.0, 129.1 (d, J = 2.5 Hz), 129.0 (d, J = 3.0 Hz), 128.9 (d, J = 2.3 Hz), 128.6, 128.2, 127.7 (d, J = 16.8 Hz), 127.3 (d, J = 42.9 Hz), 125.2 (d, J = 12.8 Hz),

123.2 (d, J = 112.5 Hz), 121.3 (d, J = 45.3 Hz), 120.3 (d, J = 43.7 Hz), 117.8, 114.7 (d, J = 3.5 Hz), 114.6 (d, J = 2.7 Hz), 114.4, 113.7 (d, J = 11.3 Hz), 113.5 (d, J = 13.3 Hz), 112.8 (d, J = 11.8 Hz), 111.5 (d, J = 11.9 Hz), 55.6 (d, J = 17.7 Hz), 55.3 (d, J = 1.7 Hz), 55.2 ppm.

^{31}P NMR (162 MHz, CDCl$_3$) δ 39.7, 38.8 ppm.

HRMS (ESI) m/z calcd. for $C_{28}H_{24}O_3P^+$ $(M+H)^+$: 439.1458, found : 439.1459.

7-methoxy-1-(2-methoxyphenyl)-2,3-diphenylphosphindole 1-oxide : 4-methoxy-1-(2-methoxyphenyl)-2,3-diphenylphosphindole 1-oxide (3i : 3i' = 1 : 6) – regioisomeric mixture inseparable in column chromatography.

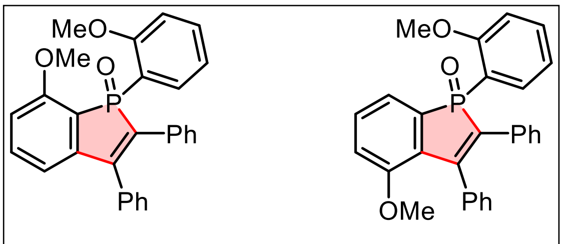

3i 3i'

According to the General Procedure for the Photoelectrochemical Annulation, bis(2-methoxyphenyl)phosphine oxide (104.89 mg, 0.4 mmol, 2.0 equiv.), diphenylacetylene (35.64 mg, 0.2 mmol, 1.0 equiv.), **PC$_1$** (8 mg, 5 mol%, 0.01 equiv.), TBAPF$_6$ (77.49 mg, 0.2 mmol, 1.0 equiv.) were dissolved in DMF for the reaction. After completion of the reaction followed by work up and silica gel column chromatography with EtOAc : Hexane (3:2) as eluent, afforded benzophosphole oxides **3i, 3i'** (64 mg, 73%) as a yellow solid.

^{1}H NMR (400 MHz, CDCl$_3$) δ 8.26 (ddd, J = 13.6, 7.6, 1.8 Hz, 0.17H), 8.10 (ddd, J = 13.3, 7.6, 1.8 Hz, 1.01H), 7.44 – 7.38 (m, 1.32H), 7.36 – 7.30 (m, 1.01H), 7.29 – 7.21 (m, 7.51H), 7.09 (ddt, J = 6.1, 4.1, 1.8 Hz, 2.72H), 7.06 – 6.91 (m, 6.02H), 6.80 – 6.69 (m, 1.66H), 3.68 (s, 0.5H), 3.56 (s, 3H), 3.45 (s, 0.52H), 3.39 (s, 2.93H) ppm.

^{13}C{^{1}H} NMR (101 MHz, CDCl$_3$) δ 162.6, 160.8 (d, J = 4.6 Hz), 160.2 (d, J = 4.9 Hz), 156.0 (d, J = 15.1 Hz), 150.1 (d, J = 22.3 Hz), 148.8 (d, J = 22.6 Hz), 146.3 (d, J = 25.5 Hz), 137.9 (d, J = 15.7 Hz), 136.3 (d, J = 6.1 Hz), 135.4 (d, J = 6.2 Hz), 135.2, 135.0 (d, J = 9.6 Hz), 134.6, 134.2 (d, J = 2.2 Hz), 133.8 (d, J = 11.5 Hz), 133.7 (d, J = 3.2 Hz), 133.6, 133.4 (d, J = 10.4 Hz), 132.7, 130.8 (d, J = 14.2 Hz), 130.5 (d, J = 1.7 Hz), 129.1 (d, J = 5.6 Hz), 129.0, 128.6 (d, J = 20.6 Hz), 128.1 (d, J = 27.1 Hz), 127.9, 127.4 (d, J = 26.5 Hz), 127.2 (d, J = 15.8 Hz), 121.1, 121.0 (d, J = 3.0 Hz), 118.2 (d, J = 8.1 Hz), 117.8 (d, J = 2.0 Hz), 117.2, 116.7 (d, J = 11.1 Hz), 112.0 (d, J = 6.0 Hz), 111.1 (d, J = 6.7 Hz), 110.8 (d, J = 6.9 Hz), 55.7 (d, J = 35.4 Hz), 55.7 (d, J = 51.8 Hz) ppm.

^{31}P NMR (162 MHz, CDCl$_3$) δ 38.6, 37.0 ppm.

HRMS (ESI) m/z calcd. for $C_{28}H_{24}O_3P^+$ $(M+H)^+$: 439.1458, found : 439.1462.

2,3-diphenyl-5-(trifluoromethyl)-1-(4-(trifluoromethyl)phenyl)phosphindole 1-oxide : 2,3-diphenyl-6-(trifluoromethyl)-1-(4-(trifluoromethyl)phenyl)phosphindole 1-oxide (3j : 3j' = 1 : 0.4) – regioisomeric mixture inseparable in column chromatography.

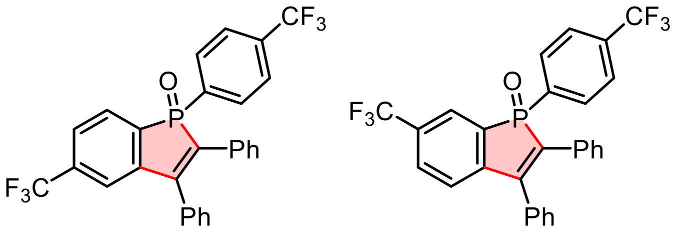

3j 3j'

According to the General Procedure for the Photoelectrochemical Annulation, bis(4-(trifluoromethyl)phenyl)phosphine oxide (135.27 mg, 0.4 mmol, 2.0 equiv.), diphenylacetylene (35.64 mg, 0.2 mmol, 1.0 equiv.), **PC_1** (8 mg, 5 mol%, 0.01 equiv.), $TBAPF_6$ (77.49 mg, 0.2 mmol, 1.0 equiv.) were dissolved in DMF for the reaction. After completion of the reaction followed by work up and silica gel column chromatography with EtOAc : Hexane (1:1) as eluent, afforded benzophosphole oxides **3j, 3j'** (74.1 mg, 72%) as a pale-yellow oil.

^{1}H NMR (400 MHz, $CDCl_3$) δ 7.88 – 7.78 (m, 2.82H), 7.73 (dd, *J* = 9.5, 7.6 Hz, 0.34H), 7.65 (d, *J* = 8.1 Hz, 0.8H), 7.59 (dt, *J* = 8.4, 2.8 Hz, 2.38H), 7.46 – 7.35 (m, 6H), 7.30 – 7.21 (m, 6.68H), 7.20 – 7.14 (m, 2.36H), 7.10 – 7.01 (m, 3.13H) ppm.

^{13}C{^{1}H} NMR (101 MHz, $CDCl_3$) δ 149.6 – 149.3 (m), 147.0 (d, *J* = 28.0 Hz), 144.6 (d, *J* = 28.5 Hz), 136.1 (d, *J* = 96.0 Hz), 134.6 – 133.9 (m), 133.3 (d, *J* = 15.1 Hz), 133.0 (d, *J* = 17.7 Hz), 131.8 (d, *J* = 15.8 Hz), 131.7, 131.6, 131.6, 131.5 (d, *J* = 10.9 Hz), 130.6, 129.5 (d, *J* = 10.2 Hz), 129.4 (d, *J* = 2.1 Hz), 129.0 (d, *J* = 6.0 Hz), 128.8 (d, *J* = 3.2 Hz), 128.7 (d, *J* = 9.1 Hz), 128.6, 128.3 (d, *J* = 9.0 Hz), 126.1 (d, *J* = 4.0 Hz), 126.0 (d, *J* = 4.2 Hz), 125.9 (d, *J* = 4.0 Hz), 124.8 (d, *J* = 9.9 Hz), 124.4 (d, *J* = 10.9 Hz), 123.3, 122.1 (d, *J* = 8.3 Hz), 89.4 ppm.

^{31}P NMR (162 MHz, $CDCl_3$) δ 37.0, 36.9 ppm.

^{19}F NMR (376 MHz, $CDCl_3$) δ -63.2, -63.7, -63.79, -63.78 ppm.

HRMS (ESI) m/z calcd. for $C_{28}H_{18}F_6OP^+$ $(M+H)^+$: 515.0994, found : 515.0999.

1-(naphthalen-1-yl)-2,3-diphenylbenzo[*g*]phosphindole 1-oxide (3k)

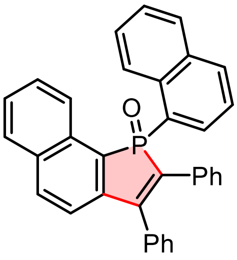

3k

According to the General Procedure for the Photoelectrochemical Annulation, di(naphthalen-1-yl)phosphine oxide (120.92 mg, 0.4 mmol, 2.0 equiv.), diphenylacetylene (35.64 mg, 0.2 mmol, 1.0 equiv.), **PC_1** (8 mg, 5 mol%, 0.01 equiv.), $TBAPF_6$ (77.49 mg, 0.2 mmol, 1.0 equiv.) were dissolved in DMF for the reaction. After completion of the reaction followed by work up and silica

gel column chromatography with EtOAc : Hexane (1:2) as eluent, afforded benzophosphole oxide **3k** (65.1 mg, 68%) as a yellow gum.

^{1}H NMR (400 MHz, CDCl$_3$) δ 8.67 (d, *J* = 8.3 Hz, 1H), 8.35 (ddd, *J* = 15.5, 7.2, 1.3 Hz, 1H), 7.98 (d, *J* = 7.7 Hz, 1H), 7.89 – 7.76 (m, 4H), 7.43 – 7.38 (m, 9H), 7.25 – 7.11 (m, 4H), 7.02 – 6.98 (m, 3H) ppm.

^{13}C{^{1}H} NMR (101 MHz, CDCl$_3$) δ 151.7 (d, *J* = 22.5 Hz), 139.9 (d, *J* = 26.3 Hz), 137.7 (d, *J* = 2.1 Hz), 137.6, 137.3 (d, *J* = 1.9 Hz), 136.8, 133.7 (d, *J* = 2.9 Hz), 133.6 (d, *J* = 2.1 Hz), 133.5 (d, *J* = 1.8 Hz), 133.4, 133.1, 132.9 (d, *J* = 9.2 Hz), 132.0, 131.4, 131.0 (d, *J* = 3.6 Hz), 130.8, 130.1 (d, *J* = 31.1 Hz), 129.6 (d, *J* = 12.3 Hz), 129.3 (d, *J* = 3.4 Hz), 129.1 (d, *J* = 5.2 Hz), 129.0, 128.7 (d, *J* = 20.1 Hz), 128.3 (d, *J* = 26.4 Hz), 128.1, 127.6 (d, *J* = 15.7 Hz), 127.3 (d, *J* = 10.7 Hz), 127.2 (d, *J* = 5.9 Hz), 126.7 (d, *J* = 13.5 Hz), 126.5 (d, *J* = 22.5 Hz), 126.2, 125.6 (d, *J* = 20.1 Hz), 125.3 (d, *J* = 32.2 Hz), 124.9 (d, *J* = 23.9 Hz), 123.9 (d, *J* = 10.3 Hz) ppm.

^{31}P NMR (162 MHz, CDCl$_3$) δ 39.4 ppm.

HRMS (ESI) m/z calcd. for $C_{34}H_{24}OP^{+}$ $(M+H)^{+}$: 479.1559, found : 479.1563.

1-(naphthalen-2-yl)-2,3-diphenylbenzo[*f*]phosphindole 1-oxide : 3-(naphthalen-2-yl)-1,2-diphenylbenzo[*e*]phosphindole 3-oxide (3l : 3l' = 1 : 0.3)[10] – regioisomeric mixture inseparable in column chromatography.

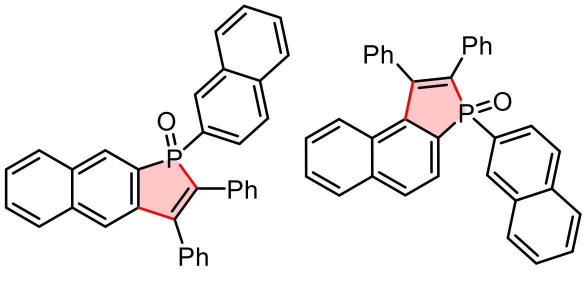

3l 3l'

According to the General Procedure for the Photoelectrochemical Annulation, di(naphthalen-2-yl)phosphine oxide (120.92 mg, 0.4 mmol, 2.0 equiv.), diphenylacetylene (35.64 mg, 0.2 mmol, 1.0 equiv.), **PC$_1$** (8 mg, 5 mol%, 0.01 equiv.), $TBAPF_6$ (77.49 mg, 0.2 mmol, 1.0 equiv.) were dissolved in DMF for the reaction. After completion of the reaction followed by work up and silica gel column chromatography with EtOAc : Hexane (1:2) as eluent, afforded benzophosphole oxides **3l, 3l'** (71.8 mg, 75%) as a yellow oil.

^{1}H NMR (400 MHz, CDCl$_3$) δ 8.77 (dd, *J* = 14.0, 1.4 Hz, 0.3H), 8.68 (dd, *J* = 14.0, 1.4 Hz, 1H), 8.23 – 8.11 (m, 0.48H), 8.02 – 7.71 (m, 8.38H), 7.64 – 7.36 (m, 13.29H), 7.32 – 7.22 (m, 2.81H), 7.19 – 7.13 (m, 2.67H), 7.06 (tdd, *J* = 6.9, 3.4, 2.0 Hz, 3.93H) ppm.

^{13}C{^{1}H} NMR (101 MHz, CDCl$_3$) δ 152.0 (d, *J* = 22.6 Hz), 140.1 (d, *J* = 26.4 Hz), 137.5 (d, *J* = 15.6 Hz), 137.4 (d, *J* = 1.7 Hz), 136.8, 135.9, 135.0 (d, *J* = 2.6 Hz), 134.4 (d, *J* = 8.6 Hz), 134.1 (d, *J* = 8.7

Hz), 133.8, 133.6, 133.0 (d, J = 13.2 Hz), 132.6 (d, J = 9.4 Hz), 131.3, 130.9 (d, J = 10.8 Hz), 130.3, 129.3, 129.2 (d, J = 3.3 Hz), 129.1 (d, J = 5.0 Hz), 128.9 (d, J = 5.6 Hz), 128.8 (d, J = 2.2 Hz), 128.7 (d, J = 5.9 Hz), 128.5, 128.3 (d, J = 1.8 Hz), 128.1 (d, J = 7.7 Hz), 127.8 (d, J = 11.2 Hz), 127.3 (d, J = 15.4 Hz), 126.9, 126.8 (d, J = 3.1 Hz), 126.6 (d, J = 4.4 Hz), 125.6 (d, J = 7.4 Hz), 125.0 (d, J = 12.7 Hz), 124.8, 123.8 (d, J = 10.3 Hz), 121.4 (d, J = 12.6 Hz) ppm.

^{31}P NMR (162 MHz, CDCl$_3$) δ 40.3, 38.7 ppm.

HRMS (ESI) m/z calcd. for $C_{34}H_{24}OP^+$ $(M+H)^+$: 479.1559, found : 479.1560.

1-ethoxy-2,3-diphenylphosphindole 1-oxide (3m)[7]

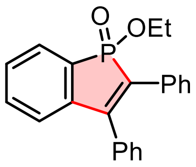

3m

According to the General Procedure for the Photoelectrochemical Annulation, ethyl phenylphosphinate (60.3 µL, 0.4 mmol, 2.0 equiv.), diphenylacetylene (35.64 mg, 0.2 mmol, 1.0 equiv.), **PC$_1$** (8 mg, 5 mol%, 0.01 equiv.), TBAPF$_6$ (77.49 mg, 0.2 mmol, 1.0 equiv.) were dissolved in DMF for the reaction. After completion of the reaction followed by work up and silica gel column chromatography with EtOAc : Hexane (2:3) as eluent, afforded benzophosphole oxide **3m** (54.7 mg, 79%) as a colourless oil.

^{1}H NMR (400 MHz, CDCl$_3$) δ 7.84 – 7.72 (m, 1H), 7.48 – 7.33 (m, 6H), 7.25 (dt, J = 5.3, 2.6 Hz, 2H), 7.19 (dt, J = 6.2, 2.7 Hz, 3H), 7.14 – 7.08 (m, 1H), 7.07 – 6.73 (m, 1H), 4.17 – 4.00 (m, 2H), 1.23 (t, J = 7.1 Hz, 3H) ppm.

^{13}C{^{1}H} NMR (101 MHz, CDCl$_3$) δ 148.7 (d, J = 27.4 Hz), 142.0 (d, J = 34.5 Hz), 133.9 (d, J = 18.1 Hz), 133.1 (d, J = 2.2 Hz), 132.5 (d, J = 8.8 Hz), 132.1 (d, J = 9.8 Hz), 131.8 (d, J = 9.9 Hz), 131.6, 130.5 (d, J = 23.3 Hz), 129.7 (d, J = 13.0 Hz), 129.2 (d, J = 24.4 Hz), 129.0 (d, J = 2.1 Hz), 128.9, 128.8, 128.7, 128.6, 128.4, 128.2 (d, J = 3.3 Hz), 127.9 (d, J = 11.8 Hz), 127.7 (d, J = 8.8 Hz), 126.7 (d, J = 31.2 Hz), 124.0 (d, J = 13.4 Hz), 62.2 (d, J = 6.4 Hz), 16.5 (d, J = 6.1 Hz) ppm.

^{31}P NMR (162 MHz, CDCl$_3$) δ 46.6 ppm.

HRMS (ESI) m/z calcd. for $C_{22}H_{20}O_2P^+$ $(M+H)^+$: 347.1195, found : 347.1197.

1-ethoxy-3-phenyl-2-(trimethylsilyl)phosphindole 1-oxide (3n)

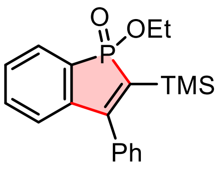

3n

According to the General Procedure for the Photoelectrochemical Annulation, ethyl phenylphosphinate (60.3 μL, 0.4 mmol, 2.0 equiv.), trimethyl(phenylethynyl)silane (39.34 μL, 0.2 mmol, 1.0 equiv.), **PC_1** (8 mg, 5 mol%, 0.01 equiv.), $TBAPF_6$ (77.49 mg, 0.2 mmol, 1.0 equiv.) were dissolved in DMF for the reaction. After completion of the reaction followed by work up and silica gel column chromatography with EtOAc : Hexane (1:3) as eluent, afforded benzophosphole oxide **3n** (55.5 mg, 81%) as a colourless oil.

^{1}H NMR (400 MHz, $CDCl_3$) δ 7.95 (ddt, J = 14.4, 6.9, 1.4 Hz, 2H), 7.59 – 7.48 (m, 4H), 7.43 (tt, J = 6.6, 1.3 Hz, 1H), 7.35 (dd, J = 8.2, 6.7 Hz, 2H), 4.29 (dqd, J = 8.2, 7.1, 1.2 Hz, 2H), 1.43 (t, J = 7.1 Hz, 3H), 0.05 (s, 9H) ppm.

^{13}C{^{1}H} NMR (101 MHz, $CDCl_3$) δ 143.2, 136.8 (d, J = 24.5 Hz), 133.0 (d, J = 3.0 Hz), 132.8 (d, J = 2.2 Hz), 132.7, 132.2, 131.3 (d, J = 11.2 Hz), 130.8, 130.5, 129.4 (d, J = 10.1 Hz), 128.8, 128.7 (d, J = 4.5 Hz), 128.2, 127.3 (d, J = 8.6 Hz), 123.9 (d, J = 14.9 Hz), 120.0 (d, J = 4.4 Hz), 101.8 (d, J = 39.6 Hz), 62.5 (d, J = 6.4 Hz), 16.5 (d, J = 6.9 Hz), 0.0 (d, J = 2.1 Hz) ppm.

^{31}P NMR (162 MHz, $CDCl_3$) δ 55.5, 9.4 ppm.

HRMS (ESI) m/z calcd. for $C_{19}H_{24}O_2PSi^+$ $(M+H)^+$: 343.1278, found : 343.1282.

1-ethoxy-2-methyl-3-phenylphosphindole 1-oxide (3o)

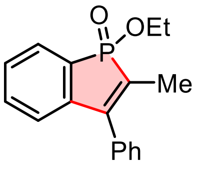

3o

According to the General Procedure for the Photoelectrochemical Annulation, ethyl phenylphosphinate (60.3 μL, 0.4 mmol, 2.0 equiv.), prop-1-yn-1-ylbenzene (25.03 μL, 0.2 mmol, 1.0 equiv.), **PC_1** (8 mg, 5 mol%, 0.01 equiv.), $TBAPF_6$ (77.49 mg, 0.2 mmol, 1.0 equiv.) were dissolved in DMF for the reaction. After completion of the reaction followed by work up and silica gel column chromatography with EtOAc : Hexane (1:2) as eluent, afforded benzophosphole oxide **3o** (43.8 mg, 77%) as a colourless oil.

^{1}H NMR (400 MHz, $CDCl_3$) δ 7.85 (ddd, J = 11.9, 8.2, 1.5 Hz, 1H), 7.72 – 7.67 (m, 1H), 7.60 – 7.53 (m, 1H), 7.50 (td, J = 6.1, 5.6, 3.1 Hz, 2H), 7.40 – 7.37 (m, 1H), 7.33 – 7.29 (m, 1H), 7.15 (dd, J = 5.2, 2.0 Hz, 1H), 7.02 (dd, J = 7.5, 3.5 Hz, 1H), 4.24 – 4.08 (m, 2H), 2.07 – 1.93 (m, 3H), 1.40 (q, J = 7.0 Hz, 3H) ppm.

^{13}C{^{1}H} NMR (101 MHz, $CDCl_3$) δ 149.3 (d, J = 28.3 Hz), 144.1 (d, J = 10.1 Hz), 142.3 (d, J = 16.3 Hz), 142.1 (d, J = 8.3 Hz), 135.8 (d, J = 20.3 Hz), 133.4, 133.2 (d, J = 2.6 Hz), 132.9 (d, J = 2.2 Hz), 132.4 (d, J = 4.3 Hz), 132.1 (d, J = 2.7 Hz), 131.7 (d, J = 9.9 Hz), 131.6 (d, J = 2.9 Hz), 131.5 (d, J = 10.4 Hz), 131.3 (d, J = 7.4 Hz), 130.9 (d, J = 11.9 Hz), 130.6 (d, J = 9.8 Hz), 129.9 (d, J = 7.6 Hz),

129.5 (d, J = 8.7 Hz), 129.0 (d, J = 2.0 Hz), 128.8, 128.7 (d, J = 3.3 Hz), 128.6 (d, J = 2.6 Hz), 128.5, 128.4 (d, J = 4.1 Hz), 128.3 (d, J = 3.6 Hz), 128.0 (d, J = 5.9 Hz), 127.9 (d, J = 5.0 Hz), 127.8, 127.6 (d, J = 3.9 Hz), 127.5 (d, J = 1.7 Hz), 126.6 (d, J = 26.9 Hz), 123.1 (d, J = 13.5 Hz), 121.9 (d, J = 13.5 Hz), 61.9 (d, J = 6.2 Hz), 16.5 (d, J = 6.5 Hz), 10.7 (d, J = 9.6 Hz) ppm.

^{31}P NMR (162 MHz, CDCl$_3$) δ 48.4 ppm.

HRMS (ESI) m/z calcd. for $C_{17}H_{18}O_2P^+$ $(M+H)^+$: 285.1039, found : 285.1042.

1-ethoxy-2,3-dipropylphosphindole 1-oxide (3p)

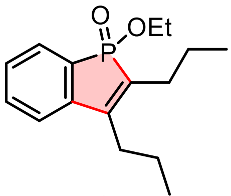

3p

According to the General Procedure for the Photoelectrochemical Annulation, ethyl phenylphosphinate (60.3 µL, 0.4 mmol, 2.0 equiv.), 4-octyne (29.34 µL, 0.2 mmol, 1.0 equiv.), **PC$_1$** (8 mg, 5 mol%, 0.01 equiv.), TBAPF$_6$ (77.49 mg, 0.2 mmol, 1.0 equiv.) were dissolved in DMF for the reaction. After completion of the reaction followed by work up and silica gel column chromatography with EtOAc : Hexane (1:2) as eluent, afforded benzophosphole oxide **3p** (41.2 mg, 74%) as a colourless oil.

^{1}H NMR (400 MHz, CDCl$_3$) δ 7.78 – 7.69 (m, 1H), 7.60 – 7.54 (m, 1H), 7.47 – 7.40 (m, 2H), 4.06 – 3.94 (m, 2H), 2.49 (dd, J = 9.1, 6.7 Hz, 1H), 2.19 – 2.08 (m, 2H), 1.67 (tdd, J = 10.2, 8.1, 4.7 Hz, 1H), 1.59 – 1.43 (m, 4H), 0.99 (q, J = 7.0 Hz, 3H), 0.95 – 0.86 (m, 3H), 0.84 – 0.66 (m, 3H) ppm.

^{13}C{^{1}H} NMR (101 MHz, CDCl$_3$) δ 149.5 (d, J = 25.9 Hz), 146.6 (d, J = 9.5 Hz), 141.6 (d, J = 36.4 Hz), 132.8 (d, J = 2.2 Hz), 132.7 (d, J = 3.5 Hz), 132.6 (d, J = 3.4 Hz), 131.7 (d, J = 9.9 Hz), 128.5 (d, J = 4.0 Hz), 128.3 (d, J = 3.6 Hz), 128.1 (d, J = 10.9 Hz), 127.1 (d, J = 8.7 Hz), 126.9, 121.3 (d, J = 13.6 Hz), 61.5 (d, J = 6.2 Hz), 28.3 (d, J = 16.0 Hz), 27.9 (d, J = 9.9 Hz), 22.0 (d, J = 2.1 Hz), 21.6 (d, J = 2.4 Hz), 16.5 (d, J = 6.4 Hz), 14.4 (d, J = 7.3 Hz), 13.9 (d, J = 2.5 Hz) ppm.

^{31}P NMR (162 MHz, CDCl$_3$) δ 49.3 ppm.

HRMS (ESI) m/z calcd. for $C_{16}H_{24}O_2P^+$ $(M+H)^+$: 279.1508, found : 279.1512.

1-methyl-2,3-diphenylphosphindole 1-oxide (3q)

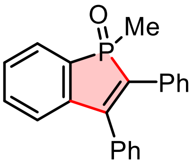

3q

According to the General Procedure for the Photoelectrochemical Annulation, methyl(phenyl)phosphine oxide (56.05 mg, 0.4 mmol, 2.0 equiv.), diphenylacetylene (35.64 mg, 0.2 mmol, 1.0 equiv.), **PC$_1$** (8 mg, 5 mol%, 0.01 equiv.), TBAPF$_6$ (77.49 mg, 0.2 mmol, 1.0 equiv.) were dissolved in DMF for the reaction. After completion of the reaction followed by work up and silica gel column chromatography with EtOAc : Hexane (1:1) as eluent, afforded benzophosphole oxide **3q** (51.9 mg, 82%) as a pale-yellow oil.

^{1}H NMR (400 MHz, CDCl$_3$) δ 7.89 – 7.83 (m, 1H), 7.47 – 7.37 (m, 7H), 7.29 (d, *J* = 7.8 Hz, 1H), 7.26 – 7.15 (m, 5H), 1.75 (d, *J* = 13.1 Hz, 3H) ppm.

^{13}C{^{1}H} NMR (101 MHz, CDCl$_3$) δ 148.1 (d, *J* = 21.4 Hz), 142.7 (d, *J* = 27.0 Hz), 134.5, 134.2 (d, *J* = 14.7 Hz), 133.6, 132.9, 132.8 (d, *J* = 1.9 Hz), 132.1, 131.7, 131.1, 130.8 (d, *J* = 9.5 Hz), 130.2, 129.3, 129.0 (d, *J* = 6.5 Hz), 128.9, 128.8, 128.6 (d, *J* = 13.2 Hz), 128.3 (d, *J* = 9.7 Hz), 128.0, 124.0 (d, *J* = 10.7 Hz), 14.8 (d, *J* = 69.3 Hz) ppm.

^{31}P NMR (162 MHz, CDCl$_3$) δ 46.1 ppm.

HRMS (ESI) m/z calcd. for $C_{21}H_{18}OP^+$ $(M+H)^+$: 317.1090, found : 317.1094.

1-methyl-2,3-di-*p*-tolylphosphindole 1-oxide (3r)

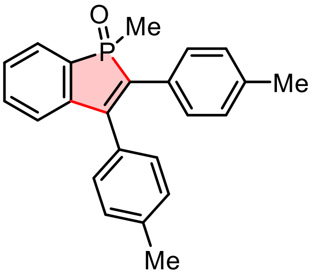

3r

According to the General Procedure for the Photoelectrochemical Annulation, methyl(phenyl)phosphine oxide (56.05 mg, 0.4 mmol, 2.0 equiv.), 1,2-di-*p*-tolylethyne (41.26 mg, 0.2 mmol, 1.0 equiv.), **PC$_1$** (8 mg, 5 mol%, 0.01 equiv.), TBAPF$_6$ (77.49 mg, 0.2 mmol, 1.0 equiv.) were dissolved in DMF for the reaction. After completion of the reaction followed by work up and silica gel column chromatography with EtOAc : Hexane (1:1) as eluent, afforded benzophosphole oxide **3r** (58.5 mg, 85%) as a pale-yellow gum.

^{1}H NMR (400 MHz, CDCl$_3$) δ 7.87 – 7.82 (m, 1H), 7.44 – 7.38 (m, 2H), 7.32 – 7.28 (m, 2H), 7.21 (d, *J* = 7.9 Hz, 2H), 7.18 – 7.13 (m, 3H), 7.04 – 7.00 (m, 2H), 2.39 (s, 3H), 2.28 (s, 3H), 1.73 (d, *J* = 13.1 Hz, 3H) ppm.

^{13}C{^{1}H} NMR (101 MHz, CDCl$_3$) δ 147.5 (d, *J* = 21.4 Hz), 143.1 (d, *J* = 27.1 Hz), 138.2 (d, *J* = 50.8 Hz), 133.4 (d, *J* = 94.4 Hz), 132.7 (d, *J* = 2.1 Hz), 132.0, 131.4 (d, *J* = 5.7 Hz), 131.2 (d, *J* = 2.0 Hz), 130.9, 130.0 (d, *J* = 10.3 Hz), 129.7, 129.3, 129.2, 129.1, 129.0, 128.8 (d, *J* = 5.6 Hz), 128.6 (d, *J* = 10.4 Hz), 128.4, 128.2 (d, *J* = 9.8 Hz), 128.0 (d, *J* = 11.8 Hz), 123.9 (d, *J* = 10.6 Hz), 21.4, 21.3, 14.8 (d, *J* = 69.1 Hz) ppm.

^{31}P NMR (162 MHz, CDCl$_3$) δ 46.5 ppm.

HRMS (ESI) m/z calcd. for $C_{23}H_{22}OP^+$ $(M+H)^+$: 345.1403, found : 345.1406.

1-ethyl-2,3-diphenylphosphindole 1-oxide (3s)[8]

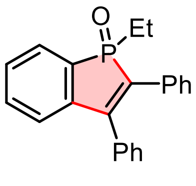

3s

According to the General Procedure for the Photoelectrochemical Annulation, ethyl(phenyl)phosphine oxide (61.66 mg, 0.4 mmol, 2.0 equiv.), diphenylacetylene (35.64 mg, 0.2 mmol, 1.0 equiv.), **PC$_1$** (8 mg, 5 mol%, 0.01 equiv.), TBAPF$_6$ (77.49 mg, 0.2 mmol, 1.0 equiv.) were dissolved in DMF for the reaction. After completion of the reaction followed by work up and silica gel column chromatography with EtOAc : Hexane (1:1) as eluent, afforded benzophosphole oxide **3s** (52.2 mg, 79%) as a colourless oil.

^{1}H NMR (400 MHz, CDCl$_3$) δ 7.90 – 7.75 (m, 1H), 7.52 – 7.32 (m, 7H), 7.31 – 7.22 (m, 2H), 7.24 – 6.97 (m, 4H), 2.14 (tt, J = 15.3, 7.6 Hz, 1H), 1.95 (ddq, J = 15.2, 13.2, 7.6 Hz, 1H), 0.98 (dt, J = 18.9, 7.6 Hz, 3H) ppm.

^{13}C{^{1}H} NMR (101 MHz, CDCl$_3$) δ 149.2 (d, J = 20.0 Hz), 143.5 (d, J = 25.6 Hz), 134.2 (d, J = 14.3 Hz), 133.5, 133.2 (d, J = 9.9 Hz), 133.0, 132.7 (d, J = 2.0 Hz), 132.1, 130.5, 129.5, 129.5 (d, J = 4.8 Hz), 129.0 (d, J = 8.0 Hz), 128.9, 128.8 (d, J = 9.9 Hz), 128.6 (d, J = 4.7 Hz), 128.5, 128.2, 128.0, 123.9 (d, J = 10.3 Hz), 21.5 (d, J = 68.3 Hz), 6.1 (d, J = 3.6 Hz) ppm.

^{31}P NMR (162 MHz, CDCl$_3$) δ 53.3 ppm.

HRMS (ESI) m/z calcd. for $C_{22}H_{20}OP^+$ $(M+H)^+$: 331.1246, found : 331.1248.

1-ethyl-2,3-di-*p*-tolylphosphindole 1-oxide (3t)

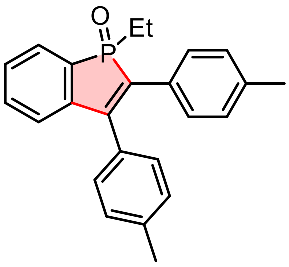

3t

According to the General Procedure for the Photoelectrochemical Annulation, ethyl(phenyl)phosphine oxide (61.66 mg, 0.4 mmol, 2.0 equiv.), 1,2-di-*p*-tolylethyne (41.26 mg, 0.2 mmol, 1.0 equiv.), **PC$_1$** (8 mg, 5 mol%, 0.01 equiv.), TBAPF$_6$ (77.49 mg, 0.2 mmol, 1.0 equiv.) were dissolved in DMF for the reaction. After completion of the reaction followed by work up and silica gel column chromatography with EtOAc : Hexane (1:1) as eluent, afforded benzophosphole oxide **3t** (57.3 mg, 80%) as a pale-yellow gum.

^{1}H NMR (400 MHz, CDCl$_3$) δ 7.83 – 7.76 (m, 1H), 7.69 – 7.48 (m, 1H), 7.44 – 7.36 (m, 2H), 7.32 – 7.28 (m, 2H), 7.20 (d, J = 7.8 Hz, 2H), 7.17 – 7.11 (m, 2H), 7.01 (d, J = 8.1 Hz, 2H), 2.38 (s, 3H), 2.27 (s, 3H), 2.18 – 2.07 (m, 1H), 1.93 (ddq, J = 15.2, 13.3, 7.6 Hz, 1H), 0.95 (dt, J = 18.9, 7.6 Hz, 3H) ppm.

$^{13}C\{^1H\}$ **NMR (101 MHz, CDCl3)** δ 148.6 (d, *J* = 20.1 Hz), 144.4 (d, *J* = 7.9 Hz), 143.8 (d, *J* = 25.6 Hz), 138.4, 137.9, 132.7 (d, *J* = 1.8 Hz), 132.4, 132.0 (d, *J* = 5.4 Hz), 131.9 (d, *J* = 5.3 Hz), 131.6 (d, *J* = 20.4 Hz), 131.4 (d, *J* = 14.7 Hz), 130.4 (d, *J* = 6.1 Hz), 130.3, 129.6, 129.5, 129.3 (d, *J* = 7.1 Hz), 129.2, 129.1, 129.0, 128.8 (d, *J* = 5.6 Hz), 128.6 (d, *J* = 6.3 Hz), 128.5 (d, *J* = 7.3 Hz), 128.2 (d, *J* = 11.5 Hz), 128.0 (d, *J* = 11.6 Hz), 123.8 (d, *J* = 10.3 Hz), 21.5 (d, *J* = 68.6 Hz), 21.3, 6.1 (d, *J* = 3.5 Hz) ppm.

^{31}P **NMR (162 MHz, CDCl3)** δ 53.5 ppm.

HRMS (ESI) m/z calcd. for $C_{24}H_{24}OP^+$ $(M+H)^+$: 359.1559, found : 359.1565.

1,2,3-triphenylphosphindole 1-sulfide (3u)[7]

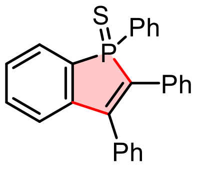

3u

According to the General Procedure for the Photoelectrochemical Annulation, diphenylphosphine sulfide (87.30 mg, 0.4 mmol, 2.0 equiv.), diphenylacetylene (35.64 mg, 0.2 mmol, 1.0 equiv.), **PC1** (8 mg, 5 mol%, 0.01 equiv.), $TBAPF_6$ (77.49 mg, 0.2 mmol, 1.0 equiv.) were dissolved in DMF for the reaction. After completion of the reaction followed by work up and silica gel column chromatography with EtOAc : Hexane (1:2) as eluent, afforded benzophosphole oxide **3u** (48.1 mg, 61%) as a white solid.

^1H **NMR (400 MHz, CDCl3)** δ 7.77 (ddd, *J* = 12.6, 8.3, 1.5 Hz, 2H), 7.73 – 7.69 (m, 1H), 7.49 – 7.31 (m, 10H), 7.24 – 7.19 (m, 3H), 7.08 (dd, *J* = 5.1, 2.0 Hz, 3H) ppm.

$^{13}C\{^1H\}$ **NMR (101 MHz, CDCl3)** δ 150.2 (d, *J* = 21.6 Hz), 143.8 (d, *J* = 27.2 Hz), 134.5, 134.2 (d, *J* = 15.1 Hz), 133.6, 133.0 (d, *J* = 2.0 Hz), 132.6 (d, *J* = 9.9 Hz), 132.4, 132.3 (d, *J* = 2.9 Hz), 131.3, 131.0 (d, *J* = 10.8 Hz), 130.2, 129.2 (d, *J* = 3.4 Hz), 129.1 (d, *J* = 2.6 Hz), 129.1 (d, *J* = 5.6 Hz), 129.0 (d, *J* = 1.4 Hz), 128.8 (d, *J* = 11.3 Hz), 128.3, 127.9, 124.1 (d, *J* = 11.0 Hz) ppm.

^{31}P **NMR (162 MHz, CDCl3)** δ 48.1 ppm.

HRMS (ESI) m/z calcd. for $C_{26}H_{20}PS^+$ $(M+H)^+$: 395.1018, found : 395.1023.

1-phenyl-2,3-dipropylphosphindole 1-sulfide (3v)

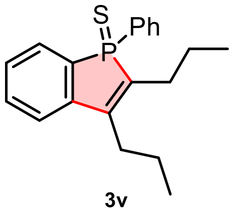

3v

According to the General Procedure for the Photoelectrochemical Annulation, diphenylphosphine sulfide (87.30 mg, 0.4 mmol, 2.0 equiv.), 4-octyne (29.34 μL, 0.2 mmol, 1.0 equiv.), **PC₁** (8 mg, 5 mol%, 0.01 equiv.), $TBAPF_6$ (77.49 mg, 0.2 mmol, 1.0 equiv.) were dissolved in DMF for the reaction. After completion of the reaction followed by work up and silica gel column chromatography with EtOAc : Hexane (1:2) as eluent, afforded benzophosphole oxide **3v** (43.7 mg, 67%) as a colourless oil.

^{1}H NMR (400 MHz, $CDCl_3$) δ 7.71 – 7.61 (m, 2H), 7.56 – 7.45 (m, 3H), 7.37 (tdd, *J* = 8.4, 7.0, 2.9 Hz, 3H), 7.29 – 7.23 (m, 1H), 2.64 – 2.55 (m, 2H), 2.47 (dddd, *J* = 16.2, 14.1, 9.1, 7.0 Hz, 1H), 2.31 – 2.18 (m, 1H), 1.64 (qd, *J* = 7.4, 2.2 Hz, 2H), 1.42 (tq, *J* = 9.2, 7.0 Hz, 2H), 1.05 (t, *J* = 7.4 Hz, 3H), 0.84 (t, *J* = 7.3 Hz, 3H) ppm.

^{13}C{^{1}H} NMR (101 MHz, $CDCl_3$) δ 150.3 (d, *J* = 19.8 Hz), 143.6 (d, *J* = 29.3 Hz), 135.2, 134.2, 132.8, 132.8 (d, *J* = 2.1 Hz), 132.0, 131.9 (d, *J* = 2.9 Hz), 131.7 (d, *J* = 11.4 Hz), 131.5 (d, *J* = 5.8 Hz), 130.9 (d, *J* = 10.8 Hz), 129.9, 128.7 (d, *J* = 12.1 Hz), 128.5, 128.4 (d, *J* = 11.8 Hz), 128.2 (d, *J* = 10.5 Hz), 121.4 (d, *J* = 11.3 Hz), 28.6 (d, *J* = 13.3 Hz), 28.3 (d, *J* = 10.8 Hz), 22.3 (d, *J* = 2.0 Hz), 21.8 (d, *J* = 2.0 Hz), 14.4, 14.4 ppm.

^{31}P NMR (162 MHz, $CDCl_3$) δ 47.9 ppm.

HRMS (ESI) m/z calcd. for $C_{20}H_{24}PS^+$ $(M+H)^+$: 327.1331, found : 327.1327.

1-phenyl-2,3-di-*p*-tolylphosphindole 1-oxide (4a)[7]

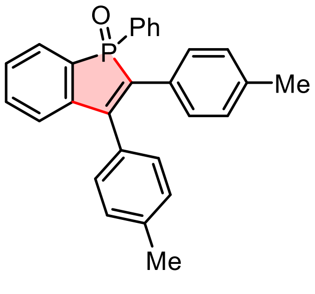

4a

According to the General Procedure for the Photoelectrochemical Annulation, diphenylphosphine oxide (80.87 mg, 0.4 mmol, 2.0 equiv.), 1,2-di-*p*-tolylethyne (41.26 mg, 0.2 mmol, 1.0 equiv.), **PC₁** (8 mg, 5 mol%, 0.01 equiv.), $TBAPF_6$ (77.49 mg, 0.2 mmol, 1.0 equiv.) were dissolved in DMF for the reaction. After completion of the reaction followed by work up and silica gel column chromatography with EtOAc : Hexane (2:3) as eluent, afforded benzophosphole oxide **4a** (75.6 mg, 93%) as a pale-yellow solid.

^{1}H NMR (400 MHz, $CDCl_3$) δ 7.80 (ddd, *J* = 12.4, 8.3, 1.4 Hz, 2H), 7.74 – 7.68 (m, 1H), 7.51 – 7.31 (m, 7H), 7.26 – 7.22 (m, 3H), 7.21 – 7.17 (m, 2H), 6.93 (d, *J* = 8.0 Hz, 2H), 2.43 (s, 3H), 2.21 (s, 3H) ppm.

^{13}C{^{1}H} NMR (101 MHz, $CDCl_3$) δ 149.5 (d, *J* = 21.6 Hz), 144.0 (d, *J* = 27.3 Hz), 138.1 (d, *J* = 84.9 Hz), 133.6 (d, *J* = 96.2 Hz), 132.9 (d, *J* = 2.0 Hz), 132.1 (d, *J* = 2.8 Hz), 131.5 (d, *J* = 3.6 Hz), 131.3,

131.0 (d, J = 10.7 Hz), 130.6, 129.8 (d, J = 9.9 Hz), 129.7, 129.6, 129.4 (d, J = 5.7 Hz), 129.1, 129.0 (d, J = 5.2 Hz), 128.9 (d, J = 1.7 Hz), 128.9 (d, J = 1.6 Hz), 128.8 (d, J = 6.2 Hz), 128.6, 128.2 (d, J = 15.1 Hz), 123.9 (d, J = 10.8 Hz), 21.4, 21.2 ppm.

^{31}P NMR (162 MHz, CDCl$_3$) δ 40.0 ppm.

HRMS (ESI) m/z calcd. for $C_{28}H_{24}OP^+$ $(M+H)^+$: 407.1559, found : 407.1563.

2,3-bis(4-ethylphenyl)-1-phenylphosphindole 1-oxide (4b)[9]

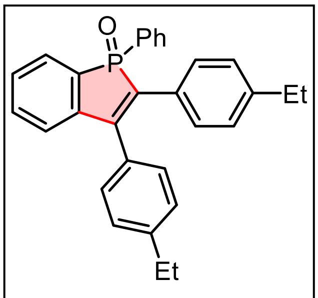

4b

According to the General Procedure for the Photoelectrochemical Annulation, diphenylphosphine oxide (80.87 mg, 0.4 mmol, 2.0 equiv.), 1,2-bis(4-ethylphenyl)ethyne (46.87 mg, 0.2 mmol, 1.0 equiv.), **PC$_1$** (8 mg, 5 mol%, 0.01 equiv.), TBAPF$_6$ (77.49 mg, 0.2 mmol, 1.0 equiv.) were dissolved in DMF for the reaction. After completion of the reaction followed by work up and silica gel column chromatography with EtOAc : Hexane (2:3) as eluent, afforded benzophosphole oxide **4b** (74.7 mg, 86%) as a pale-yellow solid.

^{1}H NMR (400 MHz, CDCl$_3$) δ 7.79 (ddd, J = 12.5, 8.3, 1.4 Hz, 2H), 7.70 – 7.65 (m, 1H), 7.48 – 7.34 (m, 5H), 7.34 – 7.27 (m, 2H), 7.24 – 7.14 (m, 4H), 6.92 (d, J = 8.1 Hz, 3H), 2.71 (q, J = 7.6 Hz, 2H), 2.49 (q, J = 7.6 Hz, 2H), 1.28 (t, J = 7.6 Hz, 3H), 1.10 (t, J = 7.6 Hz, 3H) ppm.

^{13}C{^{1}H} NMR (101 MHz, CDCl$_3$) δ 148.4 (d, J = 21.5 Hz), 143.7, 143.1 (d, J = 27.3 Hz), 142.8, 132.6 (d, J = 96.2 Hz), 131.8 (d, J = 2.0 Hz), 131.5, 131.0 (d, J = 2.9 Hz), 130.7 (d, J = 2.9 Hz), 130.5 (d, J = 4.4 Hz), 129.9 (d, J = 10.5 Hz), 129.4 (d, J = 65.5 Hz), 128.8 (d, J = 23.6 Hz), 128.0 (d, J = 3.0 Hz), 127.9 (d, J = 4.8 Hz), 127.8 (d, J = 1.7 Hz), 127.7, 127.4, 127.1, 126.7, 122.9 (d, J = 11.0 Hz), 27.7, 27.4, 14.3, 13.9 ppm.

^{31}P NMR (162 MHz, CDCl$_3$) δ 40.0 ppm.

HRMS (ESI) m/z calcd. for $C_{30}H_{28}OP^+$ $(M+H)^+$: 435.1872, found : 435.1871.

2,3-bis(4-methoxyphenyl)-1-phenylphosphindole 1-oxide (4c)[7]

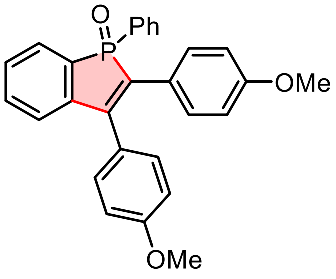

4c

According to the General Procedure for the Photoelectrochemical Annulation, diphenylphosphine oxide (80.87 mg, 0.4 mmol, 2.0 equiv.), 1,2-bis(4-methoxyphenyl)ethyne (47.66 mg, 0.2 mmol, 1.0 equiv.), **PC$_1$** (8 mg, 5 mol%, 0.01 equiv.), TBAPF$_6$ (77.49 mg, 0.2 mmol, 1.0 equiv.) were dissolved in DMF for the reaction. After completion of the reaction followed by work up and silica gel column chromatography with EtOAc : Hexane (3:2) as eluent, afforded benzophosphole oxide **4c** (71.9 mg, 82%) as a yellowish-green solid.

^{1}H NMR (400 MHz, CDCl$_3$) δ 7.80 – 7.74 (m, 2H), 7.70 – 7.65 (m, 1H), 7.48 – 7.31 (m, 5H), 7.29 – 7.21 (m, 5H), 6.97 (d, *J* = 8.8 Hz, 2H), 6.64 (d, *J* = 8.8 Hz, 2H), 3.84 (s, 3H), 3.67 (s, 3H) ppm.

^{13}C{^{1}H} NMR (101 MHz, CDCl$_3$) δ 159.8, 159.1, 148.2 (d, *J* = 21.8 Hz), 144.2 (d, *J* = 27.2 Hz), 133.5, 132.8 (d, *J* = 1.9 Hz), 132.6 (d, *J* = 3.0 Hz), 132.5 (d, *J* = 13.4 Hz), 132.1 (d, *J* = 2.8 Hz), 131.4, 130.9 (d, *J* = 10.7 Hz), 130.8 (d, *J* = 1.5 Hz), 130.7, 130.5 (d, *J* = 2.5 Hz), 130.4, 129.8, 129.0 (d, *J* = 4.8 Hz), 128.9, 128.9 (d, *J* = 1.6 Hz), 128.8 (d, *J* = 1.6 Hz), 128.7, 126.5 (d, *J* = 15.5 Hz), 125.3 (d, *J* = 10.2 Hz), 123.7 (d, *J* = 10.9 Hz), 114.5, 113.8, 55.3, 55.1 ppm.

^{31}P NMR (162 MHz, CDCl$_3$) δ 39.8 ppm.

HRMS (ESI) m/z calcd. for $C_{28}H_{24}O_3P^+$ $(M+H)^+$: 439.1458, found : 439.1464.

2,3-bis(4-fluorophenyl)-1-phenylphosphindole 1-oxide (4d)[8]

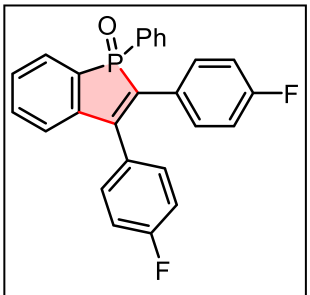

4d

According to the General Procedure for the Photoelectrochemical Annulation, diphenylphosphine oxide (80.87 mg, 0.4 mmol, 2.0 equiv.), 1,2-bis(4-fluorophenyl)ethyne (42.84 mg, 0.2 mmol, 1.0 equiv.), **PC$_1$** (8 mg, 5 mol%, 0.01 equiv.), TBAPF$_6$ (77.49 mg, 0.2 mmol, 1.0 equiv.) were dissolved in DMF for the reaction. After completion of the reaction followed by work up and silica gel column chromatography with EtOAc : Hexane (1:2) as eluent, afforded benzophosphole oxide **4d** (58.8 mg, 71%) as a pale-yellow oil.

^{1}H NMR (400 MHz, CDCl$_3$) δ 7.85 – 7.76 (m, 3H), 7.54 (ddq, *J* = 7.7, 4.1, 1.5 Hz, 2H), 7.49 – 7.42 (m, 3H), 7.31 – 7.25 (m, 2H), 7.21 (t, *J* = 8.8 Hz, 5H), 6.87 (t, *J* = 8.7 Hz, 2H) ppm.

^{13}C{^{1}H} NMR (101 MHz, CDCl$_3$) δ 163.8 (d, *J* = 57.9 Hz), 161.3 (d, *J* = 57.7 Hz), 148.9 (d, *J* = 21.8 Hz), 143.4 (d, *J* = 26.7 Hz), 133.8 (d, *J* = 96.1 Hz), 132.8 (dd, *J* = 71.2, 2.5 Hz), 131.7 (d, *J* = 106.0 Hz), 131.0 (d, *J* = 7.3 Hz), 130.9 (d, *J* = 4.5 Hz), 130.8 (d, *J* = 5.6 Hz), 129.9 (d, *J* = 4.3 Hz), 129.7 (d,

J = 3.6 Hz), 129.3 (t, J = 10.4 Hz), 129.0 (d, J = 12.5 Hz), 128.6 (d, J = 3.5 Hz), 128.5 (d, J = 3.4 Hz), 123.9 (d, J = 10.8 Hz), 116.3 (d, J = 21.7 Hz), 115.5 (d, J = 21.6 Hz) ppm.

^{31}P NMR (162 MHz, CDCl$_3$) δ 39.6 ppm.

^{19}F NMR (376 MHz, CDCl$_3$) δ -112.1, -113.0 (d, J = 1.6 Hz) ppm.

HRMS (ESI) m/z calcd. for $C_{26}H_{18}F_2OP^+$ $(M+H)^+$: 415.1058, found : 415.1061.

2,3-bis(4-chlorophenyl)-1-phenylphosphindole 1-oxide (4e)[7]

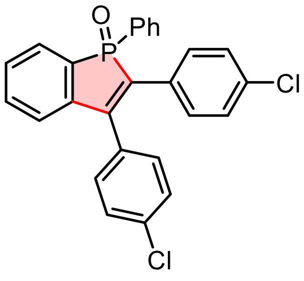

4e

According to the General Procedure for the Photoelectrochemical Annulation, diphenylphosphine oxide (80.87 mg, 0.4 mmol, 2.0 equiv.), 1,2-bis(4-chlorophenyl)ethyne (49.42 mg, 0.2 mmol, 1.0 equiv.), **PC$_1$** (8 mg, 5 mol%, 0.01 equiv.), TBAPF$_6$ (77.49 mg, 0.2 mmol, 1.0 equiv.) were dissolved in DMF for the reaction. After completion of the reaction followed by work up and silica gel column chromatography with EtOAc : Hexane (1:2) as eluent, afforded benzophosphole oxide **4e** (74.2 mg, 83%) as a pale-yellow solid.

^{1}H NMR (400 MHz, CDCl$_3$) δ 7.73 – 7.66 (m, 3H), 7.47 – 7.42 (m, 2H), 7.41 – 7.33 (m, 5H), 7.23 (d, J = 8.0 Hz, 2H), 7.16 (ddd, J = 8.8, 4.9, 2.1 Hz, 3H), 7.05 (d, J = 8.6 Hz, 2H) ppm.

^{13}C{^{1}H} NMR (101 MHz, CDCl$_3$) δ 149.1 (d, J = 21.8 Hz), 143.1 (d, J = 26.5 Hz), 135.0, 134.2 (d, J = 25.1 Hz), 133.4, 133.2 (d, J = 2.1 Hz), 132.5 (d, J = 2.9 Hz), 132.3, 132.2 (d, J = 6.6 Hz), 131.0 (d, J = 2.3 Hz), 130.9 (d, J = 3.0 Hz), 130.4, 130.2 (d, J = 5.4 Hz), 129.6 (d, J = 6.2 Hz), 129.5, 129.3 (d, J = 9.7 Hz), 129.0 (d, J = 12.5 Hz), 128.7, 128.7, 124.0 (d, J = 10.9 Hz) ppm.

^{31}P NMR (162 MHz, CDCl$_3$) δ 39.7 ppm.

HRMS (ESI) m/z calcd. for $C_{26}H_{18}Cl_2OP^+$ $(M+H)^+$: 447.0467, found : 447.0470.

2,3-bis(4-bromophenyl)-1-phenylphosphindole 1-oxide (4f)[7]

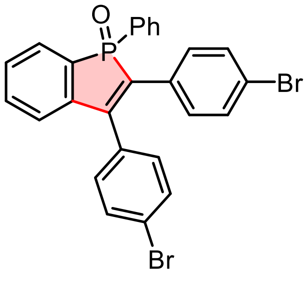

4f

According to the General Procedure for the Photoelectrochemical Annulation, diphenylphosphine oxide (80.87 mg, 0.4 mmol, 2.0 equiv.), 1,2-bis(4-bromophenyl)ethyne (67.2 mg, 0.2 mmol, 1.0 equiv.), **PC_1** (8 mg, 5 mol%, 0.01 equiv.), $TBAPF_6$ (77.49 mg, 0.2 mmol, 1.0 equiv.) were dissolved in DMF for the reaction. After completion of the reaction followed by work up and silica gel column chromatography with EtOAc : Hexane (1:2) as eluent, afforded benzophosphole oxide **4f** (83.6 mg, 78%) as a pale-yellow solid.

^{1}H NMR (400 MHz, $CDCl_3$) δ 7.66 – 7.59 (m, 2H), 7.51 – 7.47 (m, 1H), 7.41 – 7.37 (m, 4H), 7.33 – 7.26 (m, 5H), 7.16 (d, J = 9.0 Hz, 2H), 7.10 (d, J = 7.9 Hz, 2H), 7.01 (dd, J = 8.6, 1.2 Hz, 1H) ppm.

$^{13}C\{^1H\}$ NMR (101 MHz, $CDCl_3$) δ 149.2 (d, J = 21.7 Hz), 143.1 (d, J = 26.4 Hz), 133.9 (d, J = 95.5 Hz), 133.2 (d, J = 2.2 Hz), 133.0, 132.7 (d, J = 15.1 Hz), 132.5 (d, J = 2.9 Hz), 132.5, 132.2, 131.7 (d, J = 3.0 Hz), 131.4 (d, J = 9.9 Hz), 131.2, 130.9 (d, J = 10.6 Hz), 130.7, 130.5 (d, J = 5.5 Hz), 129.6 (d, J = 11.1 Hz), 129.4 (d, J = 9.7 Hz), 129.0 (d, J = 12.5 Hz), 128.6, 124.0 (d, J = 10.8 Hz), 123.0 (d, J = 44.2 Hz), 122.5, 121.9 ppm.

^{31}P NMR (162 MHz, $CDCl_3$) δ 39.6 ppm.

HRMS (ESI) m/z calcd. for $C_{26}H_{18}Br_2OP^+$ $(M+H)^+$: 534.9457, found : 534.9455.

1-phenyl-2,3-bis(4-(trifluoromethyl)phenyl)phosphindole 1-oxide (4g)[8]

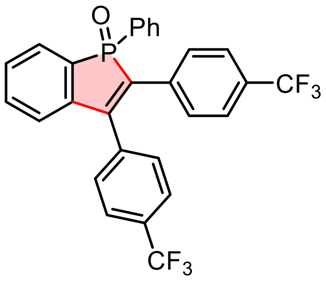

4g

According to the General Procedure for the Photoelectrochemical Annulation, diphenylphosphine oxide (80.87 mg, 0.4 mmol, 2.0 equiv.), 1,2-bis(4-(trifluoromethyl)phenyl)ethyne (62.85 mg, 0.2 mmol, 1.0 equiv.), **PC_1** (8 mg, 5 mol%, 0.01 equiv.), $TBAPF_6$ (77.49 mg, 0.2 mmol, 1.0 equiv.) were dissolved in DMF for the reaction. After completion of the reaction followed by work up and silica gel column chromatography with EtOAc : Hexane (1:2) as eluent, afforded benzophosphole oxide **4g** (91.6 mg, 89%) as a yellow oil.

^{1}H NMR (400 MHz, $CDCl_3$) δ 7.76 – 7.68 (m, 5H), 7.51 – 7.42 (m, 4H), 7.42 – 7.36 (m, 3H), 7.33 (q, J = 8.5 Hz, 4H), 7.16 (dt, J = 7.6, 2.0 Hz, 1H) ppm.

$^{13}C\{^1H\}$ NMR (101 MHz, $CDCl_3$) δ 150.0 (d, J = 21.5 Hz), 142.7 (d, J = 26.3 Hz), 137.4 (d, J = 14.7 Hz), 135.9 (d, J = 9.2 Hz), 134.5 (d, J = 94.8 Hz), 133.4 (d, J = 2.2 Hz), 132.7 (d, J = 2.9 Hz), 132.2, 131.6, 131.3, 131.0 (d, J = 11.7 Hz), 130.9 (d, J = 10.8 Hz), 130.5 (d, J = 25.3 Hz), 130.1, 130.0 (d, J

= 10.8 Hz), 129.8, 129.6, 129.5, 129.2 (d, J = 5.1 Hz), 129.1, 128.2, 127.8, 126.2 (q, J = 3.6 Hz), 125.4 (q, J = 3.8 Hz), 125.1, 124.2 (d, J = 10.7 Hz), 122.4 (d, J = 1.7 Hz), 119.7 ppm.

^{31}P NMR (162 MHz, CDCl$_3$) δ 39.8 ppm.

^{19}F NMR (376 MHz, CDCl$_3$) δ -63.3, -63.4 ppm.

HRMS (ESI) m/z calcd. for $C_{28}H_{18}F_6OP^+$ $(M+H)^+$: 515.0994, found : 515.1002.

(1-oxido-1-phenylphosphindole-2,3-diyl)bis(diphenylphosphine oxide) (4h)

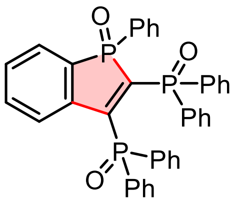

4h

According to the General Procedure for the Photoelectrochemical Annulation, diphenylphosphine oxide (80.87 mg, 0.4 mmol, 2.0 equiv.), 1,2-bis(diphenylphosphaneyl)ethyne (78.88 mg, 0.2 mmol, 1.0 equiv.), **PC$_1$** (8 mg, 5 mol%, 0.01 equiv.), TBAPF$_6$ (77.49 mg, 0.2 mmol, 1.0 equiv.) were dissolved in DMF for the reaction. After completion of the reaction followed by work up and silica gel column chromatography with EtOAc : Hexane (2:1) as eluent, afforded benzophosphole oxide **4h** (91.6 mg, 71%) as a yellow oil.

^{1}H NMR (400 MHz, CDCl$_3$) δ 7.97 – 7.73 (m, 3H), 7.70 (ddt, J = 13.8, 7.0, 1.5 Hz, 4H), 7.68 – 7.26 (m, 15H), 7.27 – 7.12 (m, 6H), 7.01 – 6.86 (m, 1H) ppm.

^{13}C{^{1}H} NMR (101 MHz, CDCl$_3$) δ 162.60, 133.39, 133.23, 132.93, 132.65, 132.62, 132.25, 132.00, 131.92, 131.82, 131.79, 131.69, 131.65, 131.60, 131.54, 131.38, 131.35, 131.12, 131.05, 130.80, 130.78, 130.69, 130.58, 130.48, 130.37, 130.28, 129.93, 129.00, 128.87, 128.56, 128.52, 128.44, 128.40, 128.38, 128.32, 128.26, 128.20, 128.16, 128.07, 127.61, 127.46 ppm.

^{31}P NMR (162 MHz, CDCl$_3$) δ 33.1, 33.0, 32.0, 31.9, 22.4 ppm.

HRMS (ESI) m/z calcd. for $C_{38}H_{30}O_3P_3^+$ $(M+H)^+$: 627.1402, found : 627.1410.

1-phenyl-2,3-dipropylphosphindole 1-oxide (4i)[7]

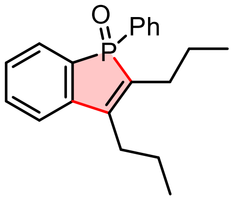

4i

According to the General Procedure for the Photoelectrochemical Annulation, diphenylphosphine oxide (80.87 mg, 0.4 mmol, 2.0 equiv.), 4-octyne (29.34 µL, 0.2 mmol, 1.0 equiv.), **PC$_1$** (8 mg, 5 mol%, 0.01 equiv.), TBAPF$_6$ (77.49 mg, 0.2 mmol, 1.0 equiv.) were dissolved in DMF for the reaction. After completion of the reaction followed by work up and silica gel column chromatography with EtOAc : Hexane (1:2) as eluent, afforded benzophosphole oxide **4i** (46.56 mg, 75%) as a colourless oil.

^{1}**H NMR (400 MHz, CDCl$_3$)** δ 7.40 (ddd, *J* = 12.3, 8.3, 1.4 Hz, 2H), 7.32 – 7.24 (m, 1H), 7.24 – 7.18 (m, 2H), 7.16 – 7.06 (m, 3H), 6.99 (tdd, *J* = 7.4, 3.8, 1.0 Hz, 1H), 2.51 – 2.23 (m, 2H), 2.23 (dddd, *J* = 16.2, 14.2, 9.1, 7.0 Hz, 1H), 2.00 (tdd, *J* = 14.8, 9.2, 6.7 Hz, 1H), 1.39 (hd, *J* = 7.3, 1.6 Hz, 2H), 1.27 – 1.09 (m, 2H), 0.79 (t, *J* = 7.4 Hz, 3H), 0.59 (t, *J* = 7.3 Hz, 3H) ppm.

^{13}**C{^{1}H} NMR (101 MHz, CDCl$_3$)** δ 150.4 (d, *J* = 19.9 Hz), 143.5 (d, *J* = 29.4 Hz), 134.5 (d, *J* = 96.0 Hz), 132.8 (d, *J* = 2.0 Hz), 132.7, 131.9 (d, *J* = 2.7 Hz), 131.6, 130.8 (d, *J* = 10.7 Hz), 130.7, 129.7, 128.7 (d, *J* = 12.1 Hz), 128.5 (d, *J* = 9.6 Hz), 128.2 (d, *J* = 10.6 Hz), 121.4 (d, *J* = 11.4 Hz), 28.5 (d, *J* = 13.2 Hz), 28.2 (d, *J* = 10.8 Hz), 22.3 (d, *J* = 2.1 Hz), 21.8 (d, *J* = 2.1 Hz), 14.4, 14.3 ppm.

^{31}**P NMR (162 MHz, CDCl$_3$)** δ 40.6 ppm.

HRMS (ESI) m/z calcd. for $C_{20}H_{24}OP^+$ $(M+H)^+$: 311.1559, found : 311.1562.

2,3-di([1,1'-biphenyl]-4-yl)-1-phenylphosphindole 1-oxide (4j)

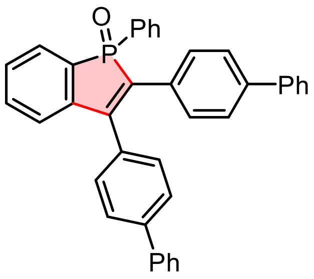

4j

According to the General Procedure for the Photoelectrochemical Annulation, diphenylphosphine oxide (80.87 mg, 0.4 mmol, 2.0 equiv.), 1,2-di([1,1'-biphenyl]-4-yl)ethyne (66.08 mg, 0.2 mmol, 1.0 equiv.), **PC$_1$** (8 mg, 5 mol%, 0.01 equiv.), $TBAPF_6$ (77.49 mg, 0.2 mmol, 1.0 equiv.) were dissolved in DMF for the reaction. After completion of the reaction followed by work up and silica gel column chromatography with EtOAc : Hexane (1:2) as eluent, afforded benzophosphole oxide **4j** (94.4 mg, 89%) as a yellowish-green gum.

^{1}**H NMR (400 MHz, CDCl$_3$)** δ 7.87 (dd, *J* = 12.6, 7.5 Hz, 2H), 7.72 (td, *J* = 20.0, 8.0 Hz, 5H), 7.51 – 7.24 (m, 20H) ppm.

^{13}**C{^{1}H} NMR (101 MHz, CDCl$_3$)** δ 149.8 (d, *J* = 21.7 Hz), 143.8 (d, *J* = 27.0 Hz), 141.0 (d, *J* = 108.4 Hz), 140.2 (d, *J* = 5.3 Hz), 133.3, 133.1 (d, *J* = 3.5 Hz), 132.6 – 132.2 (m), 131.7 (d, *J* = 9.9 Hz), 131.4, 131.0 (d, *J* = 10.5 Hz), 130.3, 129.6, 129.5 (d, *J* = 5.7 Hz), 129.3 (d, *J* = 2.6 Hz), 129.2 (d, *J* = 9.0 Hz), 129.1, 128.8 (d, *J* = 23.5 Hz), 127.8 (d, *J* = 11.0 Hz), 127.5, 127.0 (d, *J* = 8.7 Hz), 126.8, 124.2 (d, *J* = 10.7 Hz) ppm.

^{31}**P NMR (162 MHz, CDCl$_3$)** δ 40.2 ppm.

HRMS (ESI) m/z calcd. for $C_{38}H_{28}OP^+$ $(M+H)^+$: 531.1872, found : 531.1873.

1-phenyl-2,3-di(thiophen-2-yl)phosphindole 1-oxide (4k)[11]

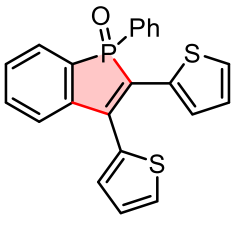

4k

According to the General Procedure for the Photoelectrochemical Annulation, diphenylphosphine oxide (80.87 mg, 0.4 mmol, 2.0 equiv.), 1,2-di(thiophen-2-yl)ethyne (38.06 mg, 0.2 mmol, 1.0 equiv.), **PC_1** (8 mg, 5 mol%, 0.01 equiv.), $TBAPF_6$ (77.49 mg, 0.2 mmol, 1.0 equiv.) were dissolved in DMF for the reaction. After completion of the reaction followed by work up and silica gel column chromatography with EtOAc : Hexane (1:2) as eluent, afforded benzophosphole oxide **4k** (65.6 mg, 84%) as a bright yellow oil.

^{1}H NMR (400 MHz, $CDCl_3$) δ 7.84 – 7.70 (m, 4H), 7.65 – 7.62 (m, 1H), 7.56 (dd, *J* = 7.4, 1.7 Hz, 1H), 7.52 – 7.48 (m, 2H), 7.45 – 7.43 (m, 2H), 7.30 – 7.27 (m, 1H), 7.20 – 7.16 (m, 2H), 7.00 (dd, *J* = 5.2, 3.7 Hz, 1H), 6.87 (dd, *J* = 5.1, 3.7 Hz, 1H) ppm.

^{13}C{^{1}H} NMR (101 MHz, $CDCl_3$) δ 144.5 (d, *J* = 25.1 Hz), 139.1 (d, *J* = 23.4 Hz), 136.7 (d, *J* = 8.4 Hz), 134.9 (d, *J* = 14.3 Hz), 133.4 (d, *J* = 2.2 Hz), 133.3 (d, *J* = 16.7 Hz), 132.8, 132.6 (d, *J* = 9.1 Hz), 132.5 (d, *J* = 3.1 Hz), 131.8 (d, *J* = 10.6 Hz), 131.7, 131.6 (d, *J* = 6.5 Hz), 131.1, 130.9 (d, *J* = 10.9 Hz), 130.8 (d, *J* = 11.5 Hz), 130.4, 130.0 (d, *J* = 4.4 Hz), 129.4, 129.1 (d, *J* = 3.5 Hz), 129.0, 129.0, 128.9, 128.9 (d, *J* = 5.3 Hz), 128.7, 128.6, 128.5, 128.3, 127.3, 127.0, 123.7 (d, *J* = 10.9 Hz), 122.1 ppm.

^{31}P NMR (162 MHz, $CDCl_3$) δ 38.8 ppm.

HRMS (ESI) m/z calcd. for $C_{22}H_{16}OPS_2^+$ $(M+H)^+$: 391.0375, found : 391.0379.

2-(4-(1-oxido-1,2-diphenylphosphindol-3-yl)phenyl)-1,3-diphenylphosphindole 1-oxide : 2,2'-(1,4-phenylene)bis(1,3-diphenylphosphindole 1-oxide) (4l : 4l' = 1 : 0.54) - regioisomeric mixture inseparable in column chromatography.

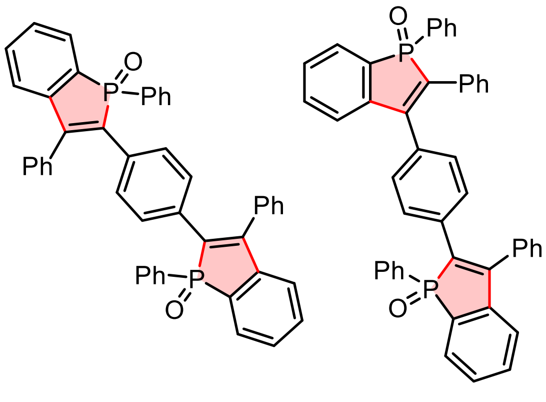

4l **4l'**

According to the General Procedure for the Photoelectrochemical Annulation, diphenylphosphine oxide (161.74 mg, 0.8 mmol, 4.0 equiv.), 1,4-bis(phenylethynyl)benzene (55.67 mg, 0.2 mmol, 1.0 equiv.), **PC_1** (8 mg, 5 mol%, 0.01 equiv.), $TBAPF_6$ (77.49 mg, 0.2 mmol, 1.0 equiv.) were dissolved in DMF for the reaction. After completion of the reaction followed by work up and silica gel column chromatography with EtOAc : Hexane (1:1) as eluent, afforded benzophosphole oxides **4l, 4l'** (105.9 mg, 78%) as a yellow gum.

^{1}H NMR (400 MHz, CDCl$_3$) δ 7.94 – 7.56 (m, 12H), 7.51 – 7.19 (m, 24.17H), 7.14 – 6.93 (m, 12.21H) ppm.

^{13}C{^{1}H} NMR (101 MHz, CDCl$_3$) δ 143.3 (d, *J* = 25.8 Hz), 134.4 (d, *J* = 98.8 Hz), 133.0 (d, *J* = 17.1 Hz), 132.3 (d, *J* = 15.1 Hz), 131.7 (d, *J* = 17.3 Hz), 130.9, 130.7, 129.8, 129.5 (d, *J* = 25.1 Hz), 129.1 (d, *J* = 4.0 Hz), 129.0, 128.2 (d, *J* = 16.3 Hz), 127.8 (d, *J* = 9.6 Hz), 124.0 ppm.

^{31}P NMR (162 MHz, CDCl$_3$) δ 39.8, 26.4 ppm.

HRMS (ESI) m/z calcd. for $C_{46}H_{33}O_2P_2^+$ $(M+H)^+$: 679.1950, found : 679.1955.

2-methyl-1,3-diphenylphosphindole 1-oxide (4m)[7]

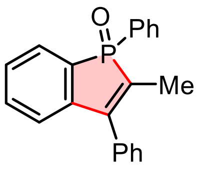

4m

According to the General Procedure for the Photoelectrochemical Annulation, diphenylphosphine oxide (161.74 mg, 0.8 mmol, 4.0 equiv.), prop-1-yn-1-ylbenzene (24.9 μL, 0.2 mmol, 1.0 equiv.), **PC$_1$** (8 mg, 5 mol%, 0.01 equiv.), $TBAPF_6$ (77.49 mg, 0.2 mmol, 1.0 equiv.) were dissolved in DMF for the reaction. After completion of the reaction followed by work up and silica gel column chromatography with EtOAc : Hexane (1:2) as eluent, afforded benzophosphole oxide **4m** (54.4 mg, 86%) as a pale-yellow oil.

^{1}H NMR (400 MHz, CDCl$_3$) δ 7.72 – 7.62 (m, 2H), 7.60 – 7.54 (m, 1H), 7.52 – 7.42 (m, 2H), 7.42 – 7.30 (m, 5H), 7.30 – 7.21 (m, 3H), 7.03 (dd, *J* = 7.6, 3.0 Hz, 1H), 1.82 (d, *J* = 12.5 Hz, 3H) ppm.

^{13}C{^{1}H} NMR (101 MHz, CDCl$_3$) δ 150.1 (d, *J* = 22.0 Hz), 144.3 (d, *J* = 28.2 Hz), 133.6 (d, *J* = 15.7 Hz), 133.2, 132.9 (d, *J* = 2.1 Hz), 132.5, 132.3 (d, *J* = 2.8 Hz), 132.1, 132.0, 131.9 (d, *J* = 9.5 Hz), 131.5 (d, *J* = 6.3 Hz), 131.0 (d, *J* = 10.7 Hz), 129.7, 129.4, 129.1 (d, *J* = 9.5 Hz), 128.8 (d, *J* = 11.0 Hz), 128.6 (d, *J* = 4.9 Hz), 128.6, 128.4 (d, *J* = 10.3 Hz), 123.2 (d, *J* = 11.2 Hz), 10.7 (d, *J* = 10.9 Hz) ppm.

^{31}P NMR (162 MHz, CDCl$_3$) δ 41.2 ppm.

HRMS (ESI) m/z calcd. for $C_{21}H_{18}OP^+$ $(M+H)^+$: 317.1090, found : 317.1092.

2-ethyl-1,3-diphenylphosphindole 1-oxide (4n)[8]

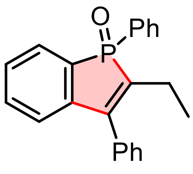

4n

According to the General Procedure for the Photoelectrochemical Annulation, diphenylphosphine oxide (161.74 mg, 0.8 mmol, 4.0 equiv.), but-1-yn-1-ylbenzene (28.42 μL, 0.2 mmol, 1.0 equiv.), **PC$_1$** (8 mg, 5 mol%, 0.01 equiv.), $TBAPF_6$ (77.49 mg, 0.2 mmol, 1.0 equiv.) were dissolved in DMF for the reaction. After completion of the reaction followed by work up and silica gel column chromatography with

EtOAc : Hexane (1:2) as eluent, afforded benzophosphole oxide **4n** (54.2 mg, 82%) as a pale-yellow oil.

^{1}H NMR (400 MHz, CDCl$_3$) δ 7.76 – 7.61 (m, 2H), 7.57 – 7.49 (m, 1H), 7.47 – 7.39 (m, 3H), 7.39 – 7.33 (m, 3H), 7.33 – 7.27 (m, 1H), 7.27 – 7.10 (m, 4H), 6.95 (dd, *J* = 7.6, 2.9 Hz, 1H), 2.38 (ddd, *J* = 15.8, 14.5, 7.6 Hz, 1H), 2.25 – 2.10 (m, 1H), 0.87 (t, *J* = 7.6 Hz, 3H) ppm.

^{13}C{^{1}H} NMR (101 MHz, CDCl$_3$) δ 150.1 (d, *J* = 22.2 Hz), 144.1 (d, *J* = 27.9 Hz), 137.7 (d, *J* = 94.0 Hz), 133.7 (d, *J* = 15.9 Hz), 133.0, 132.7 (d, *J* = 2.1 Hz), 132.2, 132.0 (d, *J* = 2.9 Hz), 131.8 (d, *J* = 9.5 Hz), 131.3 (d, *J* = 9.1 Hz), 131.2, 130.8 (d, *J* = 10.8 Hz), 130.4, 129.4, 128.8 (d, *J* = 5.2 Hz), 128.7 (d, *J* = 2.4 Hz), 128.5, 128.5 (d, *J* = 5.2 Hz), 128.4 (d, *J* = 4.3 Hz), 128.2, 123.2 (d, *J* = 10.9 Hz), 19.9 (d, *J* = 10.3 Hz), 13.7 (d, *J* = 2.4 Hz) ppm.

^{31}P NMR (162 MHz, CDCl$_3$) δ 40.7 ppm.

HRMS (ESI) m/z calcd. for $C_{22}H_{20}OP^+$ $(M+H)^+$: 331.1246, found : 331.1250.

methyl 1,3-diphenylphosphindole-2-carboxylate 1-oxide (4o)[9]

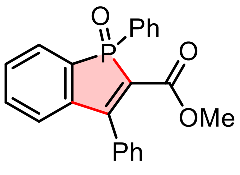

4o

According to the General Procedure for the Photoelectrochemical Annulation, diphenylphosphine oxide (161.74 mg, 0.8 mmol, 4.0 equiv.), methyl 3-phenylpropiolate (29.5 μL, 0.2 mmol, 1.0 equiv.), **PC$_1$** (8 mg, 5 mol%, 0.01 equiv.), $TBAPF_6$ (77.49 mg, 0.2 mmol, 1.0 equiv.) were dissolved in DMF for the reaction. After completion of the reaction followed by work up and silica gel column chromatography with EtOAc : Hexane (1:2) as eluent, afforded benzophosphole oxide **4o** (43.9 mg, 61%) as a pale-yellow oil.

^{1}H NMR (400 MHz, CDCl$_3$) δ 7.86 – 7.70 (m, 5H), 7.52 (dd, *J* = 5.3, 1.9 Hz, 3H), 7.49 – 7.45 (m, 3H), 7.38 (dd, *J* = 5.4, 2.4 Hz, 2H), 7.24 – 7.20 (m, 1H), 3.58 (s, 3H) ppm.

^{13}C{^{1}H} NMR (101 MHz, CDCl$_3$) δ 148.6 (d, *J* = 4.9 Hz), 142.2 (d, *J* = 25.1 Hz), 133.9, 133.6 (d, *J* = 13.2 Hz), 133.2 (d, *J* = 2.0 Hz), 133.1 (d, *J* = 1.8 Hz), 132.9, 132.6, 132.5 (d, *J* = 3.0 Hz), 132.3 (d, *J* = 2.9 Hz), 132.1 (d, *J* = 10.5 Hz), 132.0, 131.9 (d, *J* = 10.8 Hz), 131.7, 131.2 (d, *J* = 9.5 Hz), 131.0 (d, *J* = 11.1 Hz), 131.0 (d, *J* = 10.7 Hz), 130.5 (d, *J* = 21.4 Hz), 129.6 (d, *J* = 9.6 Hz), 129.5 (d, *J* = 5.8 Hz), 129.3, 129.1 (d, *J* = 2.6 Hz), 128.8 (d, *J* = 13.0 Hz), 128.7 (d, *J* = 3.9 Hz), 128.6 (d, *J* = 5.2 Hz), 128.5, 128.4 (d, *J* = 6.8 Hz), 127.9, 127.8 (d, *J* = 5.3 Hz), 127.6, 126.5 (d, *J* = 10.6 Hz), 126.3, 125.3, 123.9, 52.0 ppm.

^{31}P NMR (162 MHz, CDCl$_3$) δ 36.6 ppm.

HRMS (ESI) m/z calcd. for $C_{22}H_{18}O_3P^+$ $(M+H)^+$: 361.0988, found : 361.0988.

ethyl 1,3-diphenylphosphindole-2-carboxylate 1-oxide (4p)[7]

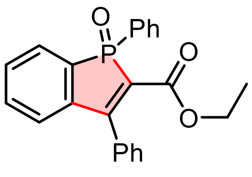

4p

According to the General Procedure for the Photoelectrochemical Annulation, diphenylphosphine oxide (161.74 mg, 0.8 mmol, 4.0 equiv.), methyl 3-phenylpropiolate (33.02 µL, 0.2 mmol, 1.0 equiv.), **PC_1** (8 mg, 5 mol%, 0.01 equiv.), $TBAPF_6$ (77.49 mg, 0.2 mmol, 1.0 equiv.) were dissolved in DMF for the reaction. After completion of the reaction followed by work up and silica gel column chromatography with EtOAc : Hexane (1:2) as eluent, afforded benzophosphole oxide **4p** (50.2 mg, 67%) as a pale-yellow oil.

^{1}H NMR (400 MHz, $CDCl_3$) δ 7.88 – 7.75 (m, 4H), 7.69 – 7.65 (m, 1H), 7.55 (dt, *J* = 7.2, 1.6 Hz, 2H), 7.51 – 7.46 (m, 3H), 7.37 (dd, *J* = 5.3, 1.9 Hz, 2H), 7.30 – 7.28 (m, 2H), 3.93 (q, *J* = 7.1 Hz, 2H), 0.89 (t, *J* = 7.2 Hz, 3H) ppm.

^{13}C{^{1}H} NMR (101 MHz, $CDCl_3$) δ 164.0 (d, *J* = 18.6 Hz), 162.4 (d, *J* = 12.2 Hz), 142.2 (d, *J* = 25.2 Hz), 134.2 (d, *J* = 7.6 Hz), 133.3, 133.2 (d, *J* = 2.1 Hz), 133.1, 133.1 (d, *J* = 5.0 Hz), 132.9 (d, *J* = 7.7 Hz), 132.7 (d, *J* = 2.8 Hz), 132.5 (d, *J* = 2.9 Hz), 132.2, 132.1 (d, *J* = 9.4 Hz), 132.0 (d, *J* = 4.0 Hz), 131.8 (d, *J* = 4.1 Hz), 131.7, 131.5, 131.1 (d, *J* = 11.2 Hz), 130.7 (d, *J* = 11.5 Hz), 130.5, 129.6 (d, *J* = 9.6 Hz), 129.4 (d, *J* = 6.2 Hz), 129.1 (d, *J* = 15.3 Hz), 128.9 (d, *J* = 13.0 Hz), 128.7 (d, *J* = 13.0 Hz), 128.5 (d, *J* = 5.8 Hz), 128.4 (d, *J* = 4.8 Hz), 128.3, 128.1 (d, *J* = 11.1 Hz), 127.9 (d, *J* = 5.4 Hz), 127.6 (d, *J* = 12.9 Hz), 126.7 (d, *J* = 6.1 Hz), 126.5 (d, *J* = 10.5 Hz), 125.7, 60.8, 13.6 ppm.

^{31}P NMR (162 MHz, $CDCl_3$) δ 35.8 ppm.

HRMS (ESI) m/z calcd. for $C_{23}H_{20}O_3P^+$ $(M+H)^+$: 375.1145, found : 375.1149.

ethyl 3-(4-chlorophenyl)-1-phenylphosphindole-2-carboxylate 1-oxide (4q)

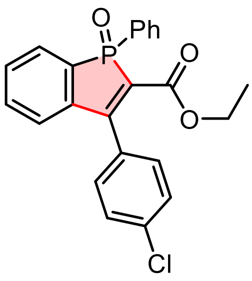

4q

According to the General Procedure for the Photoelectrochemical Annulation, diphenylphosphine oxide (161.74 mg, 0.8 mmol, 4.0 equiv.), ethyl 3-(4-chlorophenyl)propiolate (41.73 mg, 0.2 mmol, 1.0 equiv.), **PC_1** (8 mg, 5 mol%, 0.01 equiv.), $TBAPF_6$ (77.49 mg, 0.2 mmol, 1.0 equiv.) were dissolved in DMF for the reaction. After completion of the reaction followed by work up and silica gel column chromatography with EtOAc : Hexane (1:2) as eluent, afforded benzophosphole oxide **4q** (56.4 mg, 69%) as a colourless oil.

^{1}H NMR (400 MHz, $CDCl_3$) δ 7.88 – 7.75 (m, 4H), 7.69 – 7.65 (m, 1H), 7.55 (dt, J = 7.2, 1.6 Hz, 2H), 7.51 – 7.46 (m, 3H), 7.37 (dd, J = 5.3, 1.9 Hz, 2H), 7.30 – 7.28 (m, 2H), 3.93 (q, J = 7.1 Hz, 2H), 0.89 (t, J = 7.2 Hz, 3H) ppm.

^{13}C{^{1}H} NMR (101 MHz, $CDCl_3$) δ 165.0 (d, J = 18.6 Hz), 163.4 (d, J = 12.2 Hz), 143.2 (d, J = 25.2 Hz), 135.2 (d, J = 7.6 Hz), 134.3, 134.2 (d, J = 2.1 Hz), 134.1, 134.1 (d, J = 5.0 Hz), 133.9 (d, J = 7.7 Hz), 133.7 (d, J = 2.8 Hz), 133.5 (d, J = 2.9 Hz), 132.8 (d, J = 4.1 Hz), 132.7, 132.5, 132.1 (d, J = 11.2 Hz), 131.7 (d, J = 11.5 Hz), 131.5, 130.6 (d, J = 9.6 Hz), 130.4 (d, J = 6.2 Hz), 130.1 (d, J = 15.3 Hz), 129.9 (d, J = 13.0 Hz), 129.7 (d, J = 13.0 Hz), 129.4, 129.3, 129.1 (d, J = 11.1 Hz), 128.9 (d, J = 5.4 Hz), 128.6 (d, J = 12.9 Hz), 127.7 (d, J = 6.1 Hz), 127.5 (d, J = 10.5 Hz), 126.7, 61.8, 14.6 ppm.

^{31}P NMR (162 MHz, $CDCl_3$) δ 39.8 ppm.

HRMS (ESI) m/z calcd. for $C_{23}H_{19}ClO_3P^+$ $(M+H)^+$: 409.0755, found : 409.0758.

methyl 1-phenyl-3-(pyridin-2-yl)phosphindole-2-carboxylate 1-oxide (4r)

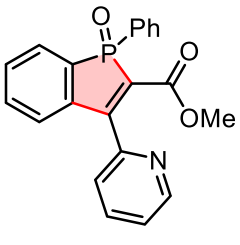

4r

According to the General Procedure for the Photoelectrochemical Annulation, diphenylphosphine oxide (161.74 mg, 0.8 mmol, 4.0 equiv.), methyl 3-(pyridin-2-yl)propiolate (32.23 mg, 0.2 mmol, 1.0 equiv.), **PC_1** (8 mg, 5 mol%, 0.01 equiv.), $TBAPF_6$ (77.49 mg, 0.2 mmol, 1.0 equiv.) were dissolved in DMF for the reaction. After completion of the reaction followed by work up and silica gel column chromatography with EtOAc : Hexane (2:1) as eluent, afforded benzophosphole oxide **4r** (40.5 mg, 56%) as a brown gum.

^{1}H NMR (400 MHz, $CDCl_3$) δ 8.67 – 8.59 (m, 1H), 8.55 (ddd, J = 4.7, 1.9, 0.9 Hz, 2H), 7.97 – 7.89 (m, 3H), 7.80 – 7.76 (m, 2H), 7.56 – 7.52 (m, 3H), 7.49 – 7.45 (m, 2H), 3.97 (s, 3H) ppm.

^{13}C{^{1}H} NMR (101 MHz, $CDCl_3$) δ 150.5, 149.1, 136.8, 136.7, 133.5, 132.3, 132.0, 131.9, 131.0, 130.9, 129.0, 128.7, 128.6, 124.9, 123.5, 122.6, 52.7 ppm.

^{31}P NMR (162 MHz, $CDCl_3$) δ 44.3 ppm.

HRMS (ESI) m/z calcd. for $C_{21}H_{17}NO_3P^+$ $(M+H)^+$: 362.0941, found : 362.0943.

3-(4-methoxyphenyl)-2-(4-nitrophenyl)-1-phenylphosphindole 1-oxide : 2-(4-methoxyphenyl)-3-(4-nitrophenyl)-1-phenylphosphindole 1-oxide (4s : 4s' = 1 : 0.84) - regioisomeric mixture inseparable in column chromatography.

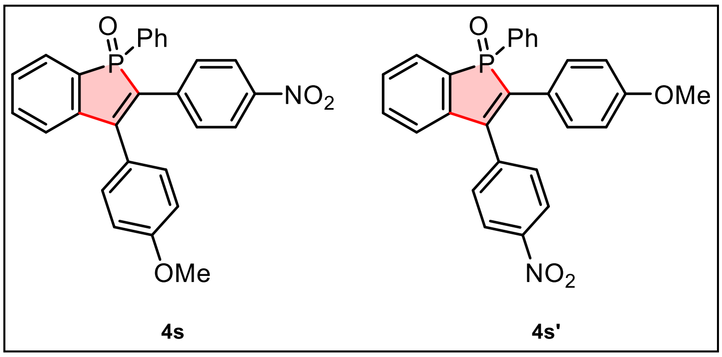

According to the General Procedure for the Photoelectrochemical Annulation, diphenylphosphine oxide (161.74 mg, 0.8 mmol, 4.0 equiv.), 1-methoxy-4-((4-nitrophenyl)ethynyl)benzene (50.65 mg, 0.2 mmol, 1.0 equiv.), **PC_1** (8 mg, 5 mol%, 0.01 equiv.), $TBAPF_6$ (77.49 mg, 0.2 mmol, 1.0 equiv.) were dissolved in DMF for the reaction. After completion of the reaction followed by work up and silica gel column chromatography with EtOAc : Hexane (2:1) as eluent, afforded benzophosphole oxides **4s**, **4s'** (51.7 mg, 57%) as a dark orange-red gum.

^{1}H NMR (400 MHz, $CDCl_3$) δ 7.86 (dd, J = 12.5, 7.6 Hz, 2H), 7.79 – 7.61 (m, 5H), 7.56 – 7.35 (m, 10H), 7.27 – 7.19 (m, 2H), 7.17 – 7.04 (m, 3H), 6.98 – 6.78 (m, 4H), 6.64 (d, J = 8.8 Hz, 1H), 3.80 (s, 2H), 3.69 (s, 1H) ppm.

^{13}C{^{1}H} NMR (101 MHz, $CDCl_3$) δ 159.4, 140.4, 133.2, 132.9, 132.5 (d, J = 11.7 Hz), 132.0 (d, J = 10.1 Hz), 131.0, 130.4, 129.1, 128.8 (d, J = 12.9 Hz), 127.8, 124.4, 123.6, 118.4 (d, J = 6.5 Hz), 116.7, 115.6, 114.1 (d, J = 21.2 Hz), 55.3, 55.1 ppm.

^{31}P NMR (162 MHz, $CDCl_3$) δ 39.8, 24.9 ppm.

HRMS (ESI) m/z calcd. for $C_{27}H_{21}NO_4P^+$ $(M+H)^+$: 454.1203, found : 454.1200.

1,3-diphenyl-2-(trimethylsilyl)phosphindole 1-oxide (4t)[7]

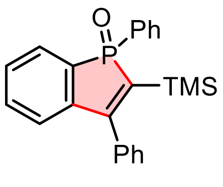

According to the General Procedure for the Photoelectrochemical Annulation, diphenylphosphine oxide (161.74 mg, 0.8 mmol, 4.0 equiv.), trimethyl(phenylethynyl)silane (39.34 µL, 0.2 mmol, 1.0 equiv.), **PC_1** (8 mg, 5 mol%, 0.01 equiv.), $TBAPF_6$ (77.49 mg, 0.2 mmol, 1.0 equiv.) were dissolved in DMF for the reaction. After completion of the reaction followed by work up and silica gel column chromatography with EtOAc : Hexane (1:2) as eluent, afforded benzophosphole oxide **4t** (62.9 mg, 84%) as a colourless oil.

^{1}H NMR (400 MHz, $CDCl_3$) δ 7.93 – 7.86 (m, 1H), 7.76 (ddd, J = 12.3, 8.2, 1.4 Hz, 2H), 7.64 – 7.58 (m, 1H), 7.51 – 7.42 (m, 6H), 7.39 – 7.34 (m, 1H), 7.33 – 7.28 (m, 2H), 6.92 (ddt, J = 8.3, 2.0, 0.8 Hz, 1H), -0.15 (s, 9H) ppm.

^{13}C{^{1}H} NMR (101 MHz, $CDCl_3$) δ 159.4, 140.4, 133.2, 132.9, 132.5 (d, J = 11.7 Hz), 132.0 (d, J = 10.1 Hz), 131.0, 130.4, 129.1, 128.8 (d, J = 12.9 Hz), 127.8, 124.4, 123.6, 118.4 (d, J = 6.5 Hz), 116.7, 115.6, 114.1 (d, J = 21.2 Hz), 55.3 ppm.

^{31}P NMR (162 MHz, $CDCl_3$) δ 47.1, 8.0 ppm.

HRMS (ESI) m/z calcd. for $C_{23}H_{24}OPSi^+$ $(M+H)^+$: 375.1329, found : 375.1329.

2-(2-hydroxypropan-2-yl)-1,3-diphenylphosphindole 1-oxide (4u)[7]

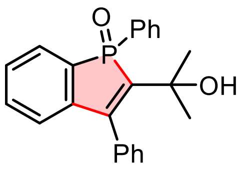

According to the General Procedure for the Photoelectrochemical Annulation, diphenylphosphine oxide (161.74 mg, 0.8 mmol, 4.0 equiv.), 2-methyl-4-phenylbut-3-yn-2-ol (32.04 mg, 0.2 mmol, 1.0 equiv.), **PC_1** (8 mg, 5 mol%, 0.01 equiv.), $TBAPF_6$ (77.49 mg, 0.2 mmol, 1.0 equiv.) were dissolved in DMF for the reaction. After completion of the reaction followed by work up and silica gel column chromatography with EtOAc : Hexane (1:1) as eluent, afforded benzophosphole oxide **4u** (67.0 mg, 68%) as a colourless gum.

^{1}H NMR (400 MHz, $CDCl_3$) δ 7.81 – 7.74 (m, 2H), 7.72 – 7.60 (m, 1H), 7.57 – 7.53 (m, 1H), 7.51 – 7.47 (m, 3H), 7.46 – 7.40 (m, 3H), 7.34 (ddd, J = 7.5, 6.0, 1.6 Hz, 1H), 7.28 (dtd, J = 6.5, 3.8, 2.0 Hz, 1H), 7.26 – 7.15 (m, 1H), 6.70 – 6.65 (m, 1H), 3.22 (broad s, 1H), 1.30 (s, 3H), 1.11 (s, 3H) ppm.

^{13}C{^{1}H} NMR (101 MHz, $CDCl_3$) δ 148.8 (d, J = 20.4 Hz), 145.1 (d, J = 28.3 Hz), 144.5 (d, J = 92.0 Hz), 134.7 (d, J = 16.3 Hz), 132.9 (d, J = 1.9 Hz), 132.6 (d, J = 2.9 Hz), 132.1, 132.0 (d, J = 3.0 Hz), 131.8 (d, J = 6.0 Hz), 131.3 (d, J = 10.4 Hz), 131.0 (d, J = 11.1 Hz), 130.8 (d, J = 7.0 Hz), 130.7, 130.1, 129.2 (d, J = 22.4 Hz), 129.0 (d, J = 2.9 Hz), 128.9, 128.8 (d, J = 7.9 Hz), 128.6 (d, J = 4.0 Hz), 128.5, 128.4 (d, J = 2.0 Hz), 128.3 (d, J = 1.5 Hz), 128.2, 127.6 (d, J = 23.9 Hz), 123.6 (d, J = 11.1 Hz), 73.4 (d, J = 6.9 Hz), 31.8 (d, J = 2.4 Hz), 30.8 (d, J = 2.2 Hz) ppm.

^{31}**P NMR (162 MHz, CDCl$_3$)** δ 41.6 ppm.

HRMS (ESI) m/z calcd. for $C_{23}H_{22}O_2P^+$ $(M+H)^+$: 361.1352, found : 361.1353.

1-(1-oxido-1,3-diphenylphosphindol-2-yl)ethan-1-one (4v)[7]

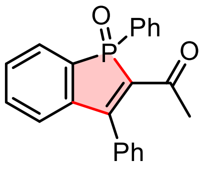

4v

According to the General Procedure for the Photoelectrochemical Annulation, diphenylphosphine oxide (161.74 mg, 0.8 mmol, 4.0 equiv.), 4-phenylbut-3-yn-2-one (29.13 µL, 0.2 mmol, 1.0 equiv.), **PC$_1$** (8 mg, 5 mol%, 0.01 equiv.), TBAPF$_6$ (77.49 mg, 0.2 mmol, 1.0 equiv.) were dissolved in DMF for the reaction. After completion of the reaction followed by work up and silica gel column chromatography with EtOAc : Hexane (1:1) as eluent, afforded benzophosphole oxide **4v** (51.0 mg, 74%) as a colourless gum.

^{1}**H NMR (400 MHz, CDCl$_3$)** δ 7.80 – 7.69 (m, 3H), 7.54 (dd, J = 5.4, 1.7 Hz, 3H), 7.50 (ddd, J = 6.1, 3.2, 2.1 Hz, 2H), 7.44 (tdd, J = 7.2, 2.7, 1.1 Hz, 3H), 7.36 (dd, J = 7.1, 2.5 Hz, 2H), 7.19 – 7.16 (m, 1H), 1.92 (s, 3H) ppm.

^{13}**C{^{1}H} NMR (101 MHz, CDCl$_3$)** δ 195.8 (d, J = 9.0 Hz), 160.1 (d, J = 19.0 Hz), 142.4 (d, J = 26.0 Hz), 135.3 (d, J = 92.9 Hz), 133.6, 133.4 (d, J = 13.5 Hz), 133.2 (d, J = 2.1 Hz), 133.1, 132.6, 132.5 (d, J = 3.0 Hz), 132.4, 132.1, 131.7 (d, J = 10.6 Hz), 131.5 (d, J = 10.8 Hz), 131.0 (d, J = 11.2 Hz), 130.8 (d, J = 6.1 Hz), 130.7, 129.8, 129.7 (d, J = 3.2 Hz), 129.6, 129.5 (d, J = 9.4 Hz), 129.3 (d, J = 10.3 Hz), 129.1 (d, J = 3.9 Hz), 128.9 (d, J = 12.9 Hz), 128.7, 128.6 (d, J = 2.8 Hz), 128.4 (d, J = 5.3 Hz), 128.3, 128.1 (d, J = 5.3 Hz), 127.9, 126.5 (d, J = 10.7 Hz), 123.7 (d, J = 10.5 Hz), 30.7 (d, J = 2.9 Hz) ppm.

^{31}**P NMR (162 MHz, CDCl$_3$)** δ 37.6 ppm.

HRMS (ESI) m/z calcd. for $C_{22}H_{18}O_2P^+$ $(M+H)^+$: 345.1039, found : 345.1038.

22. References:

(1) Speckmeier, E.; Fischer, T. G.; Zeitler, K. A toolbox approach to construct broadly applicable Metal-Free catalysts for photoredox chemistry: Deliberate tuning of redox potentials and importance of halogens in Donor–Acceptor Cyanoarenes. *J. Am. Chem. Soc.* **2018**, *140*, 15353–15365.

(2) Huang, W.; Byun, J.; Rörich, I.; Ramanan, C.; Blom, P. W. M.; Lu, H.; Wang, D.; Da Silva, L. C.; Li, R.; Wang, L.; Landfester, K.; Zhang, K. a. I. Asymmetric Covalent Triazine Framework for Enhanced Visible-Light Photoredox Catalysis via Energy Transfer Cascade. *Angew. Chem., Int. Ed.* **2018**, *57*, 8316–8320.

(3) Rehm, D.; Weller, A. Kinetics of Fluorescence Quenching by Electron and H-Atom Transfer. *Isr. J. Chem*. **1970**, *8*, 259−271.

(4) Roy, S.; Sharma, B.; Pécaut, J.; Simon, P.; Fontecave, M.; Tran, P. D.; Derat, E.; Artero, V. Molecular Cobalt Complexes with Pendant Amines for Selective Electrocatalytic Reduction of Carbon Dioxide to Formic Acid. *J. Am. Chem. Soc*. **2017**, *139*, 3685–3696.

(5) Swinehart, D. F. The Beer-Lambert Law. *J. Chem. Educ*. **1962**, *39*, 333–335.

(6) Boaz, H.; Rollefson, G. K. The Quenching of Fluorescence. Deviations from the Stern-Volmer Law. *J. Am. Chem. Soc*. **1950**, *72*, 3435-3443.

(7) Unoh, Y.; Hirano, K.; Satoh, T.; Miura, M. An Approach to Benzophosphole Oxides through Silver- or Manganese-Mediated Dehydrogenative Annulation Involving C-C and C-P Bond Formation. *Angew. Chem., Int. Ed.* 2013, *52*, 12975–12979.

(8) Huang, W.; Byun, J.; Rörich, I., Ramanan, C.; Blom, P. W. M.; Lu, H.; Wang, D.; Da Silva, L. C.; Li, R.; Wang, L.; Landfester, K.; Zhang, K. a. I. Asymmetric Covalent Triazine Framework for Enhanced Visible-Light Photoredox catalysis via Energy Transfer Cascade. *Angew. Chem., Int. Ed.* **2018**, *57*, 8316–8320.

(9) Ma, D.; Chen, W.; Hu, G.; Zhang, Y.; Gao, Y.; Yin, Y.; Zhao, Y. $K_2S_2O_8$-mediated metal-free direct P–H/C–H functionalization: a convenient route to benzo[*b*]phosphole oxides from unactivated alkynes. *Green Chem*. **2016**, *18*, 3522–3526.

(10) Quint, V.; Morlet-Savary, F.; Lohier, J.; Lalevée, J.; Gaumont, A.; Lakhdar, S. Metal-Free, Visible Light-Photocatalyzed Synthesis of Benzo[*b*]phosphole Oxides: Synthetic and Mechanistic Investigations. *J. Am. Chem. Soc.* **2016**, *138*, 7436–7441.

(11) Budnikova, Y. H. Transition metal-promoted reactions of diarylphosphine oxides as a synthetic method for organophosphorus heterocyclic compounds. *Chemistry of Heterocyclic Compounds*, **2018**, *54*, 269–279.

23. ^{1}H, and ^{13}C NMR Spectra of PC_1:

^{1}H NMR (400 MHz, $CDCl_3$)

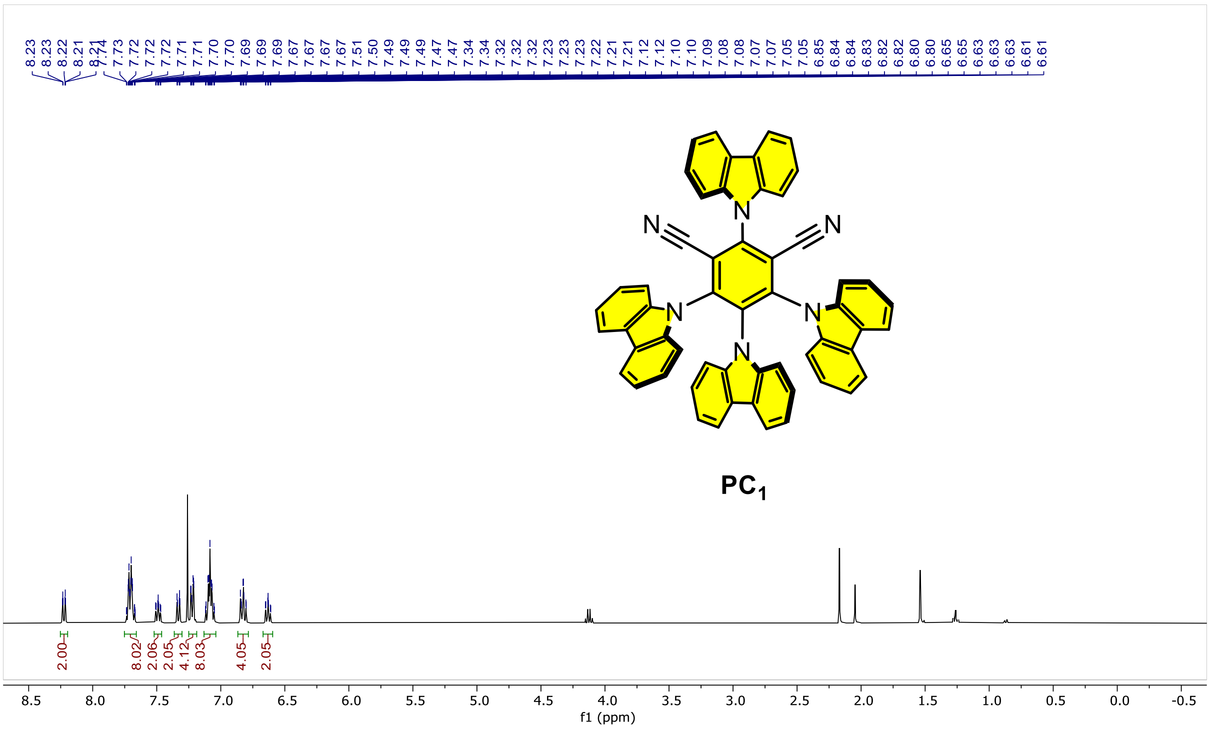

^{13}C NMR (101 MHz, $CDCl_3$)

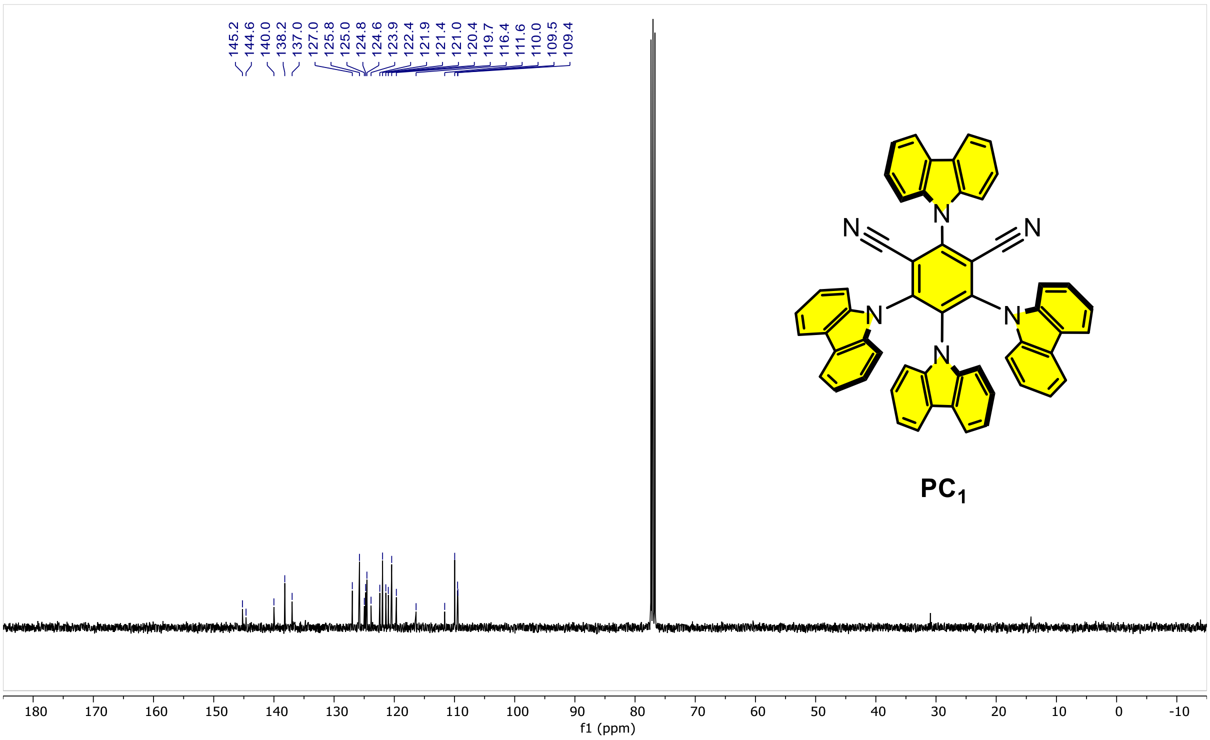

24. ^{1}H, ^{13}C, ^{31}P, and ^{19}F NMR Spectra of the Products:

^{1}H NMR (400 MHz, $CDCl_3$) of compound **3a**

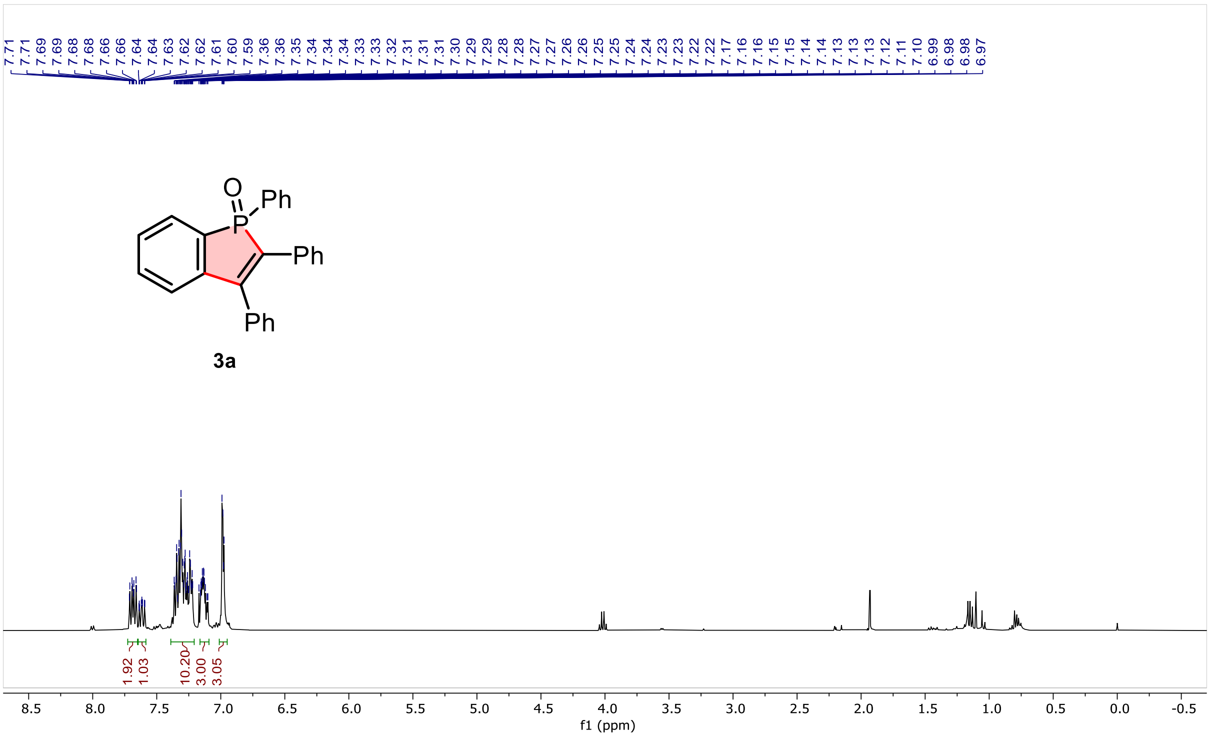

^{13}C NMR (101 MHz, $CDCl_3$) of compound **3a**

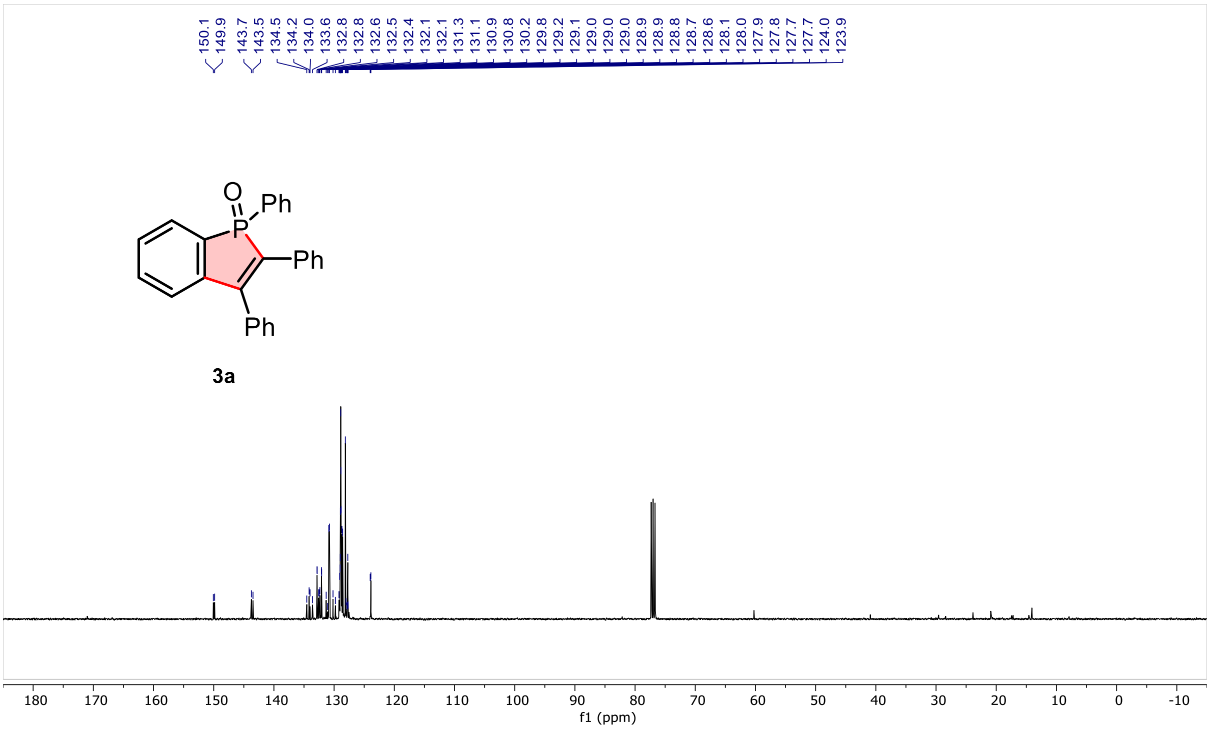

^{31}P NMR (162 MHz, $CDCl_3$) of compound **3a**

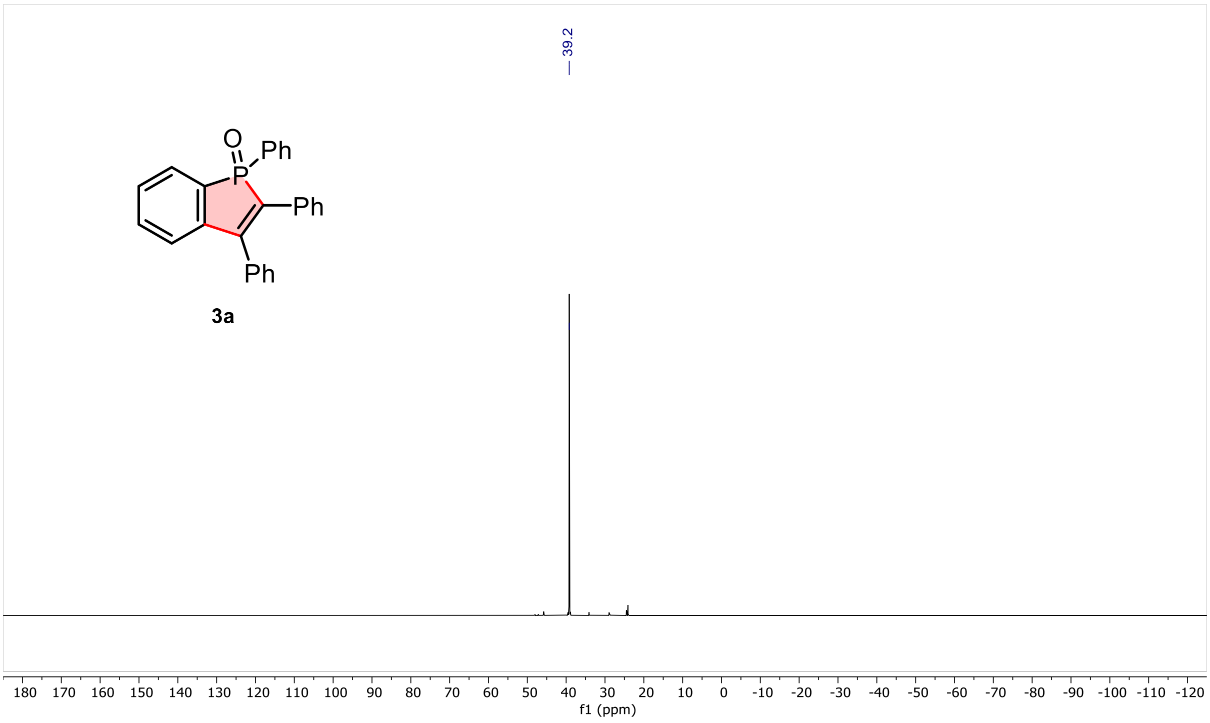

^{1}H NMR (400 MHz, $CDCl_3$) of compound **3b, 3b'**

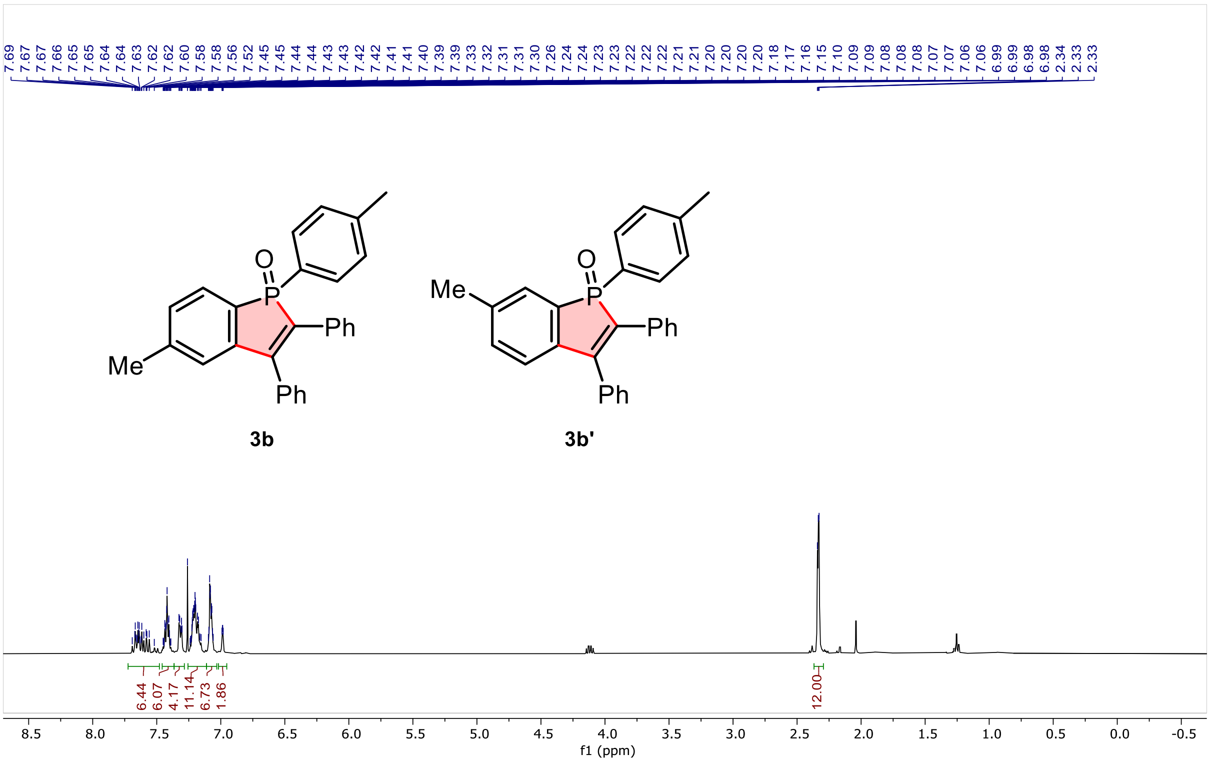

^{13}C NMR (101 MHz, $CDCl_3$) of compound **3b, 3b'**

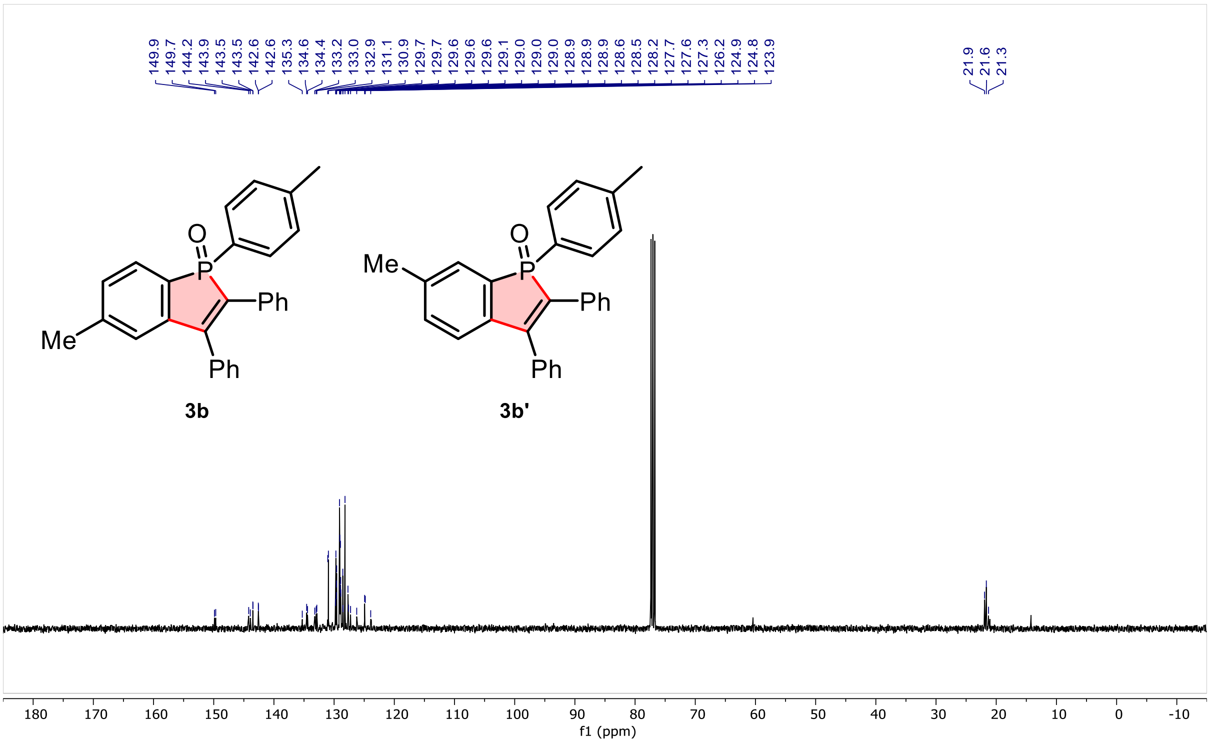

^{31}P NMR (162 MHz, $CDCl_3$) of compound **3b, 3b'**

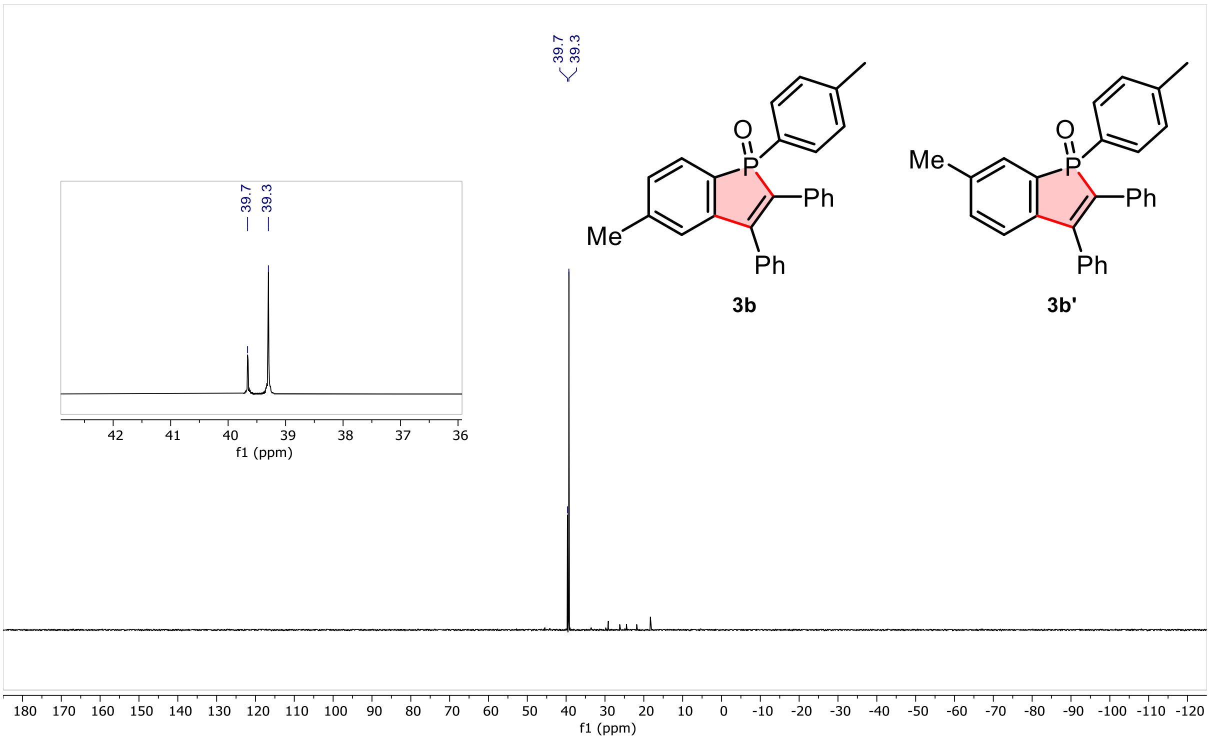

^{1}H NMR (400 MHz, $CDCl_3$) of compound **3c, 3c’**

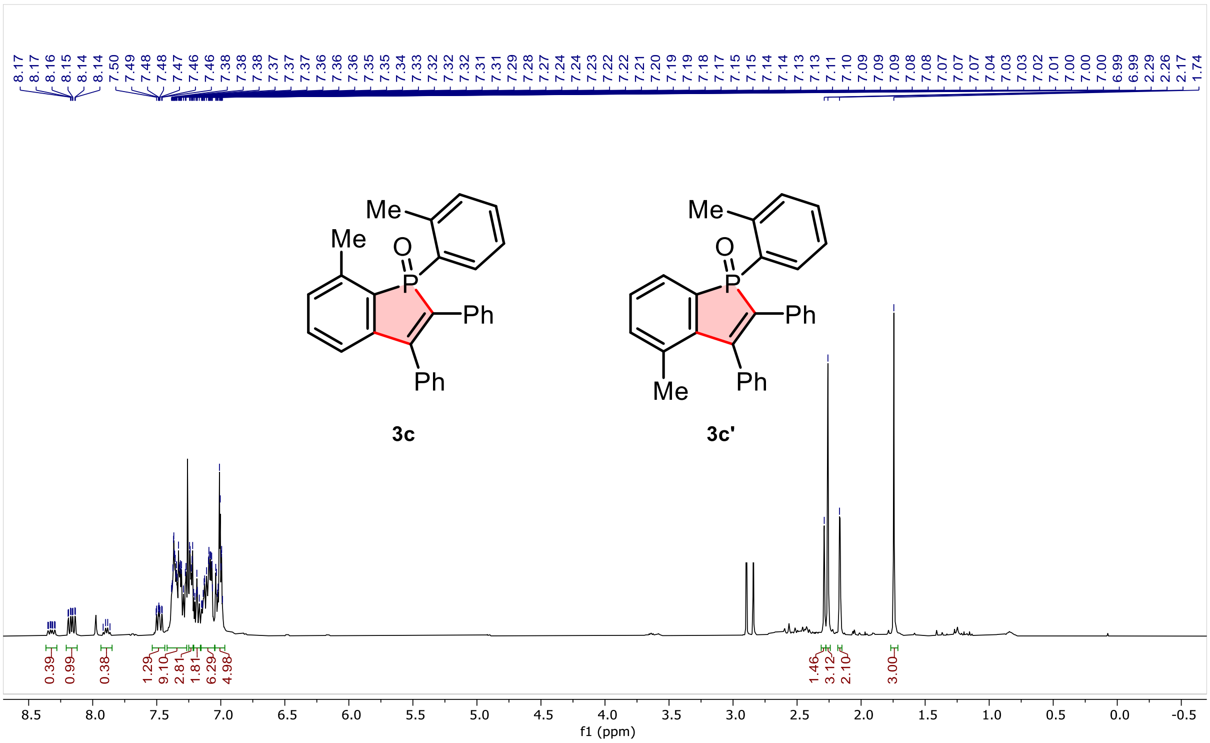

^{13}C NMR (101 MHz, $CDCl_3$) of compound **3c, 3c’**

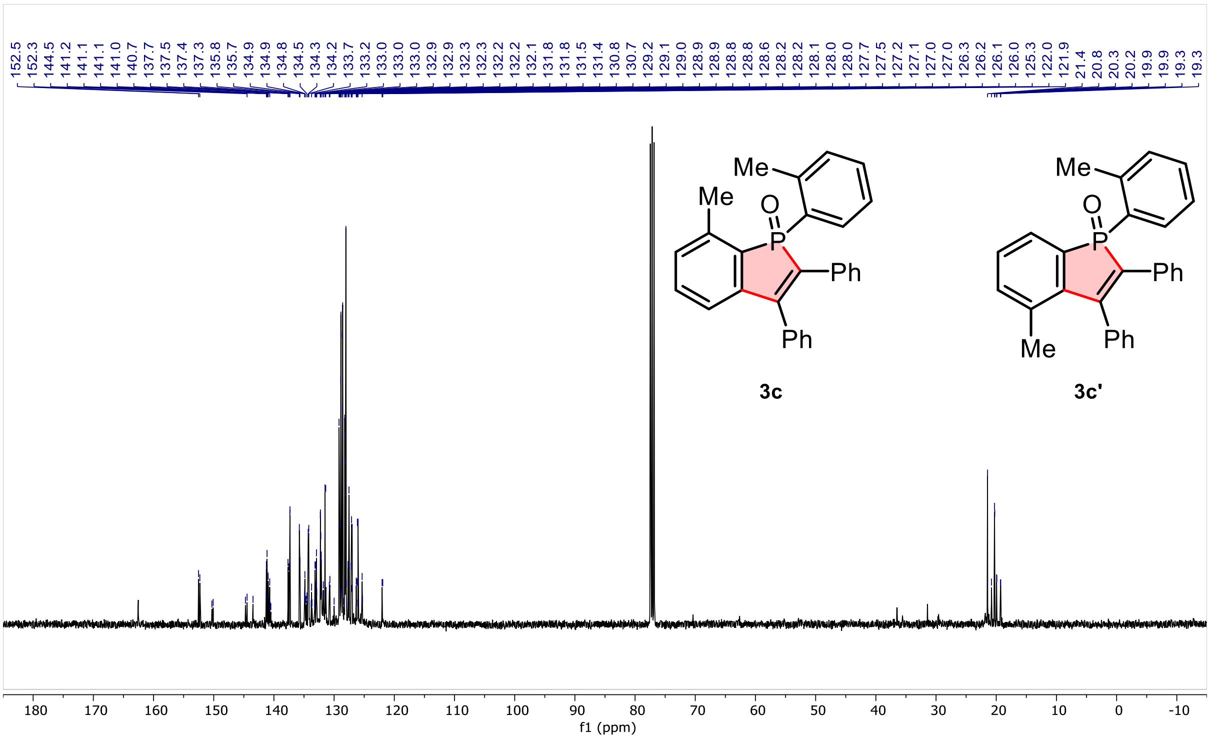

^{31}P NMR (162 MHz, $CDCl_3$) of compound **3c, 3c'**

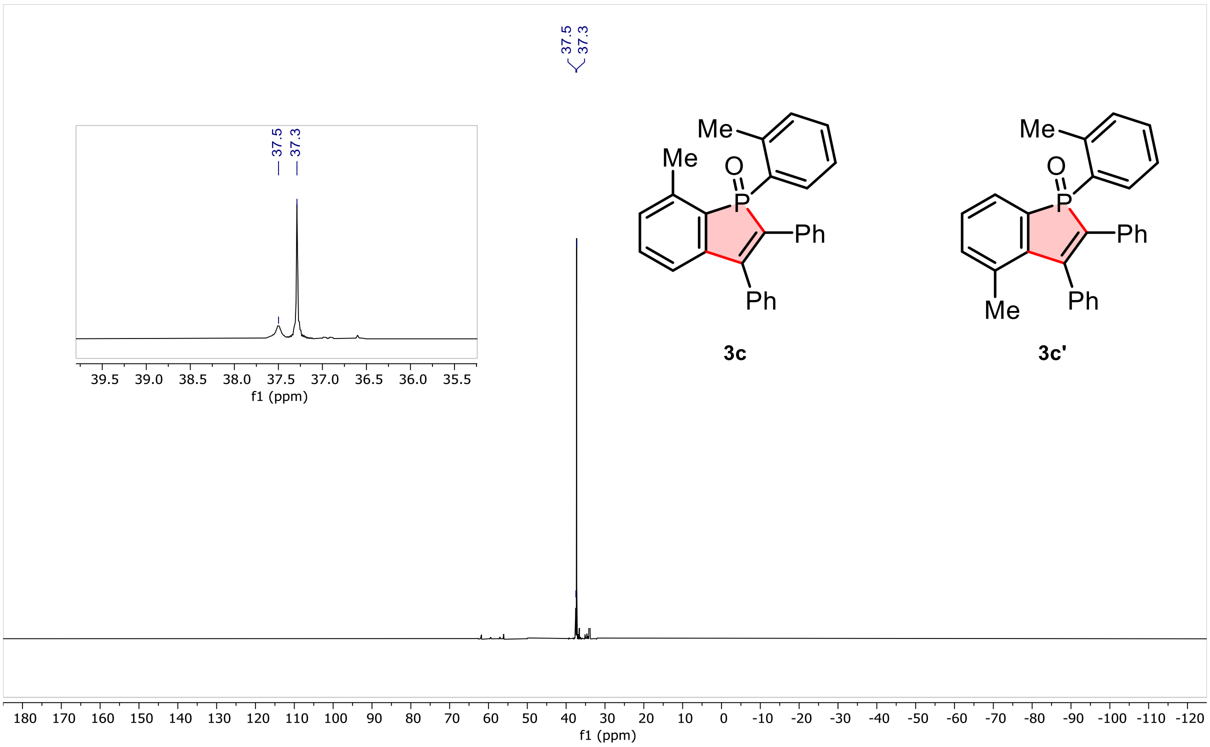

^{1}H NMR (400 MHz, $CDCl_3$) of compound **3d**

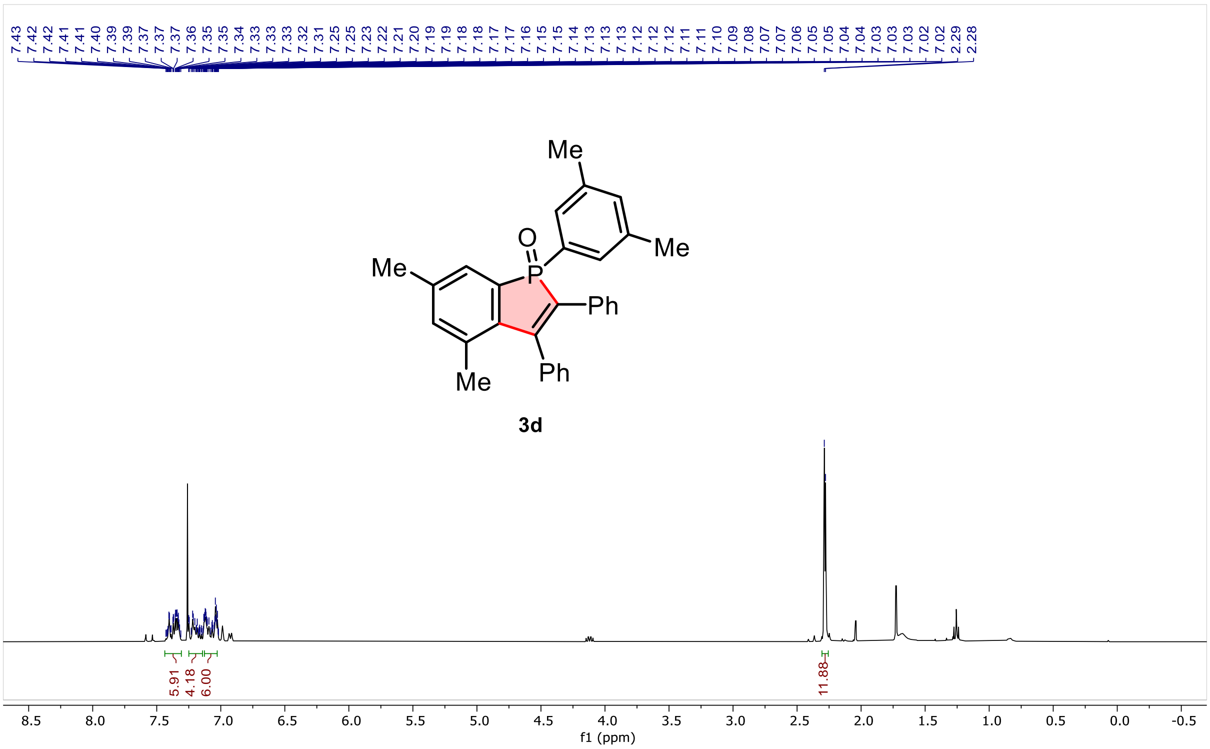

^{13}C NMR (101 MHz, $CDCl_3$) of compound **3d**

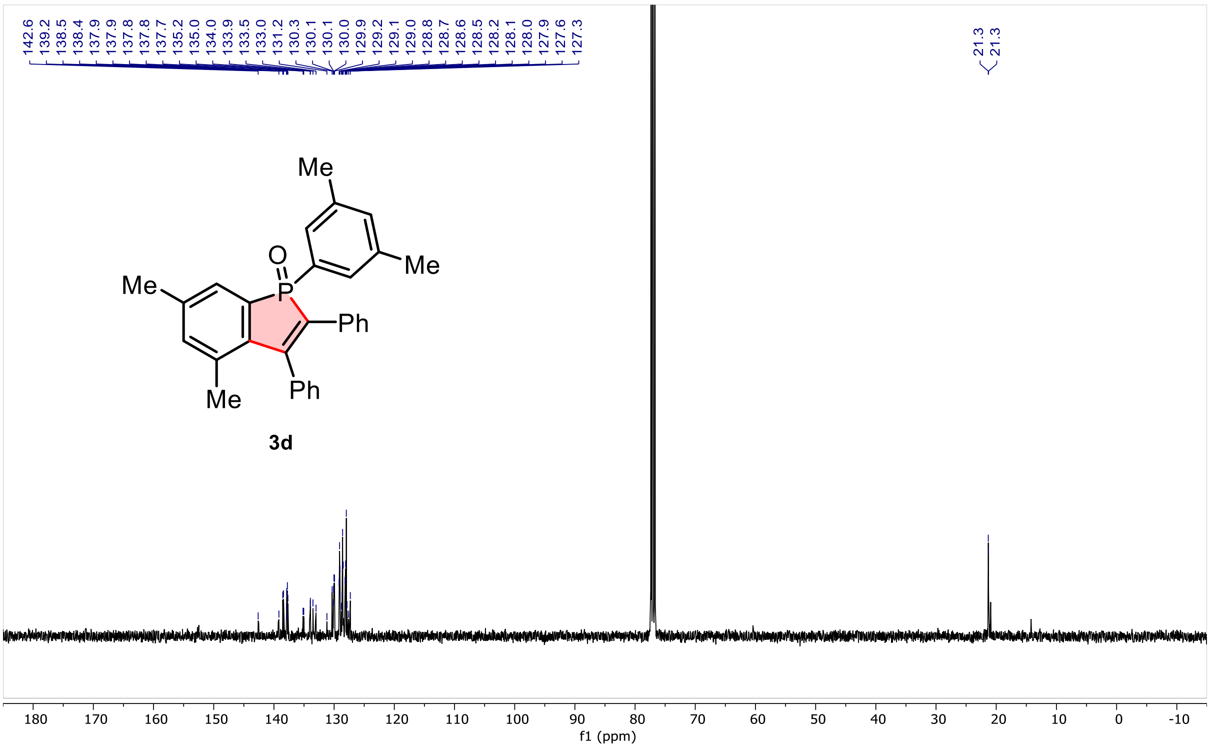

^{31}P NMR (162 MHz, $CDCl_3$) of compound **3d**

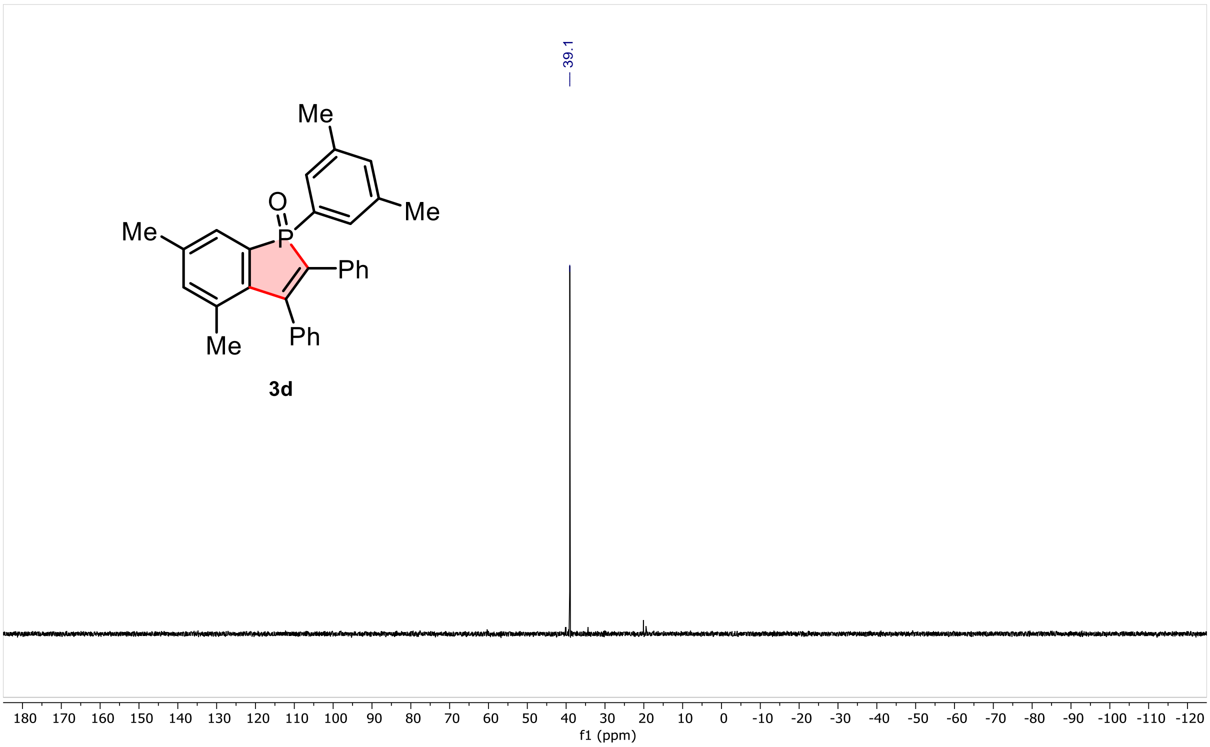

^{1}H NMR (400 MHz, $CDCl_3$) of compound **3e, 3e'**

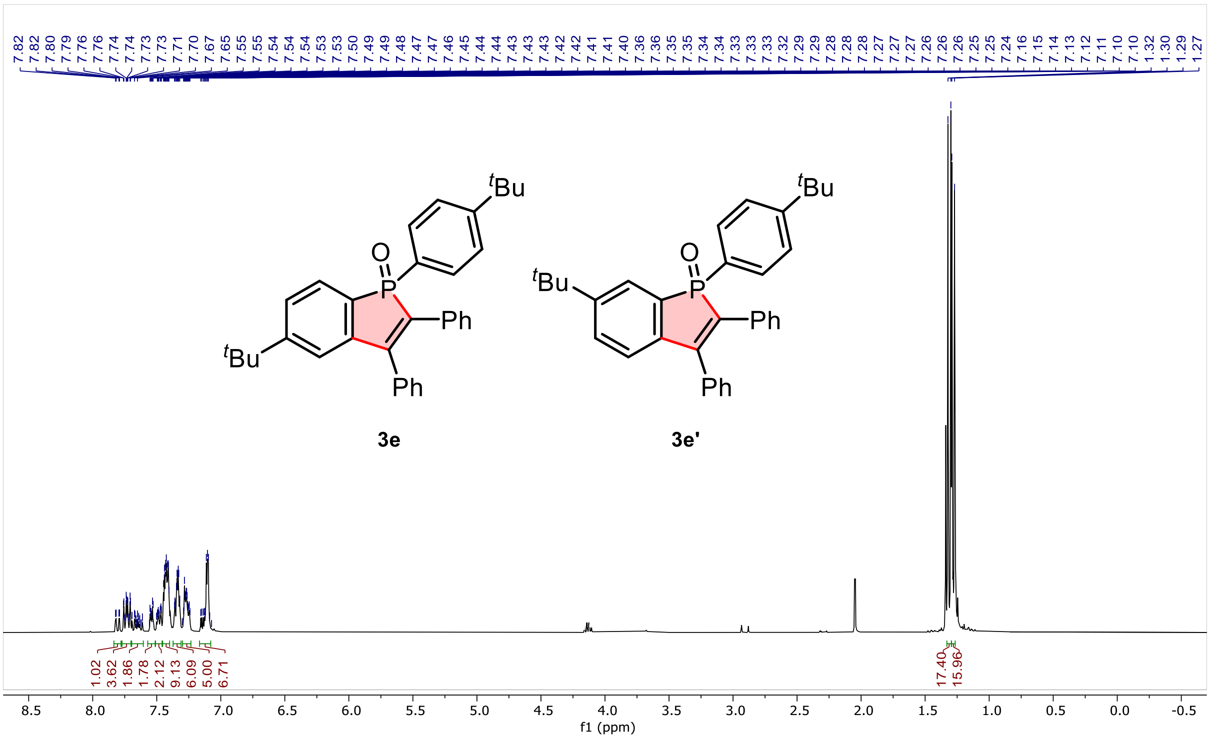

^{13}C NMR (101 MHz, $CDCl_3$) of compound **3e, 3e'**

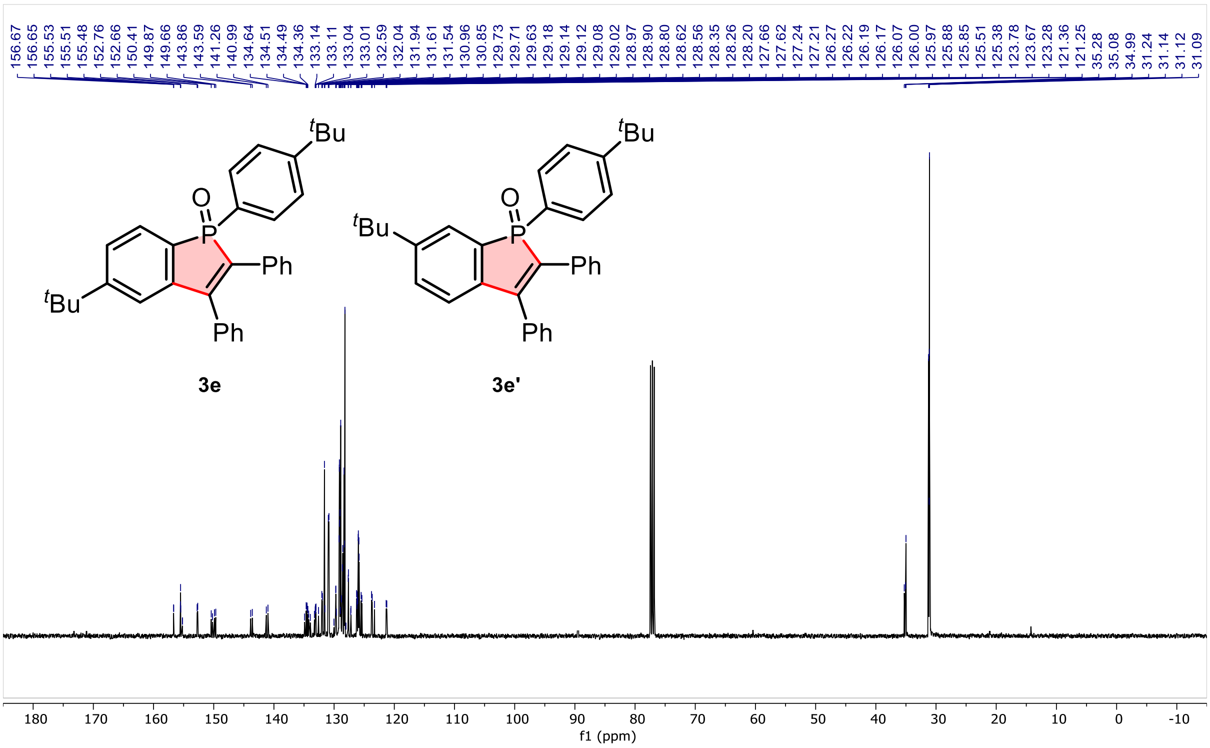

^{31}P NMR (162 MHz, $CDCl_3$) of compound **3e, 3e'**

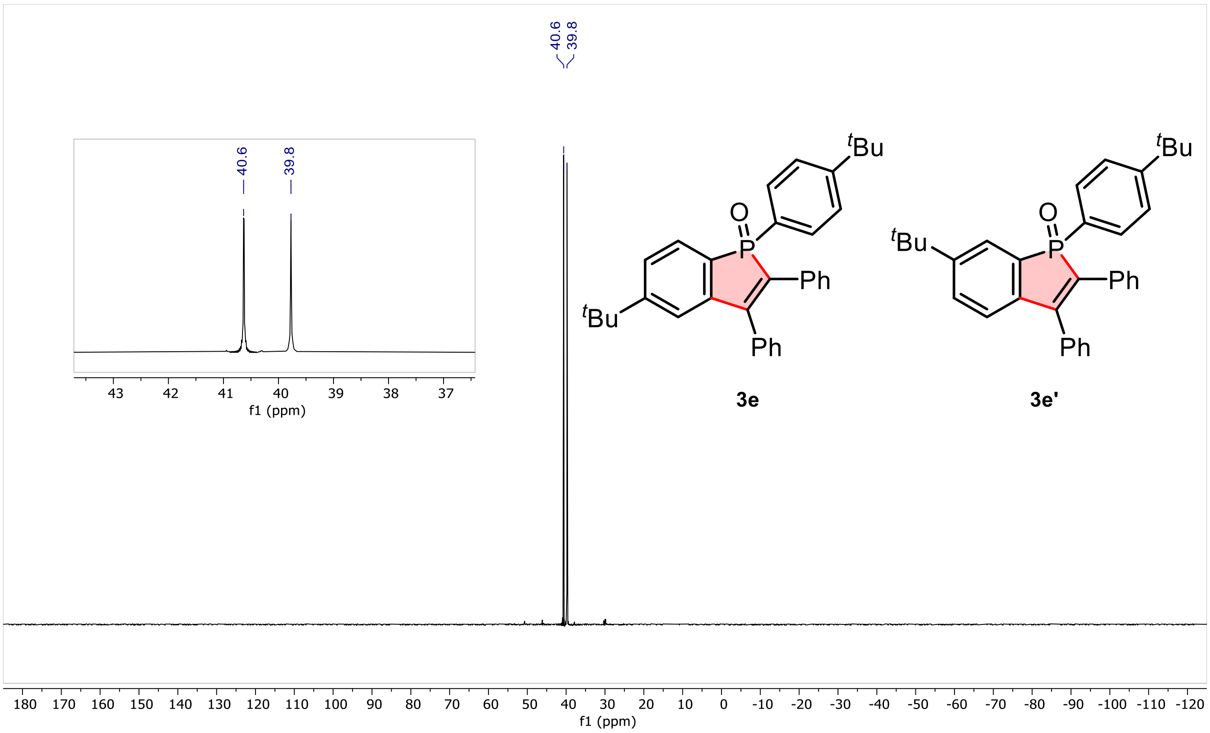

^{1}H NMR (400 MHz, CDCl$_3$) of compound **3f**

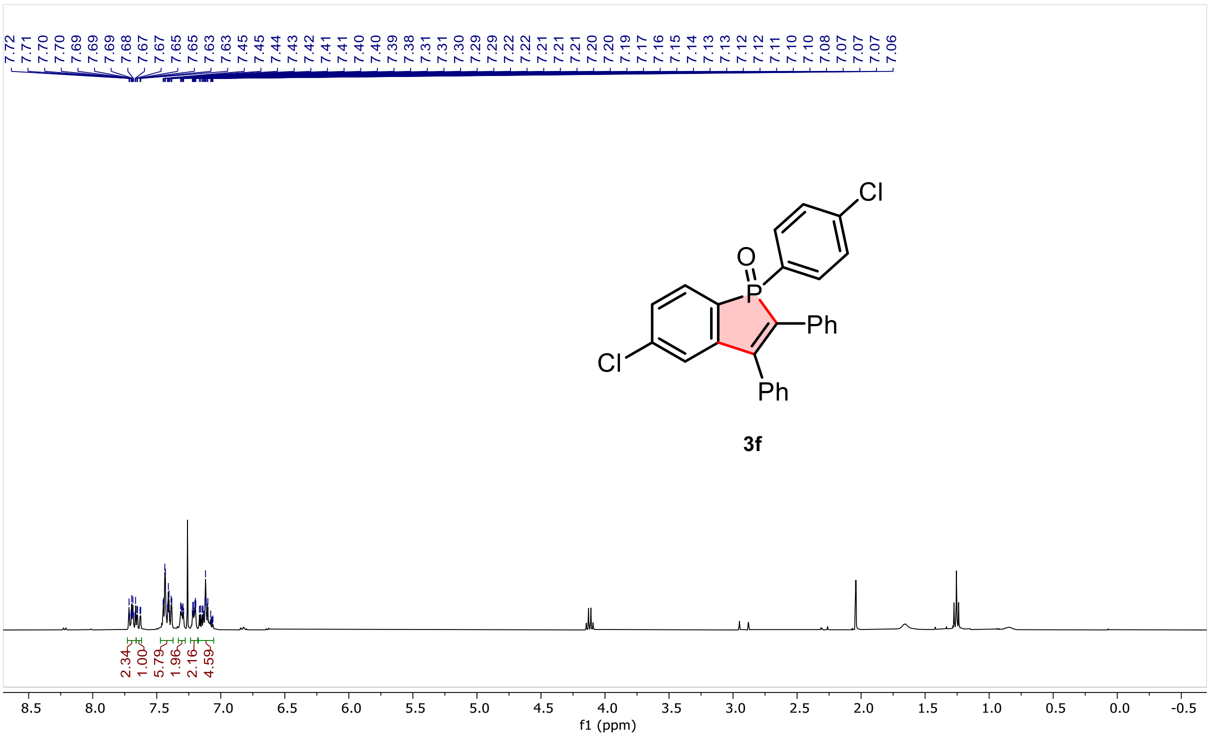

^{13}C NMR (101 MHz, CDCl$_3$) of compound **3f**

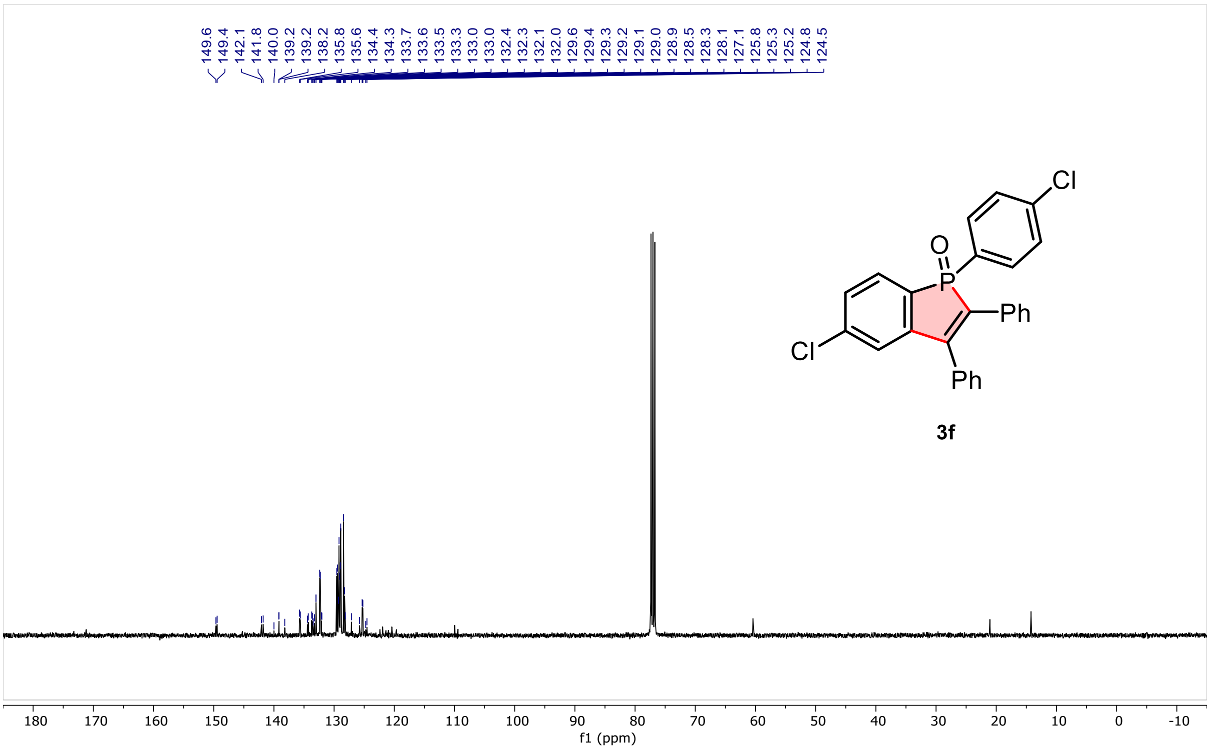

^{31}P NMR (162 MHz, $CDCl_3$) of compound **3f**

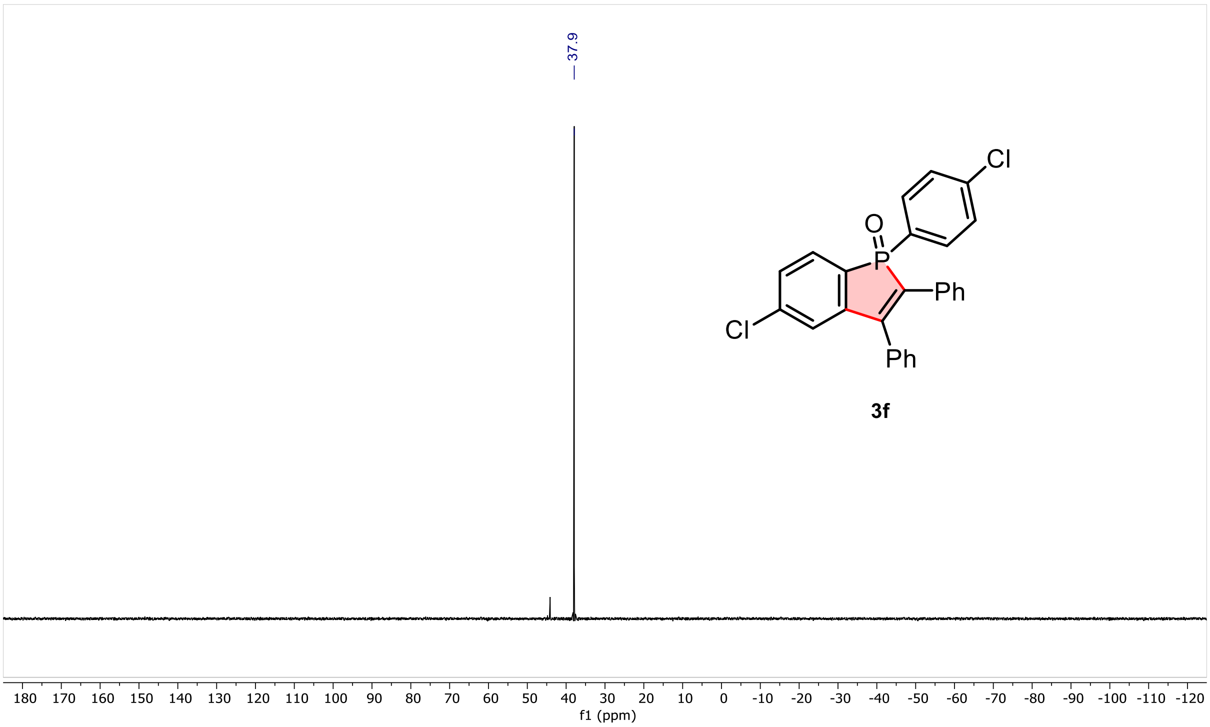

^{1}H NMR (400 MHz, $CDCl_3$) of compound **3f’**

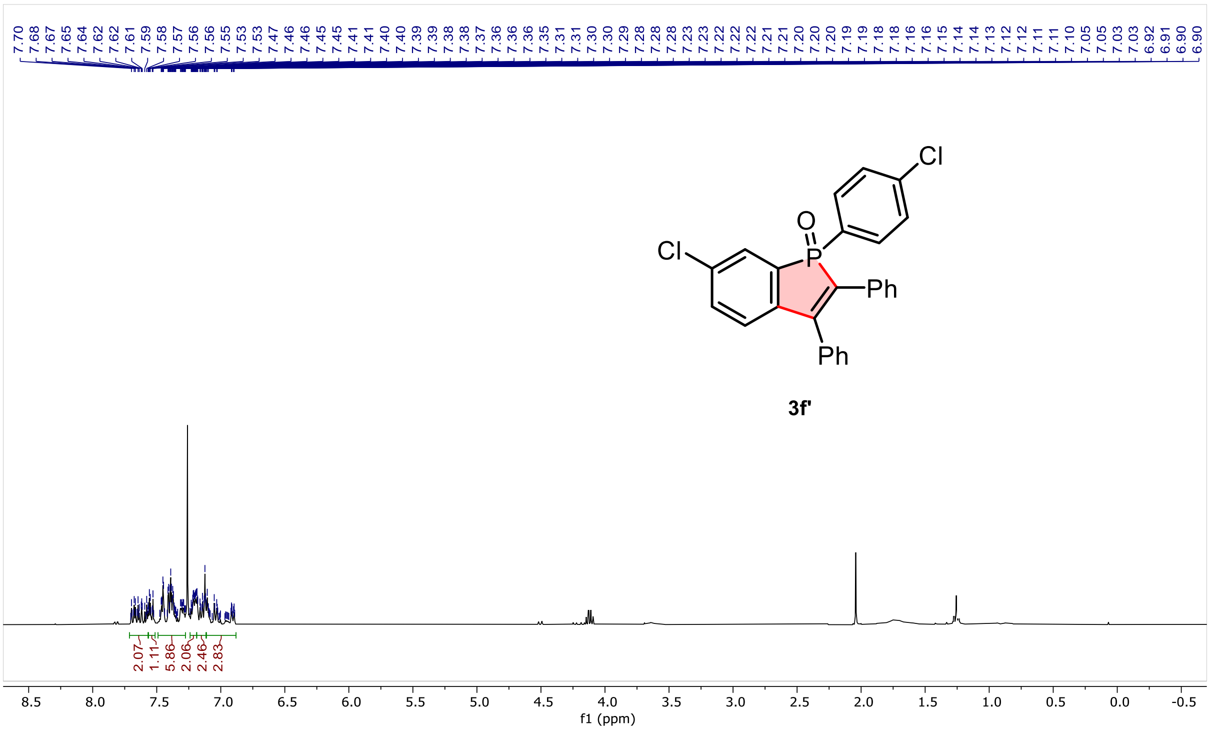

^{13}C NMR (101 MHz, $CDCl_3$) of compound **3f’**

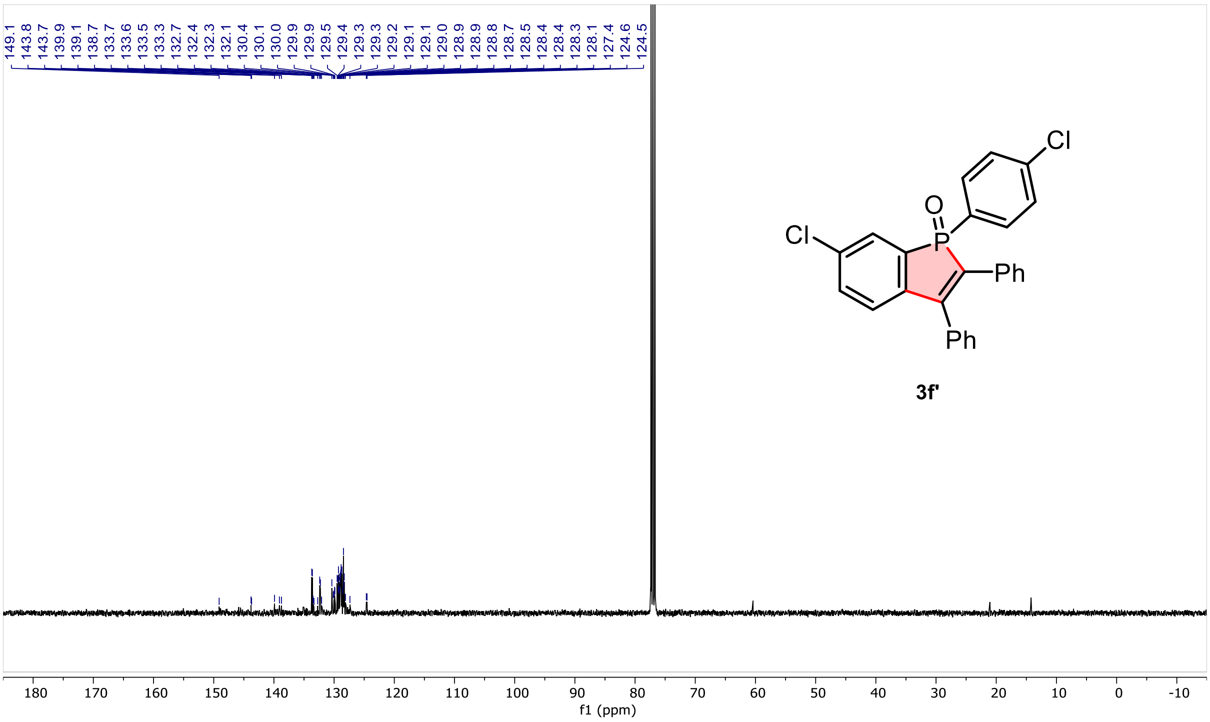

^{31}P NMR (162 MHz, $CDCl_3$) of compound **3f’**

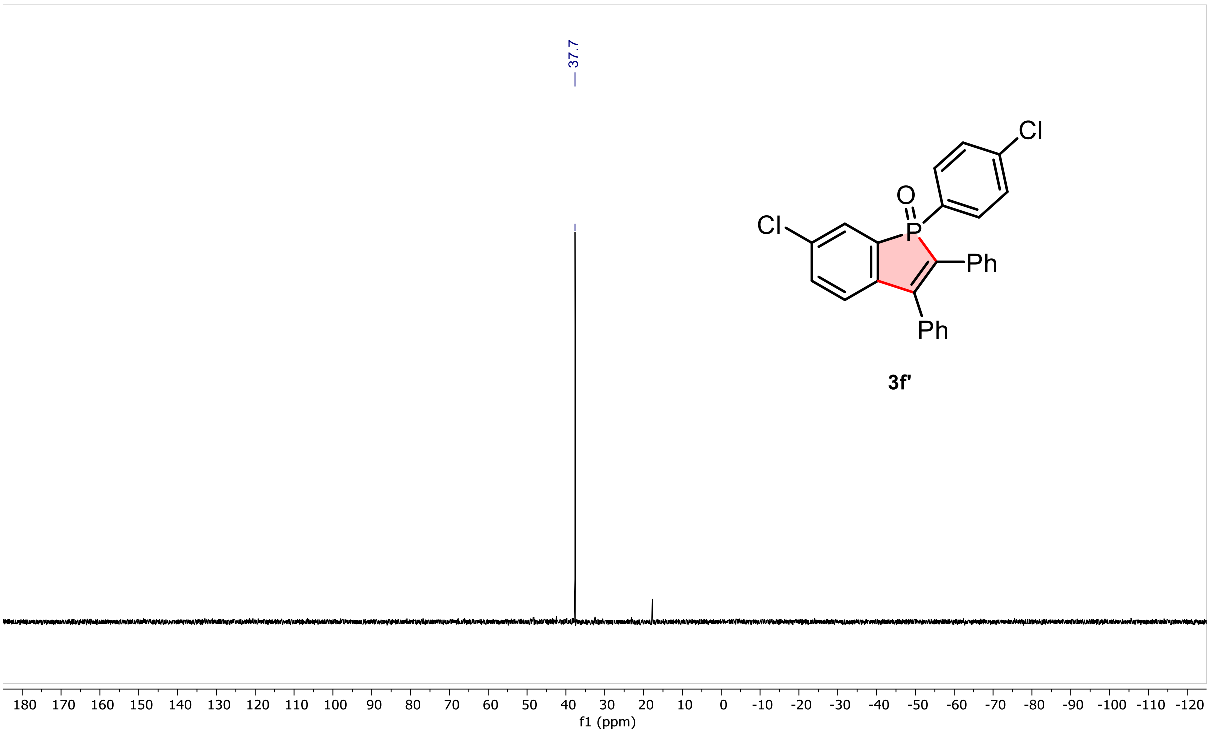

^{1}H NMR (400 MHz, $CDCl_3$) of compound **3g, 3g'**

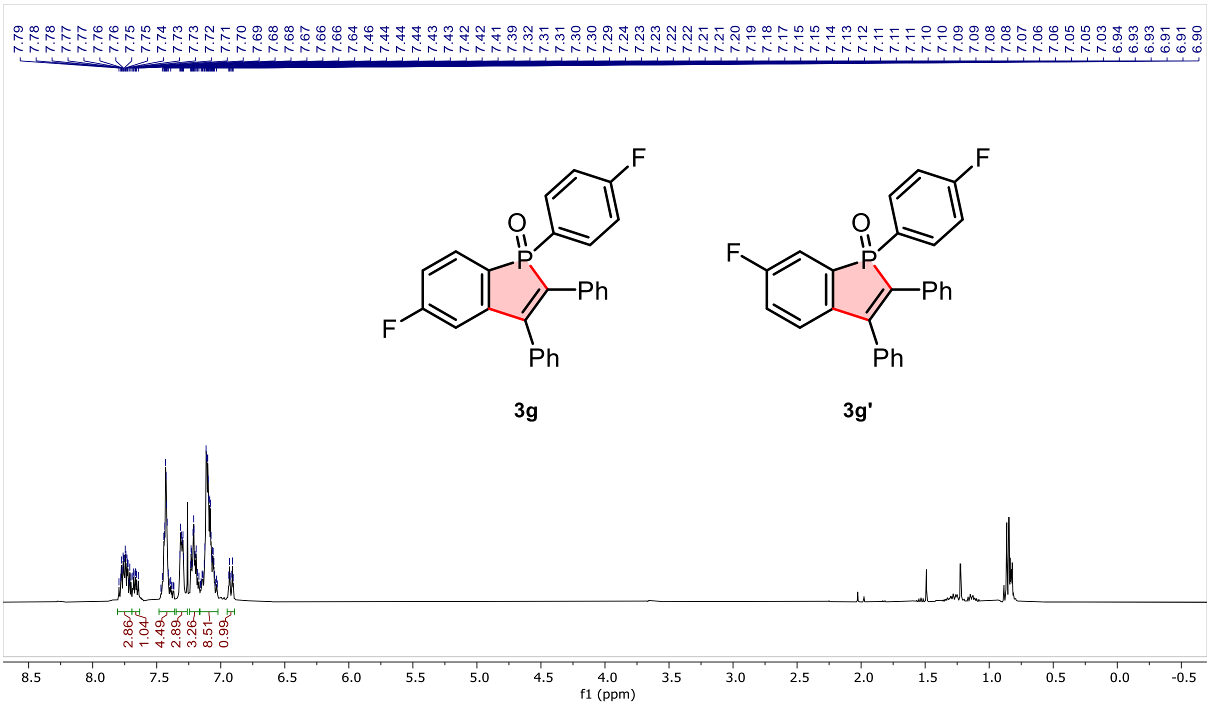

^{13}C NMR (101 MHz, $CDCl_3$) of compound **3g, 3g'**

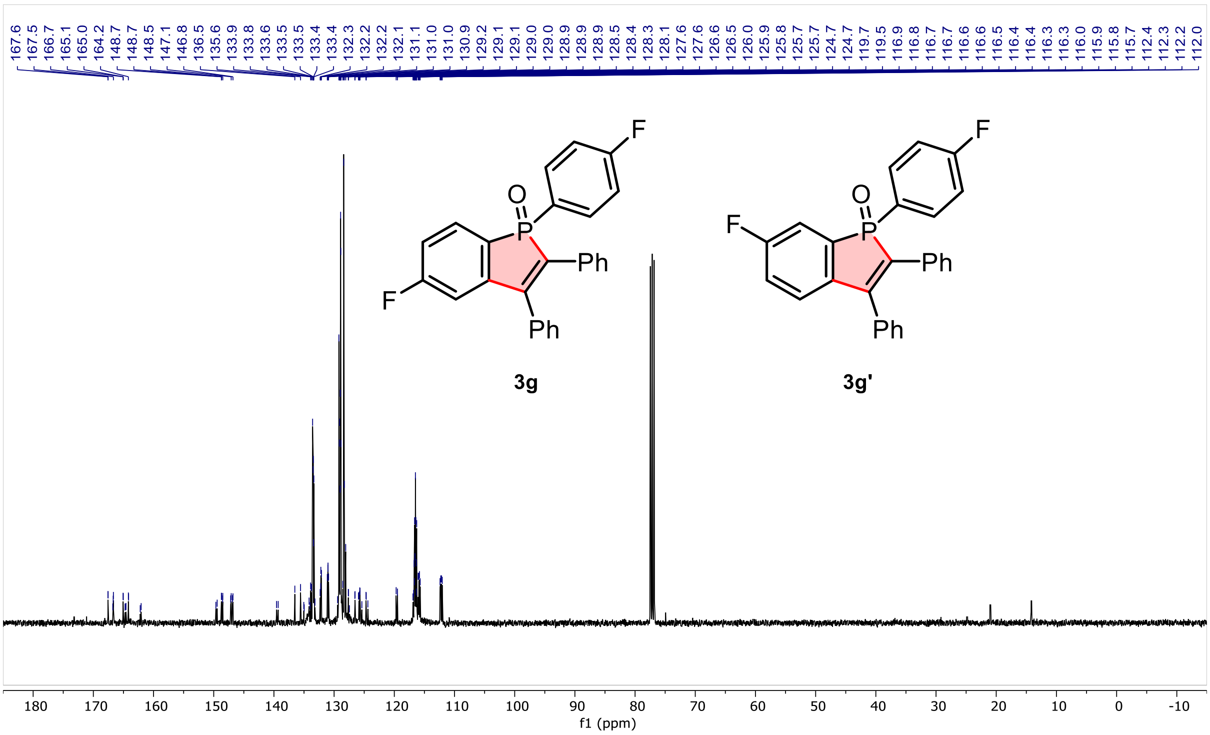

^{31}P NMR (162 MHz, $CDCl_3$) of compound **3g, 3g'**

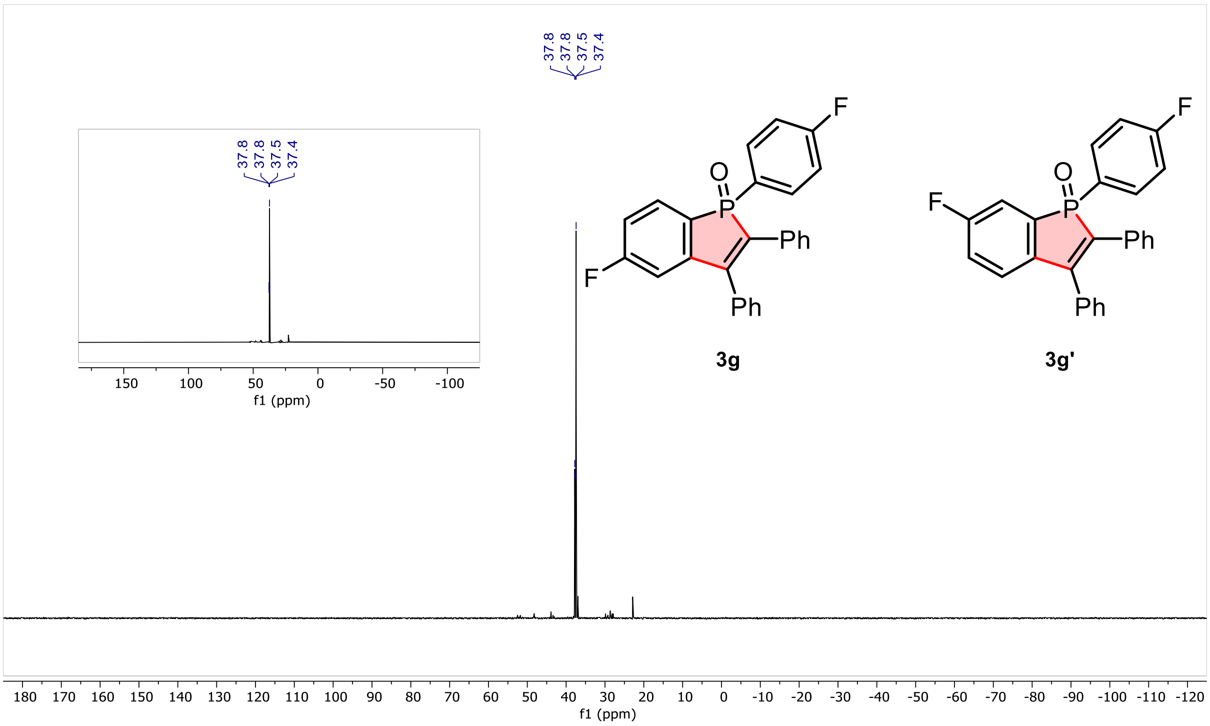

^{19}F NMR (376 MHz, $CDCl_3$) of compound **3g, 3g'**

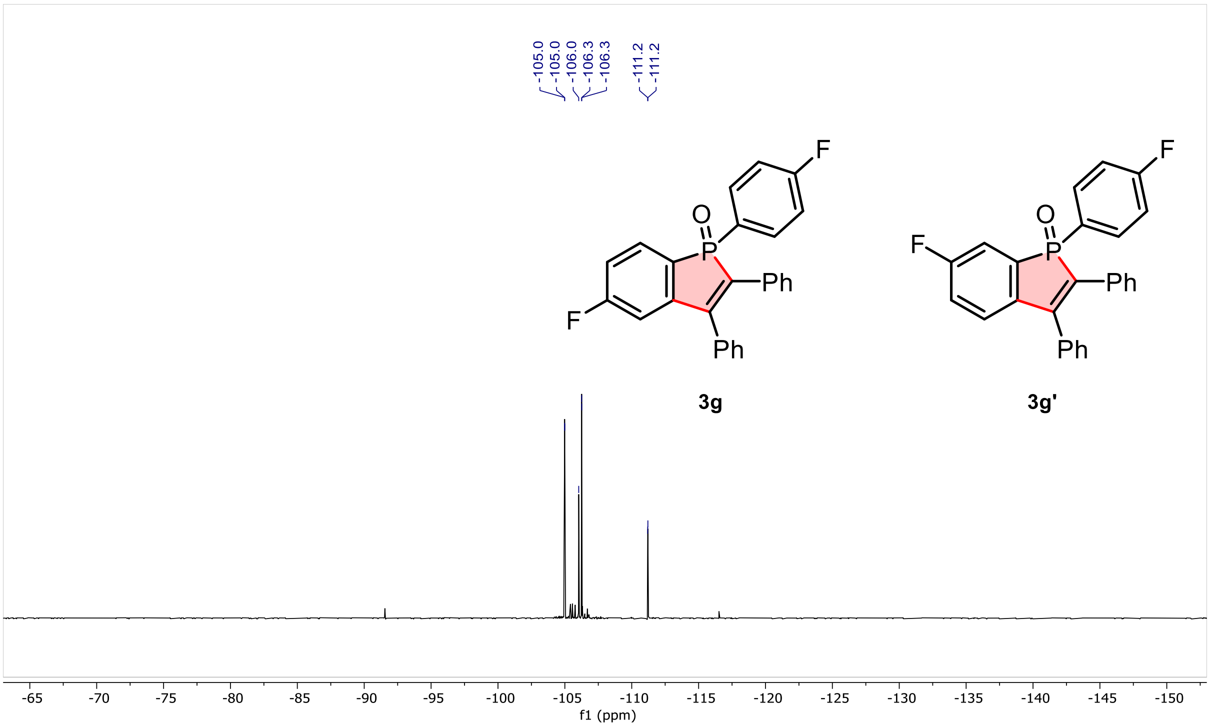

^{1}H NMR (400 MHz, $CDCl_3$) of compound **3h, 3h'**

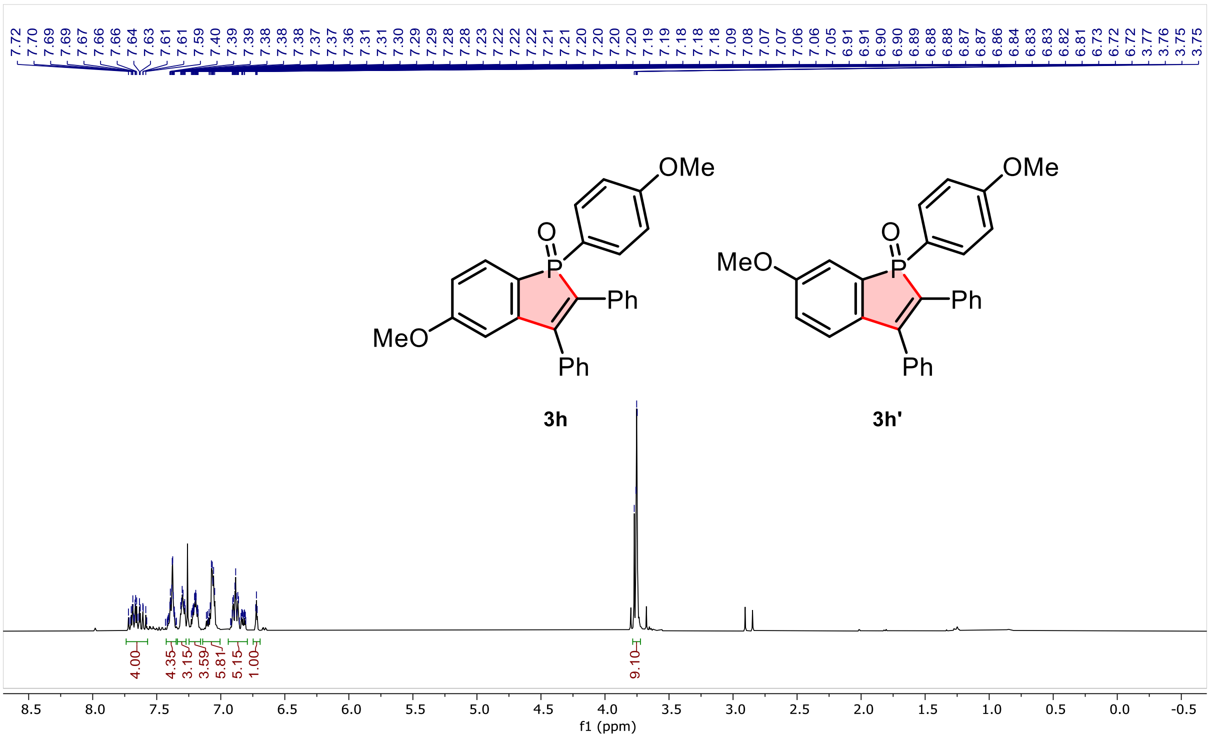

^{13}C NMR (101 MHz, $CDCl_3$) of compound **3h, 3h'**

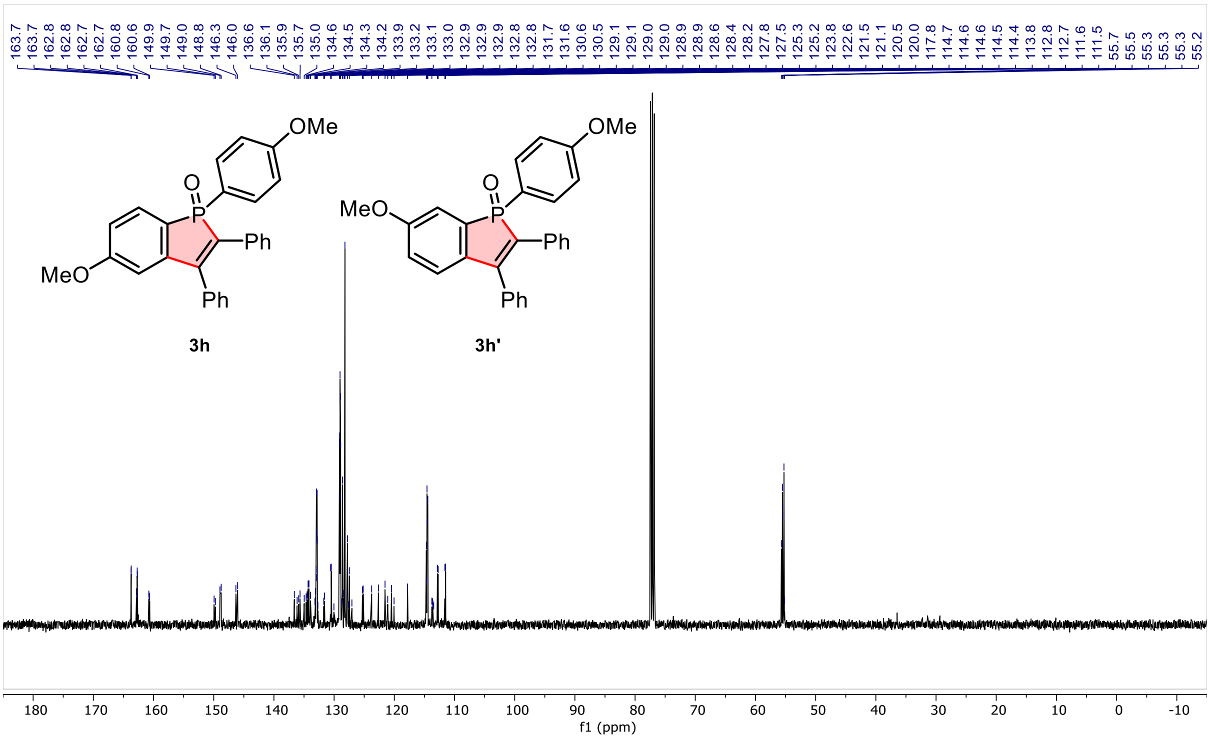

^{31}P NMR (162 MHz, $CDCl_3$) of compound **3h, 3h'**

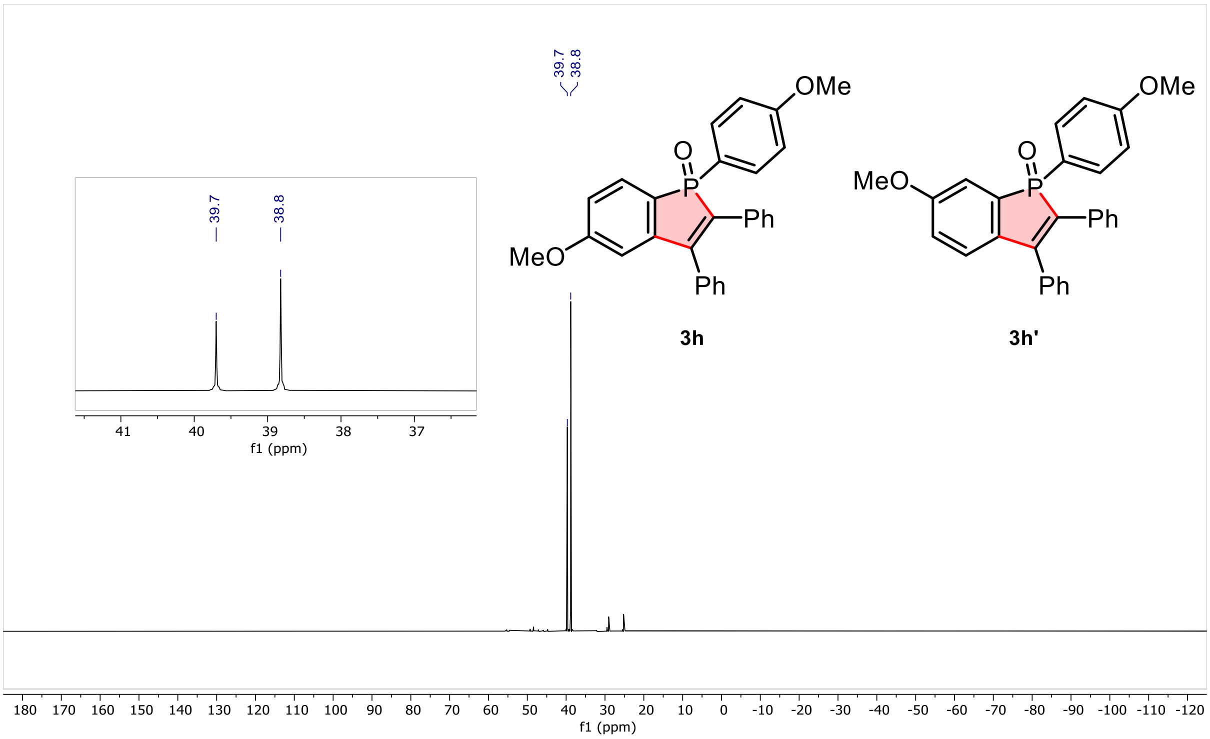

^{1}H NMR (400 MHz, $CDCl_3$) of compound **3i, 3i'**

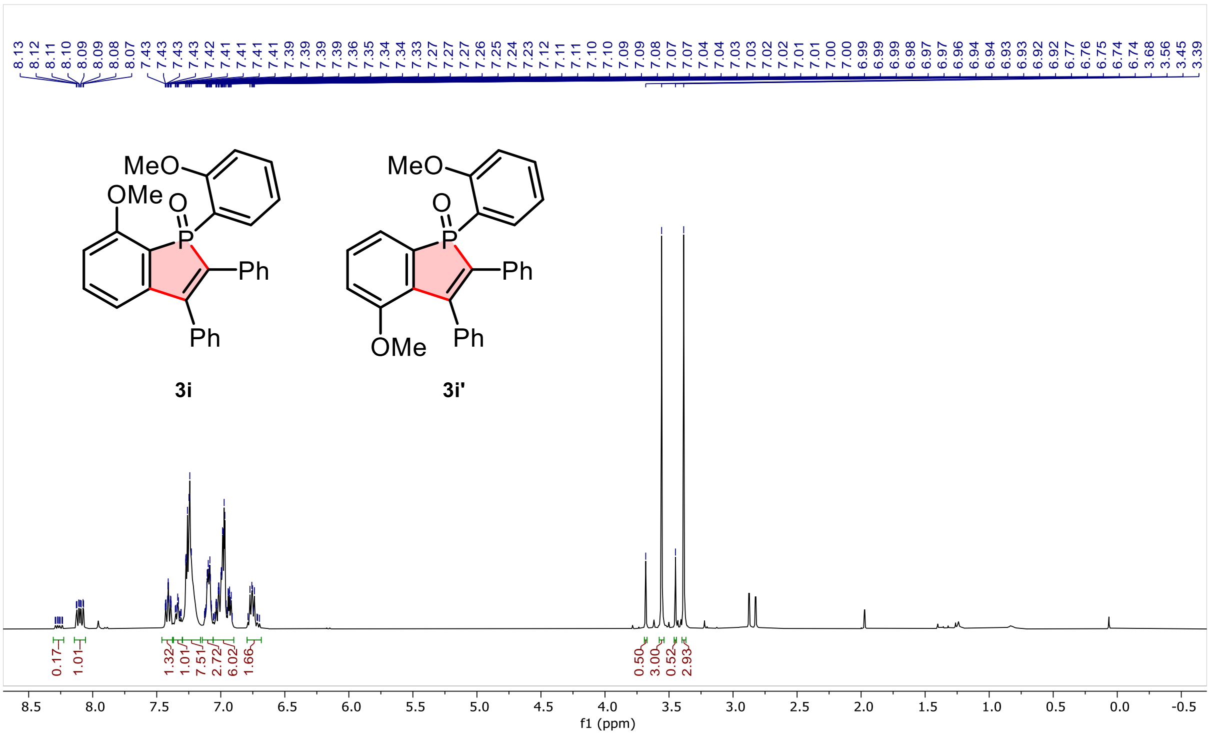

^{13}C NMR (101 MHz, $CDCl_3$) of compound **3i, 3i'**

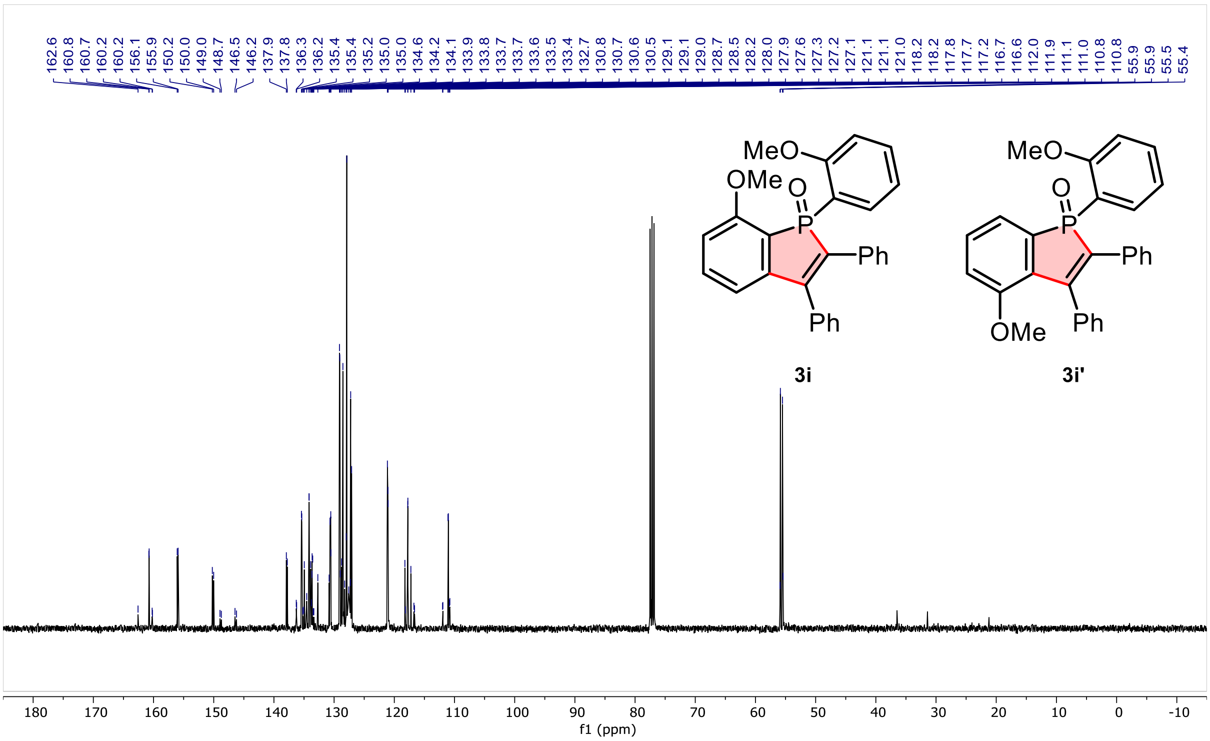

^{31}P NMR (162 MHz, $CDCl_3$) of compound **3i, 3i'**

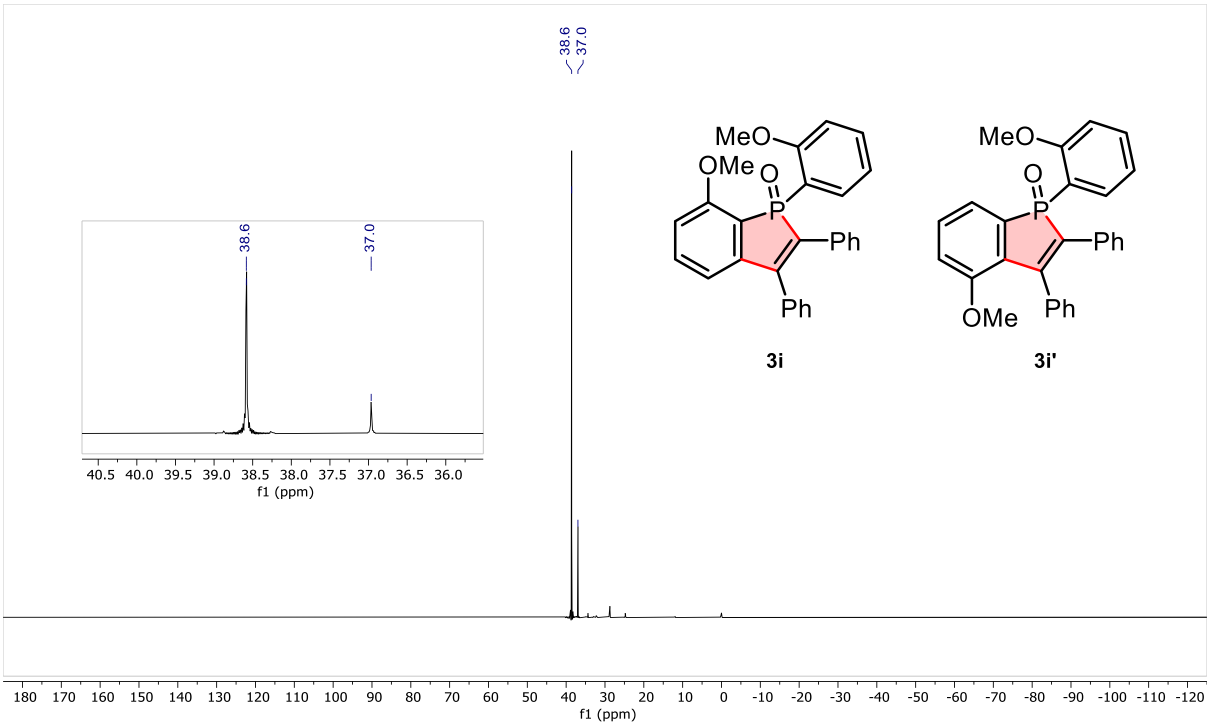

^{1}H NMR (400 MHz, $CDCl_3$) of compound **3j, 3j'**

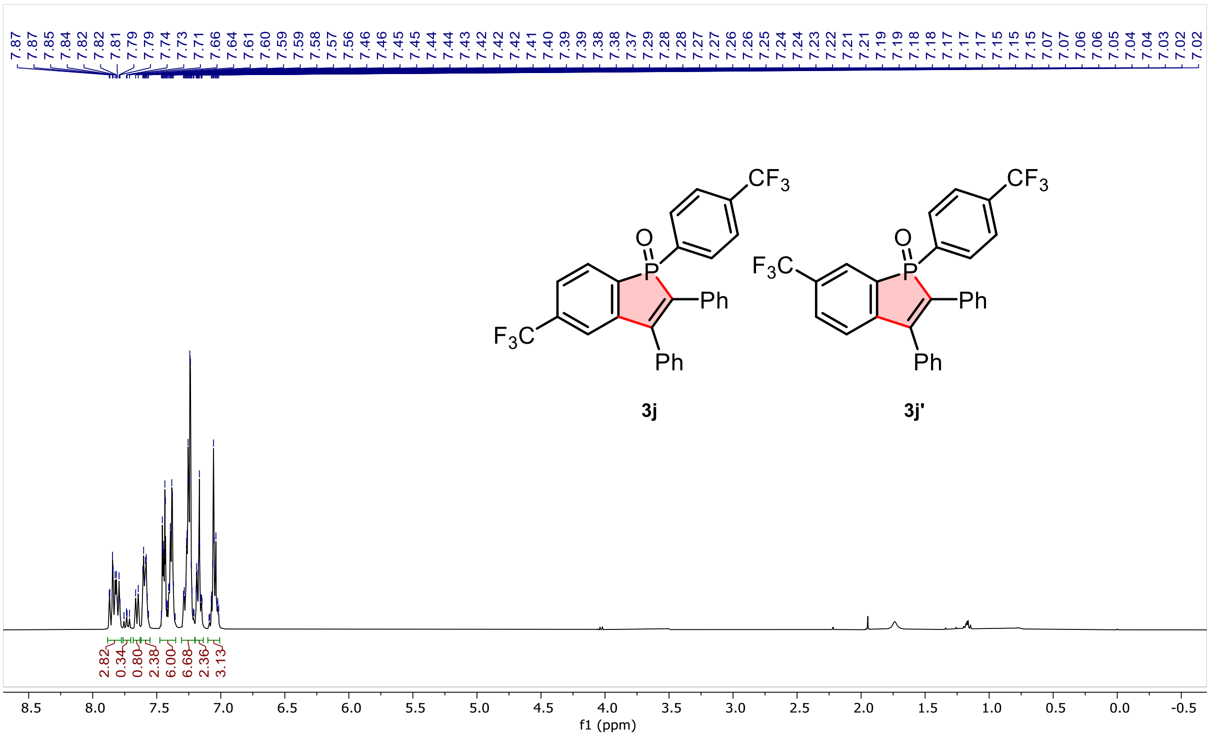

^{13}C NMR (101 MHz, $CDCl_3$) of compound **3j, 3j'**

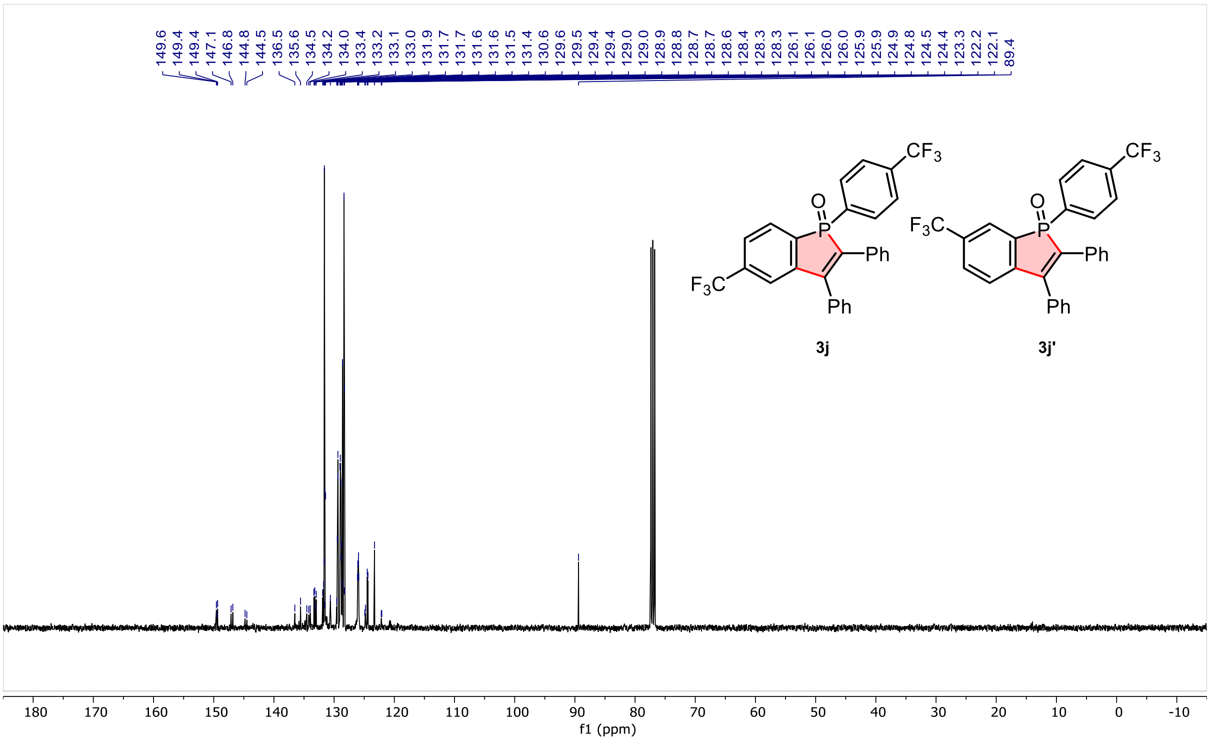

^{31}P NMR (162 MHz, $CDCl_3$) of compound **3j, 3j'**

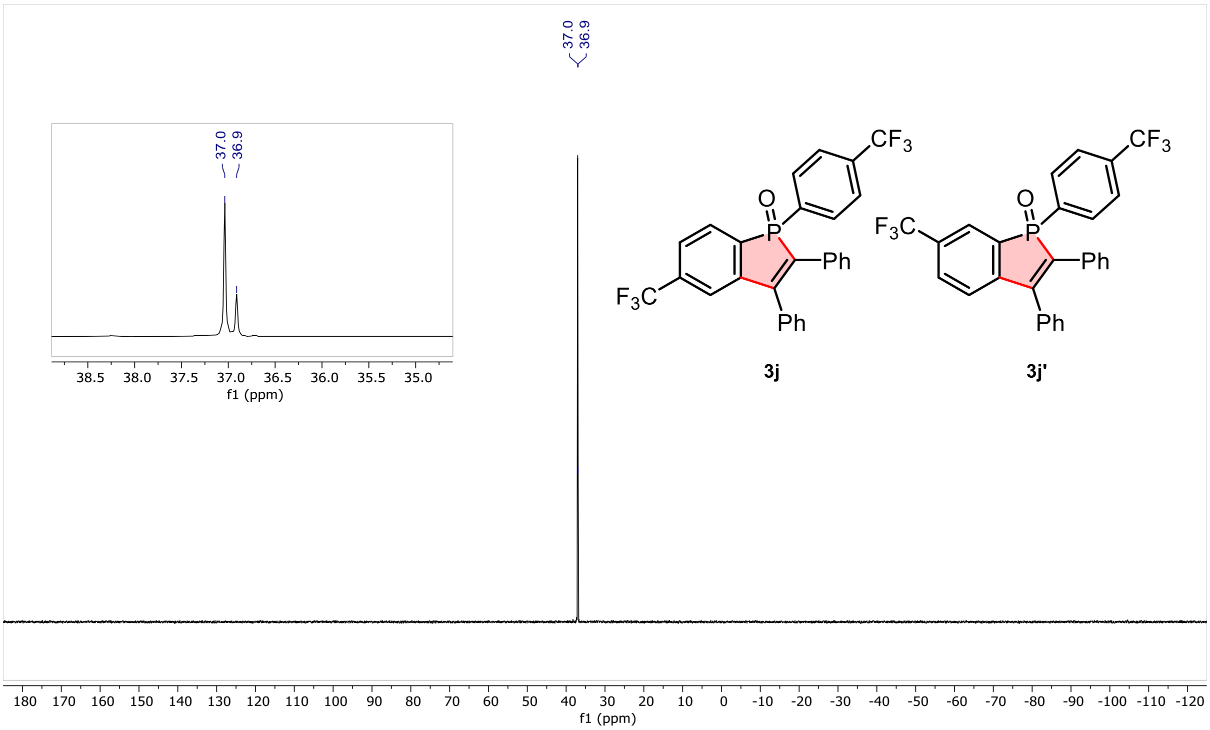

^{19}F NMR (376 MHz, $CDCl_3$) of compound **3j, 3j'**

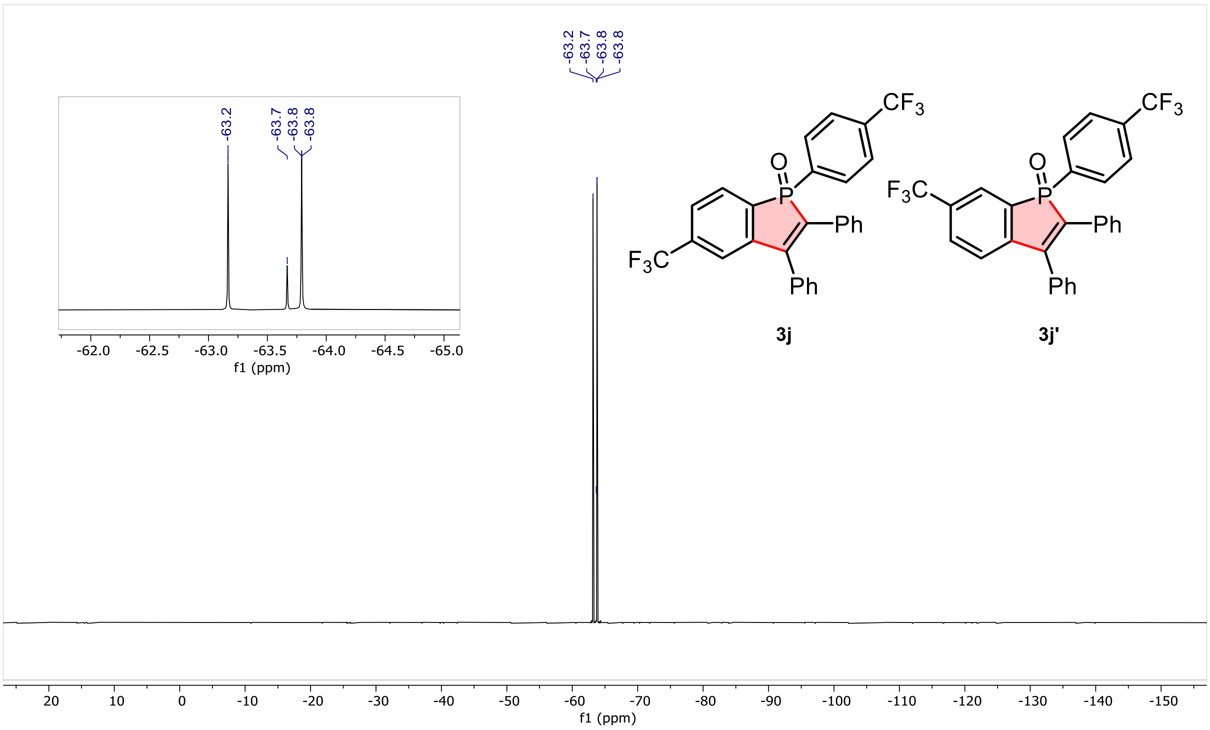

^{1}H NMR (400 MHz, $CDCl_3$) of compound **3k**

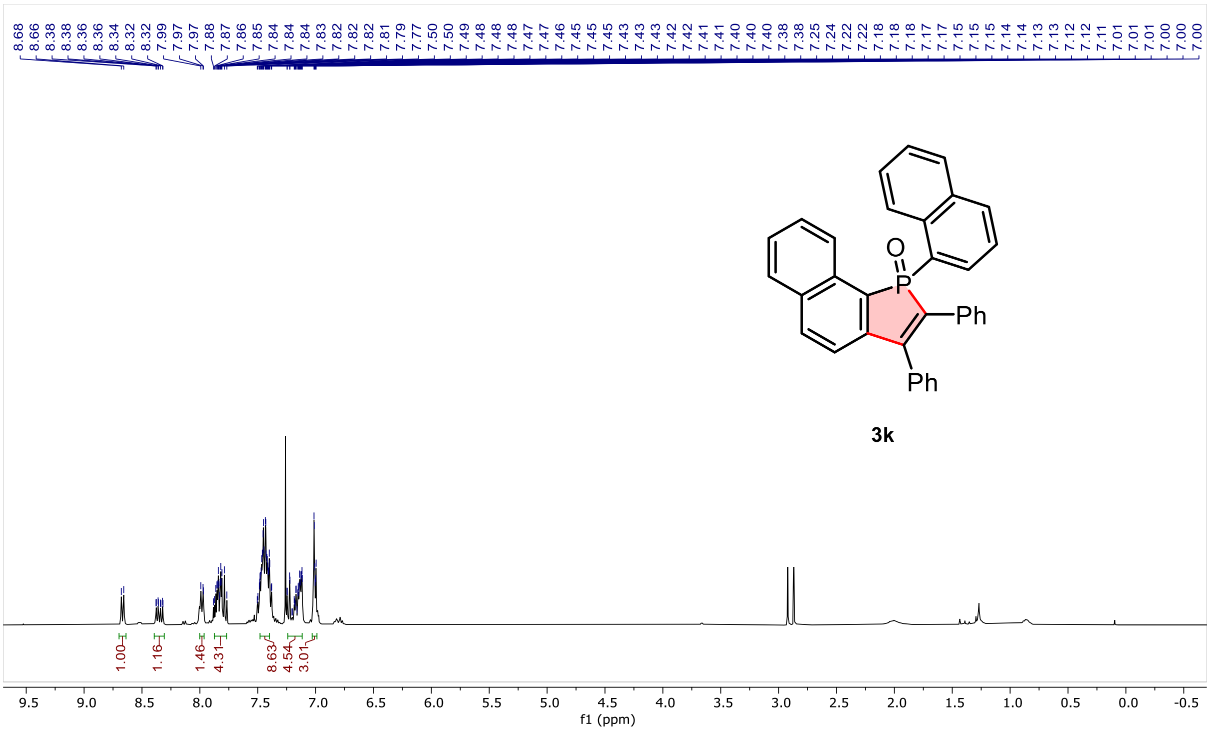

^{13}C NMR (101 MHz, $CDCl_3$) of compound **3k**

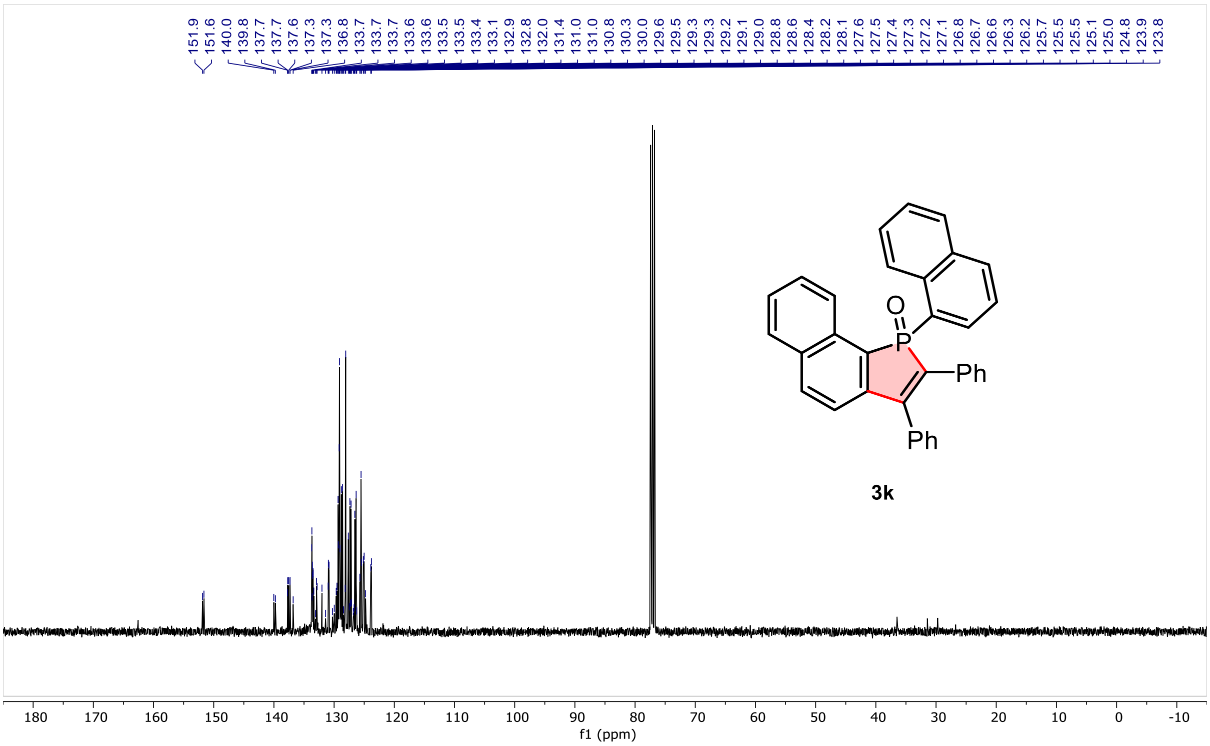

^{31}P NMR (162 MHz, $CDCl_3$) of compound **3k**

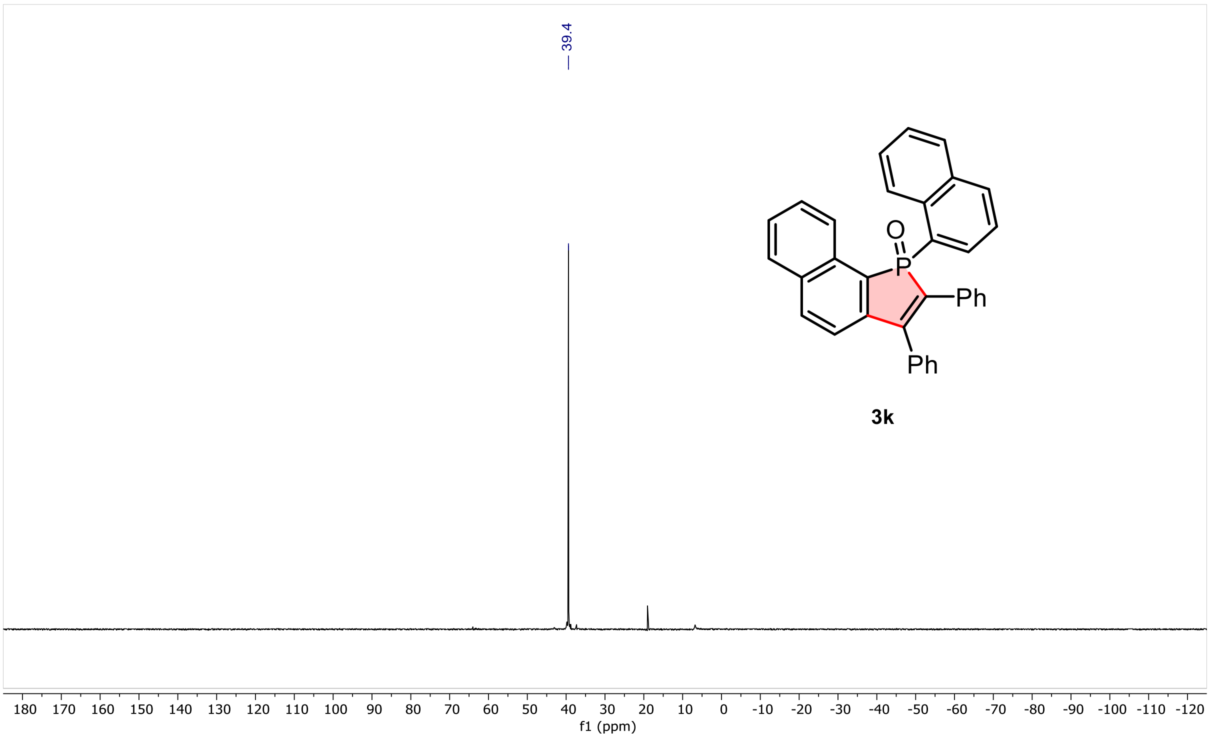

3k

^{1}H NMR (400 MHz, $CDCl_3$) of compound **3l, 3l’**

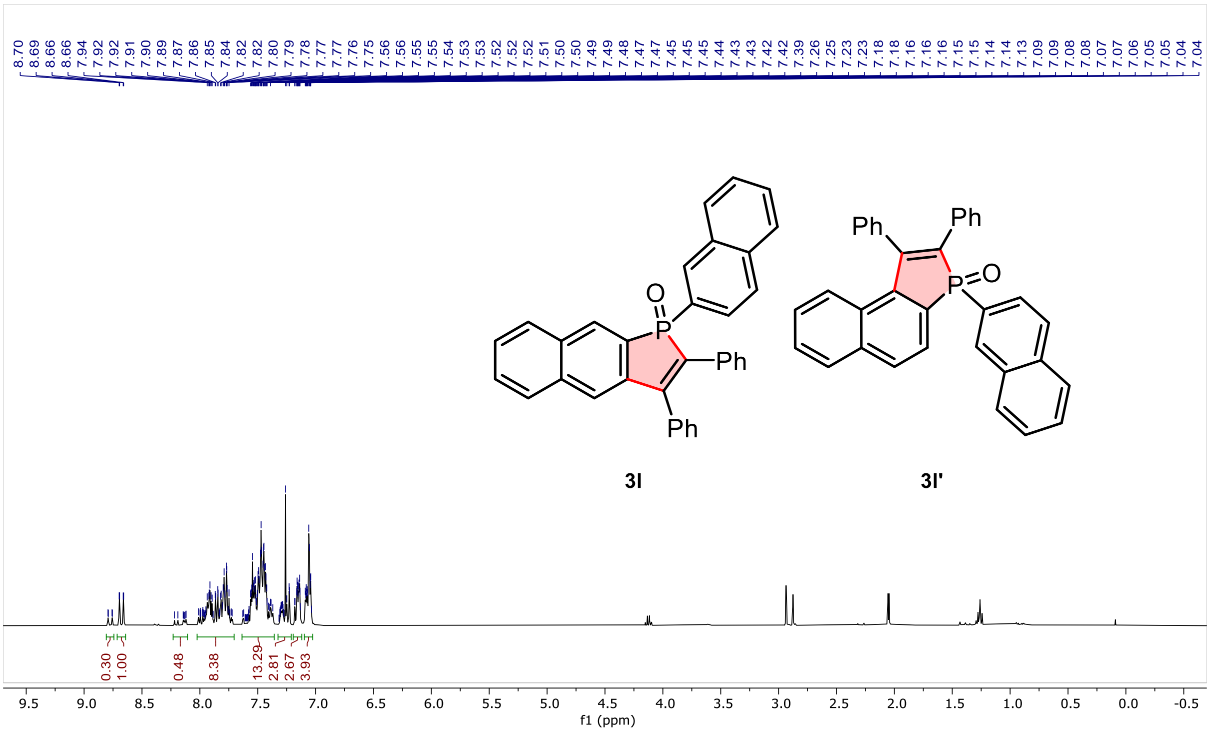

^{13}C NMR (101 MHz, $CDCl_3$) of compound **3l, 3l’**

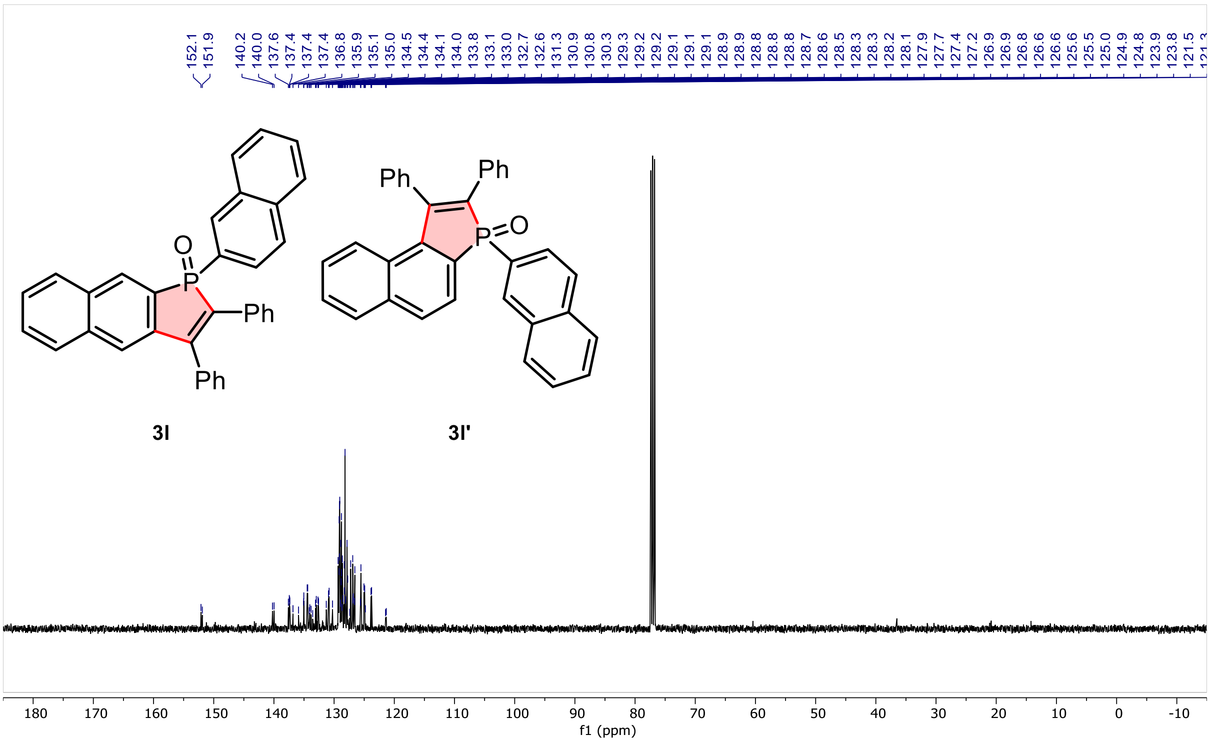

^{31}P NMR (162 MHz, $CDCl_3$) of compound **3l, 3l'**

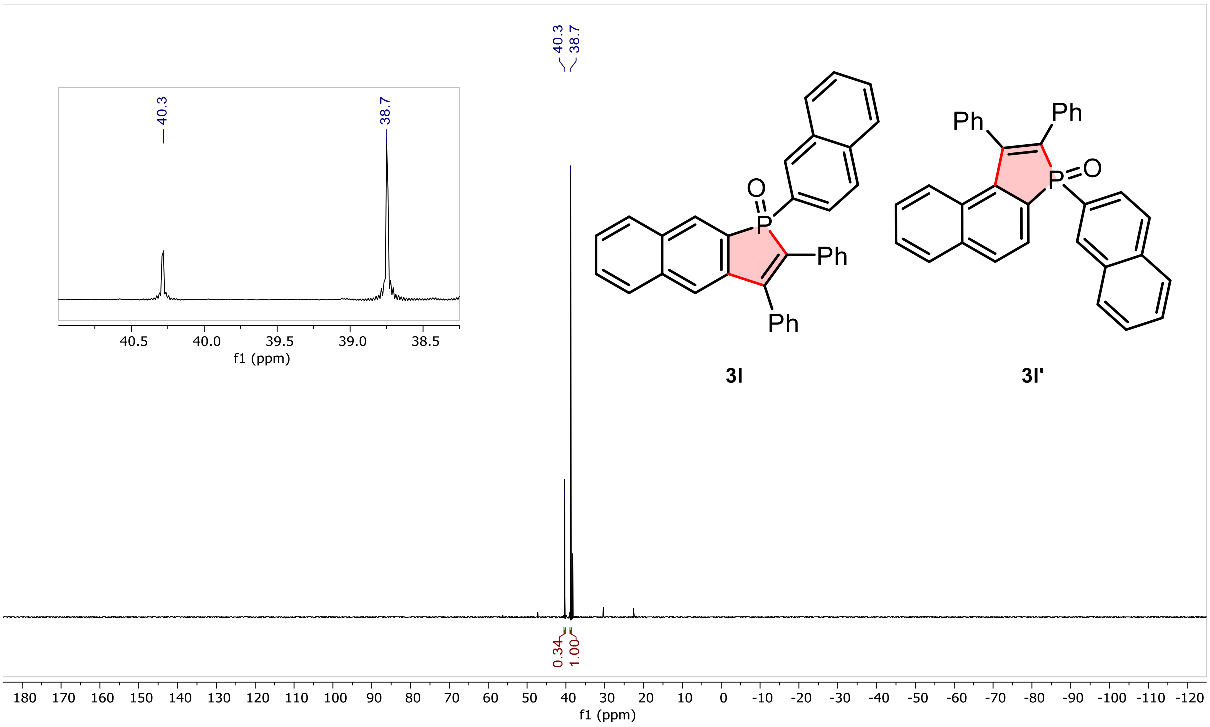

^{1}H NMR (400 MHz, CDCl3) of compound **3m**

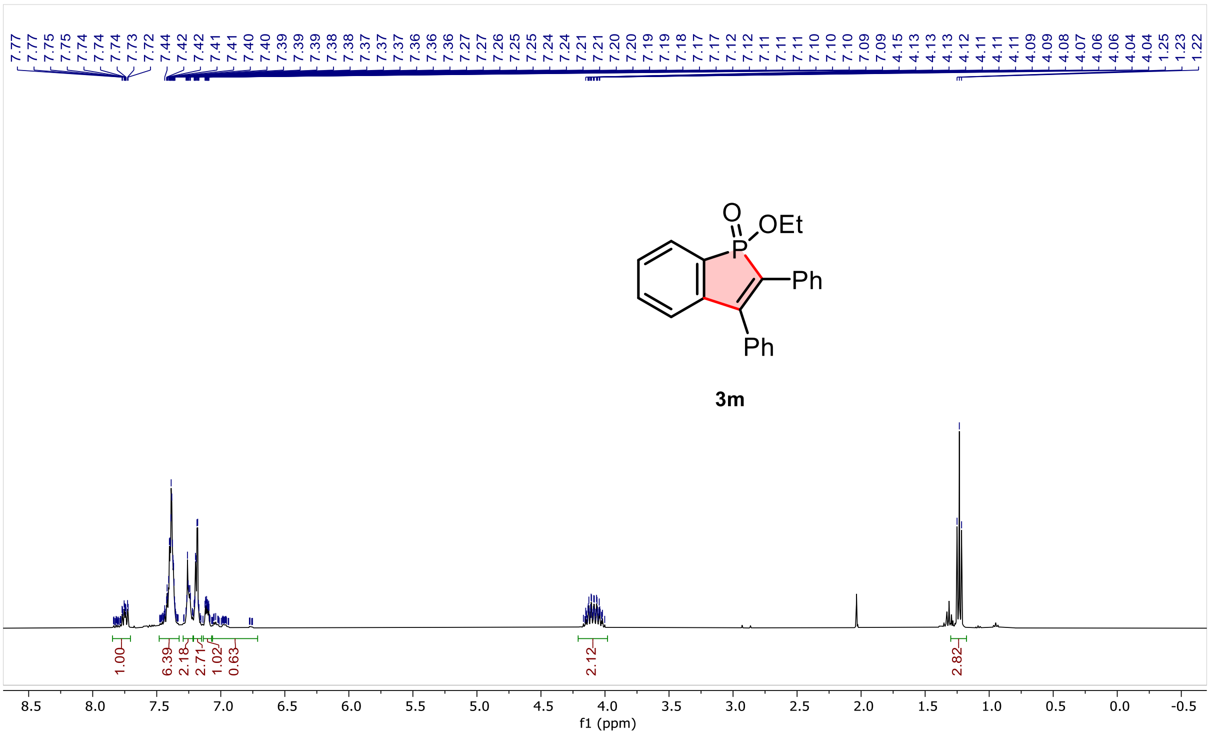

^{13}C NMR (101 MHz, CDCl3) of compound **3m**

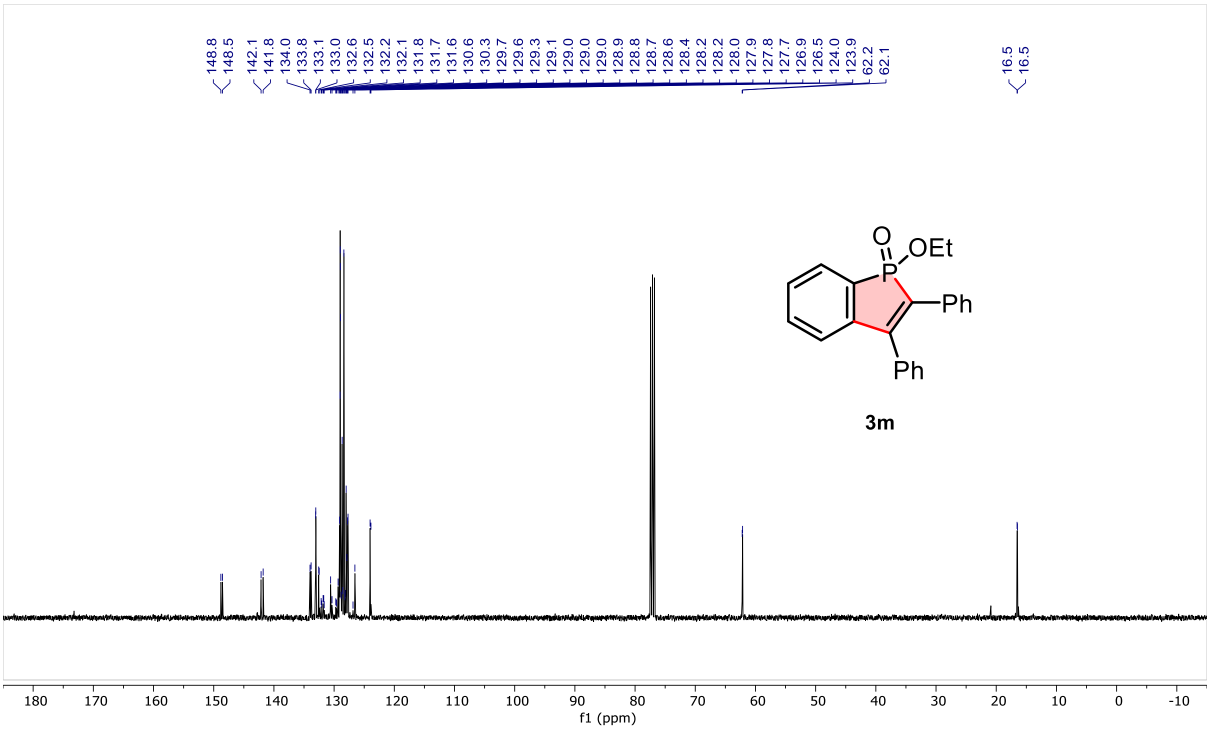

^{31}P NMR (162 MHz, $CDCl_3$) of compound **3m**

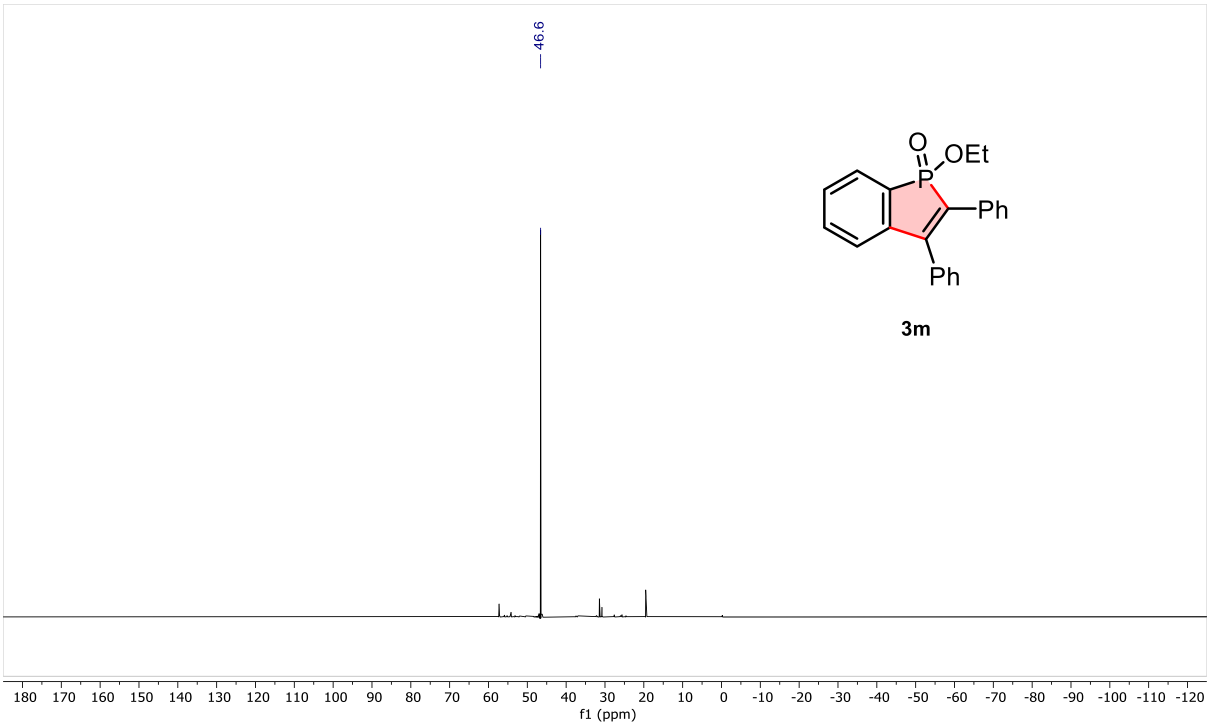

^{1}H NMR (400 MHz, CDCl$_3$) of compound **3n**

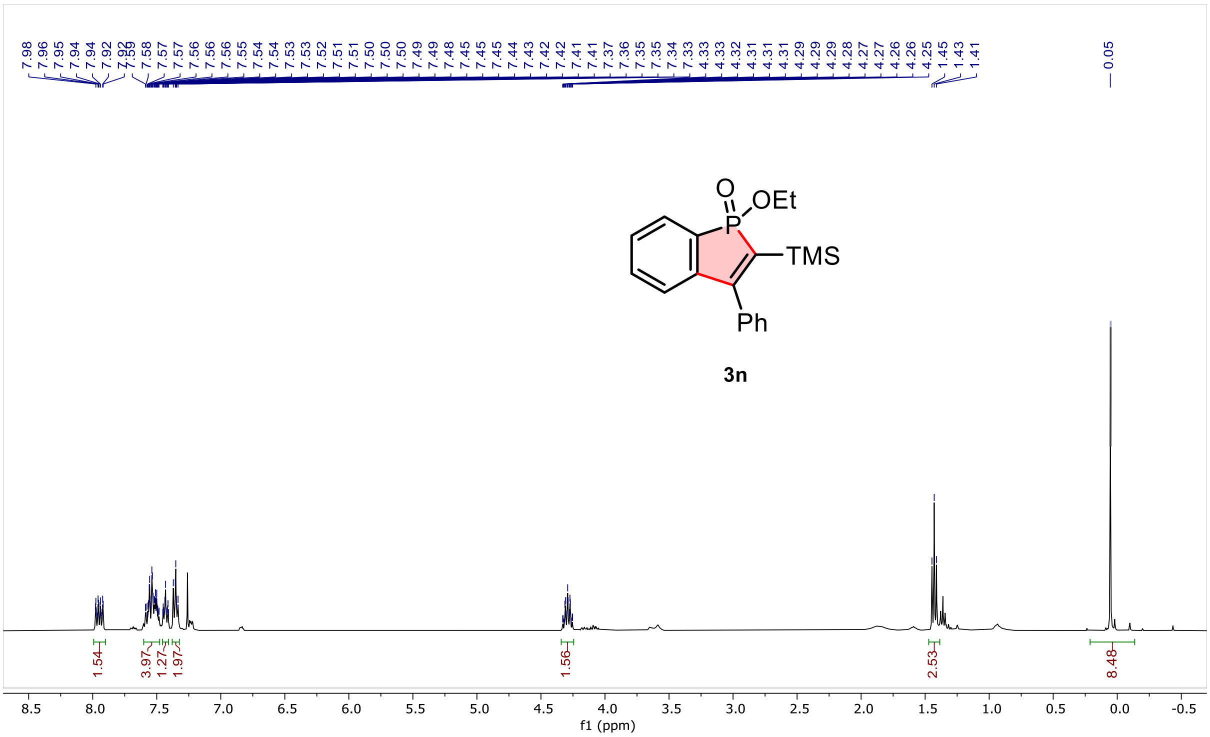

^{13}C NMR (101 MHz, CDCl$_3$) of compound **3n**

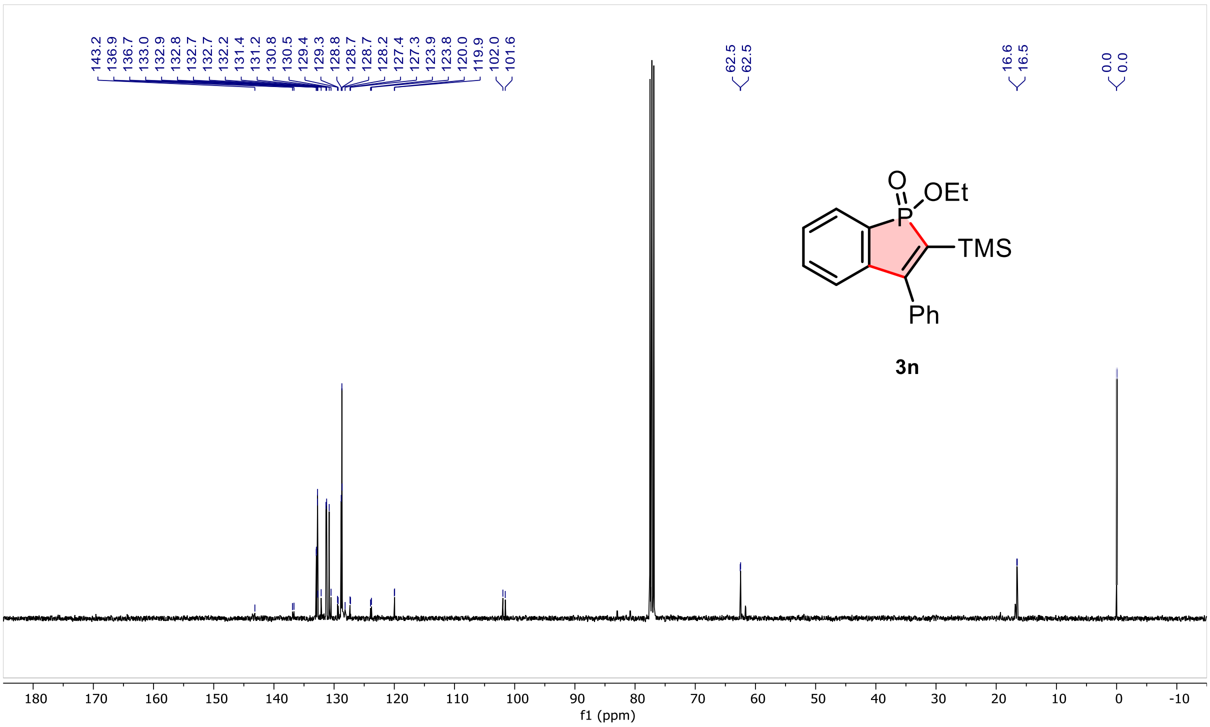

^{31}P NMR (162 MHz, CDCl$_3$) of compound **3n**

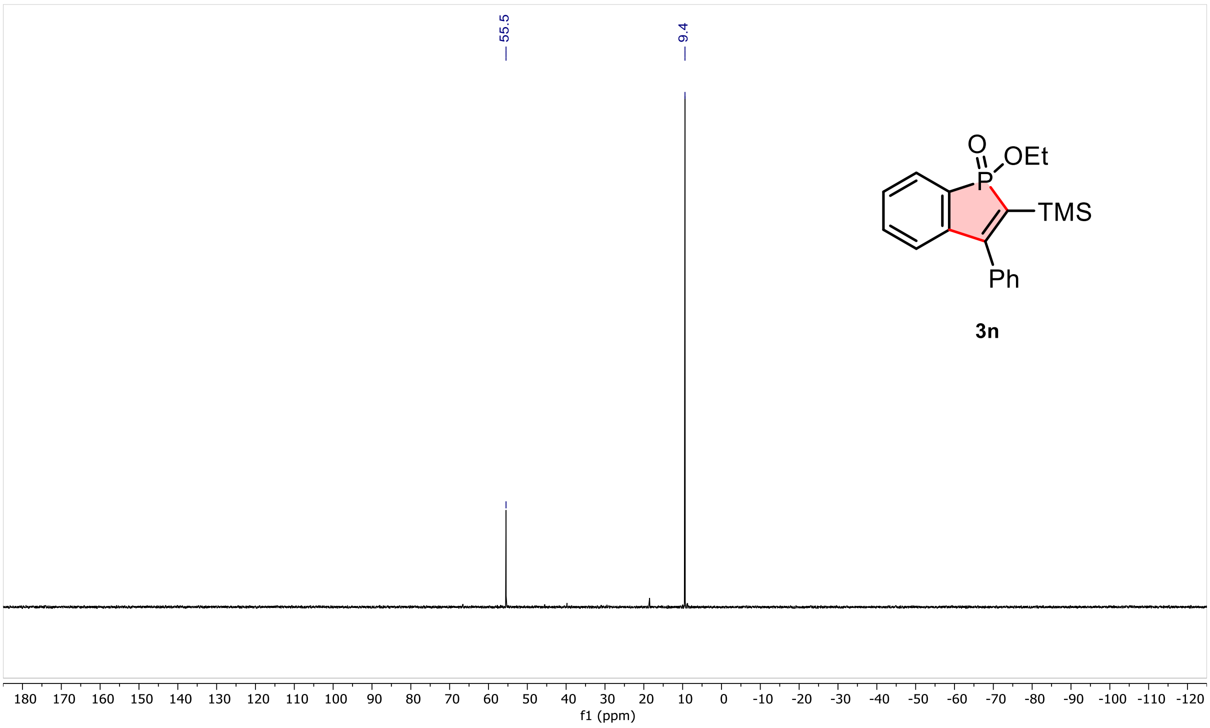

^{1}H NMR (400 MHz, CDCl$_3$) of compound **3o**

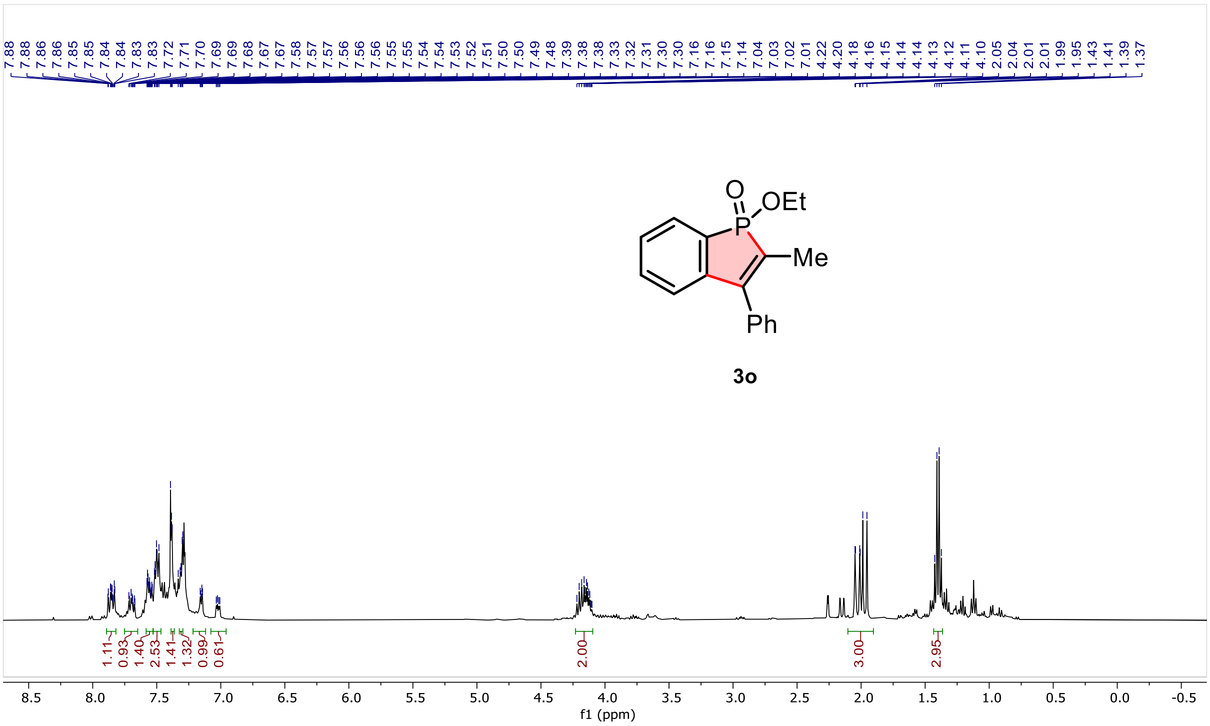

^{13}C NMR (101 MHz, CDCl$_3$) of compound **3o**

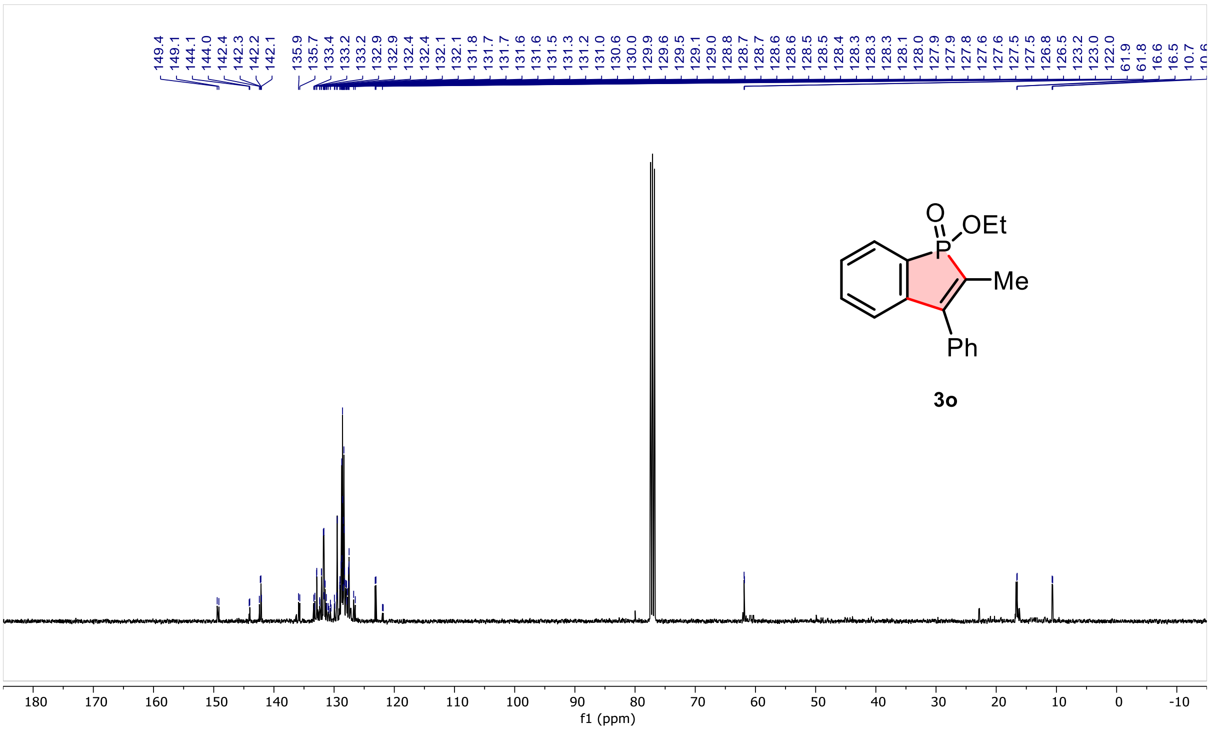

^{31}P NMR (162 MHz, $CDCl_3$) of compound **3o**

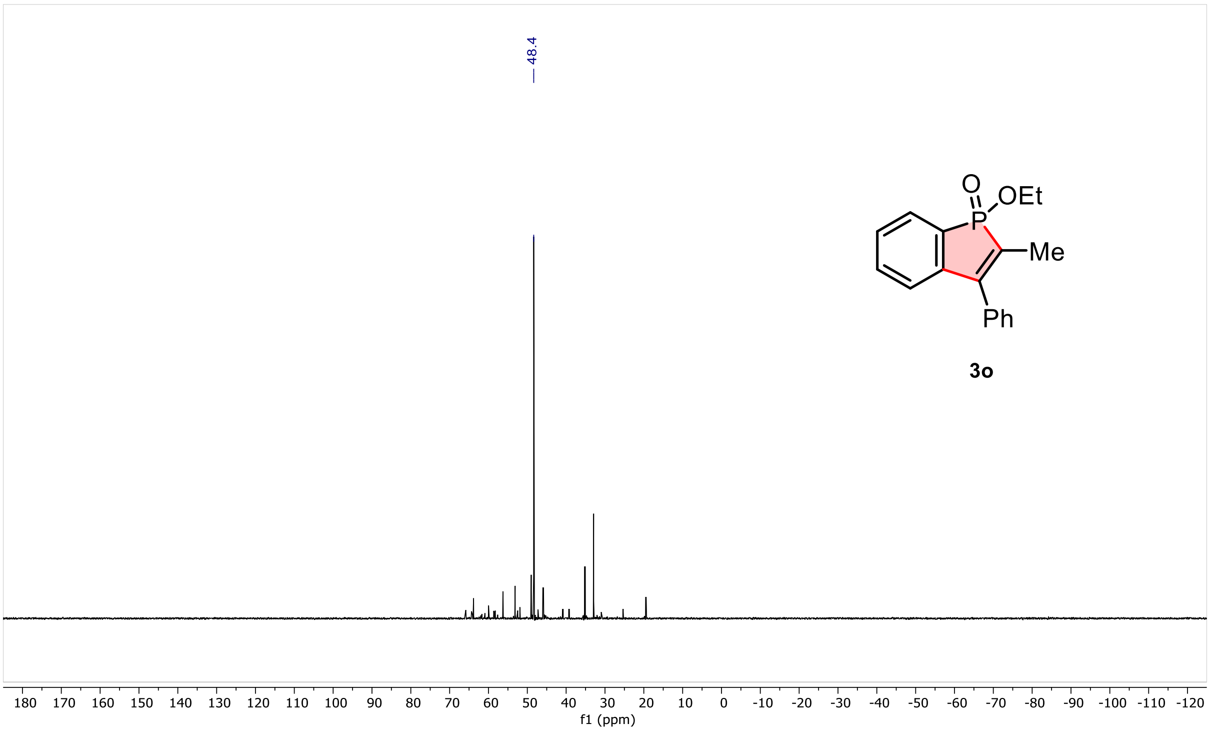

^{1}H NMR (400 MHz, $CDCl_3$) of compound **3p**

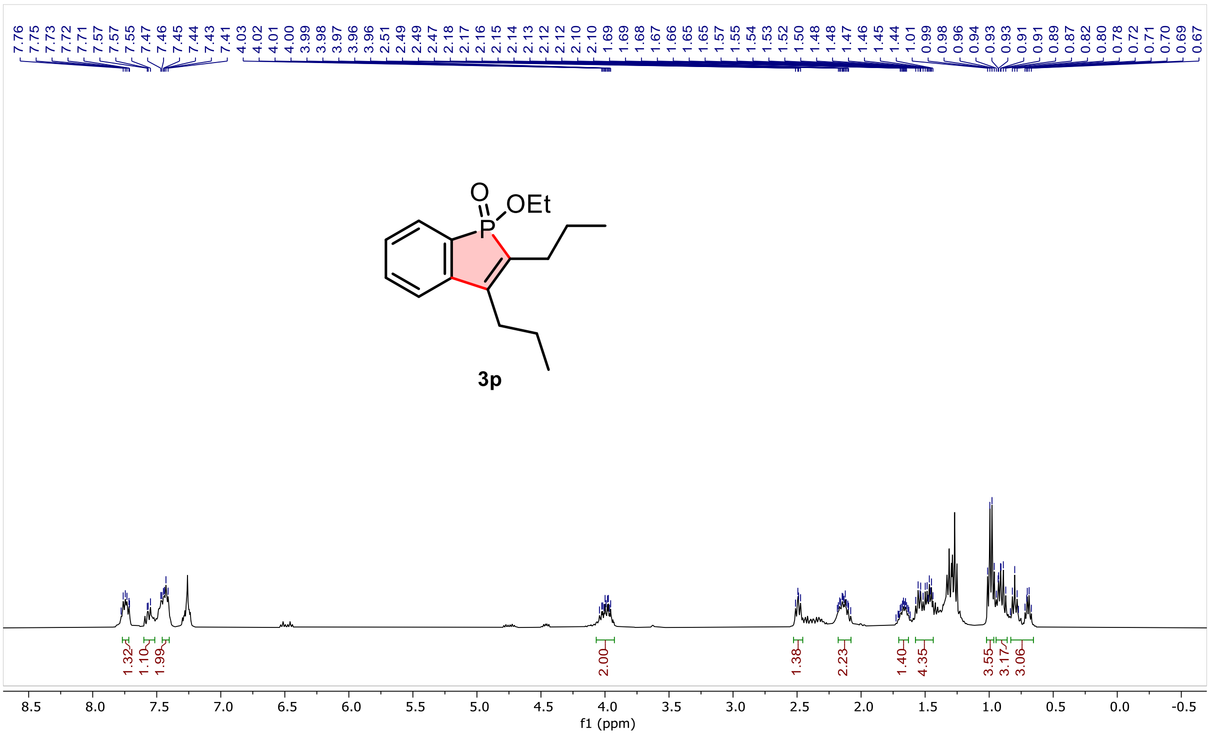

^{13}C NMR (101 MHz, $CDCl_3$) of compound **3p**

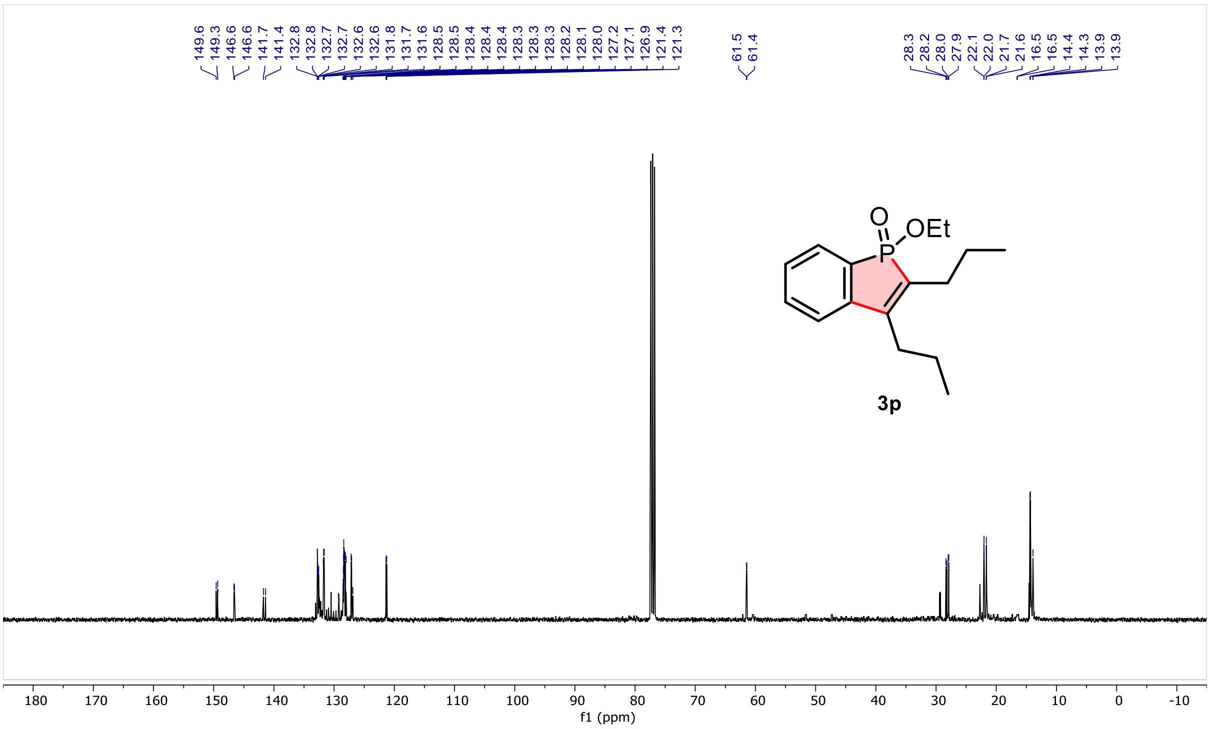

^{31}P NMR (162 MHz, $CDCl_3$) of compound **3p**

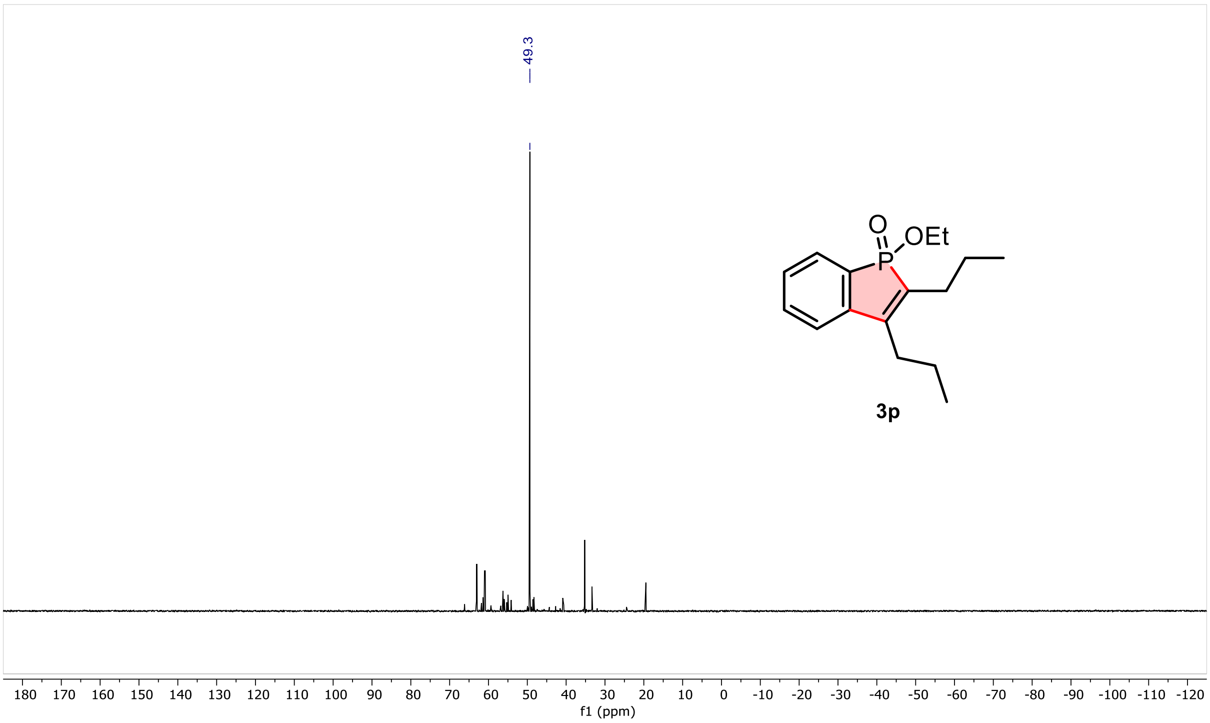

^{1}H NMR (400 MHz, $CDCl_3$) of compound **3q**

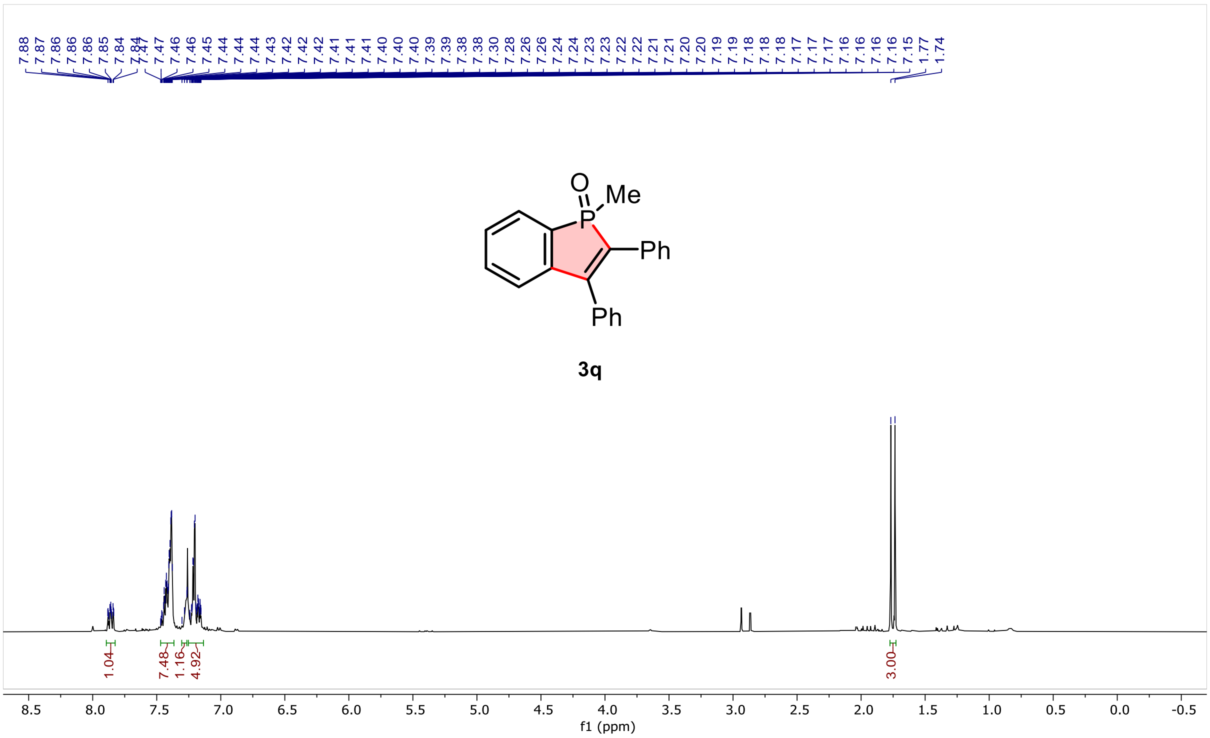

^{13}C NMR (101 MHz, $CDCl_3$) of compound **3q**

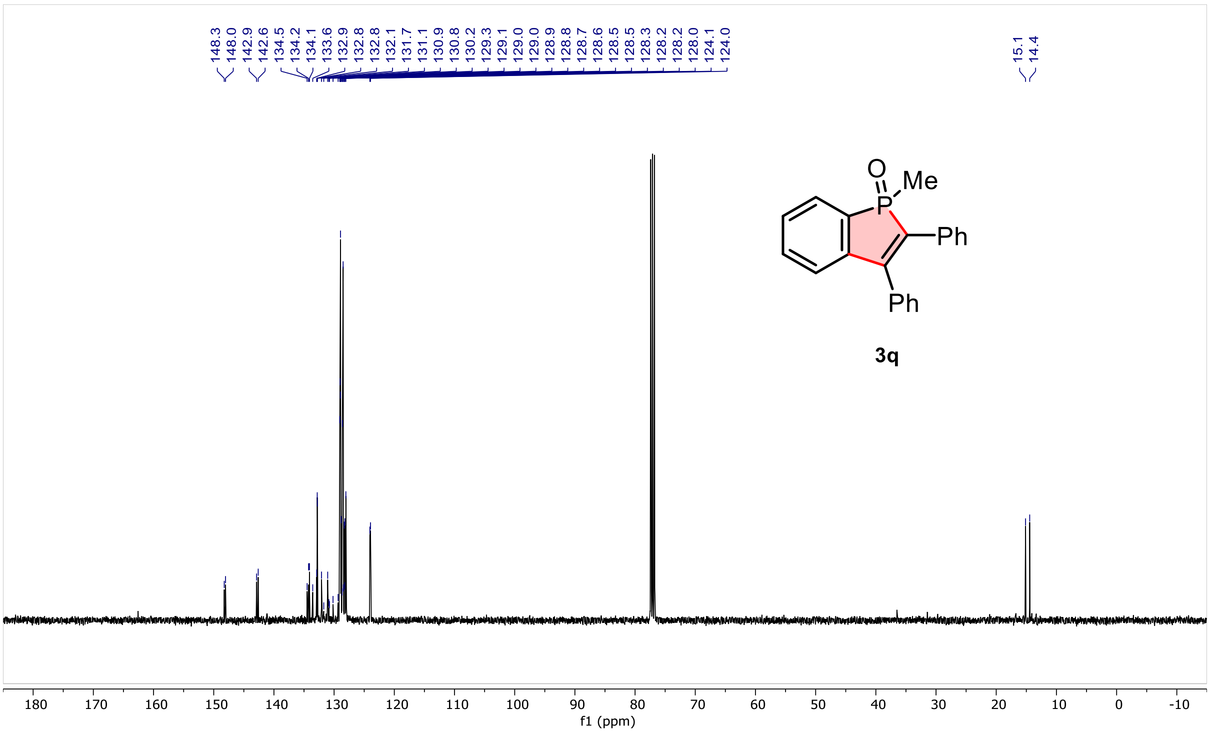

^{31}P NMR (162 MHz, $CDCl_3$) of compound **3q**

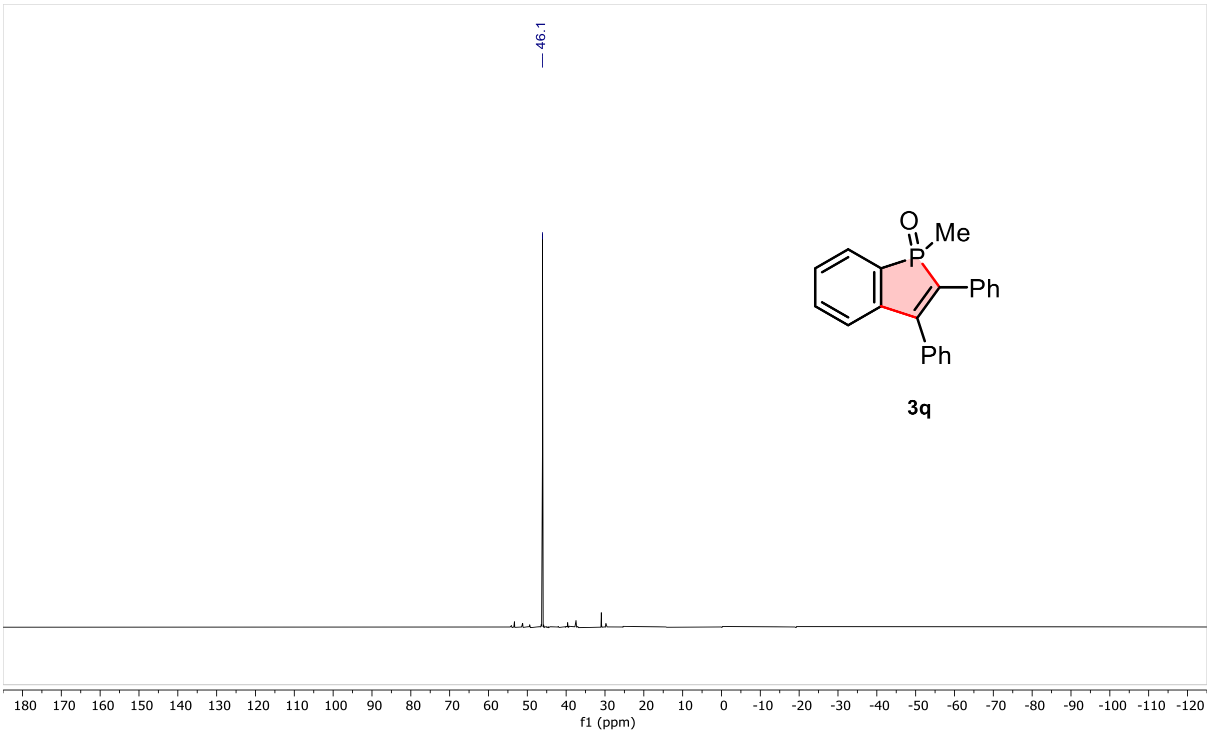

^{1}H NMR (400 MHz, $CDCl_3$) of compound **3r**

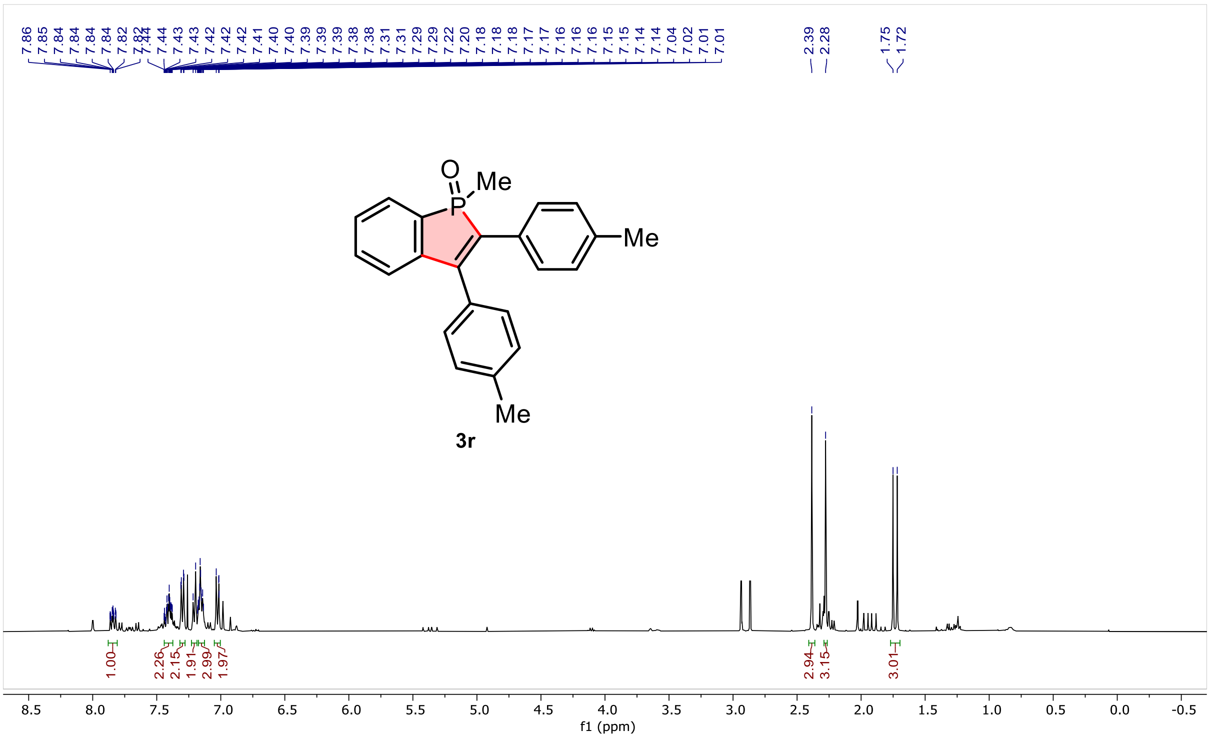

^{13}C NMR (101 MHz, $CDCl_3$) of compound **3r**

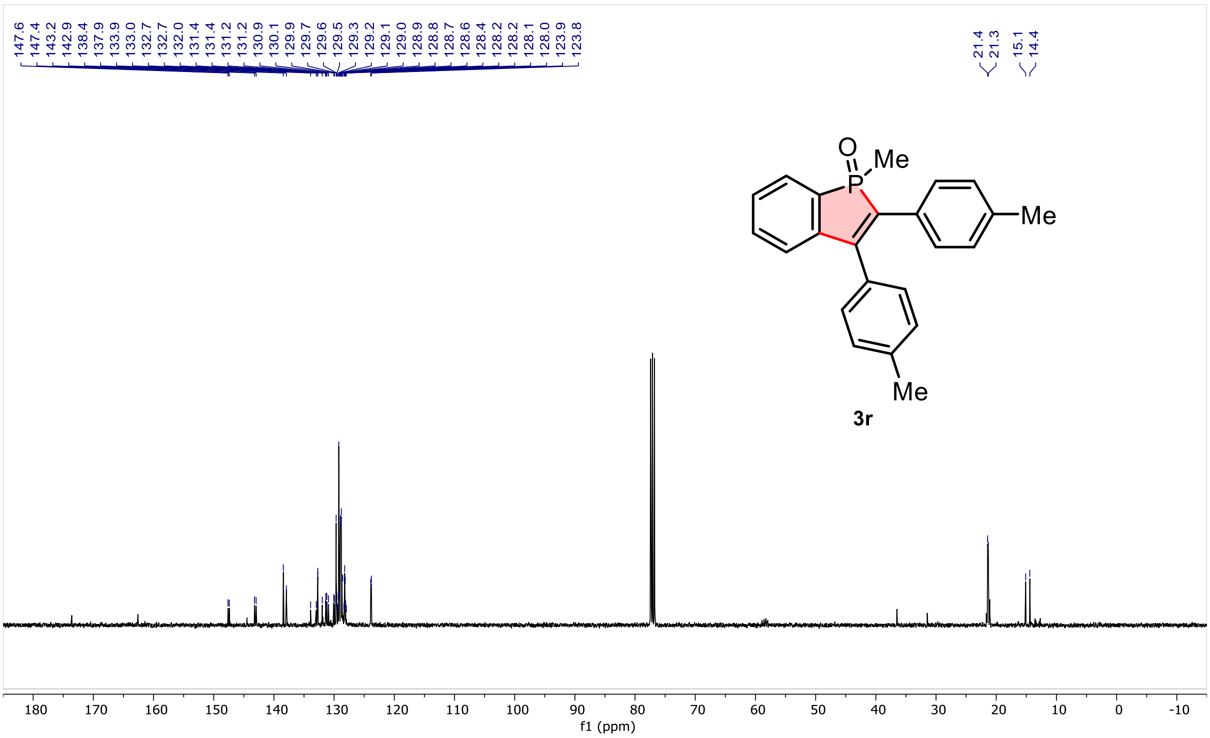

^{31}P NMR (162 MHz, $CDCl_3$) of compound **3r**

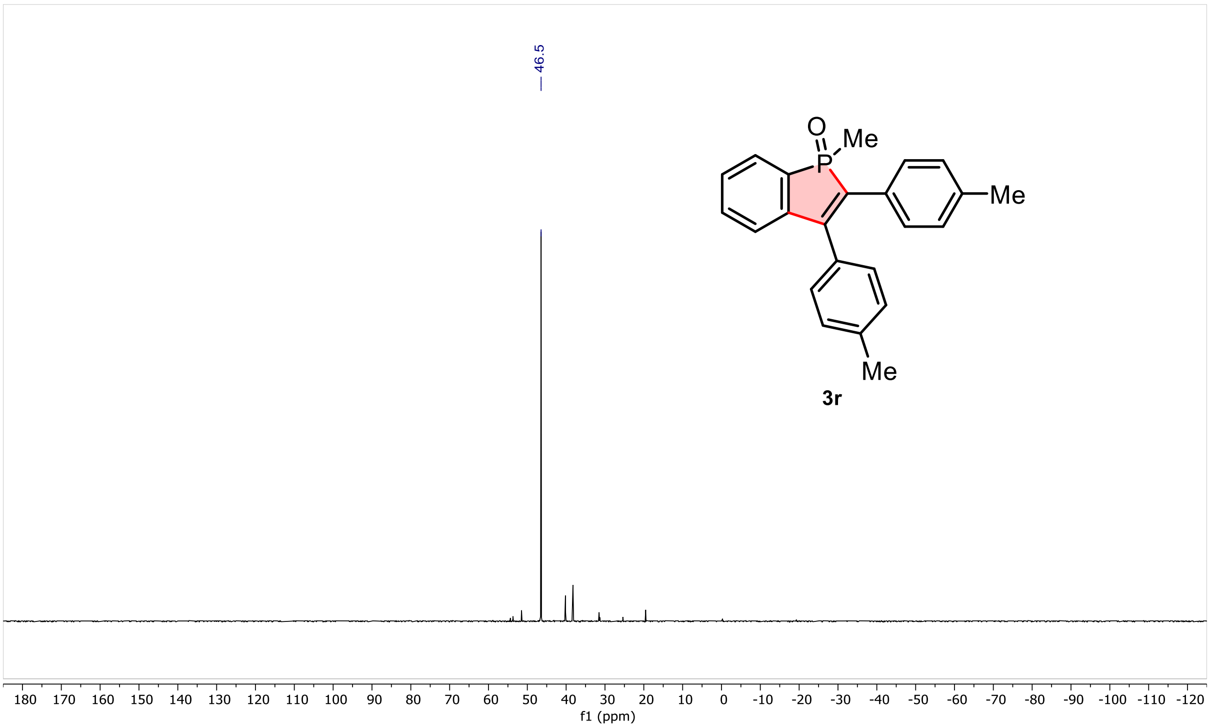

^{1}H NMR (400 MHz, $CDCl_3$) of compound **3s**

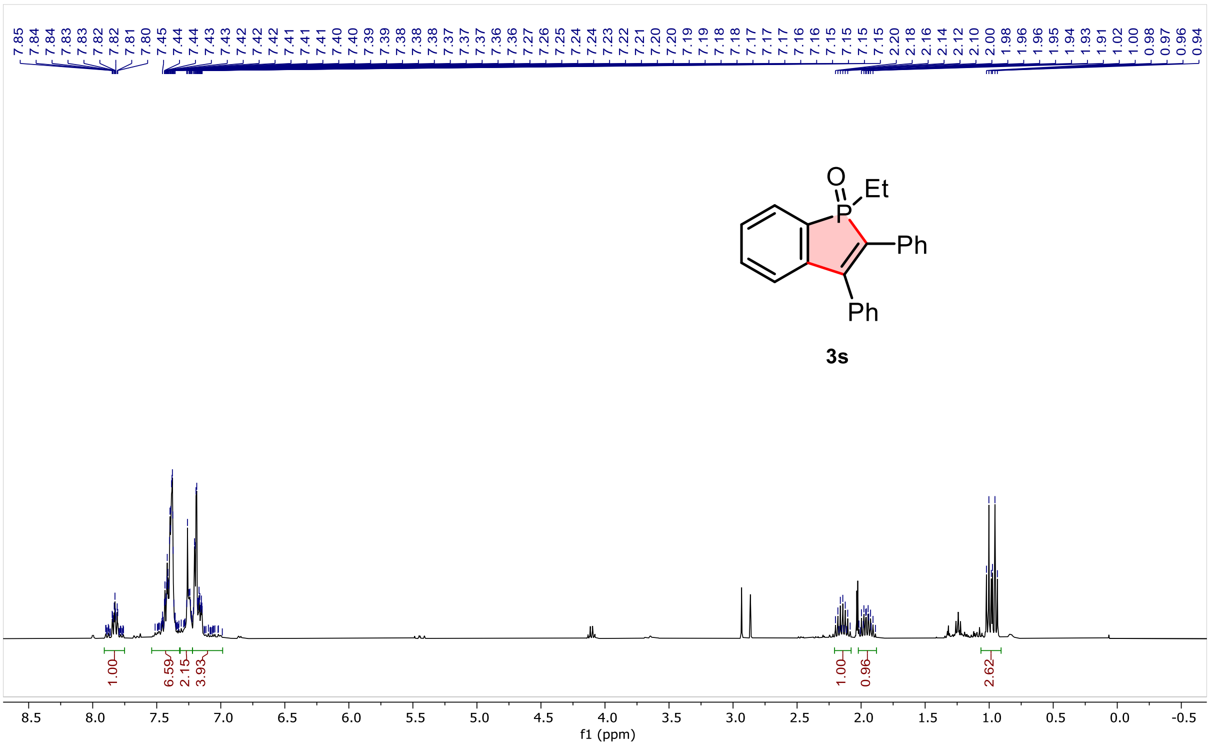

^{13}C NMR (101 MHz, $CDCl_3$) of compound **3s**

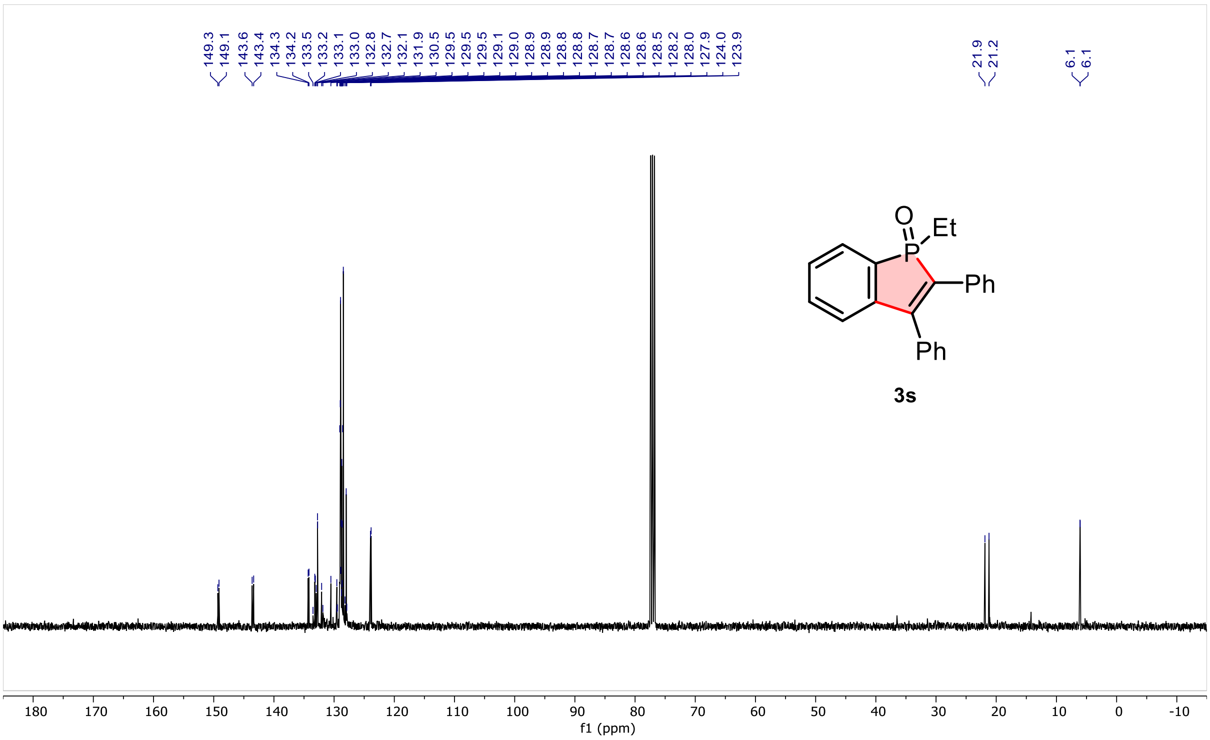

^{31}P NMR (162 MHz, $CDCl_3$) of compound **3s**

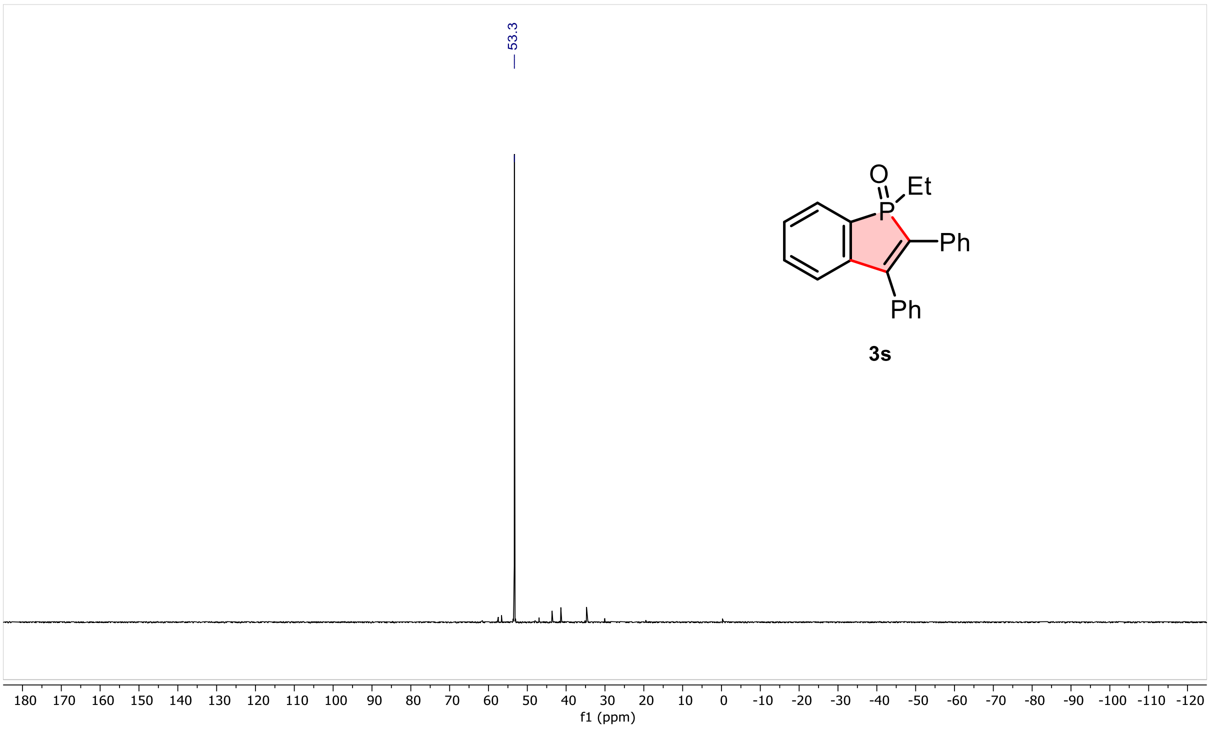

^{1}H NMR (400 MHz, $CDCl_3$) of compound **3t**

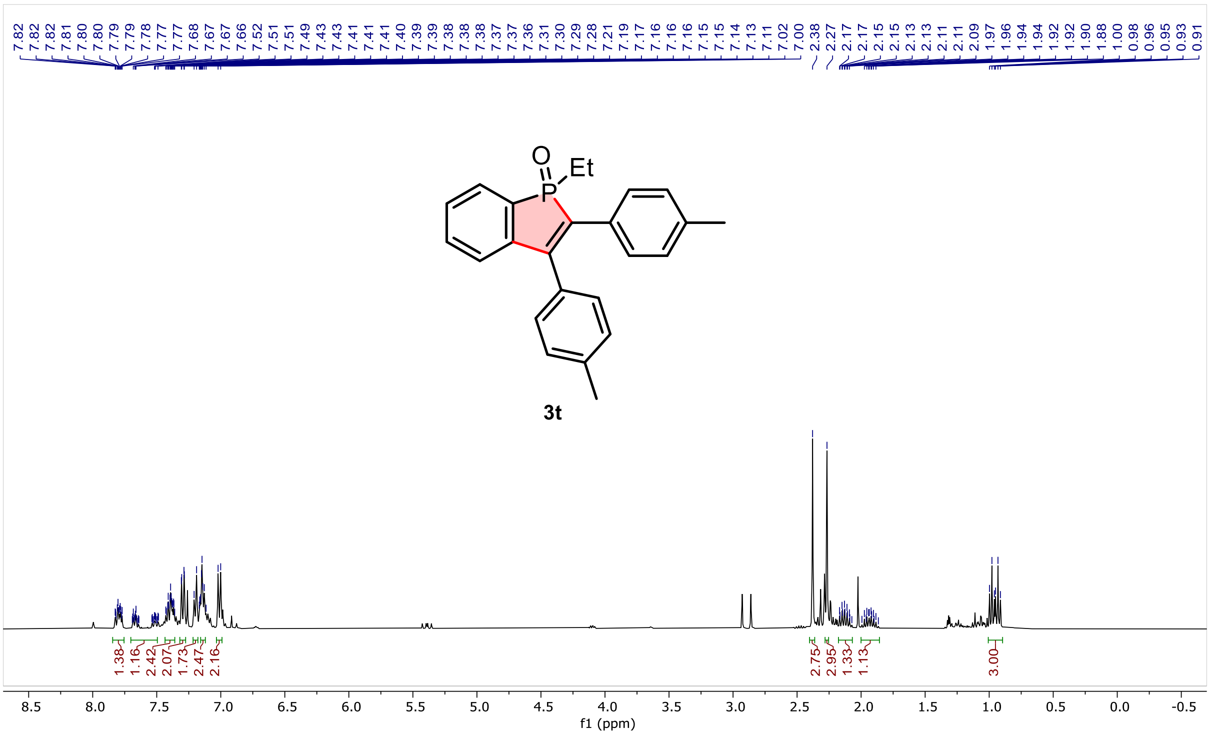

^{13}C NMR (101 MHz, $CDCl_3$) of compound **3t**

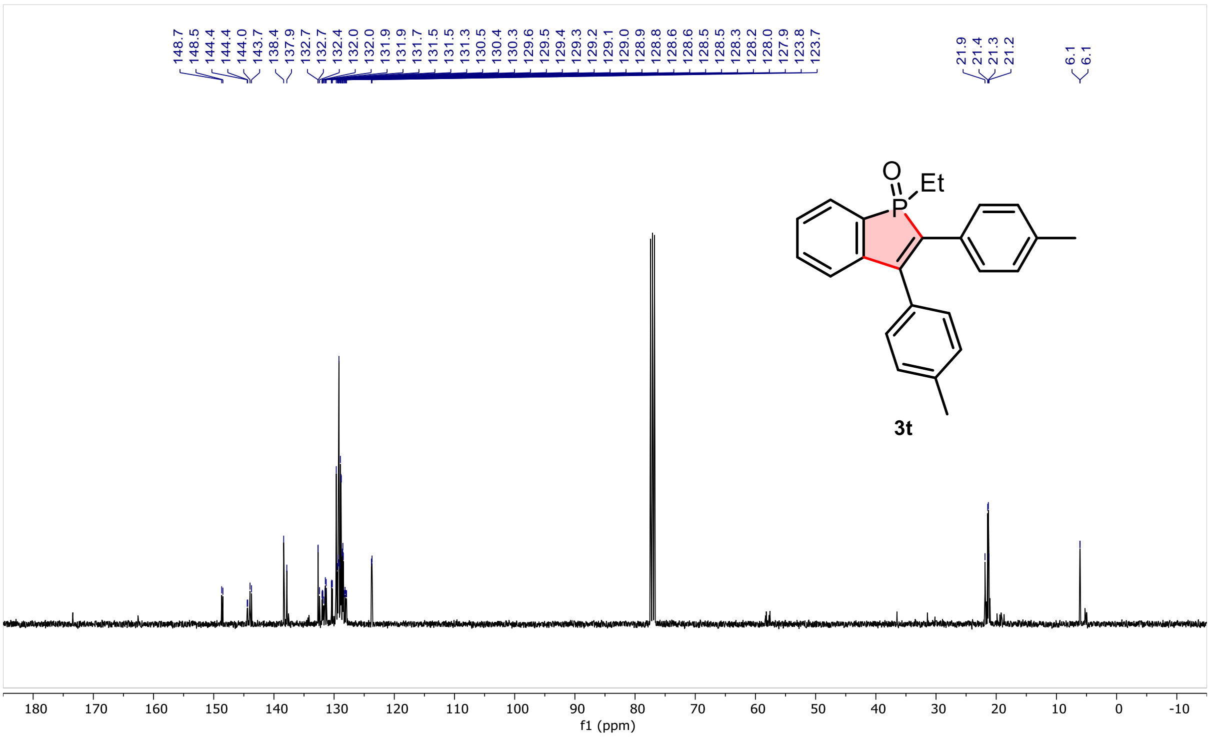

^{31}P NMR (162 MHz, $CDCl_3$) of compound **3t**

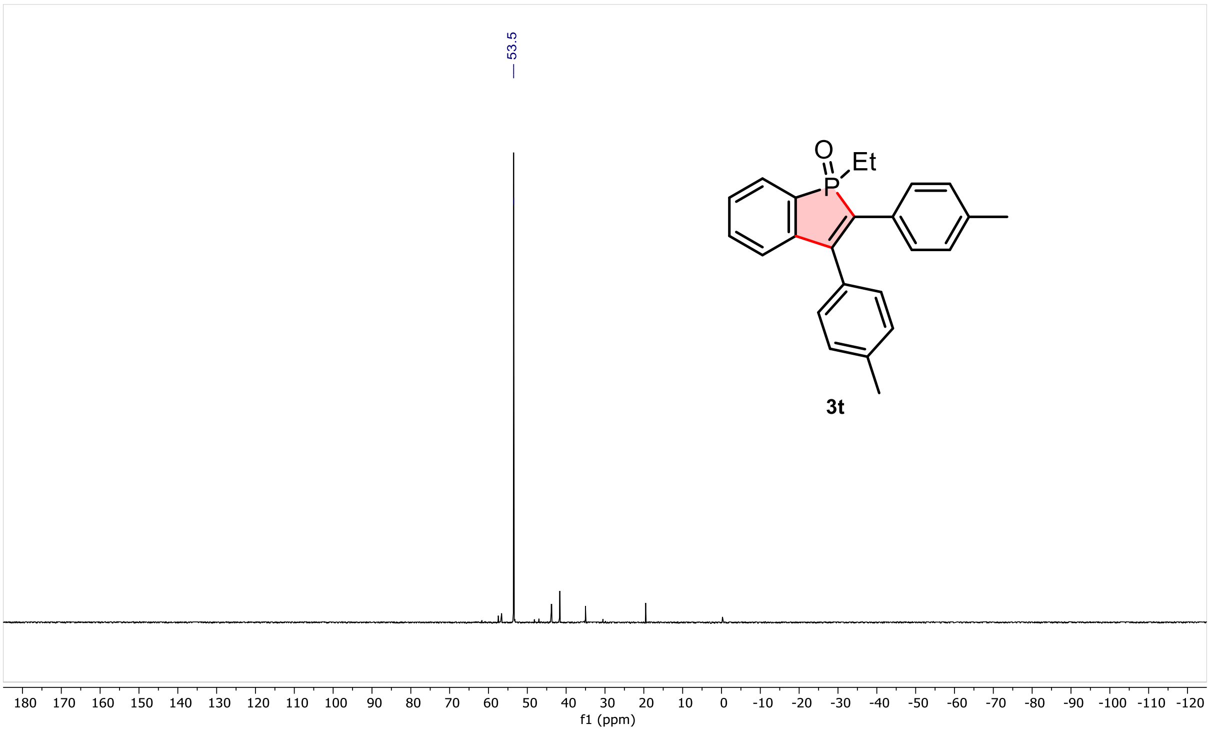

^{1}H NMR (400 MHz, $CDCl_3$) of compound **3u**

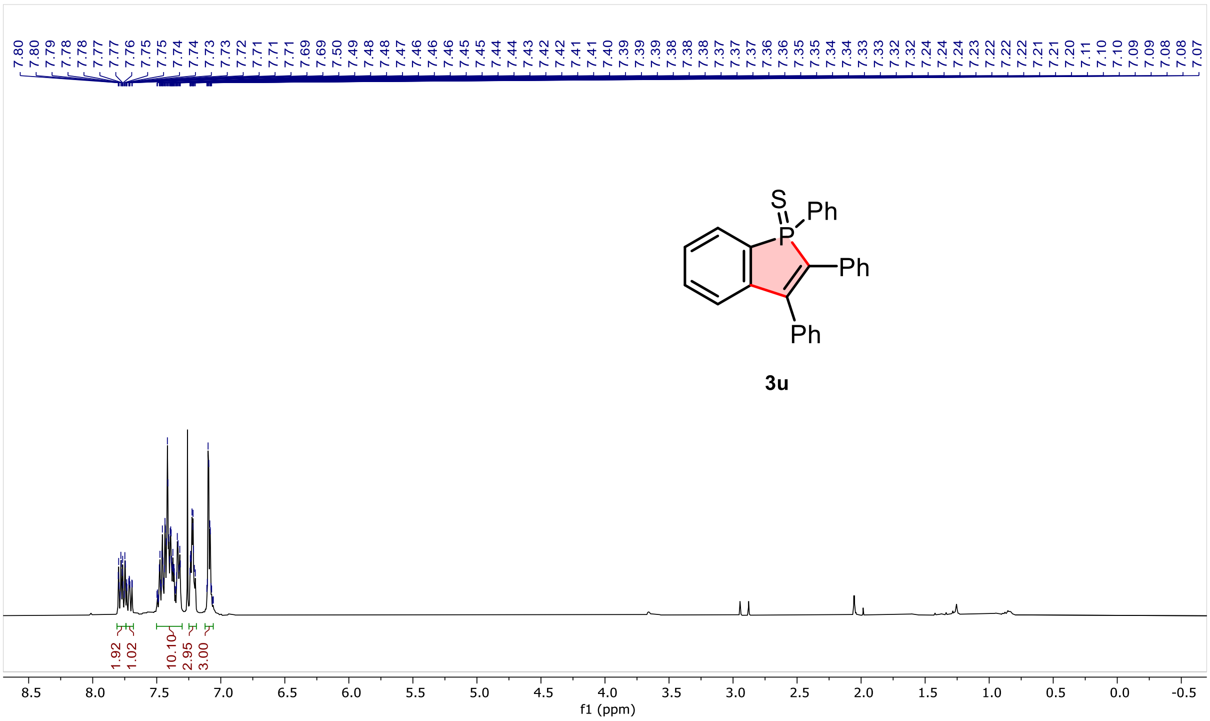

^{13}C NMR (101 MHz, $CDCl_3$) of compound **3u**

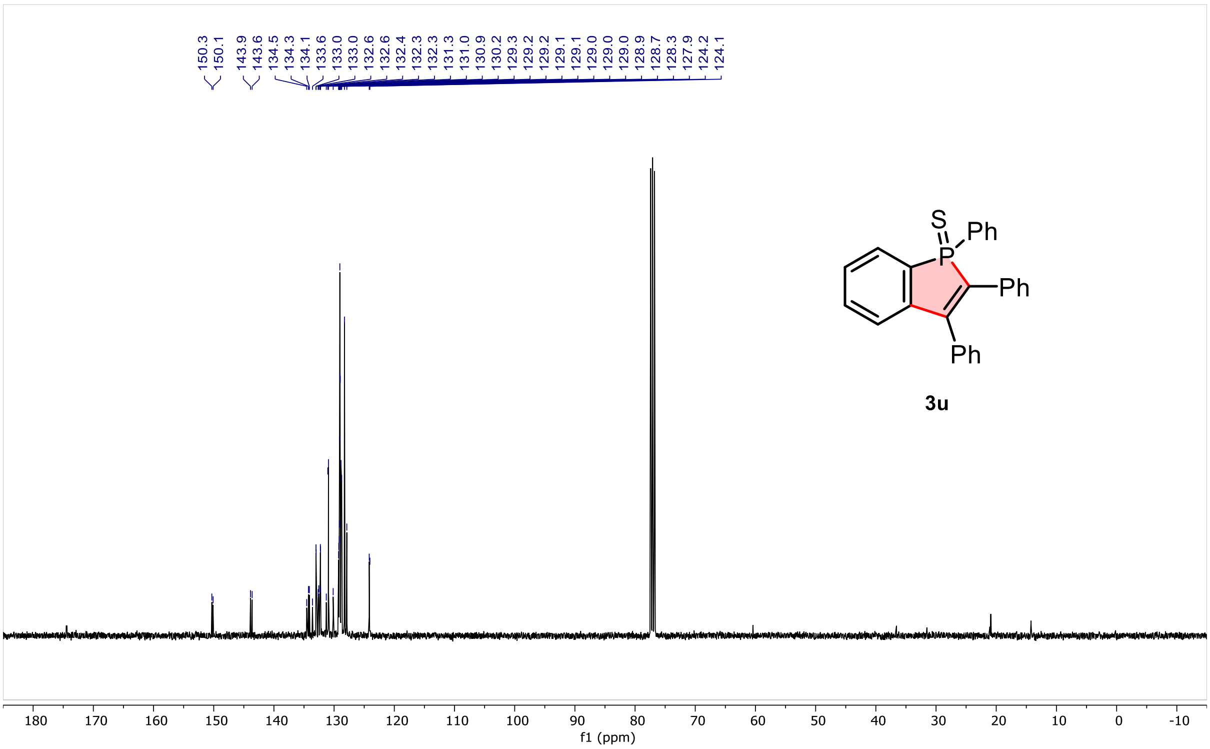

^{31}P NMR (162 MHz, $CDCl_3$) of compound **3u**

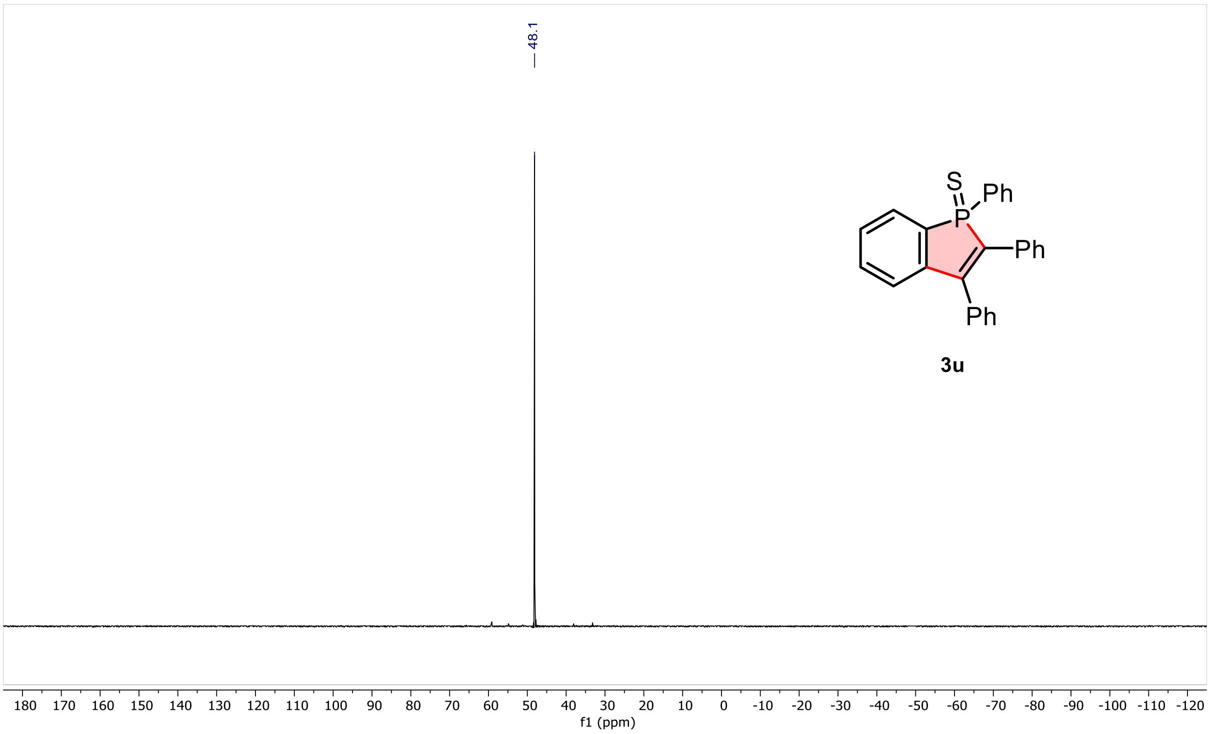

^{1}H NMR (400 MHz, $CDCl_3$) of compound **3v**

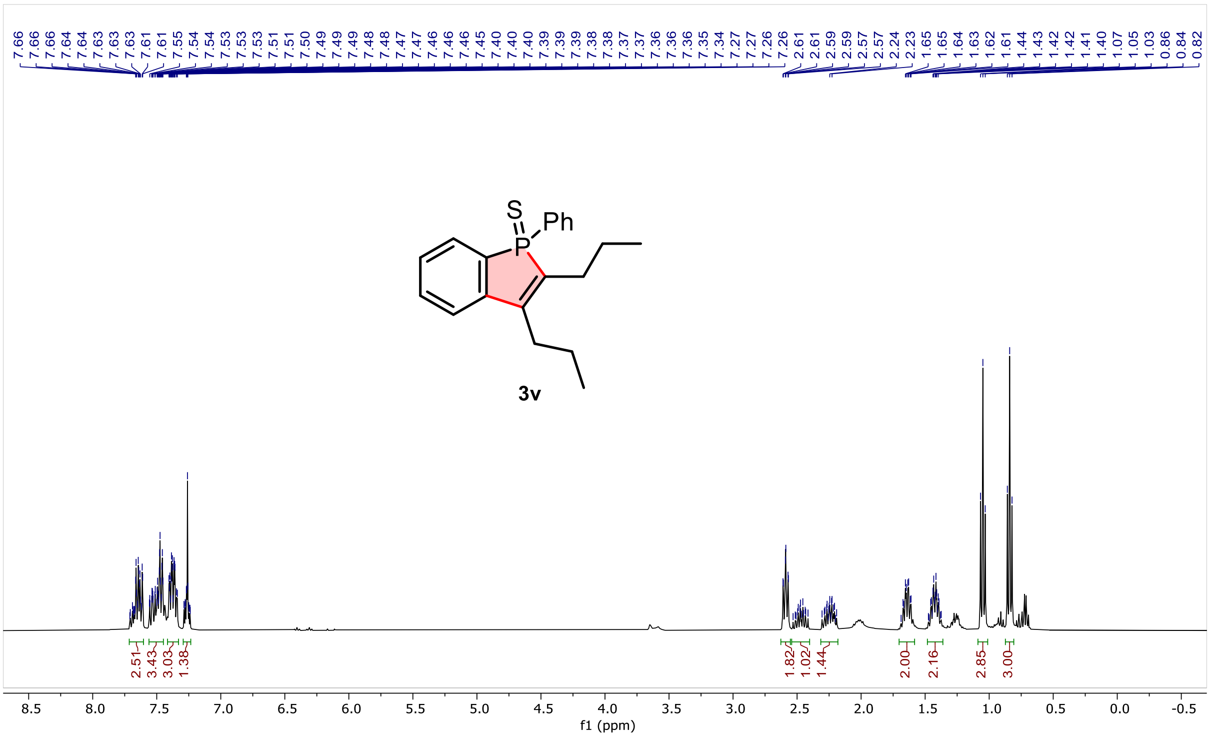

^{13}C NMR (101 MHz, $CDCl_3$) of compound **3v**

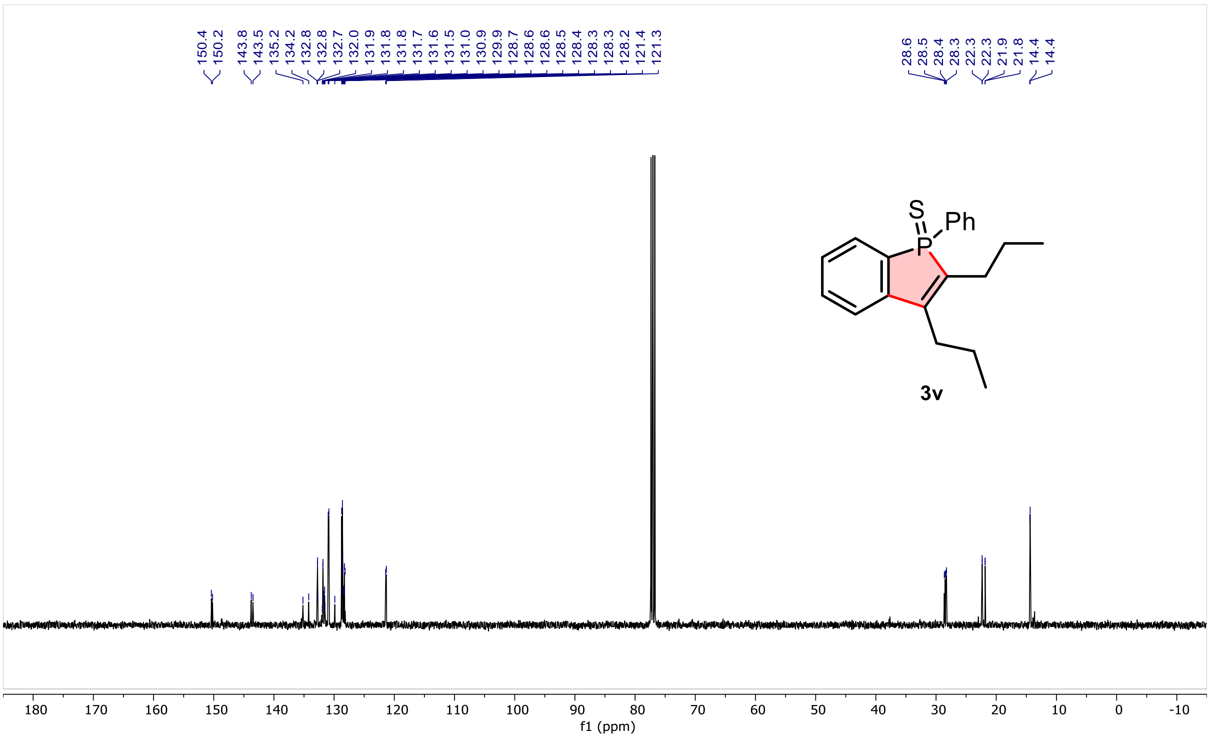

^{31}P NMR (162 MHz, $CDCl_3$) of compound **3v**

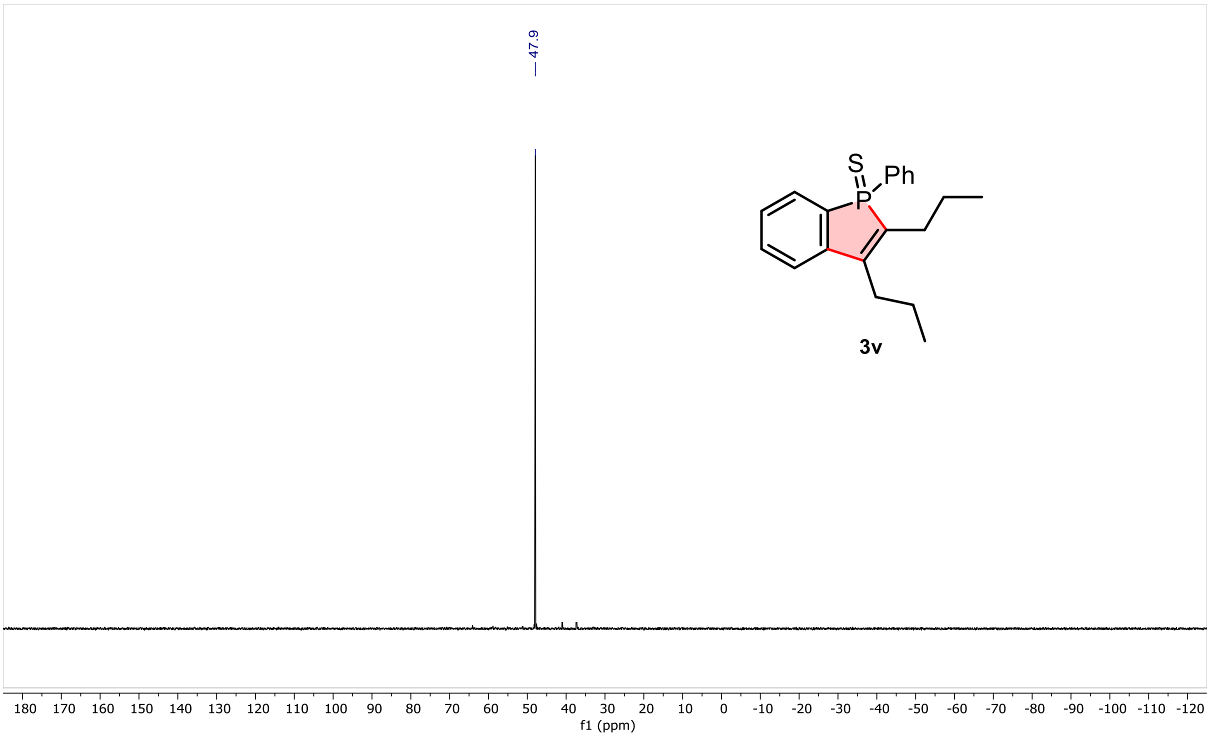

^{1}H NMR (400 MHz, $CDCl_3$) of compound **4a**

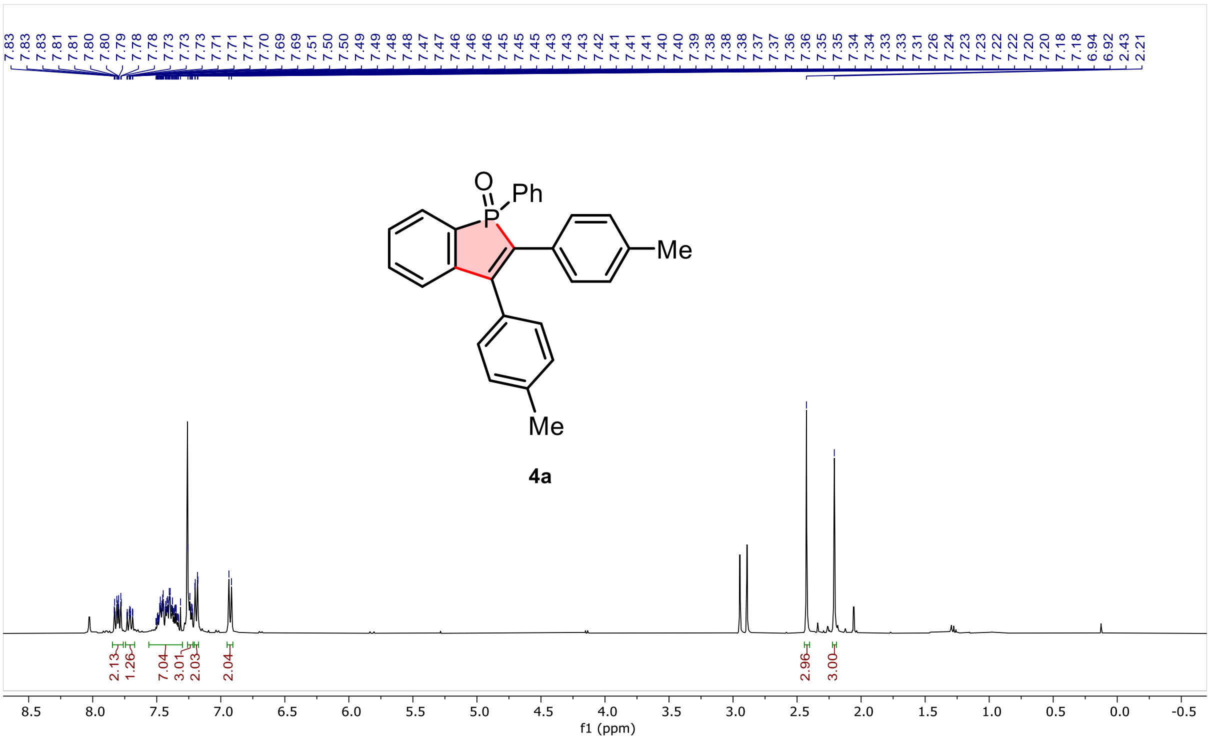

^{13}C NMR (101 MHz, $CDCl_3$) of compound **4a**

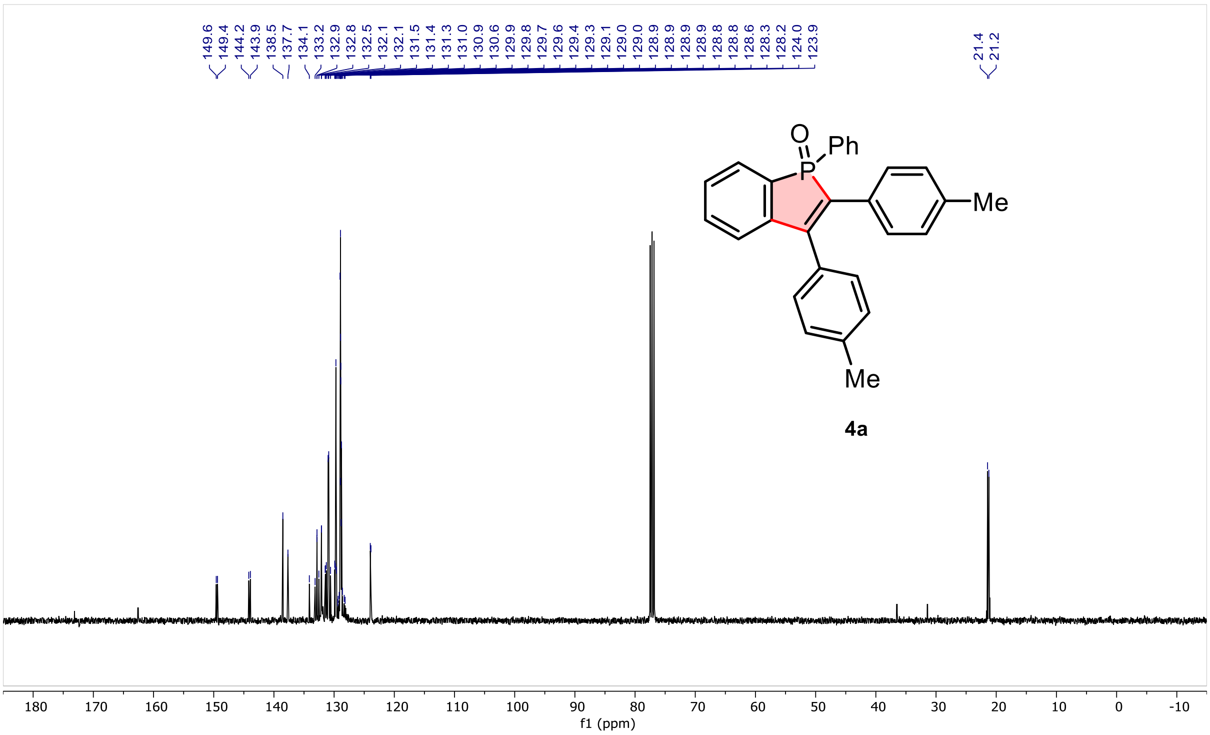

^{31}P NMR (162 MHz, $CDCl_3$) of compound **4a**

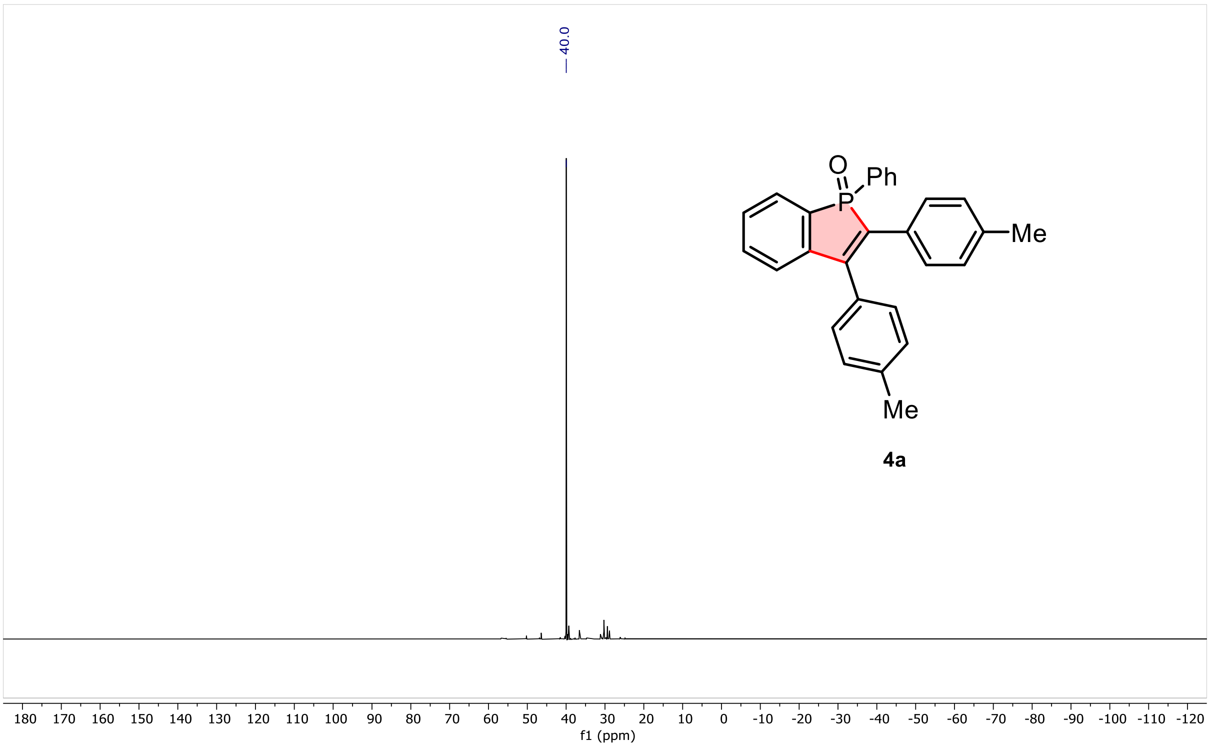

^{1}H NMR (400 MHz, $CDCl_3$) of compound **4b**

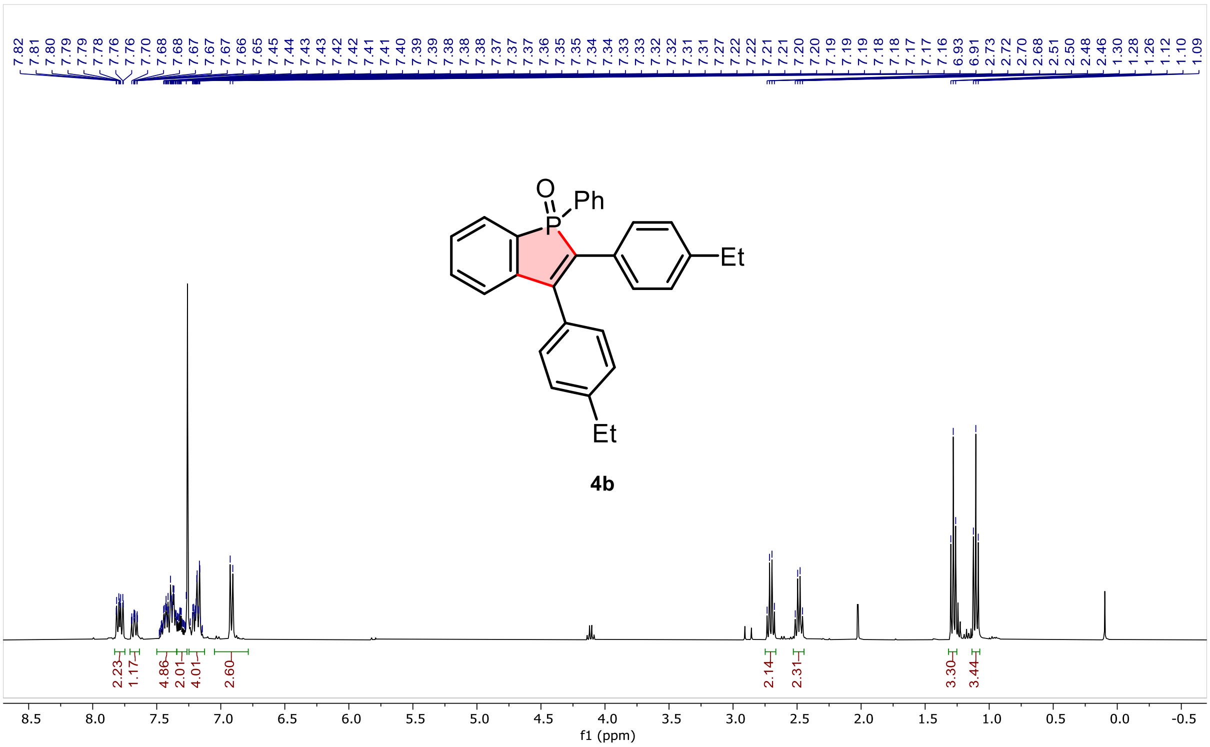

^{13}C NMR (101 MHz, $CDCl_3$) of compound **4b**

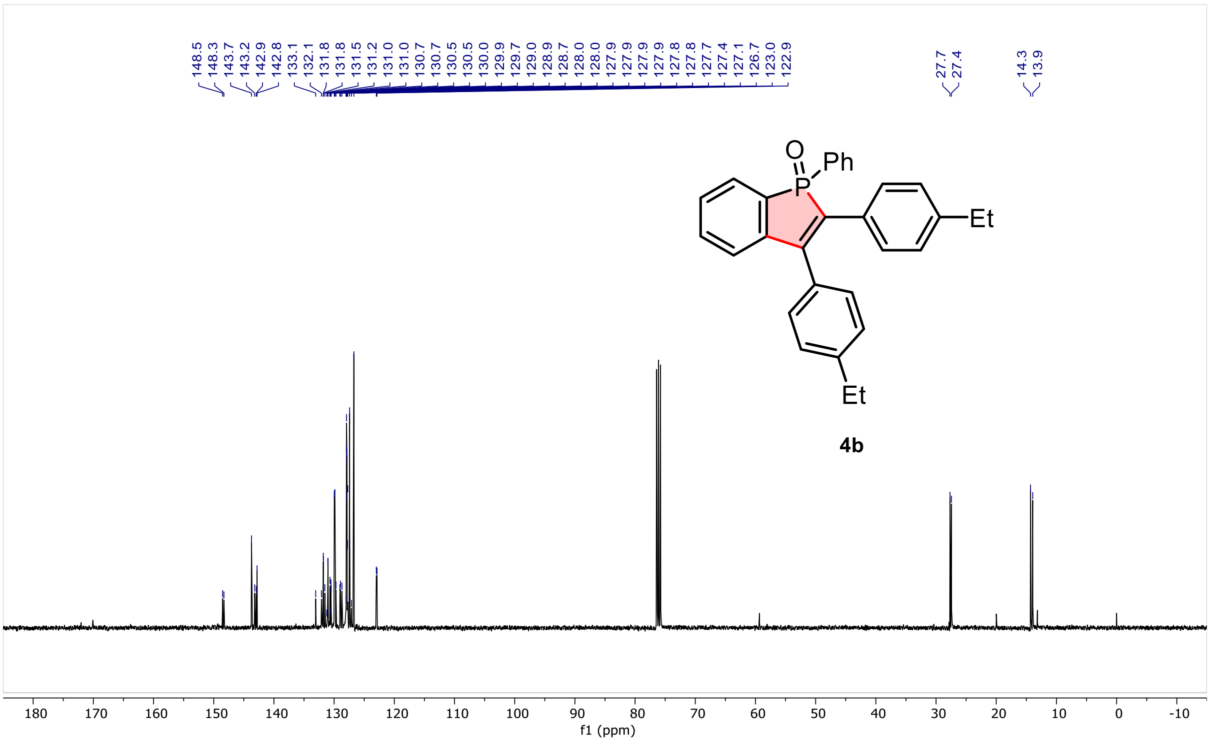

^{31}P NMR (162 MHz, $CDCl_3$) of compound **4b**

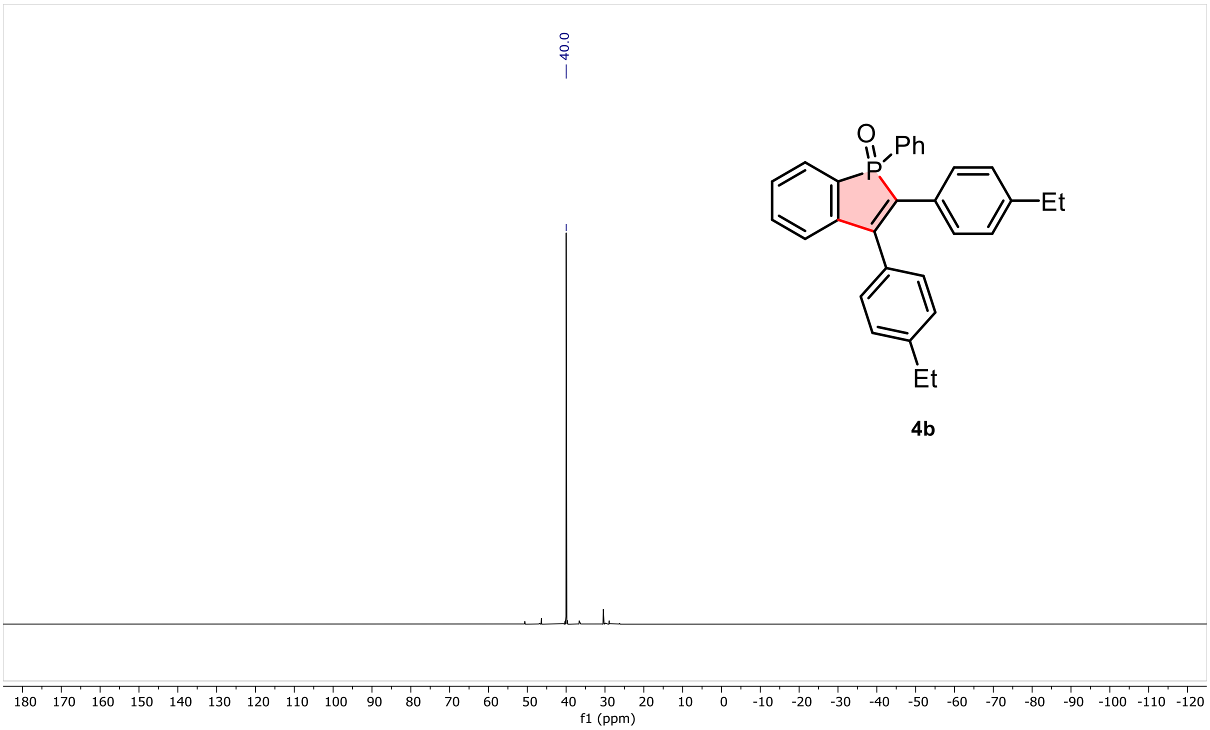

^{1}H NMR (400 MHz, $CDCl_3$) of compound **4c**

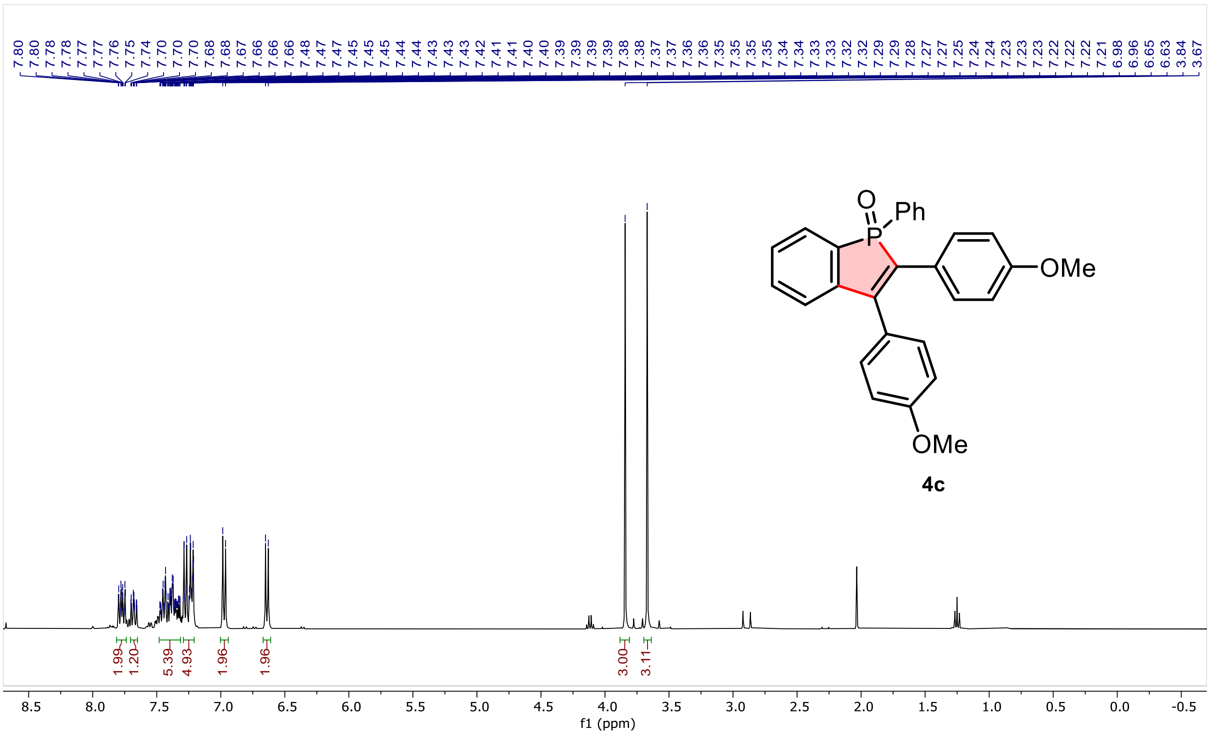

^{13}C NMR (101 MHz, $CDCl_3$) of compound **4c**

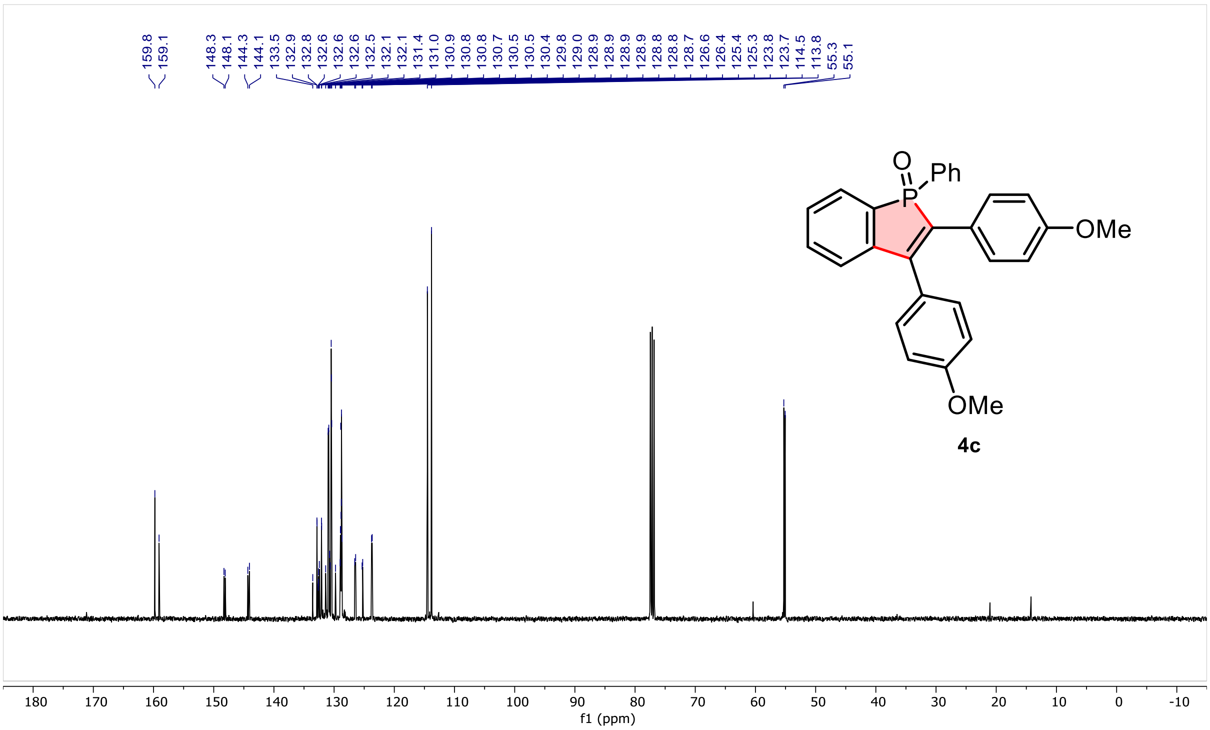

^{31}P NMR (162 MHz, $CDCl_3$) of compound **4c**

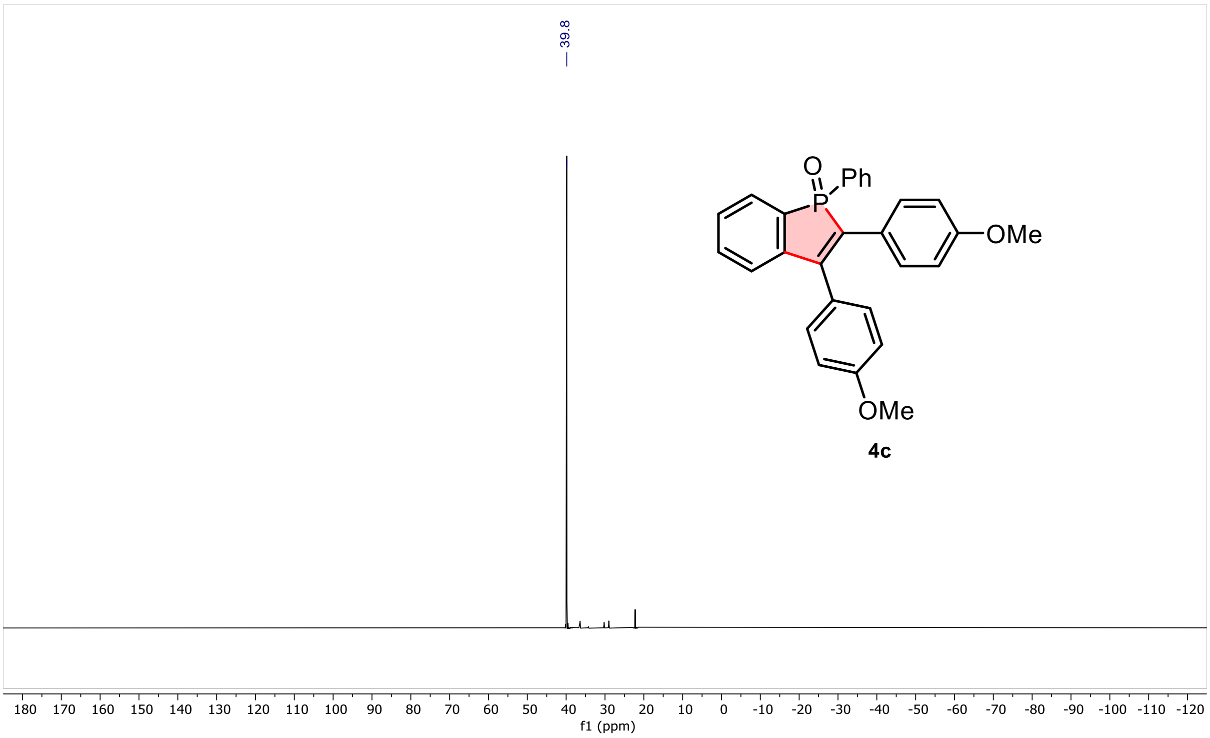

^{1}H NMR (400 MHz, $CDCl_3$) of compound **4d**

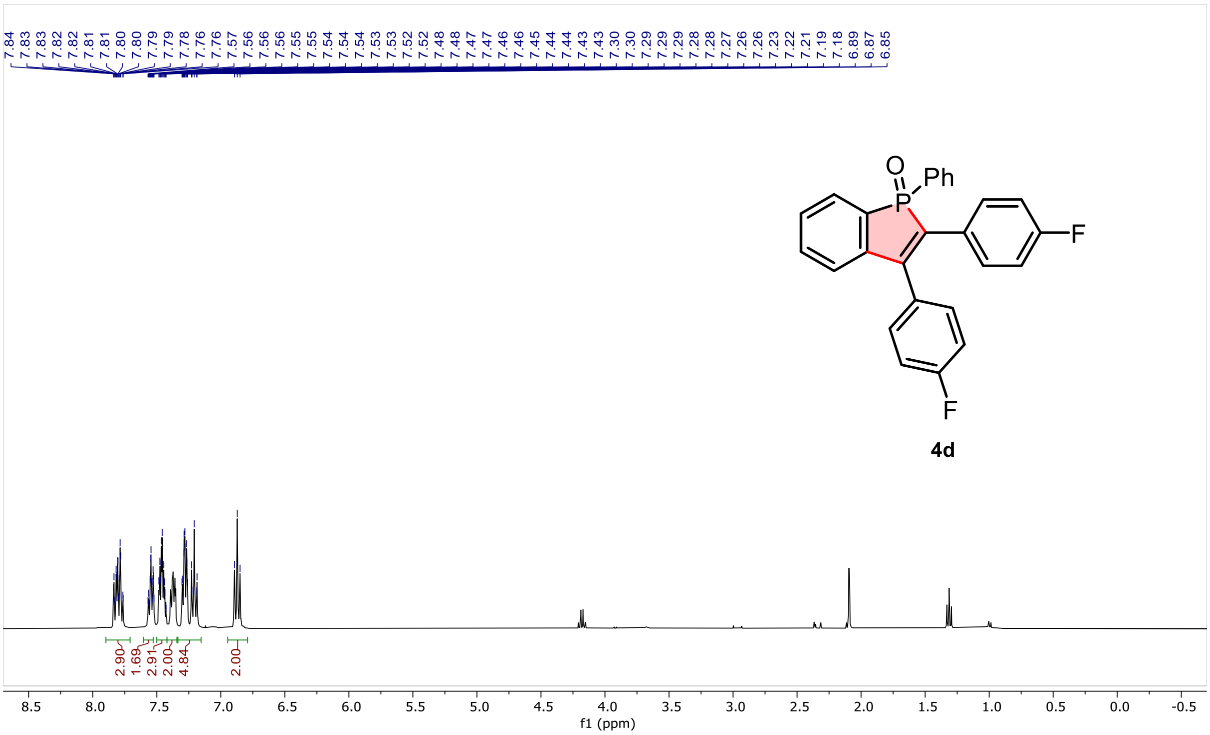

^{13}C NMR (101 MHz, $CDCl_3$) of compound **4d**

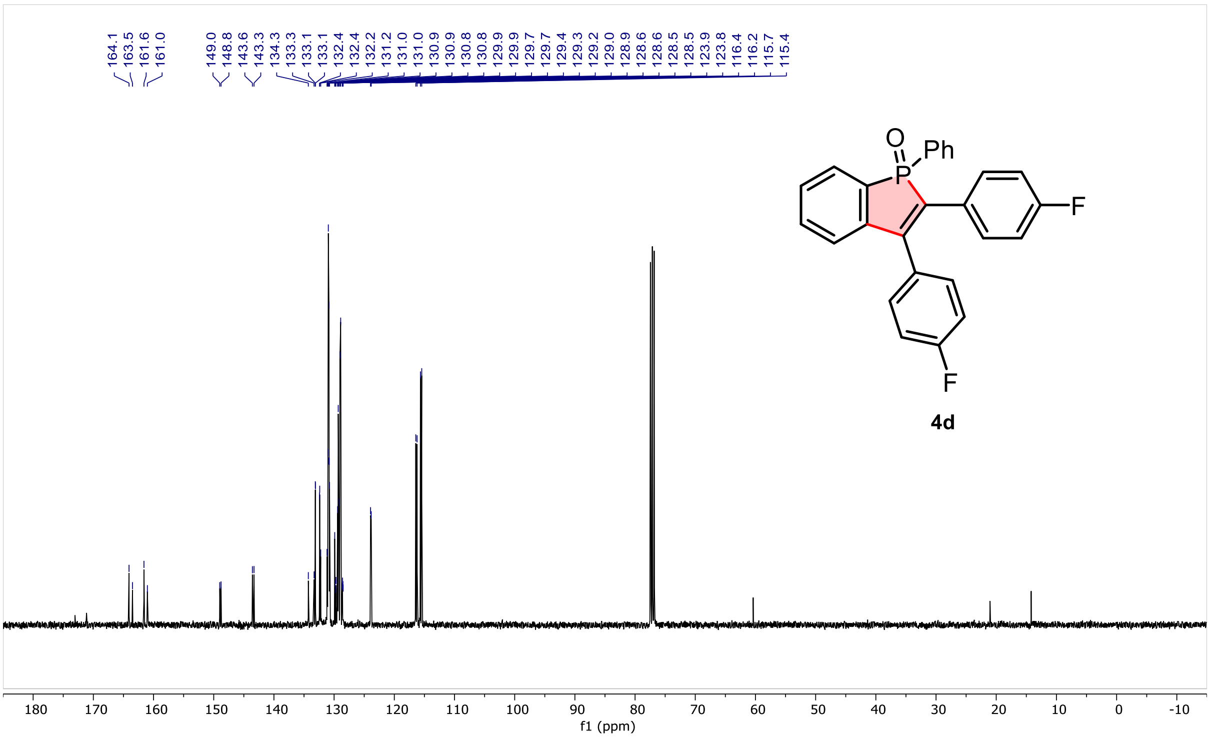

^{31}P NMR (162 MHz, CDCl$_3$) of compound **4d**

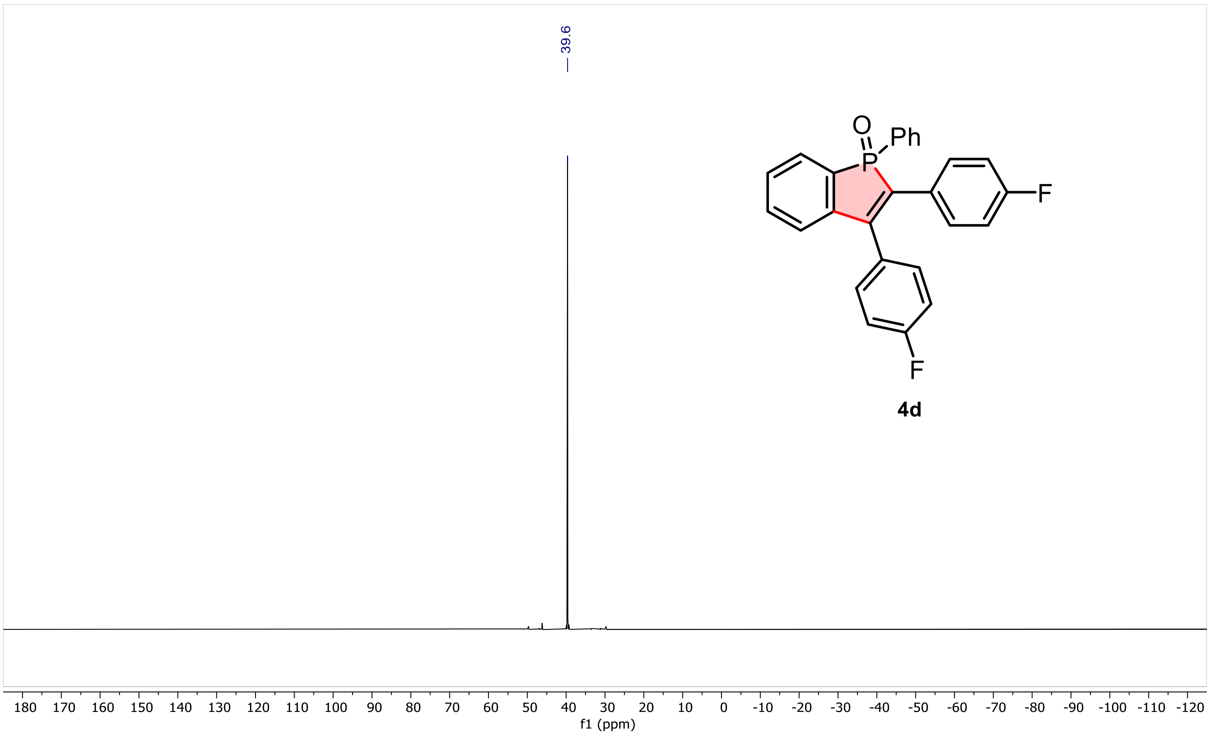

^{19}F NMR (376 MHz, CDCl$_3$) of compound **4d**

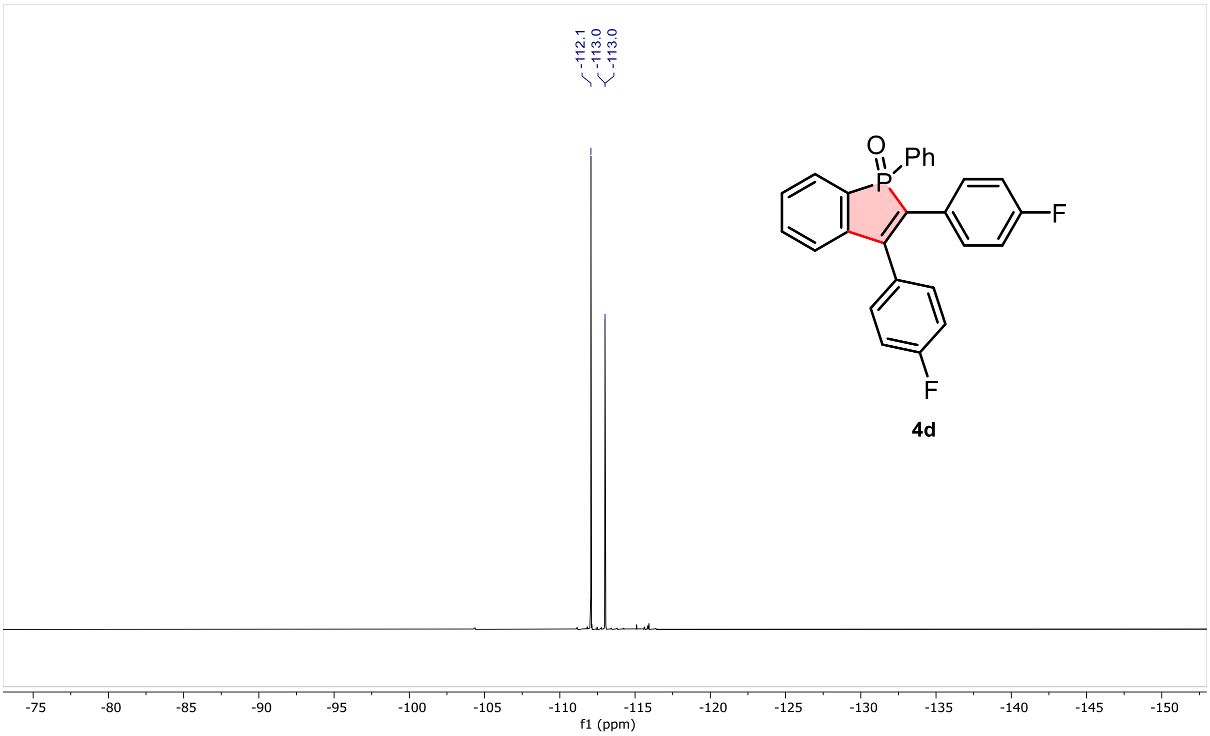

^{1}H NMR (400 MHz, $CDCl_3$) of compound **4e**

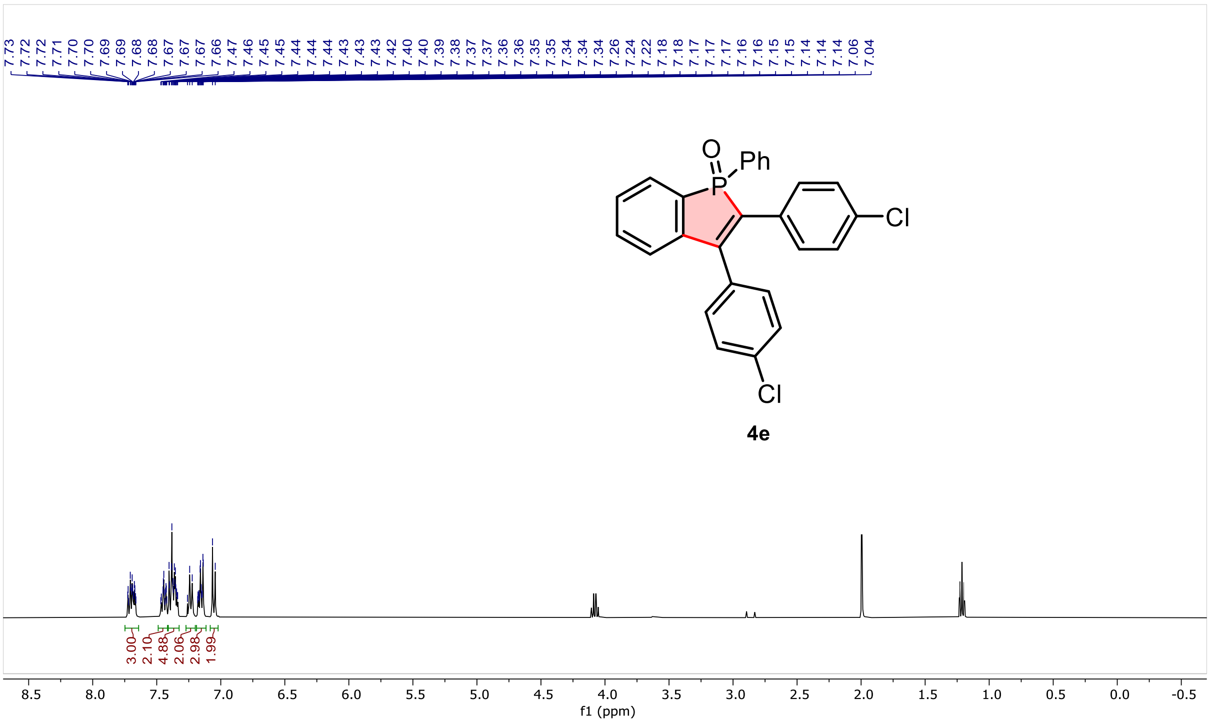

^{13}C NMR (101 MHz, $CDCl_3$) of compound **4e**

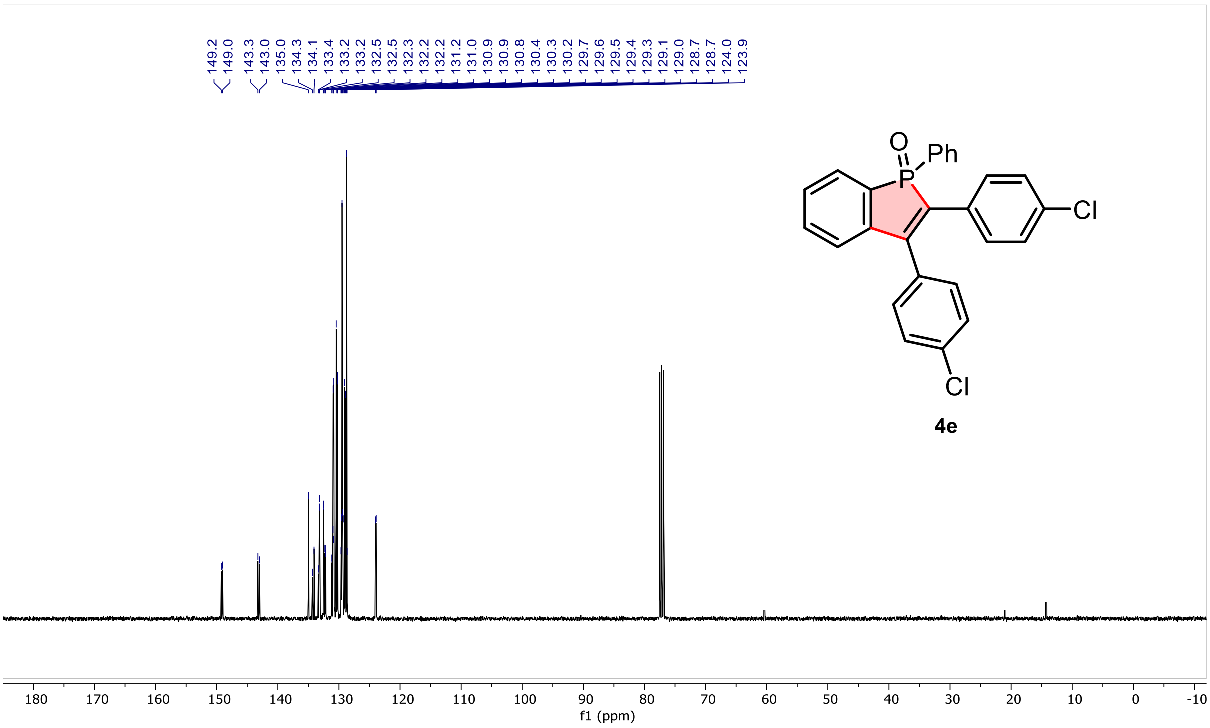

^{31}P NMR (162 MHz, $CDCl_3$) of compound **4e**

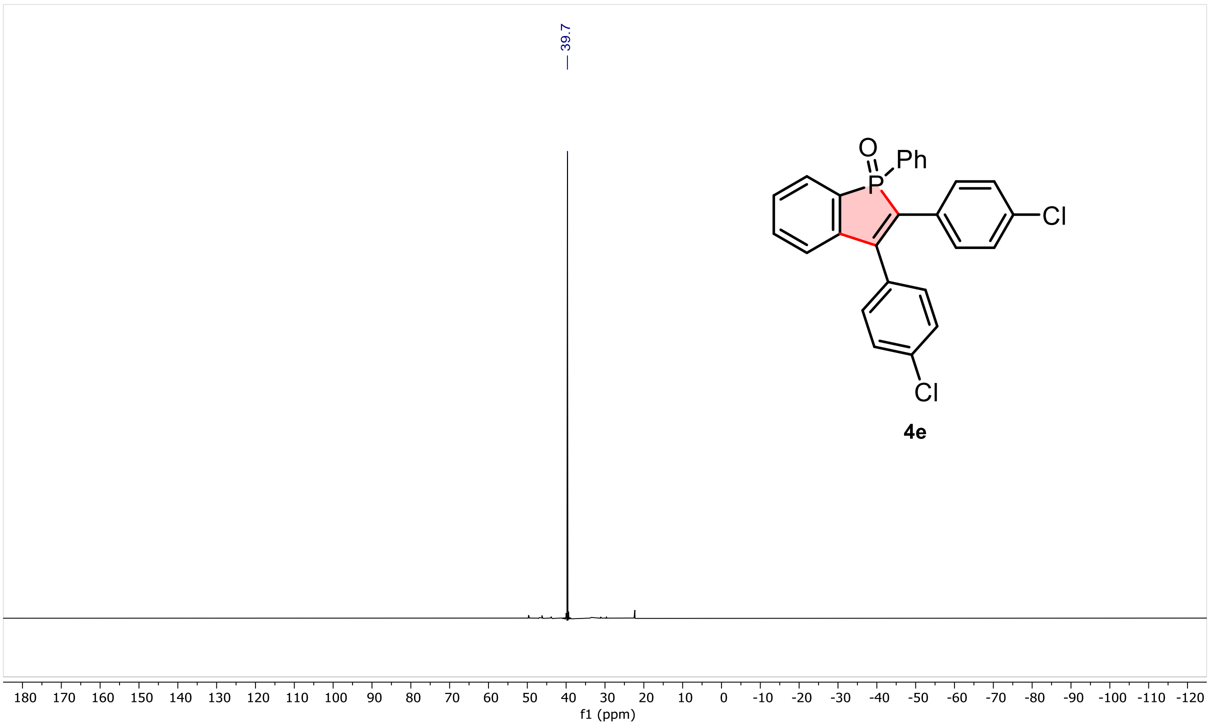

^{1}H NMR (400 MHz, $CDCl_3$) of compound **4f**

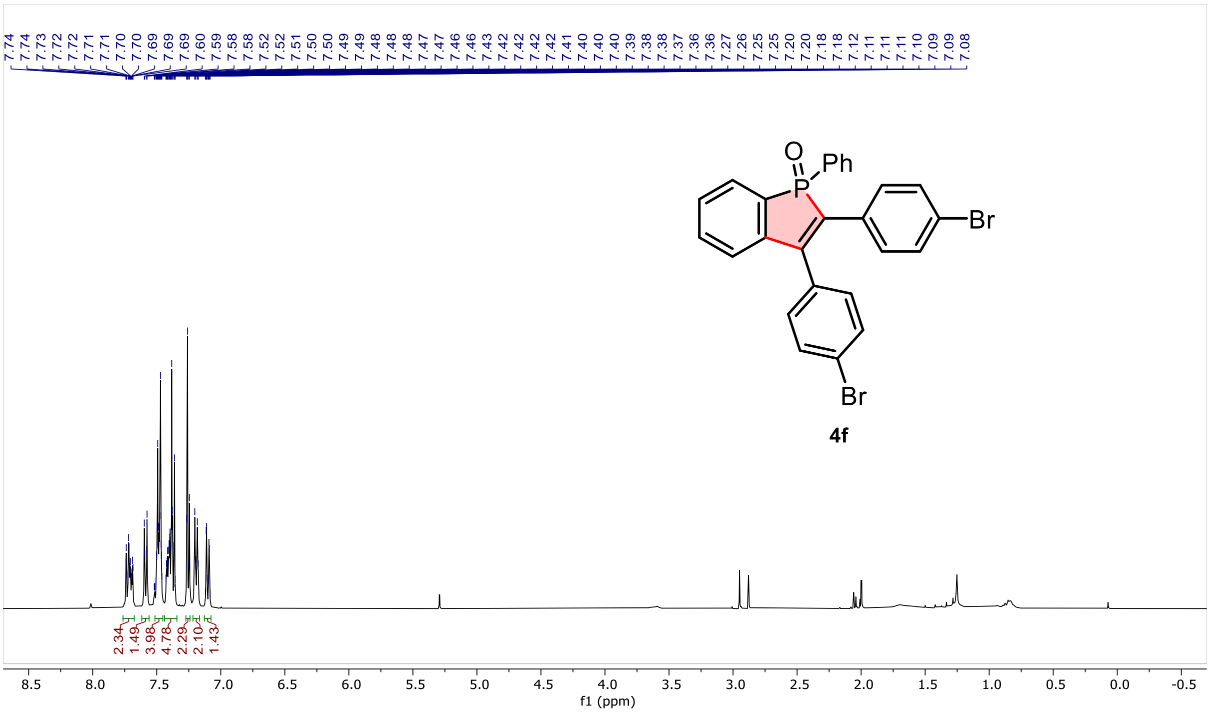

^{13}C NMR (101 MHz, $CDCl_3$) of compound **4f**

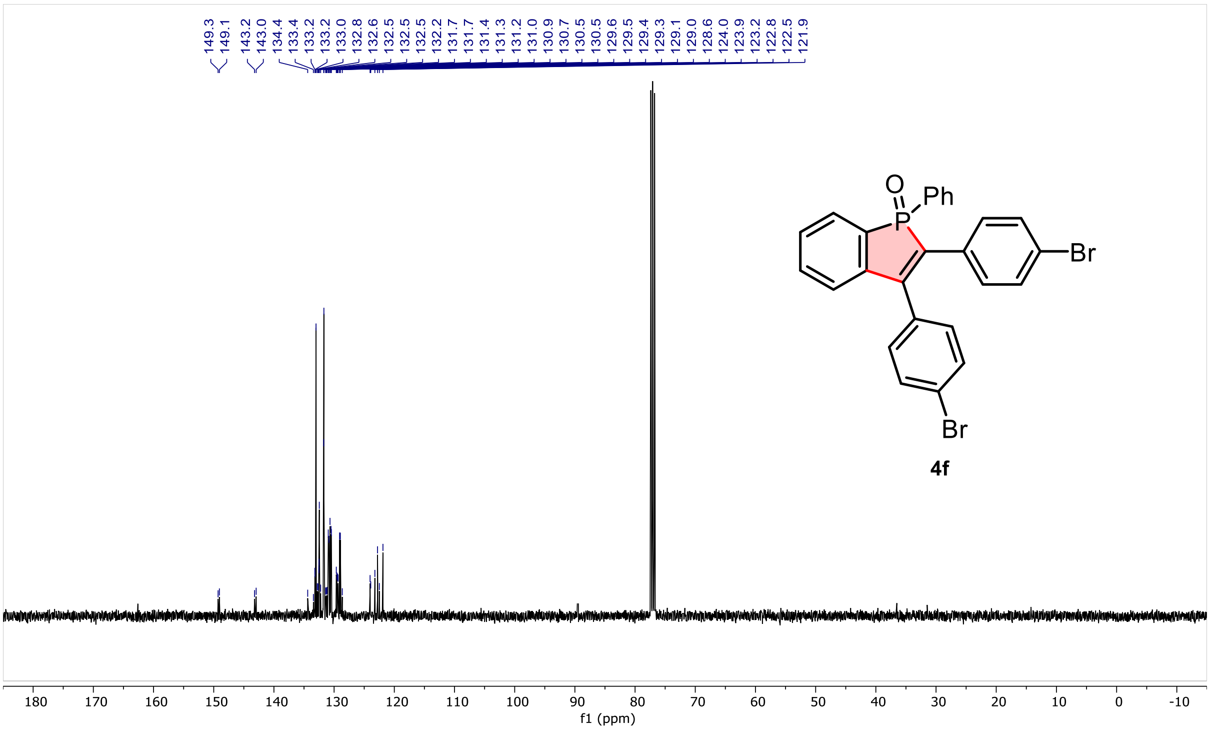

^{31}P NMR (162 MHz, $CDCl_3$) of compound **4f**

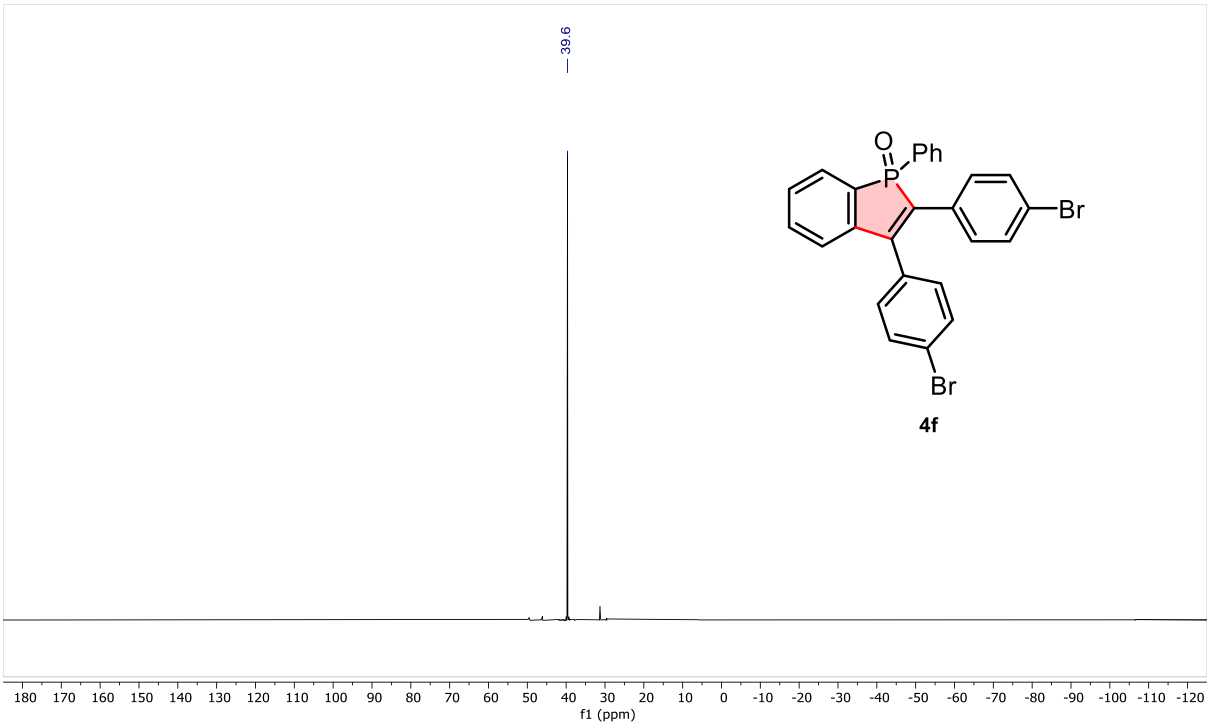

^{1}H NMR (400 MHz, $CDCl_3$) of compound **4g**

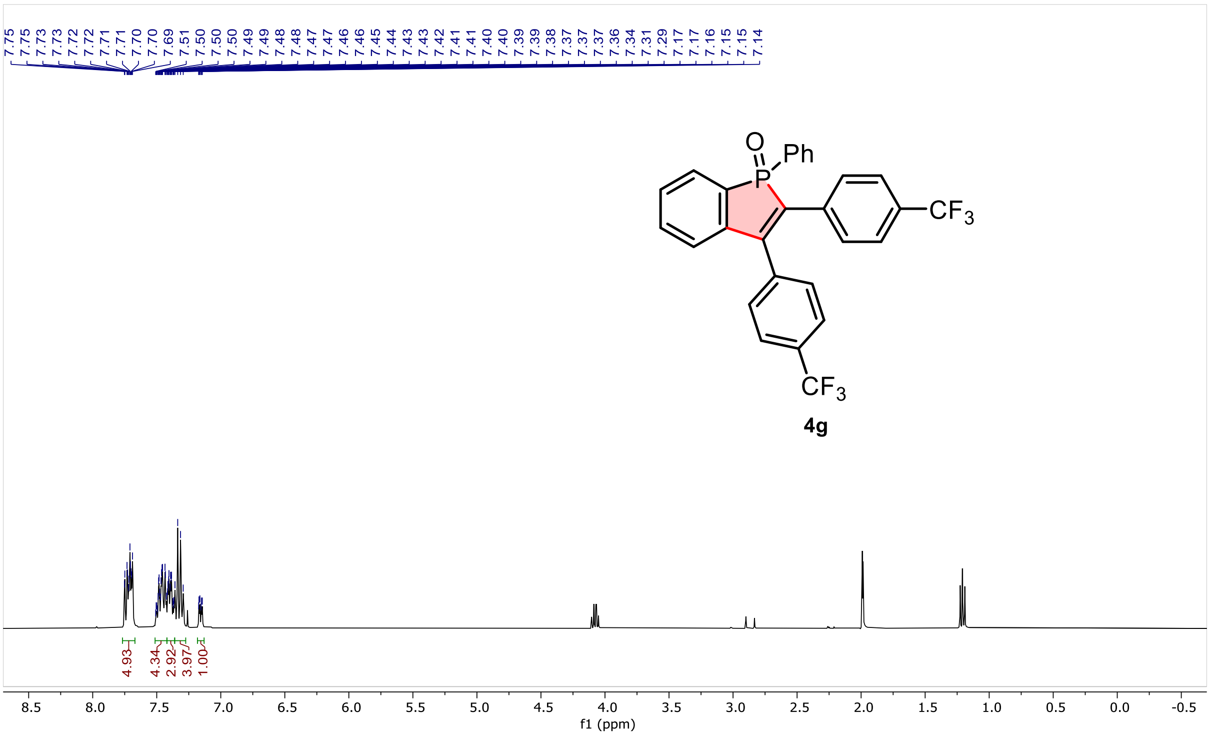

^{13}C NMR (101 MHz, $CDCl_3$) of compound **4g**

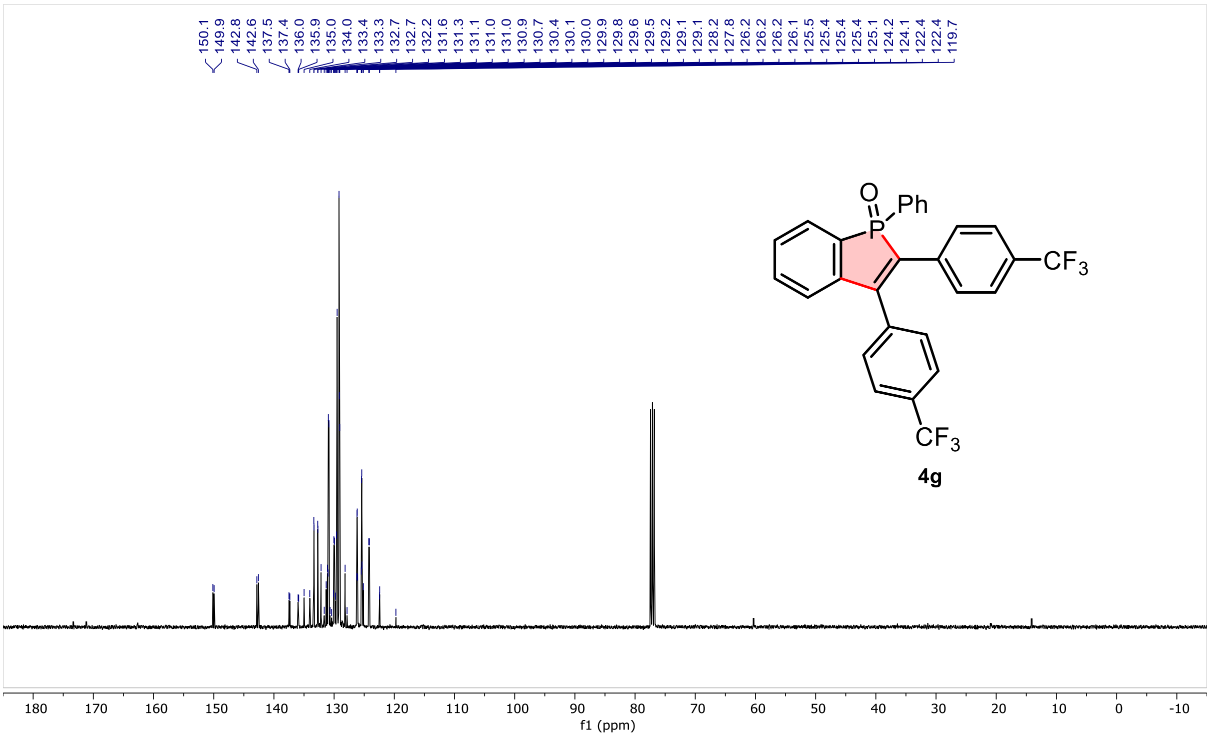

^{31}P NMR (162 MHz, $CDCl_3$) of compound **4g**

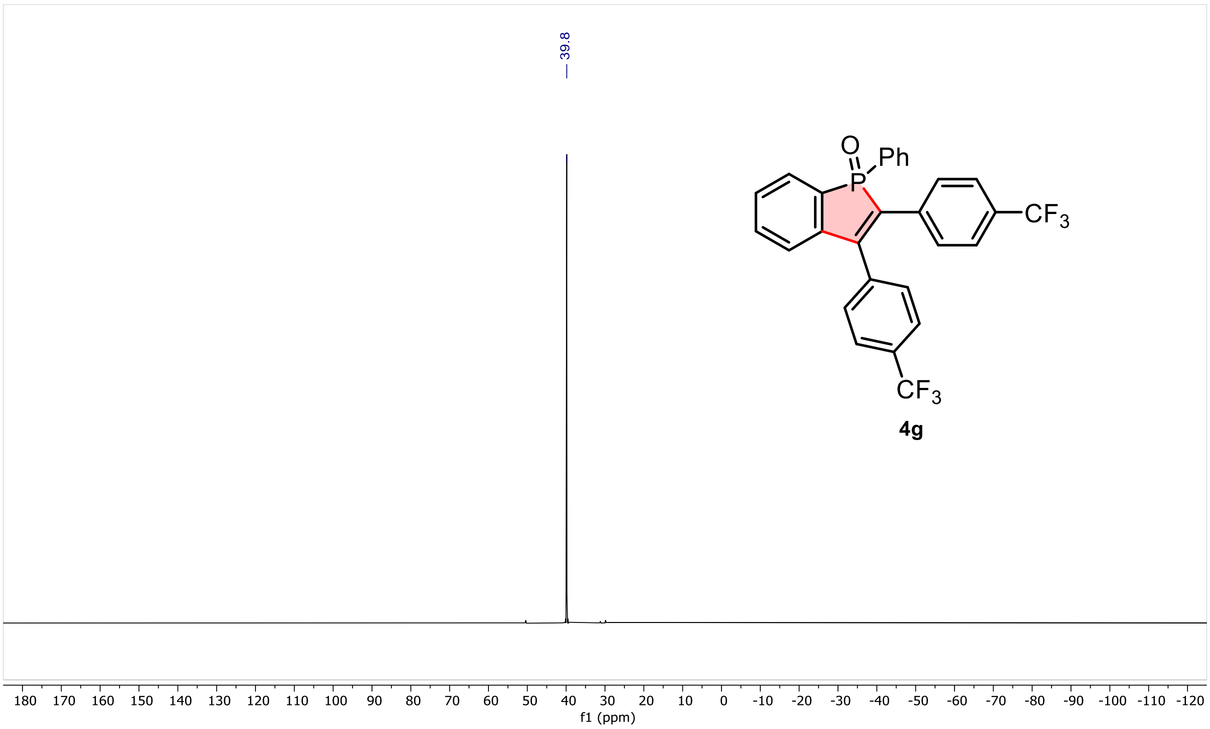

^{19}F NMR (376 MHz, $CDCl_3$) of compound **4g**

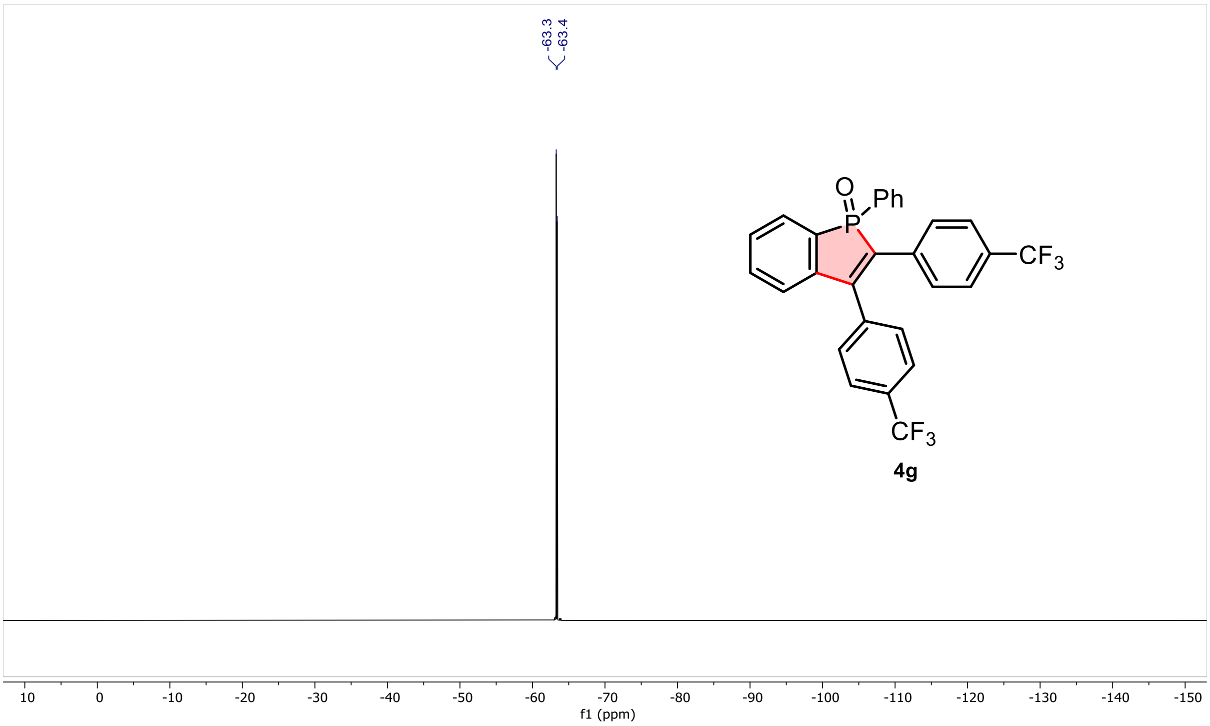

^{1}H NMR (400 MHz, $CDCl_3$) of compound **4h**

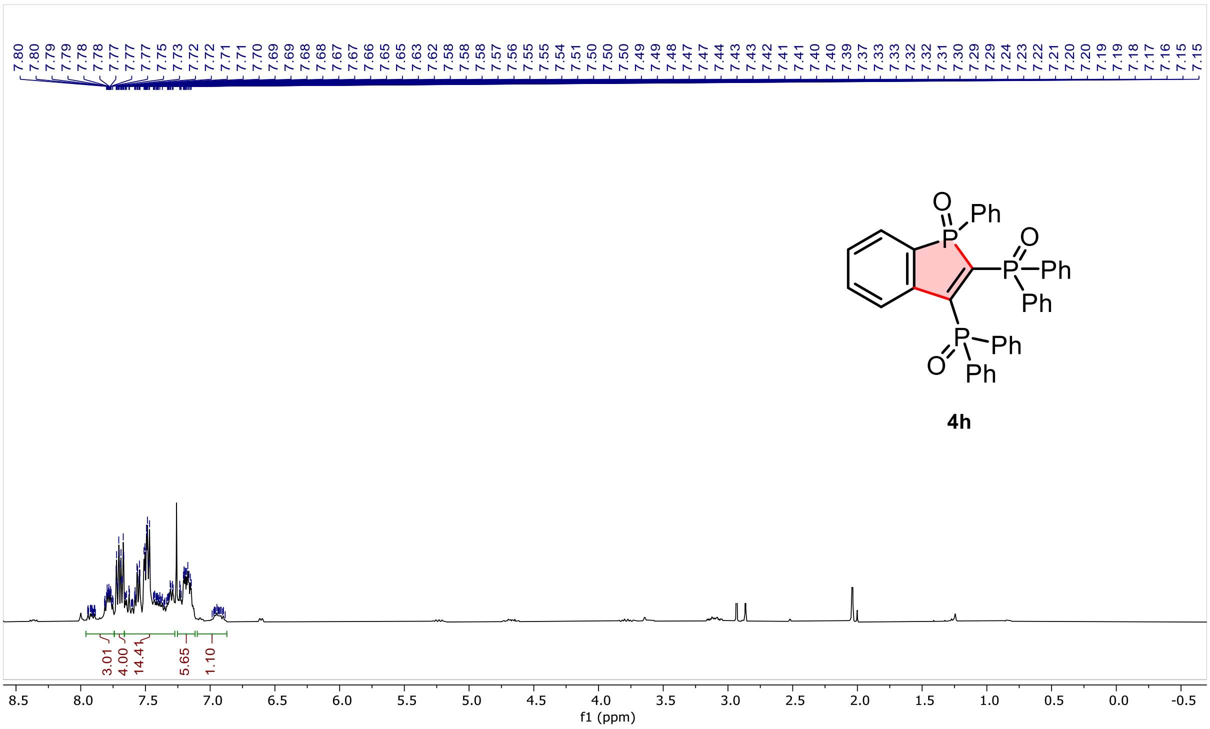

^{13}C NMR (101 MHz, $CDCl_3$) of compound **4h**

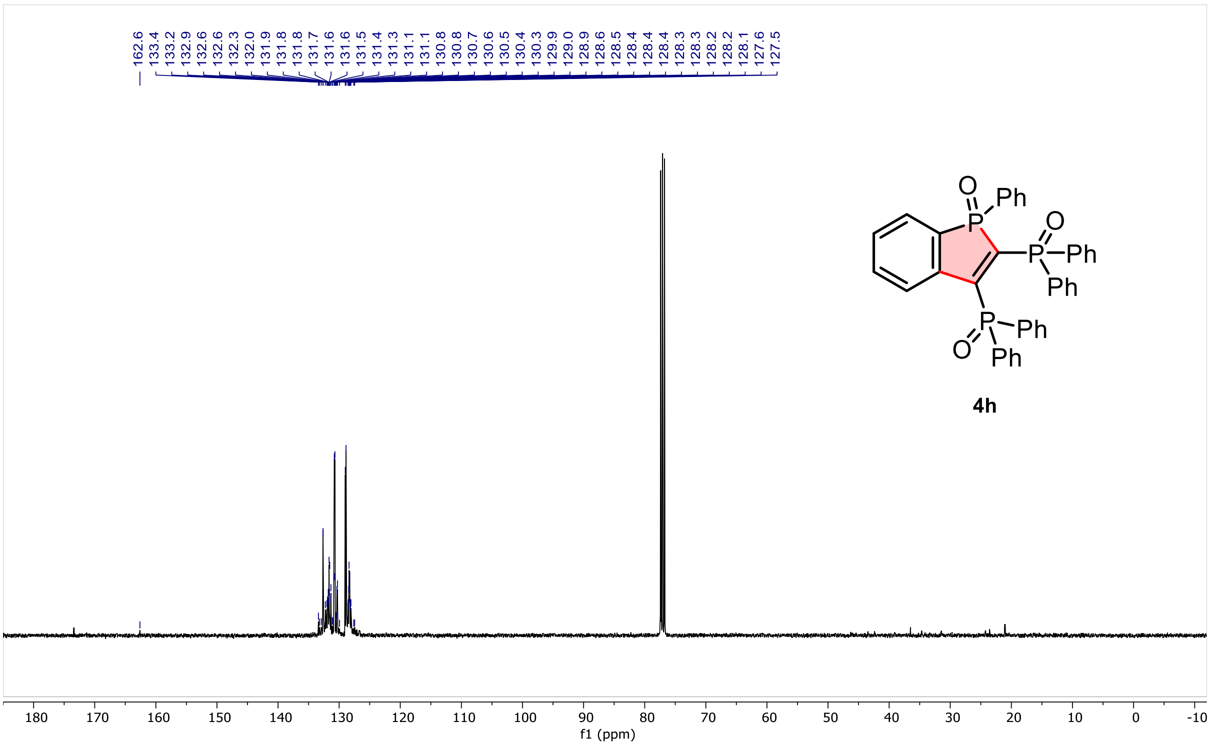

^{31}P NMR (162 MHz, $CDCl_3$) of compound **4h**

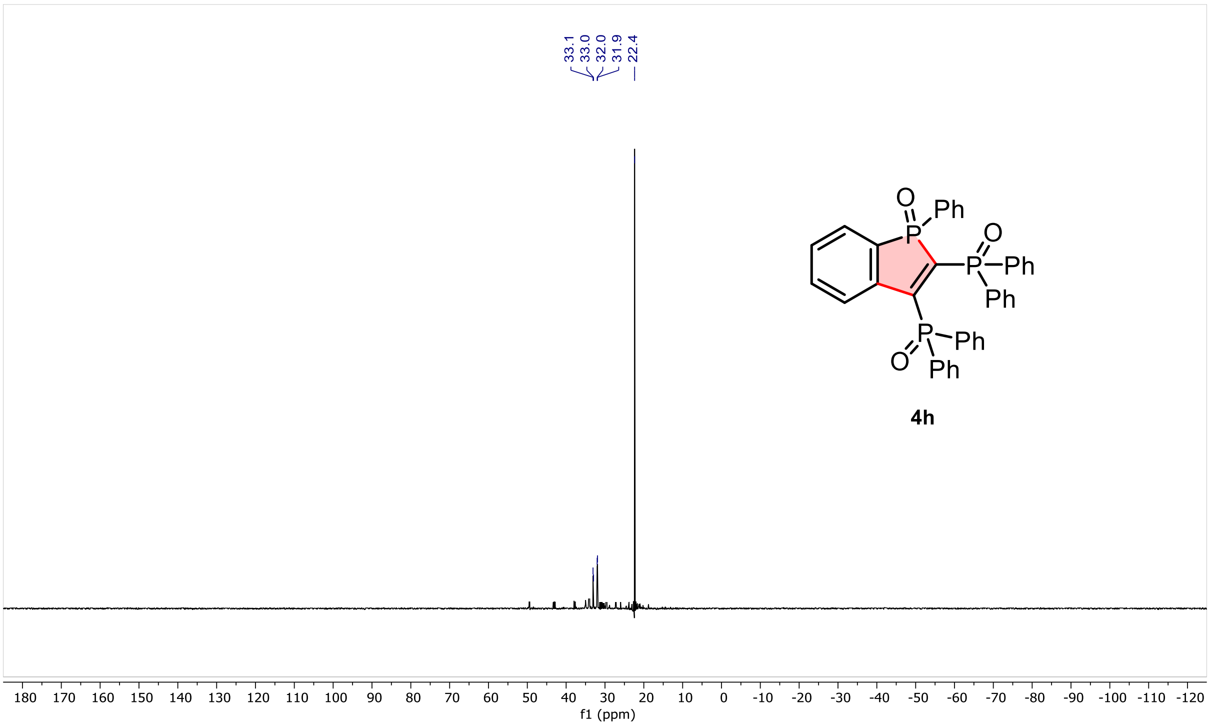

^1H NMR (400 MHz, $CDCl_3$) of compound **4i**

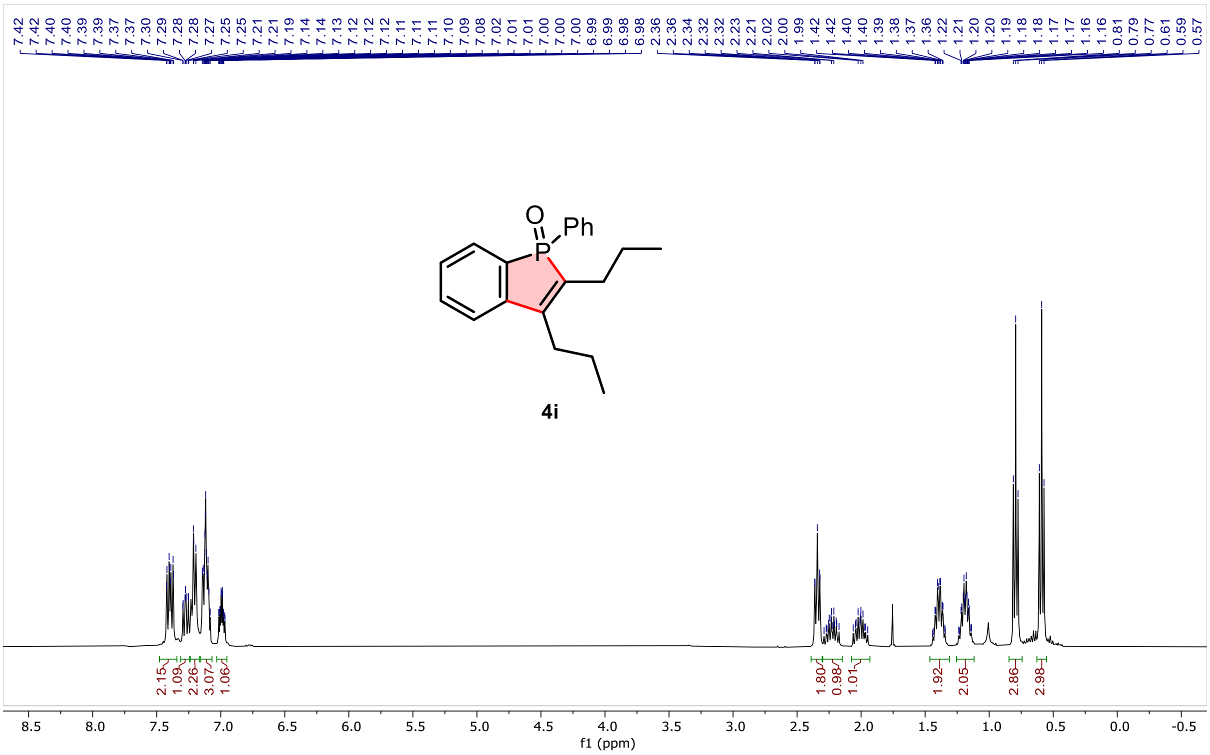

^{13}C NMR (101 MHz, $CDCl_3$) of compound **4i**

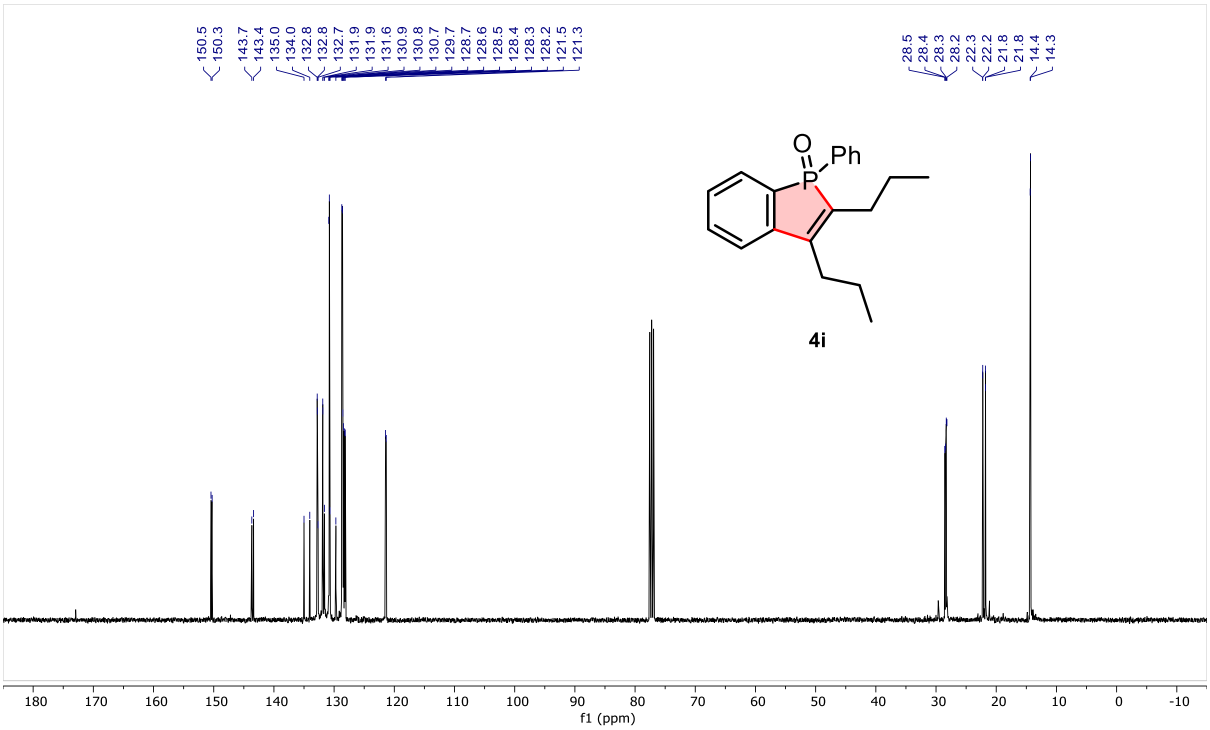

^{31}P NMR (162 MHz, $CDCl_3$) of compound **4i**

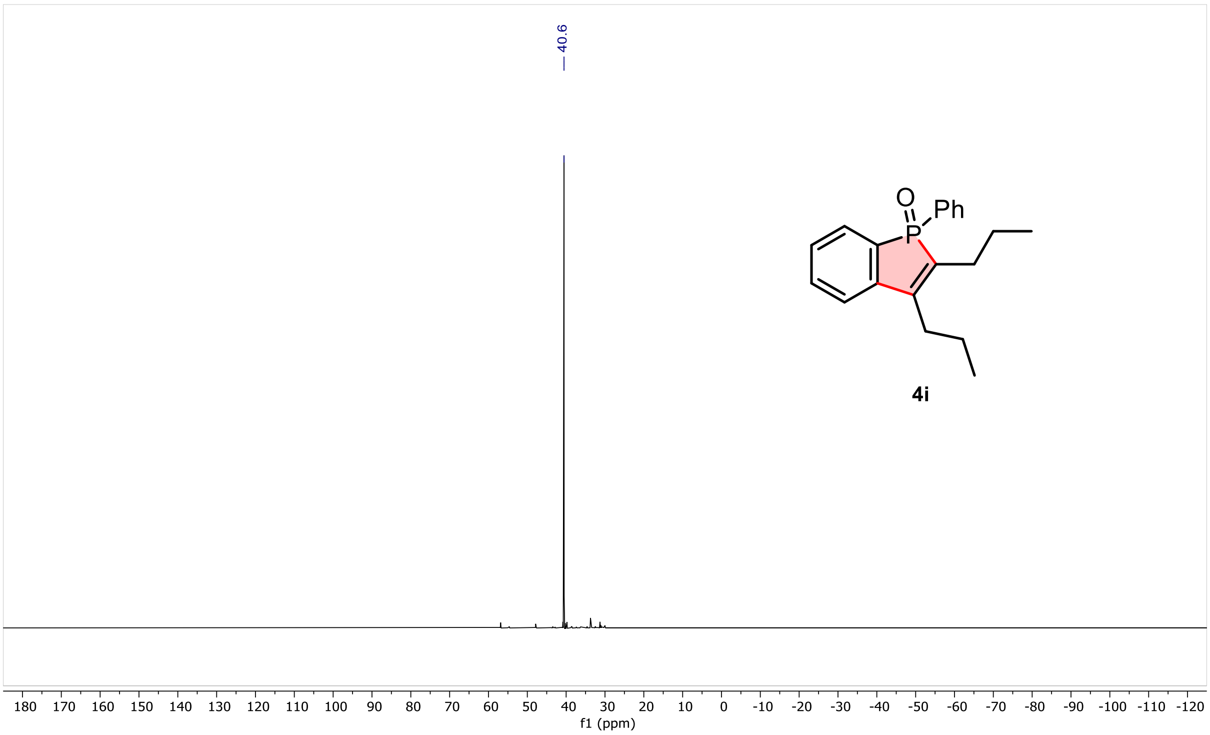

^{1}H NMR (400 MHz, $CDCl_3$) of compound **4j**

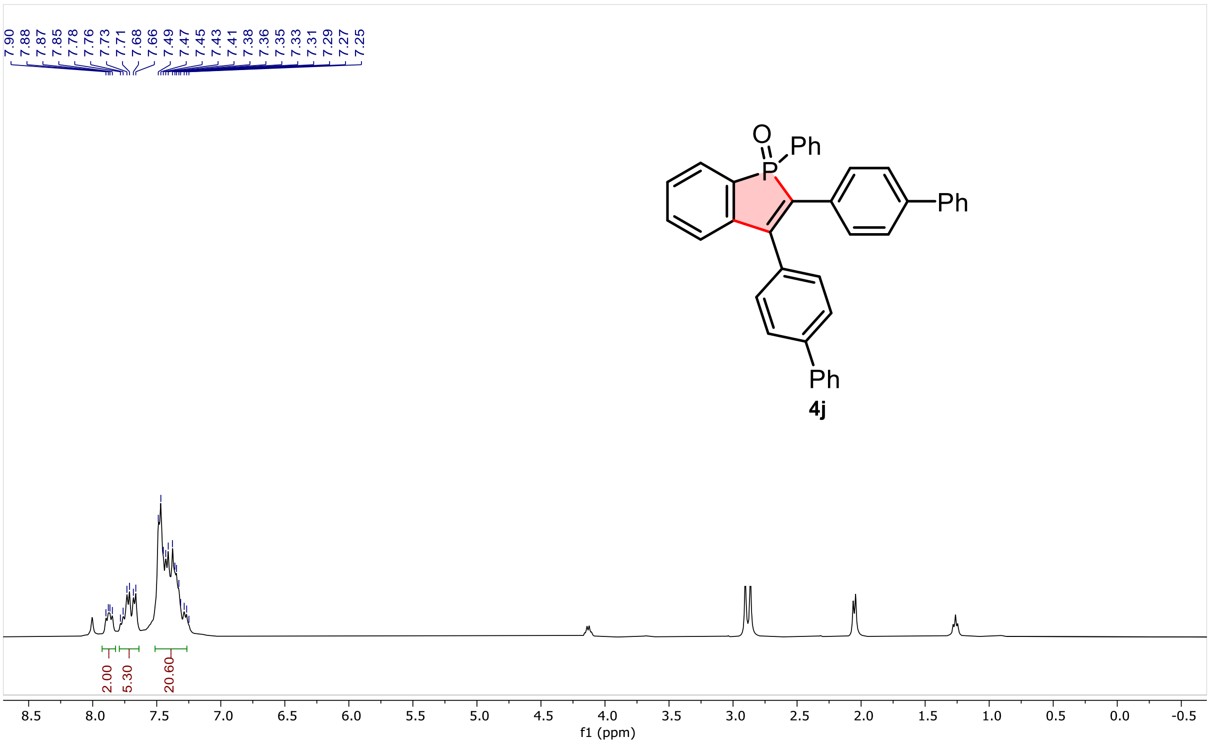

^{13}C NMR (101 MHz, $CDCl_3$) of compound **4j**

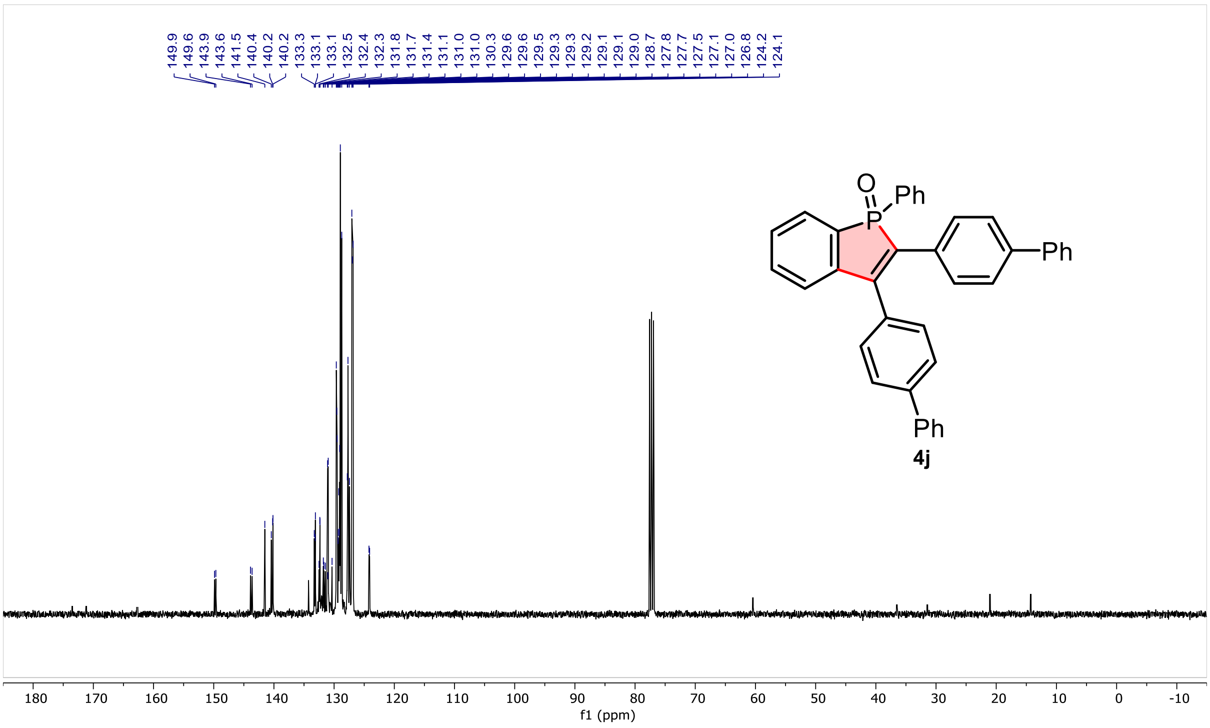

^{31}P NMR (162 MHz, $CDCl_3$) of compound **4j**

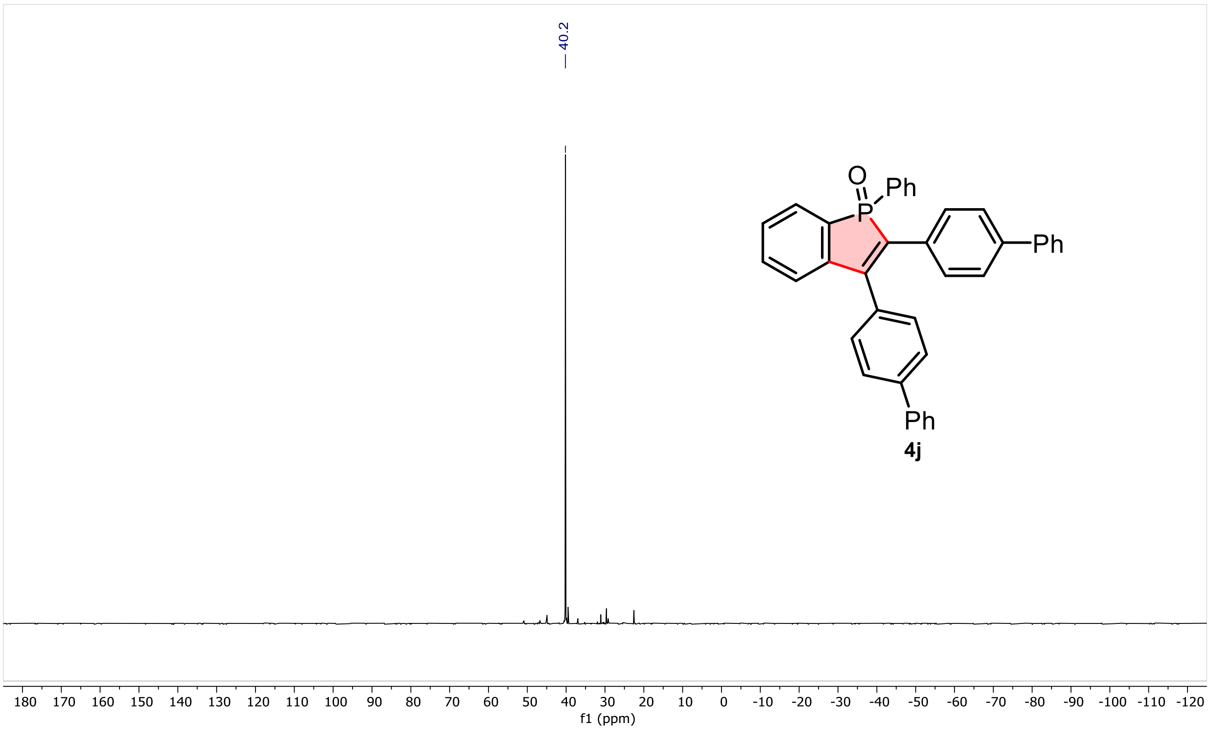

^{1}H NMR (400 MHz, $CDCl_3$) of compound **4k**

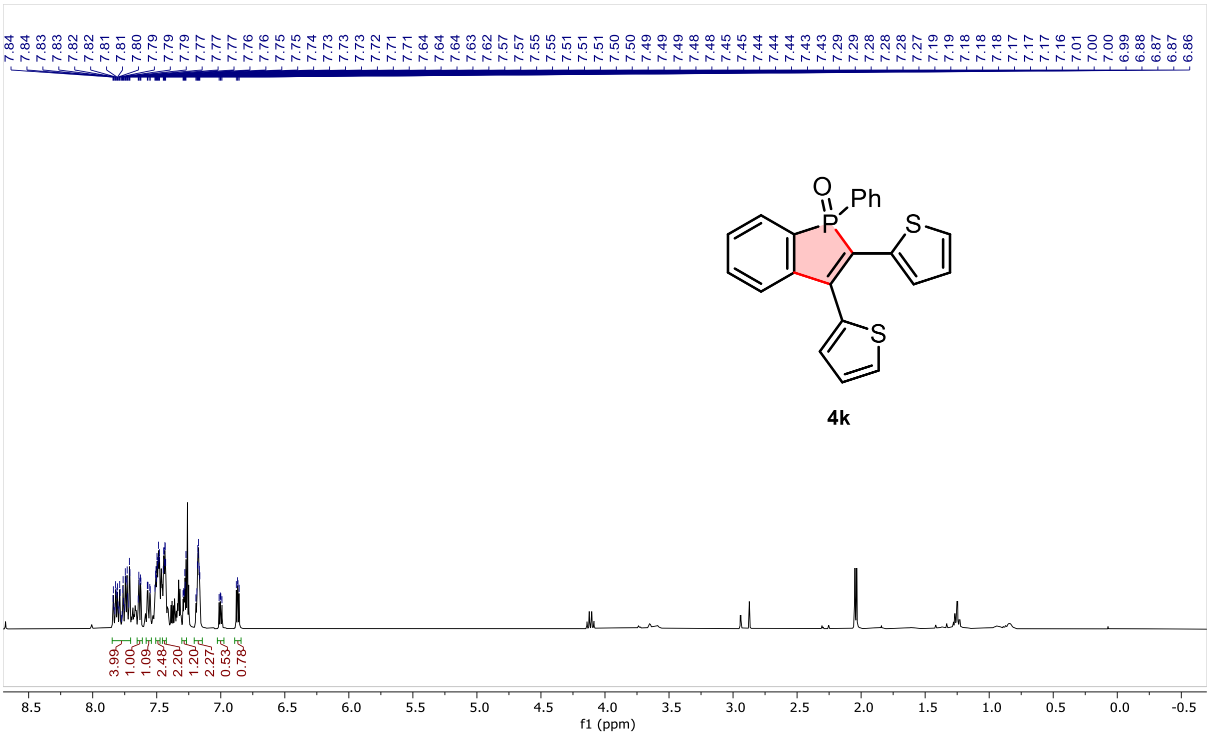

^{13}C NMR (101 MHz, $CDCl_3$) of compound **4k**

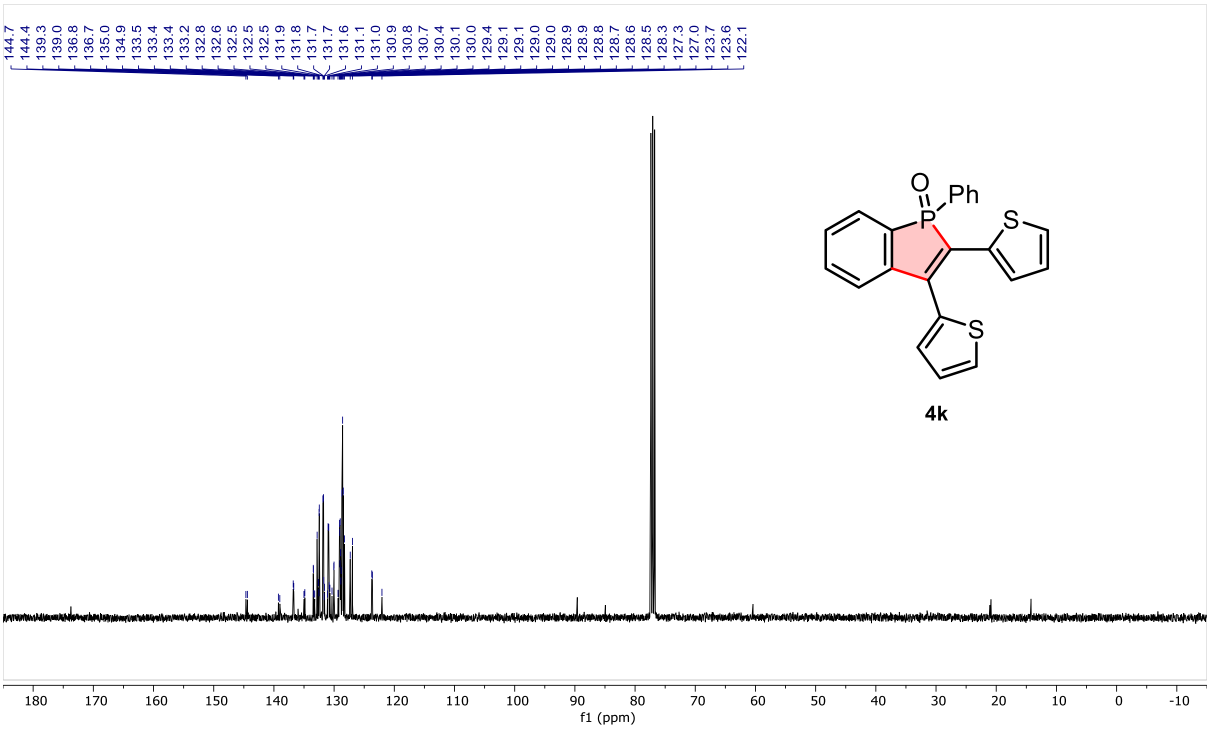

^{31}P NMR (162 MHz, $CDCl_3$) of compound **4k**

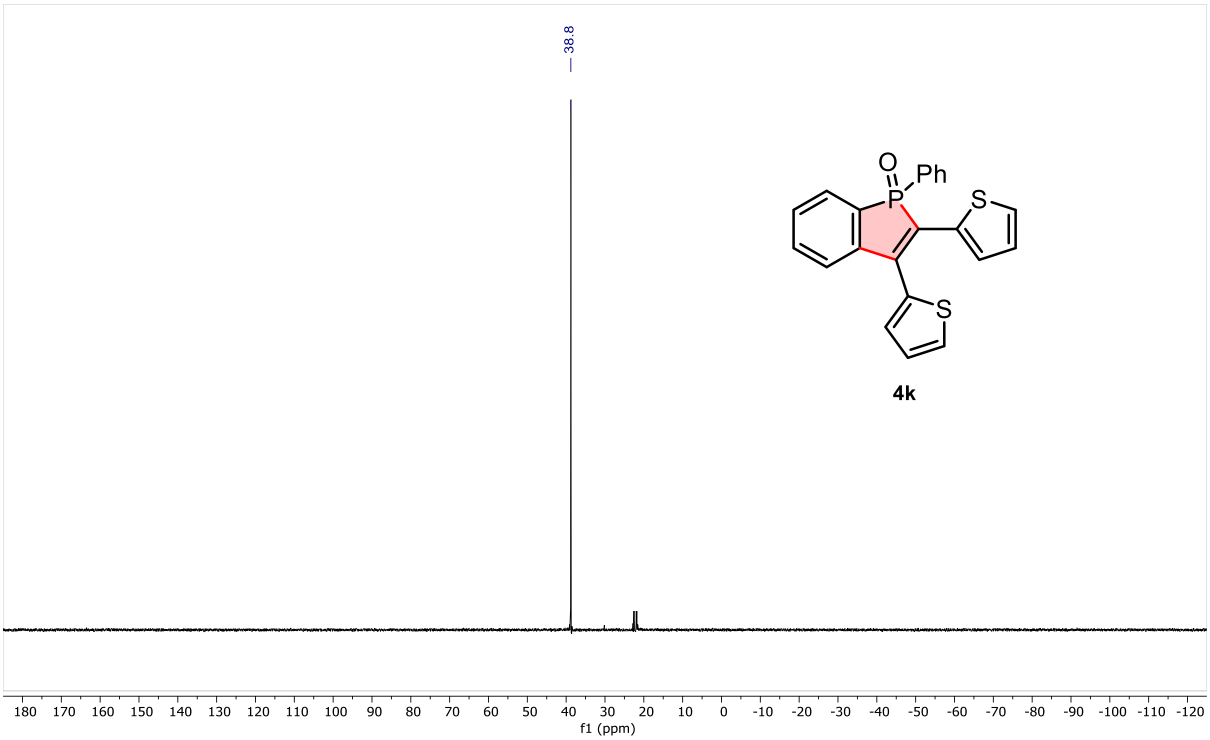

^{1}H NMR (400 MHz, $CDCl_3$) of compound **4l, 4l'**

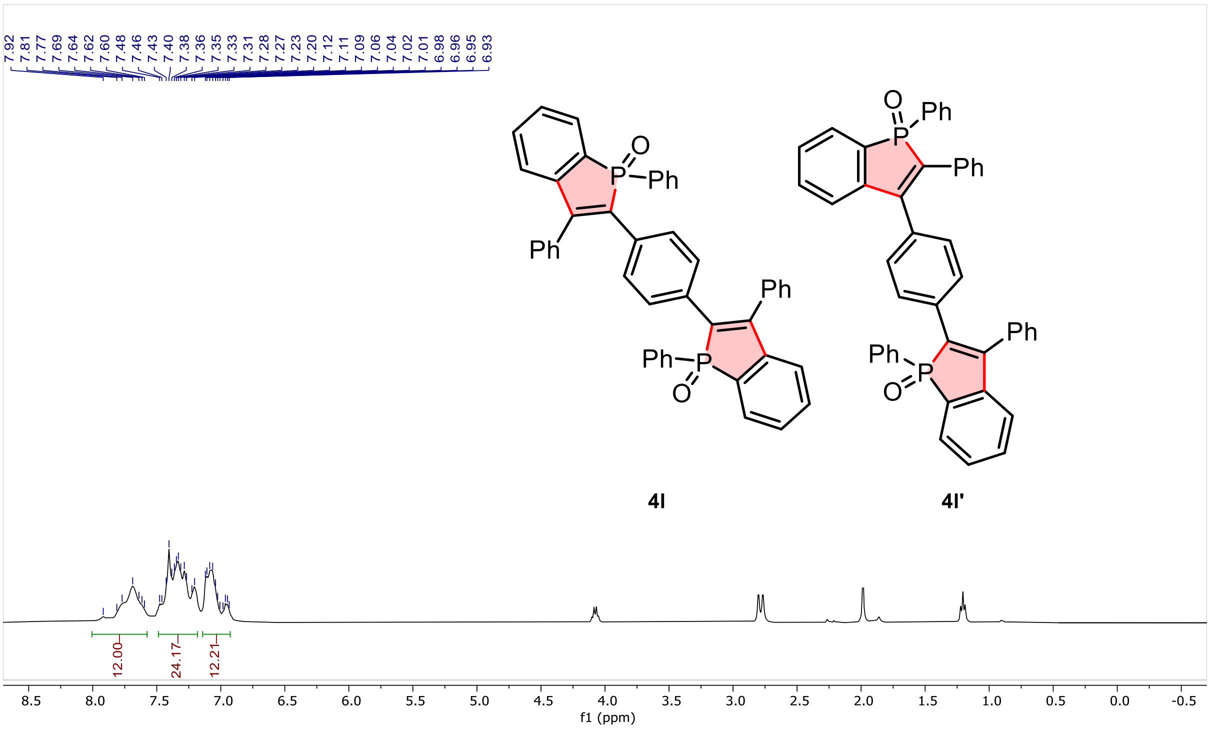

^{13}C NMR (101 MHz, $CDCl_3$) of compound **4l, 4l'**

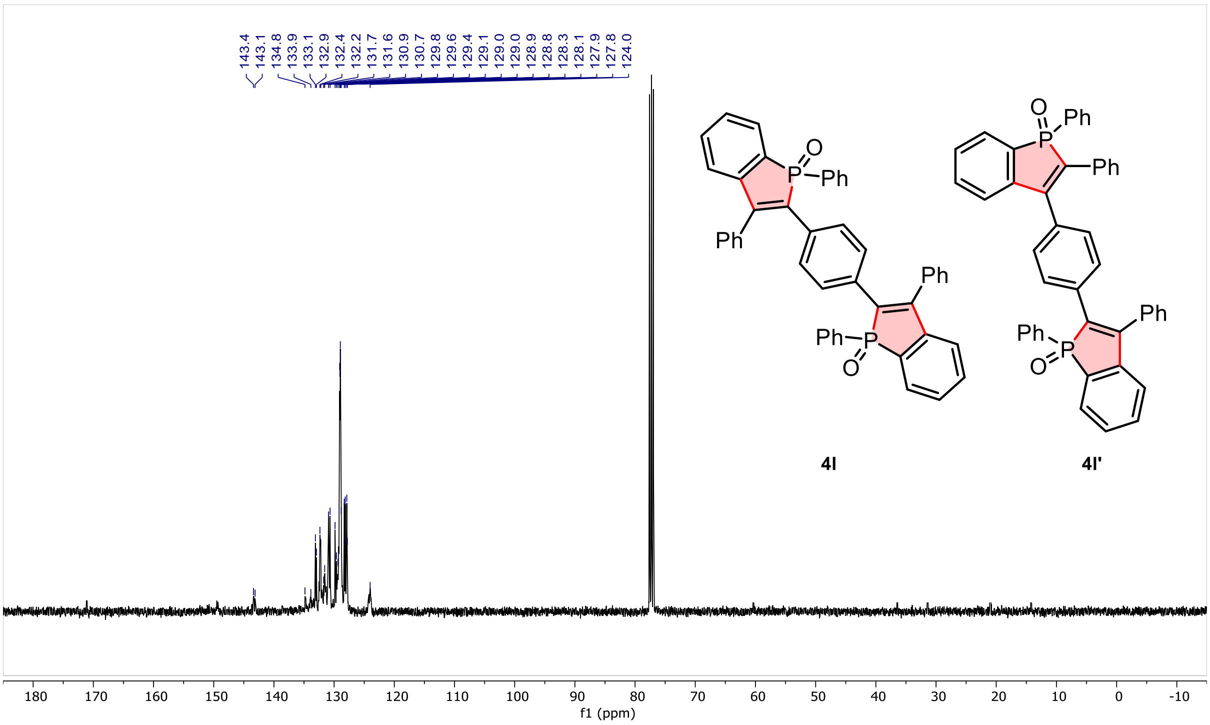

^{31}P NMR (162 MHz, CDCl$_3$) of compound **4l, 4l'**

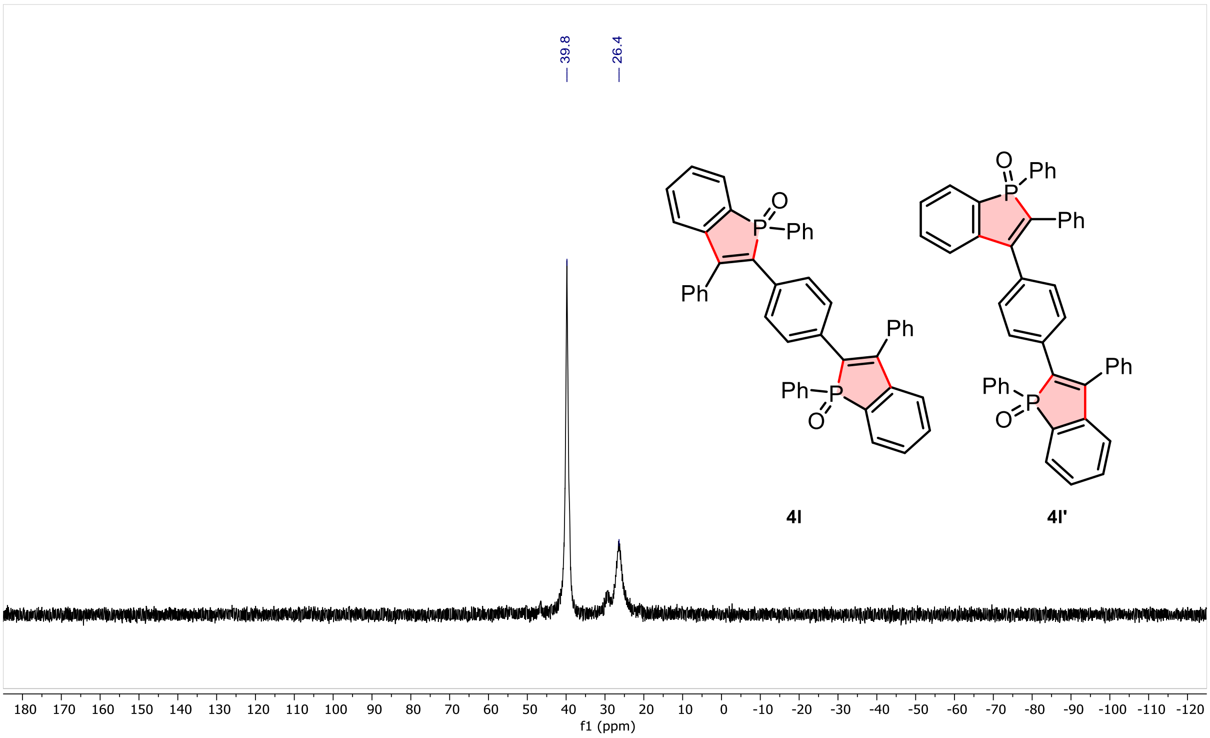

^{1}H NMR (400 MHz, $CDCl_3$) of compound **4m**

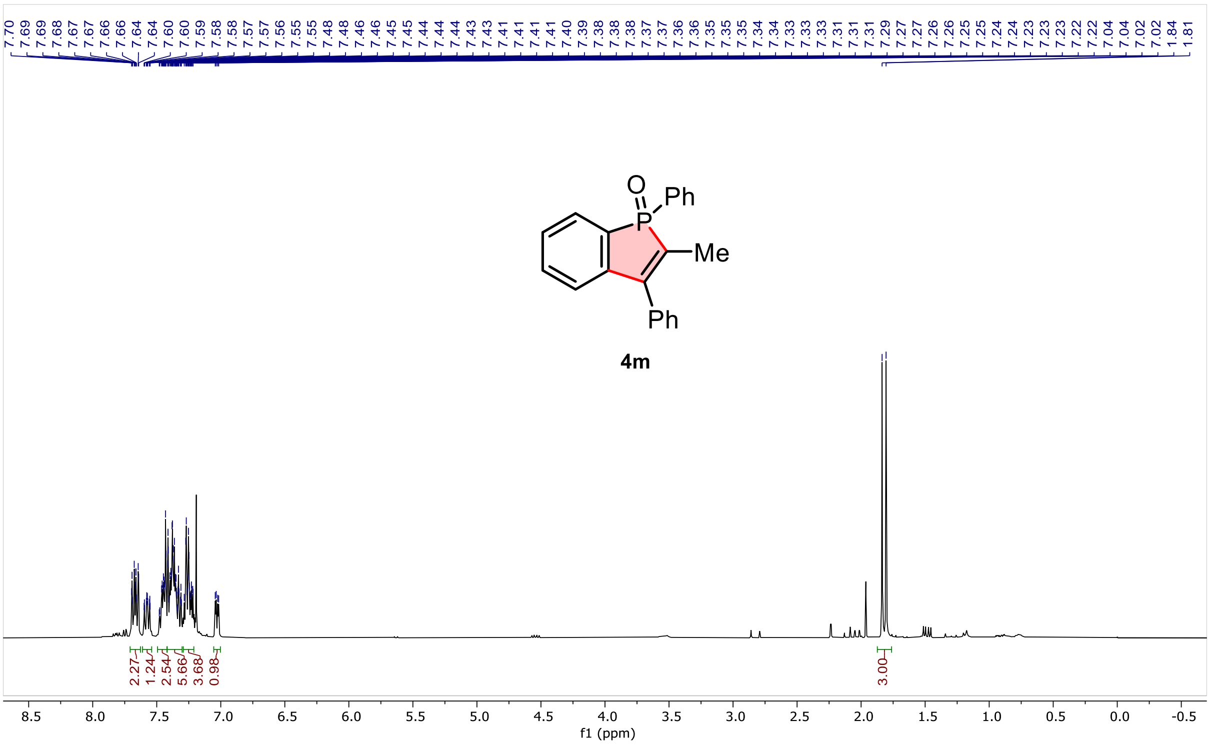

^{13}C NMR (101 MHz, $CDCl_3$) of compound **4m**

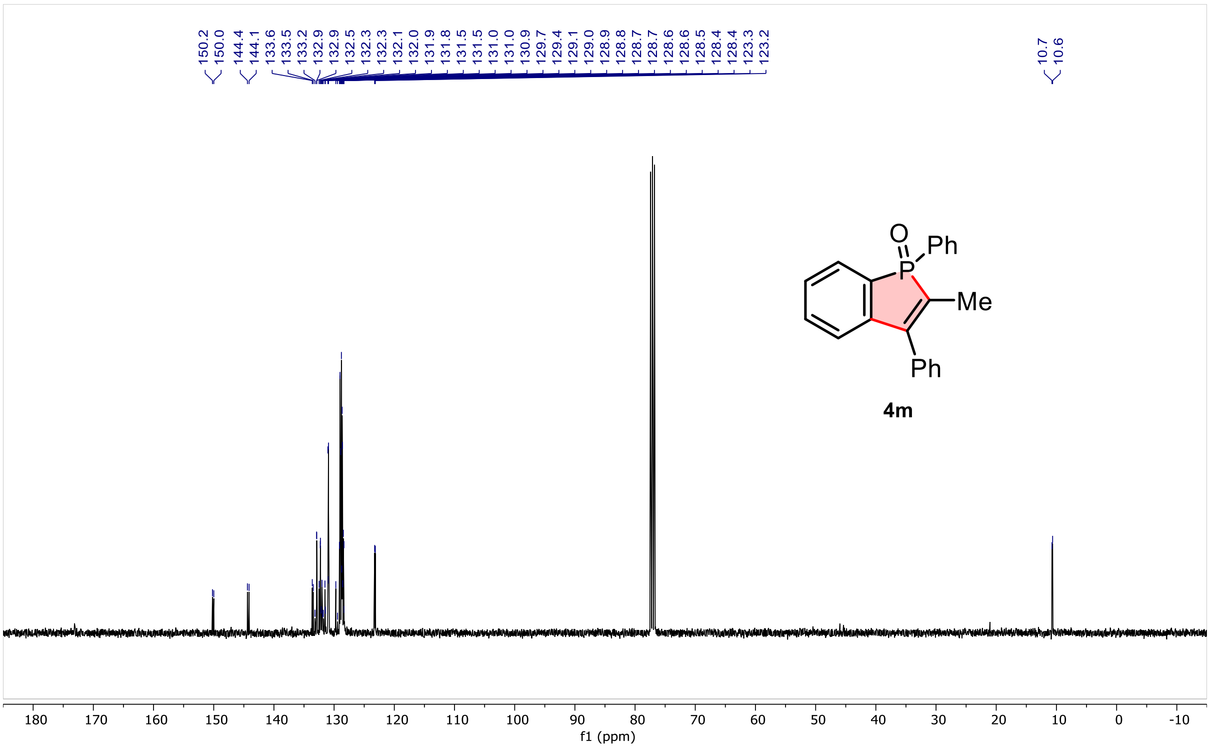

^{31}P NMR (162 MHz, $CDCl_3$) of compound **4m**

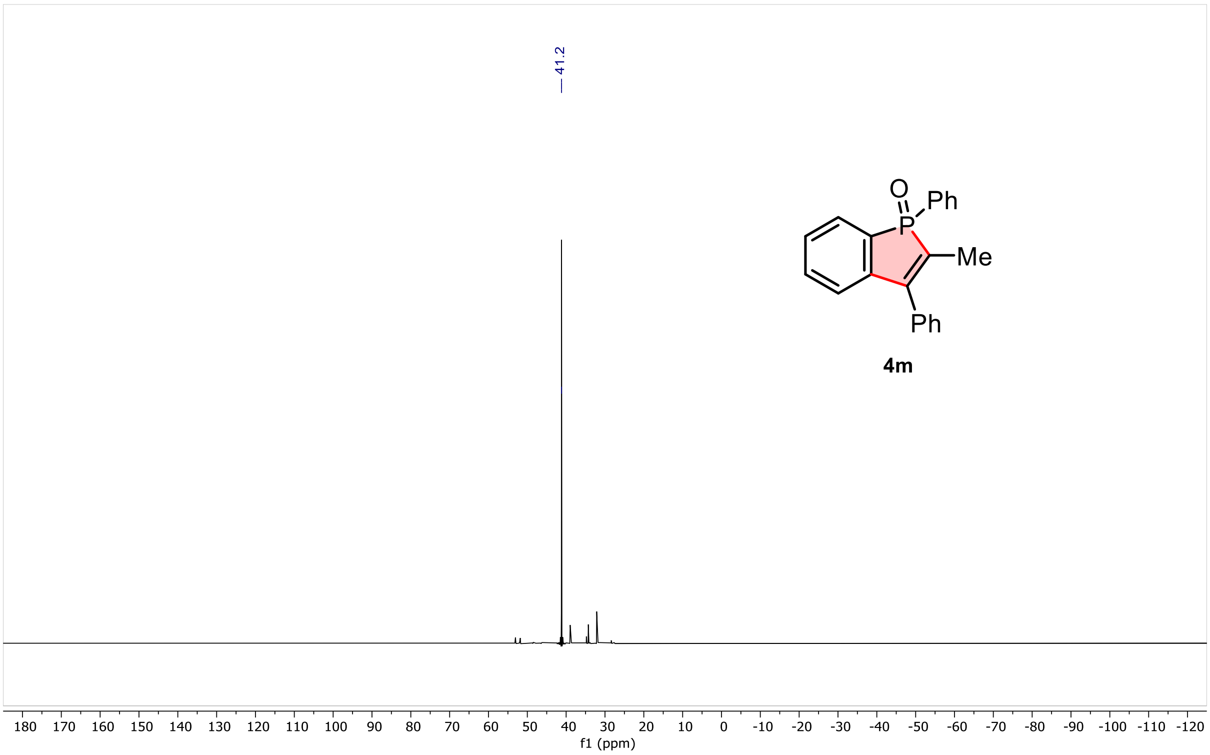

^{1}H NMR (400 MHz, $CDCl_3$) of compound **4n**

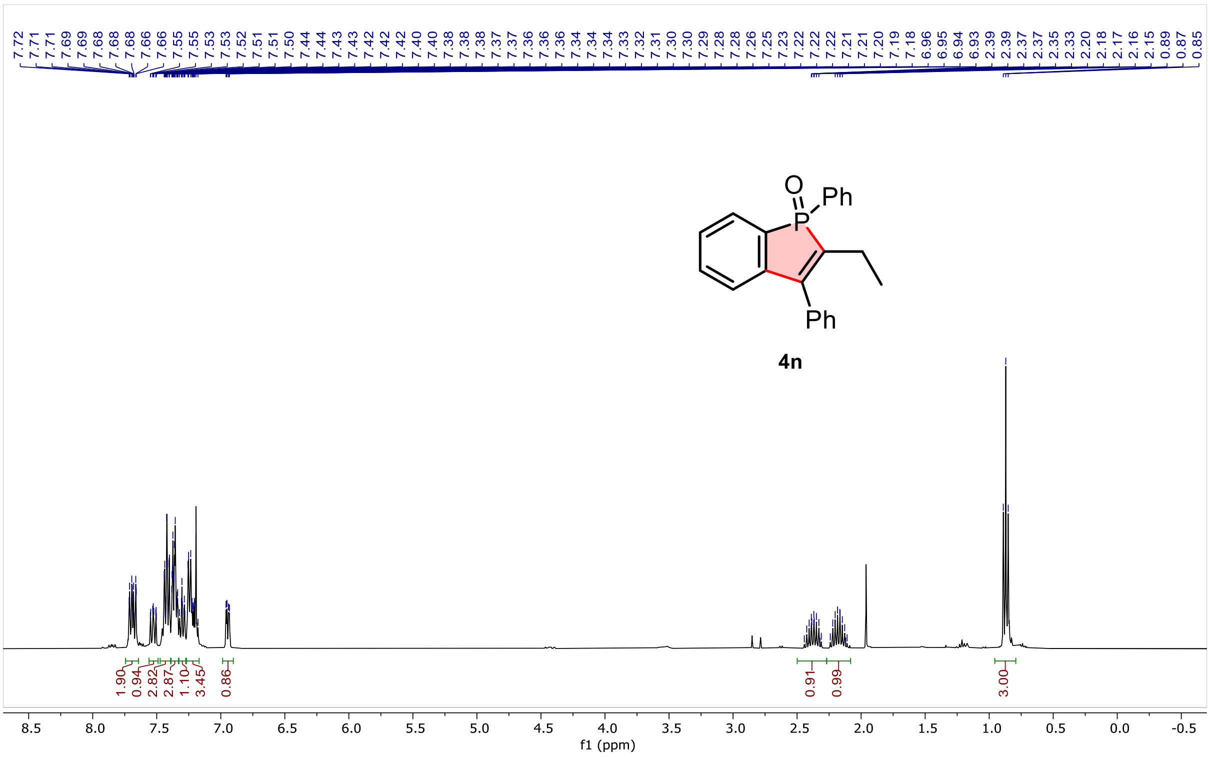

^{13}C NMR (101 MHz, $CDCl_3$) of compound **4n**

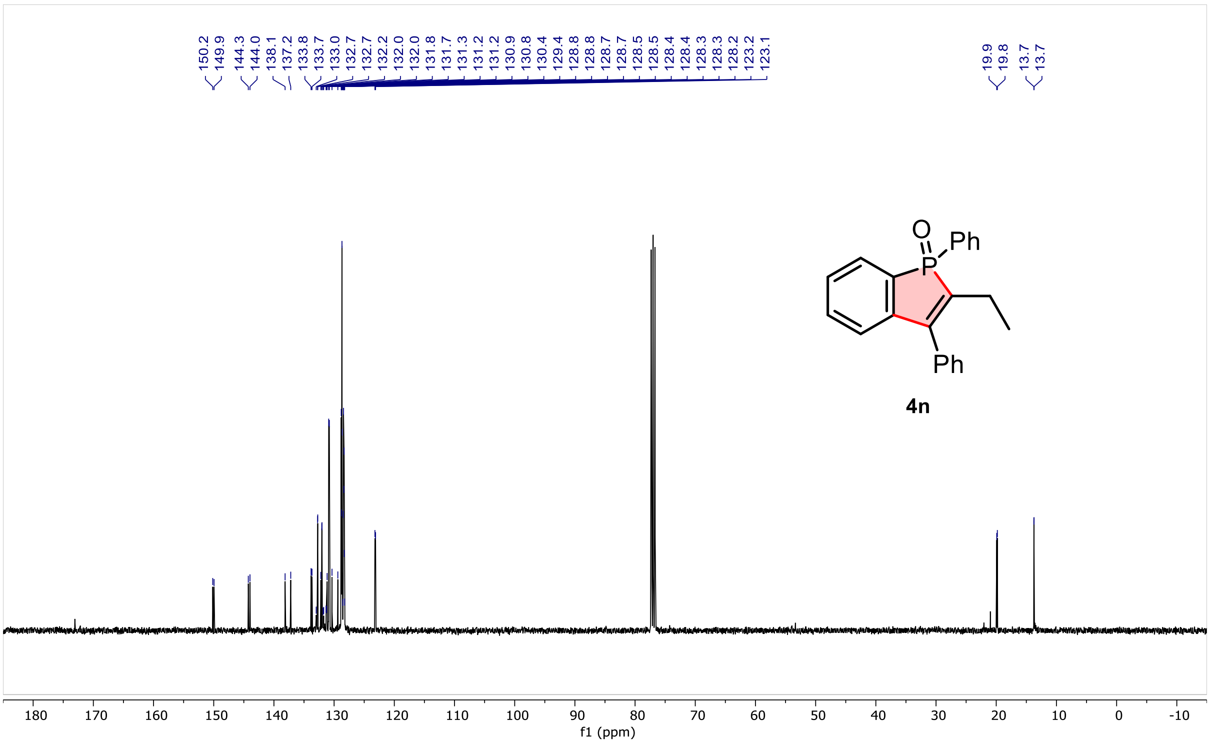

^{31}P NMR (162 MHz, CDCl$_3$) of compound **4n**

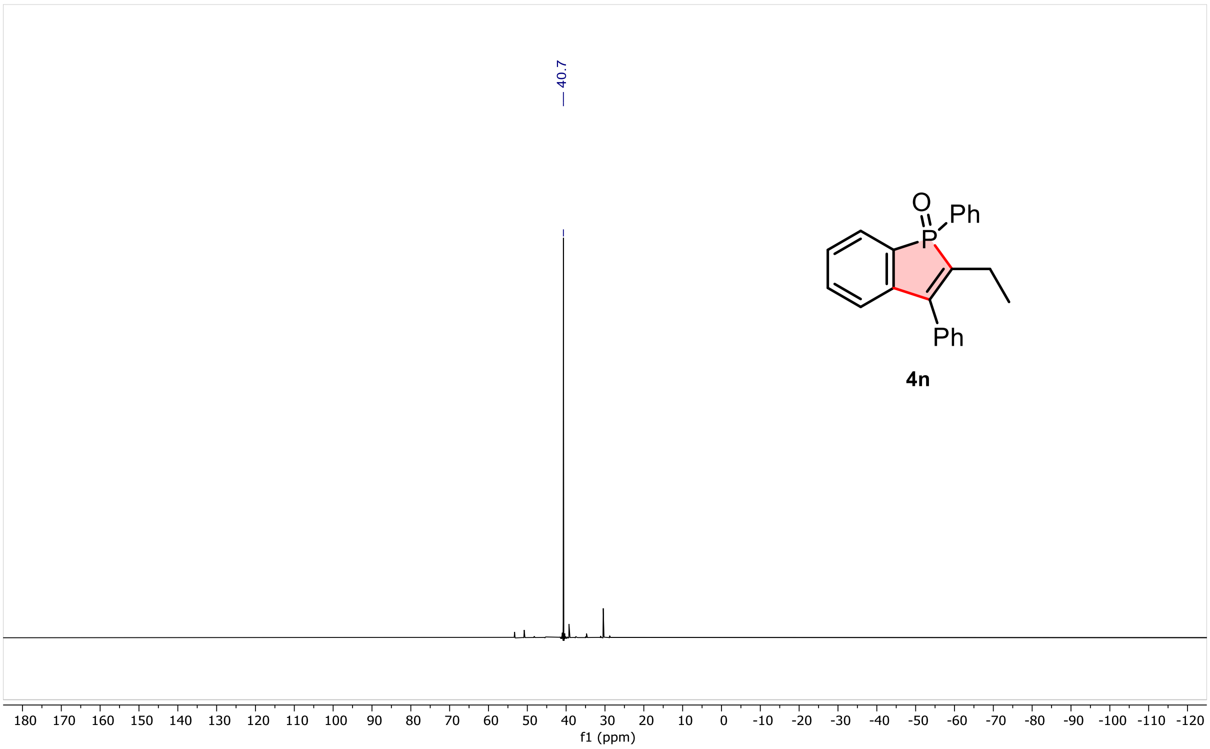

^{1}H NMR (400 MHz, $CDCl_3$) of compound **4o**

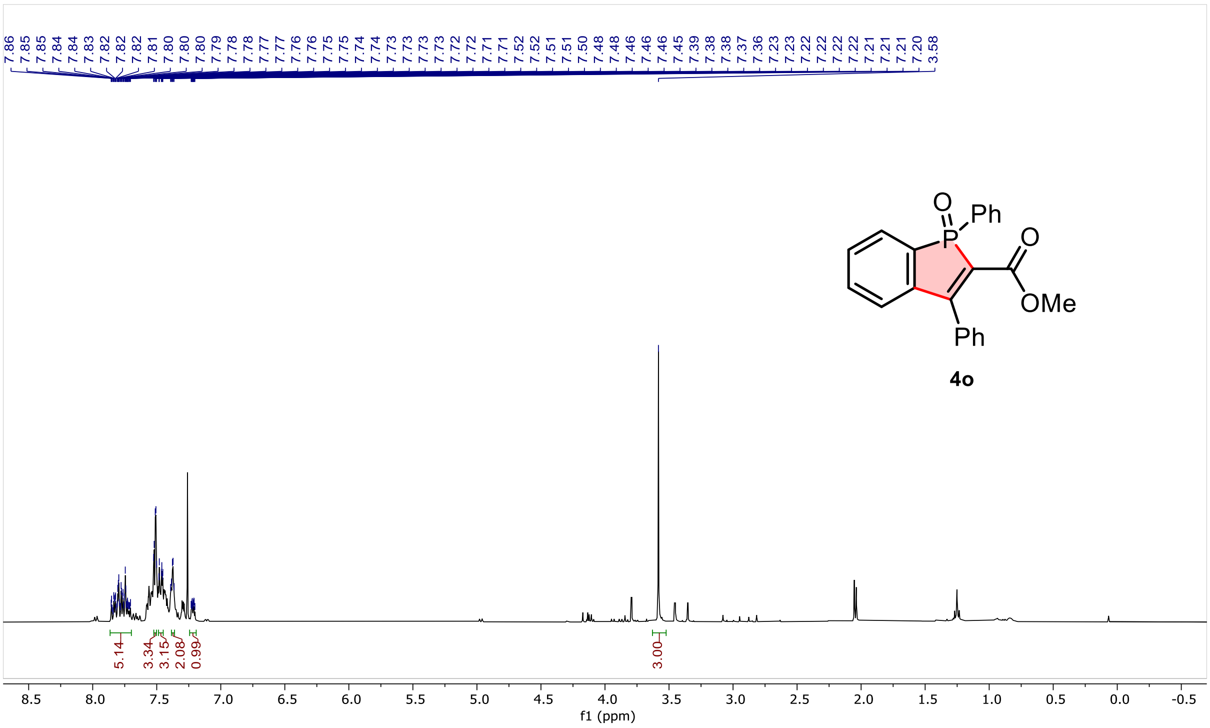

^{13}C NMR (101 MHz, $CDCl_3$) of compound **4o**

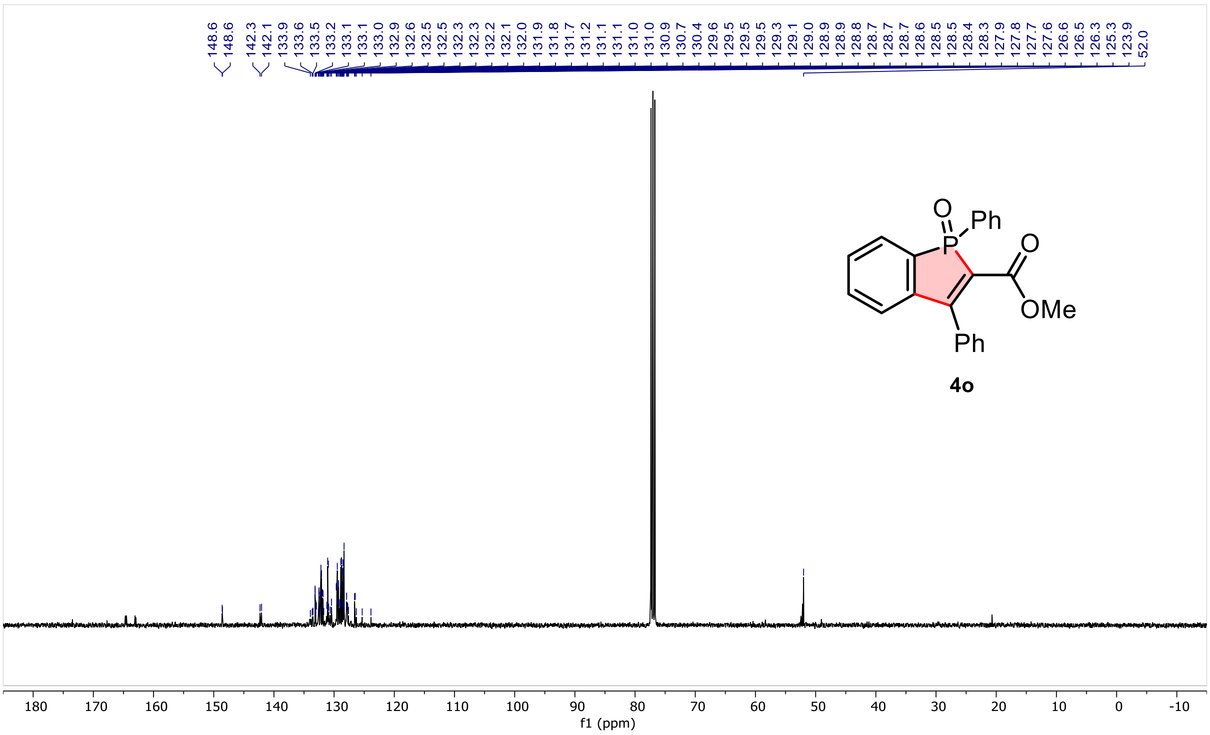

^{31}P NMR (162 MHz, $CDCl_3$) of compound **4o**

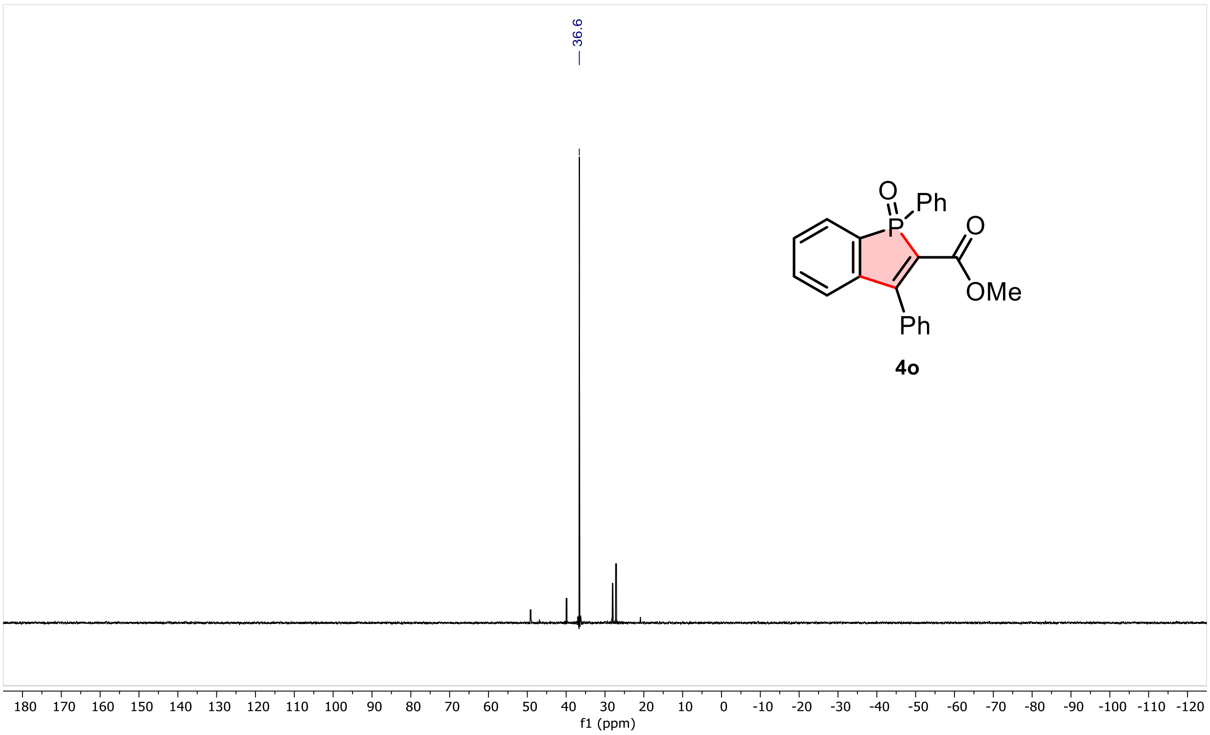

^{1}H NMR (400 MHz, $CDCl_3$) of compound **4p**

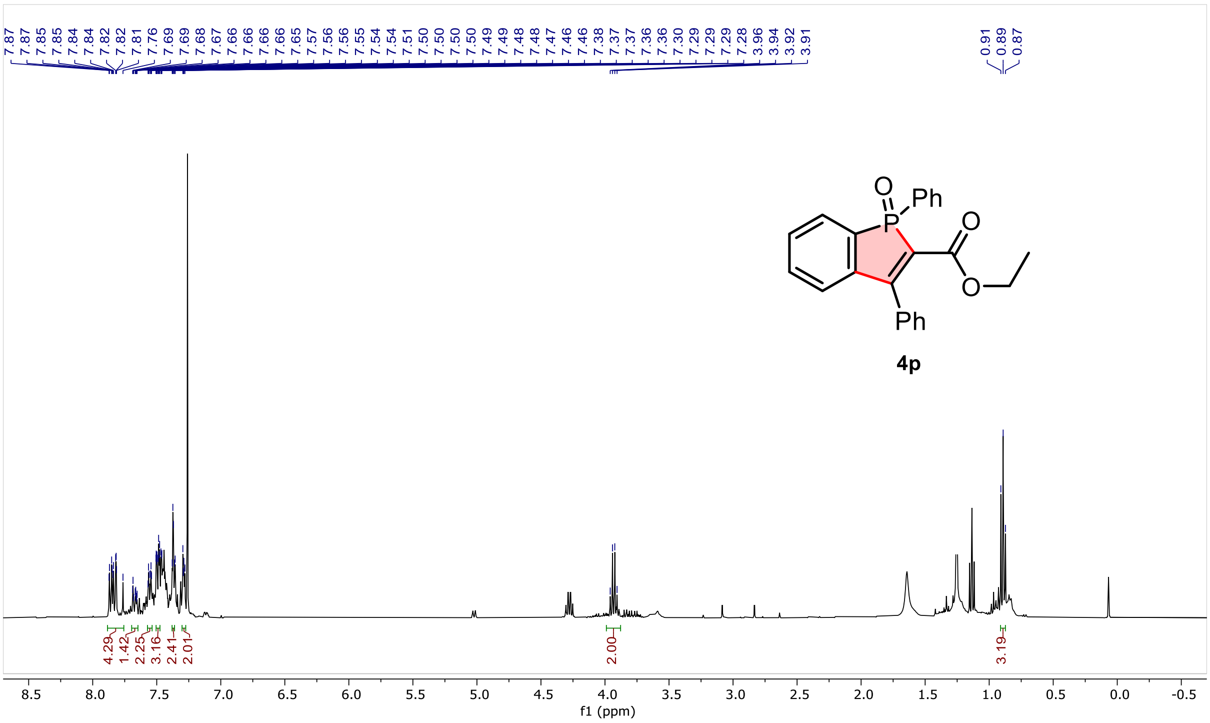

^{13}C NMR (101 MHz, $CDCl_3$) of compound **4p**

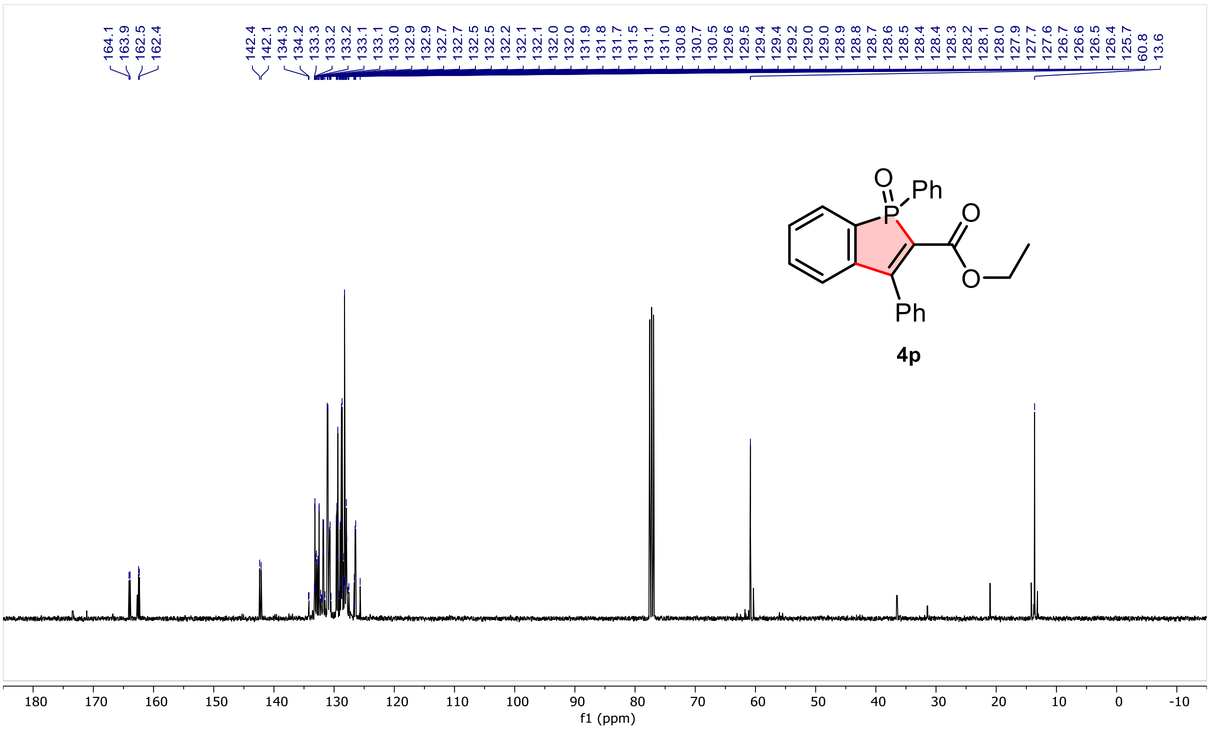

^{31}P NMR (162 MHz, $CDCl_3$) of compound **4p**

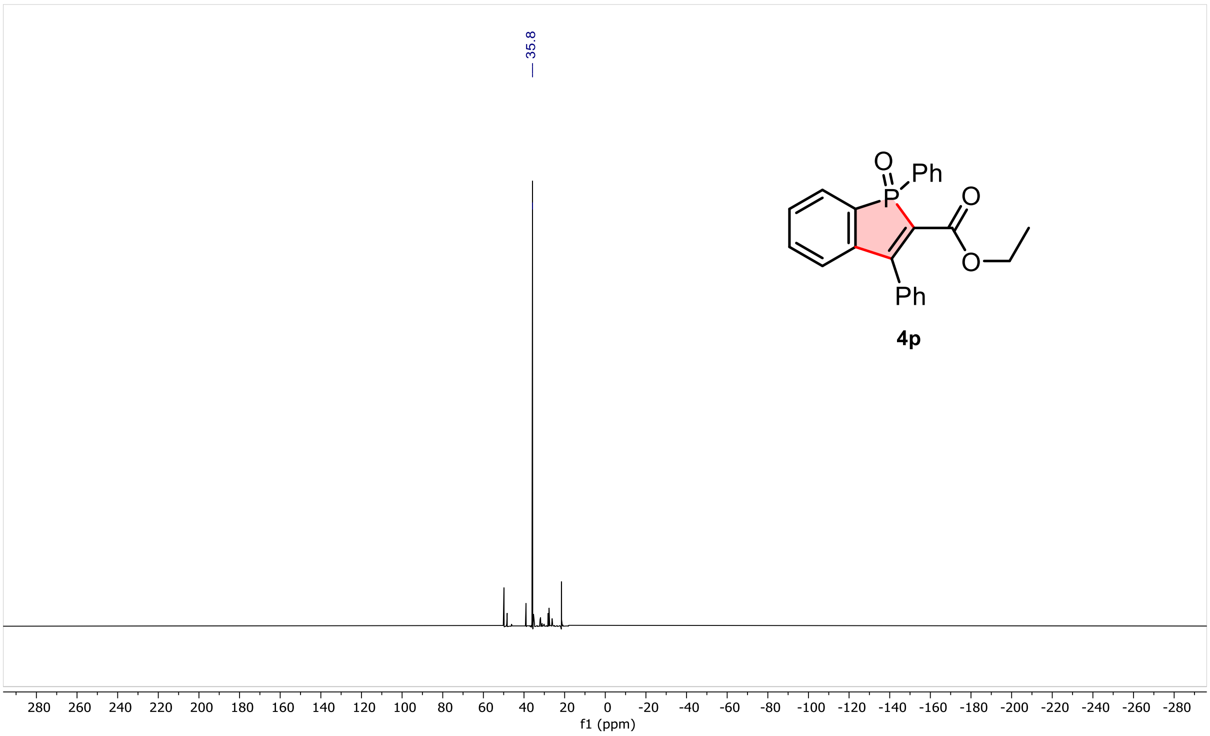

^{1}H NMR (400 MHz, $CDCl_3$) of compound **4q**

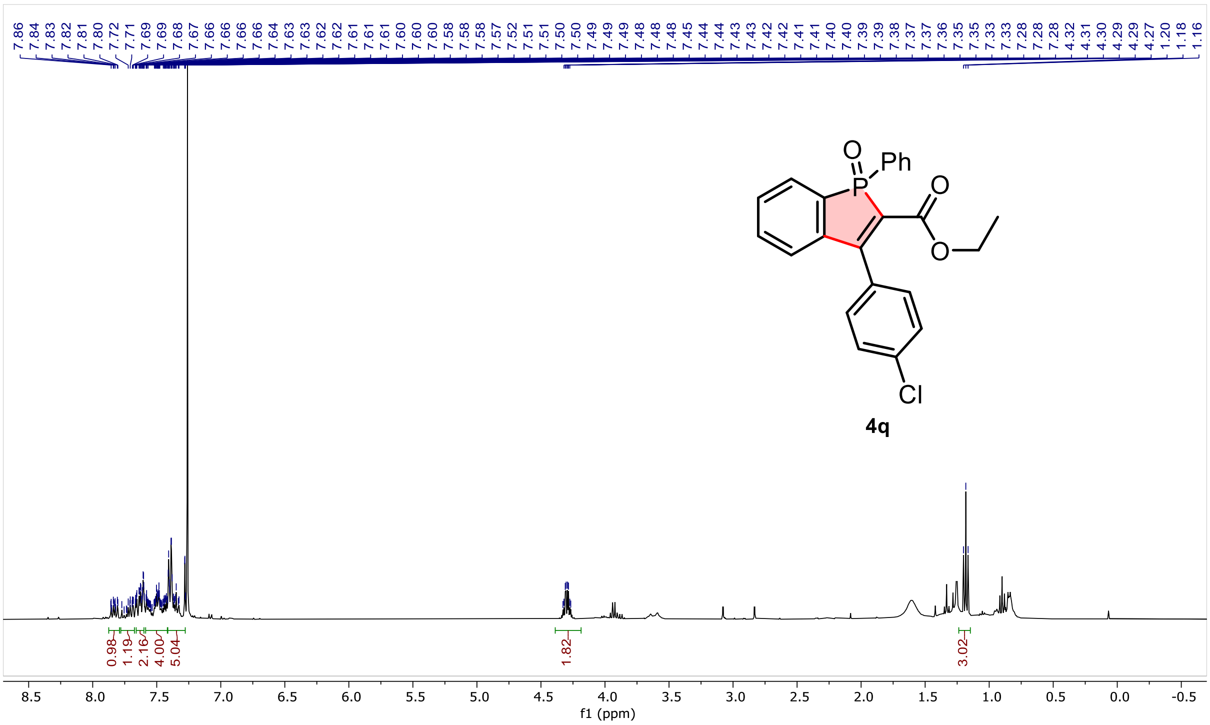

^{13}C NMR (101 MHz, $CDCl_3$) of compound **4q**

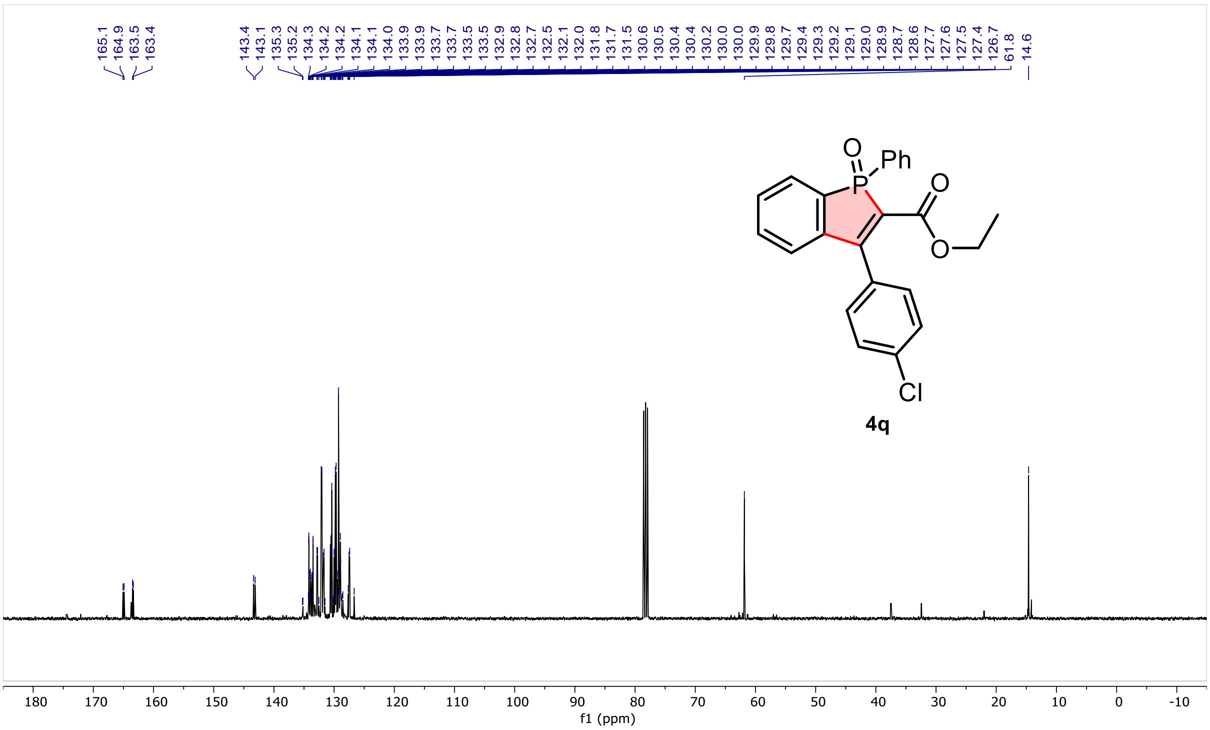

^{31}P NMR (162 MHz, $CDCl_3$) of compound **4q**

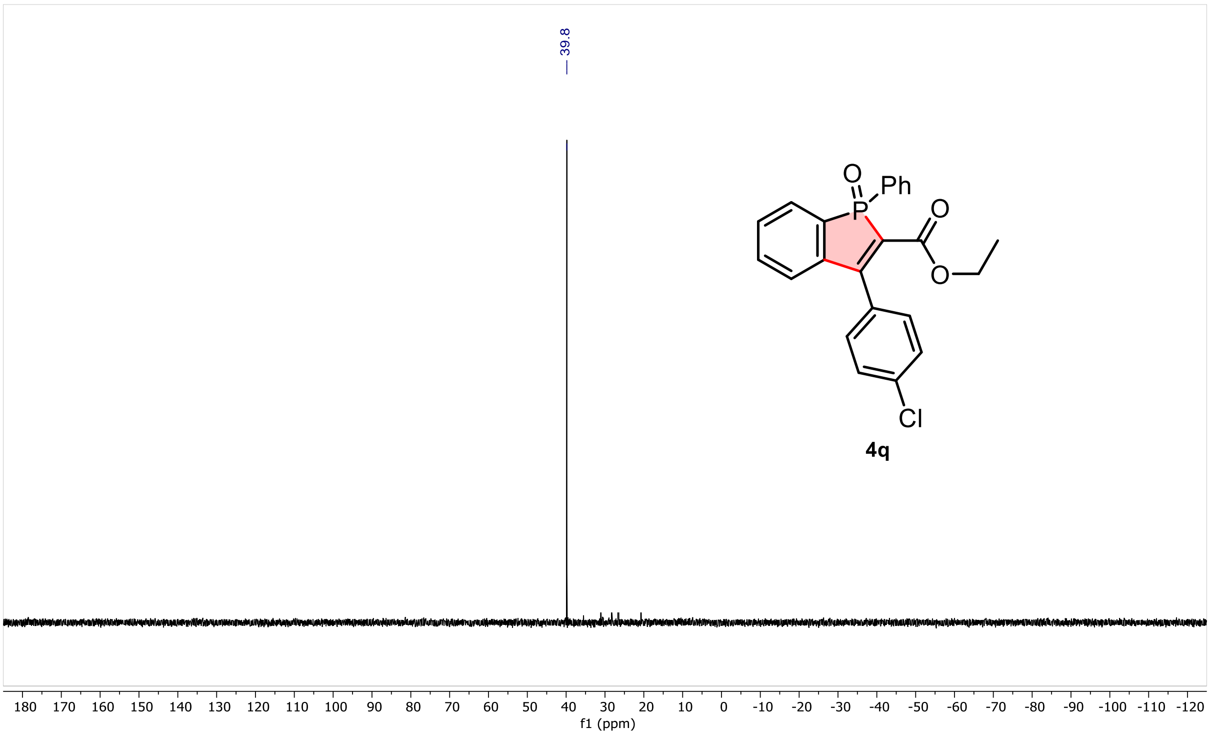

^{1}H NMR (400 MHz, $CDCl_3$) of compound **4r**

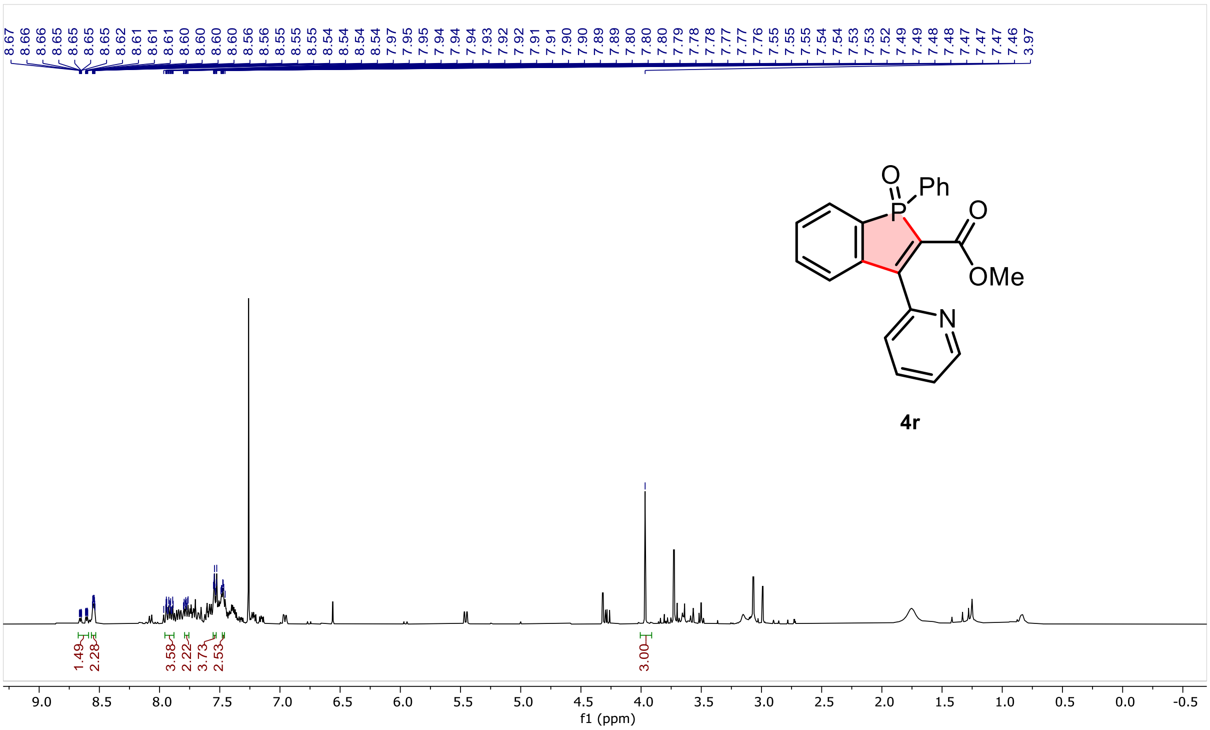

^{13}C NMR (101 MHz, $CDCl_3$) of compound **4r**

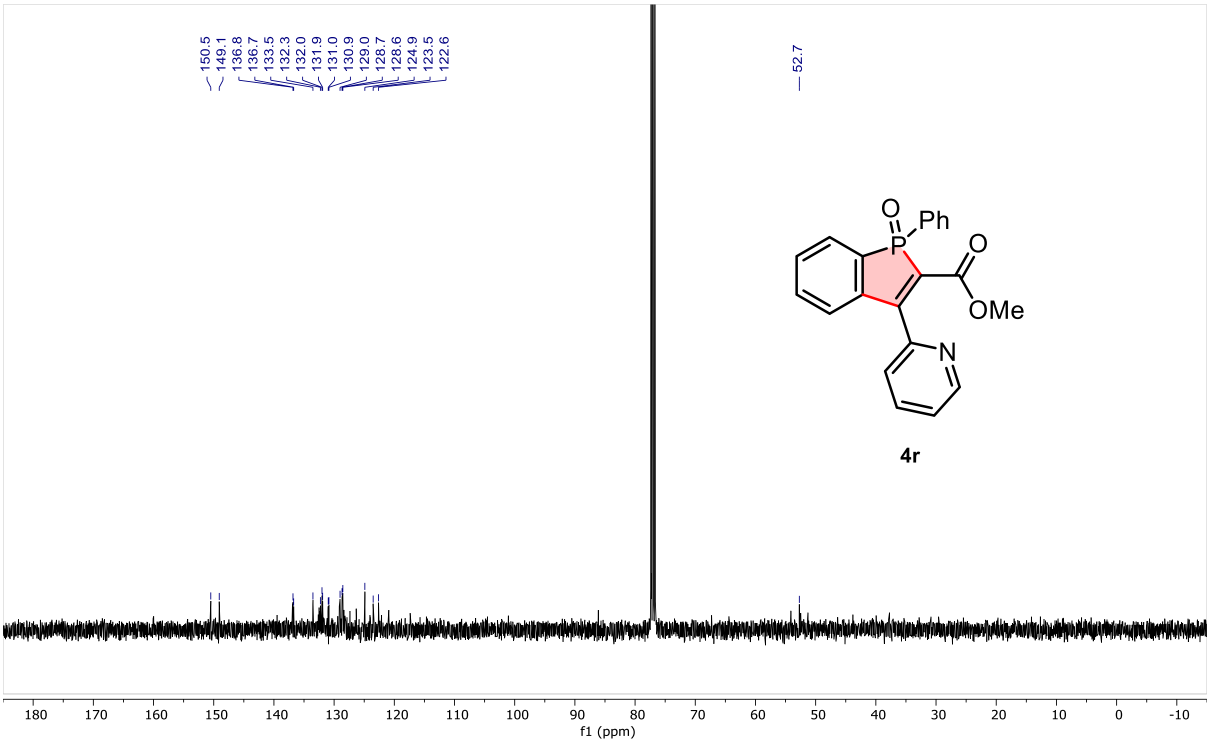

^{31}P NMR (162 MHz, CDCl$_3$) of compound **4r**

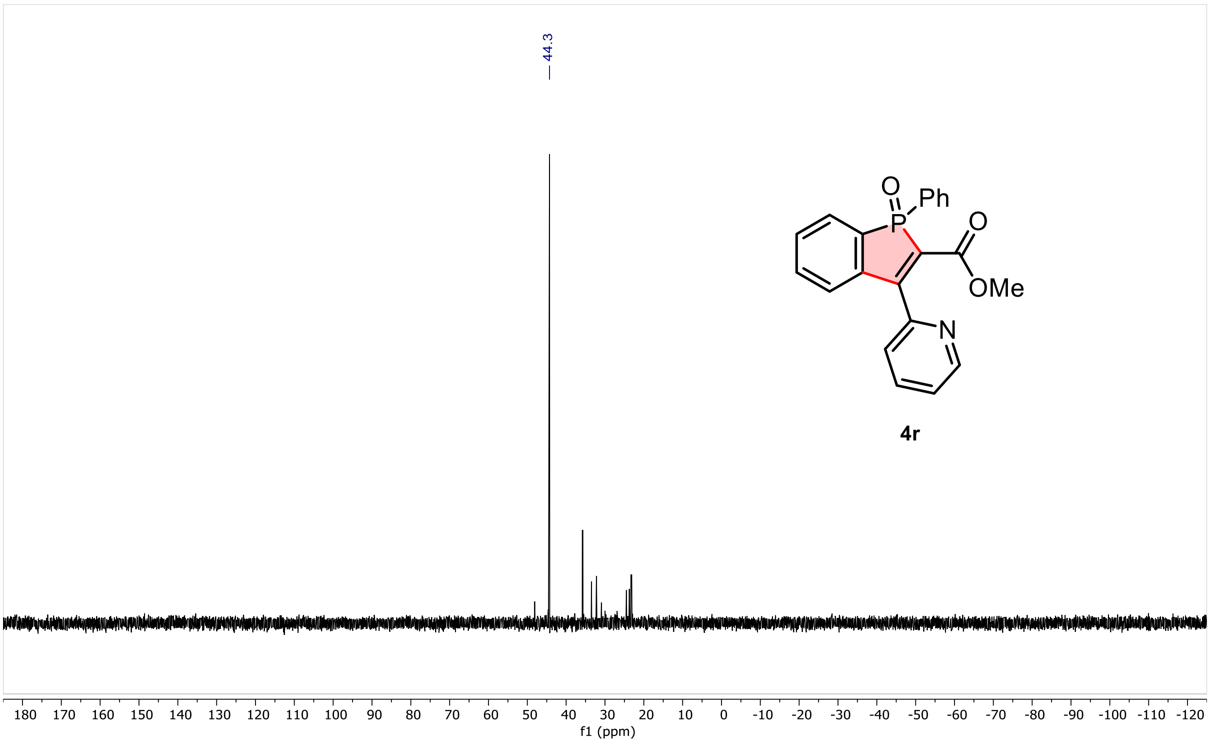

^{1}H NMR (400 MHz, $CDCl_3$) of compound **4s, 4s'**

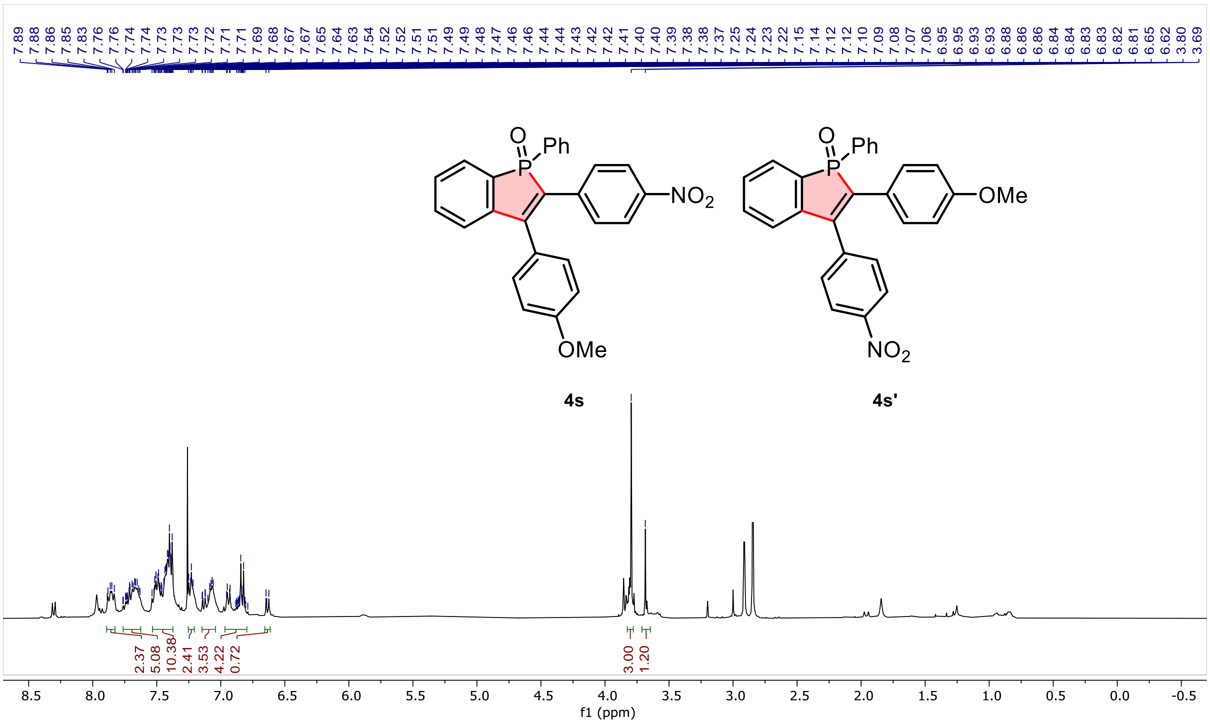

^{13}C NMR (101 MHz, $CDCl_3$) of compound **4s, 4s'**

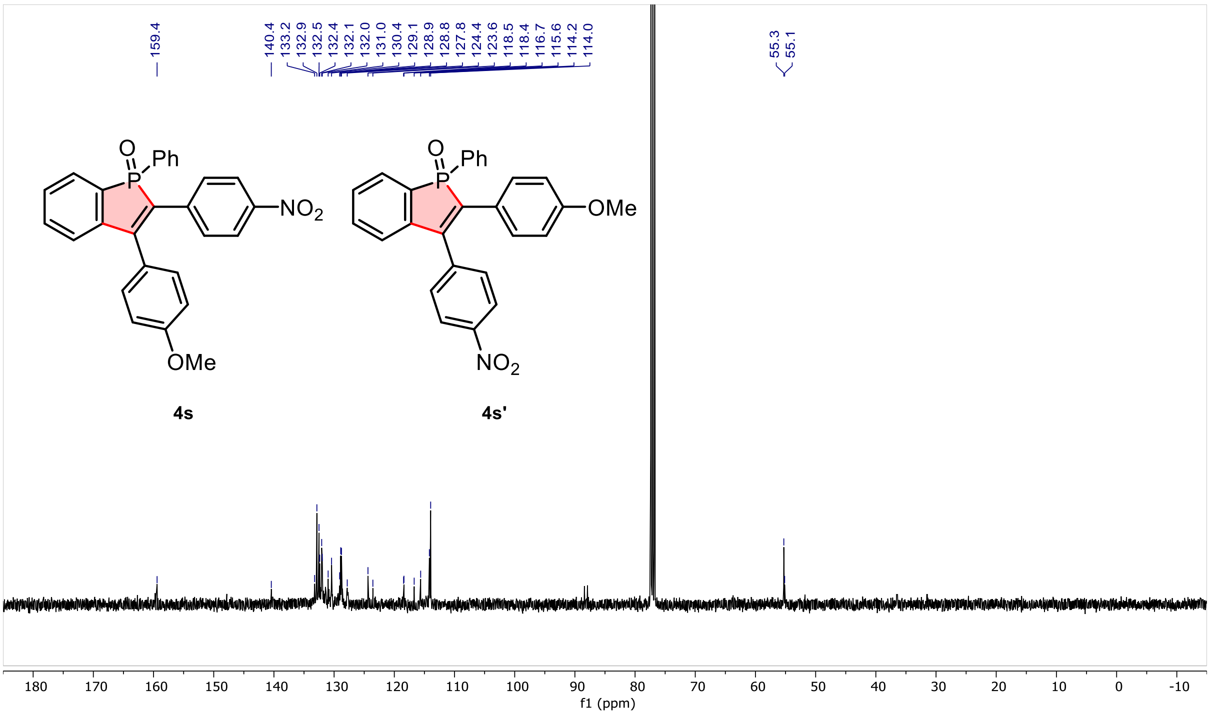

^{31}P NMR (162 MHz, CDCl$_3$) of compound **4s, 4s'**

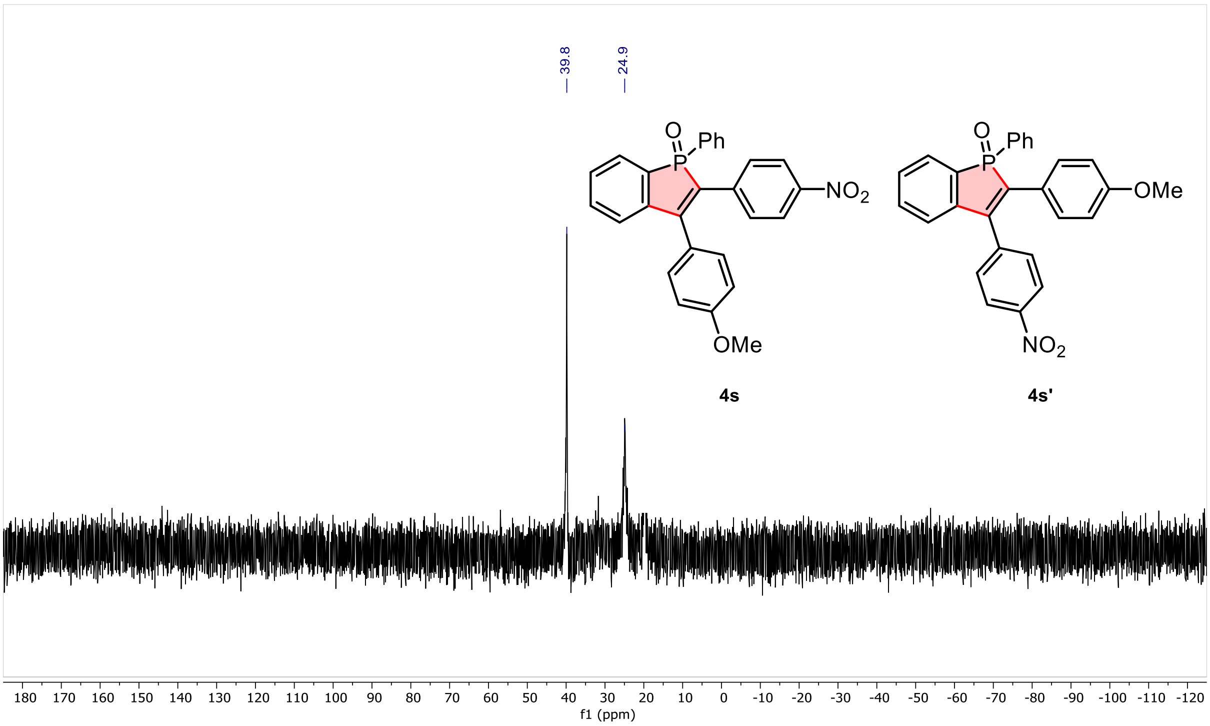

^{1}H NMR (400 MHz, $CDCl_3$) of compound **4t**

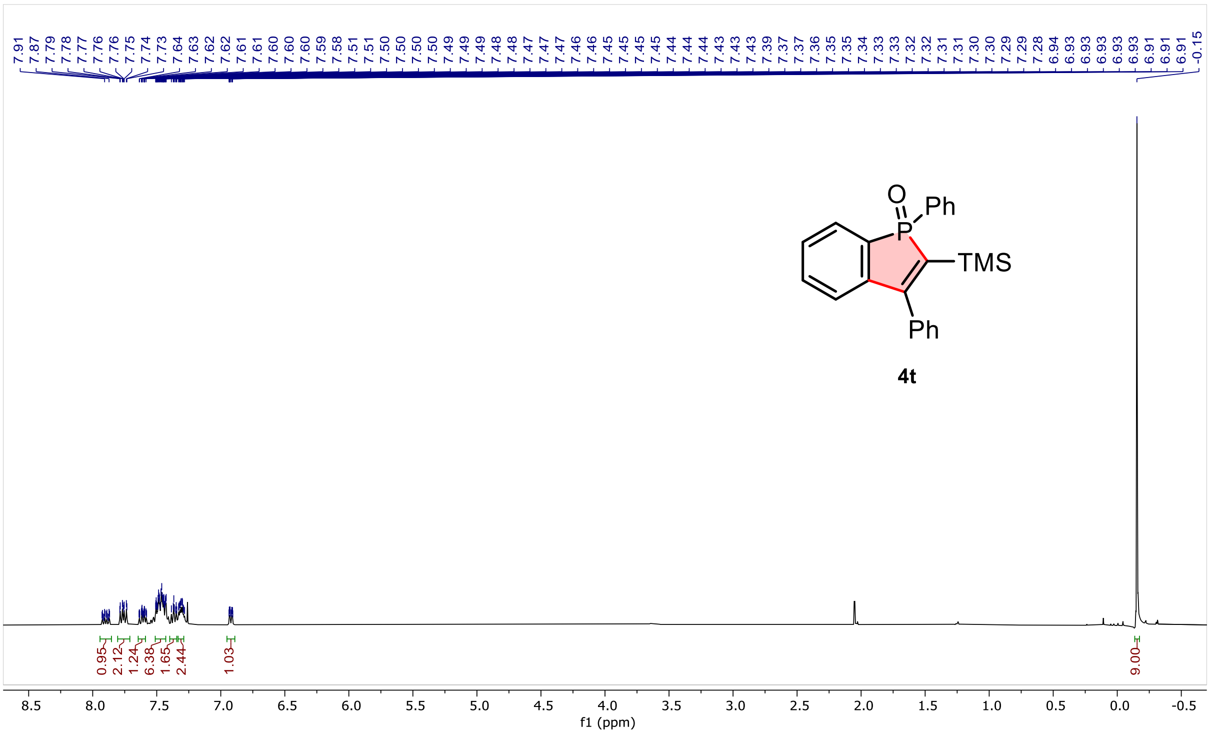

^{13}C NMR (101 MHz, $CDCl_3$) of compound **4t**

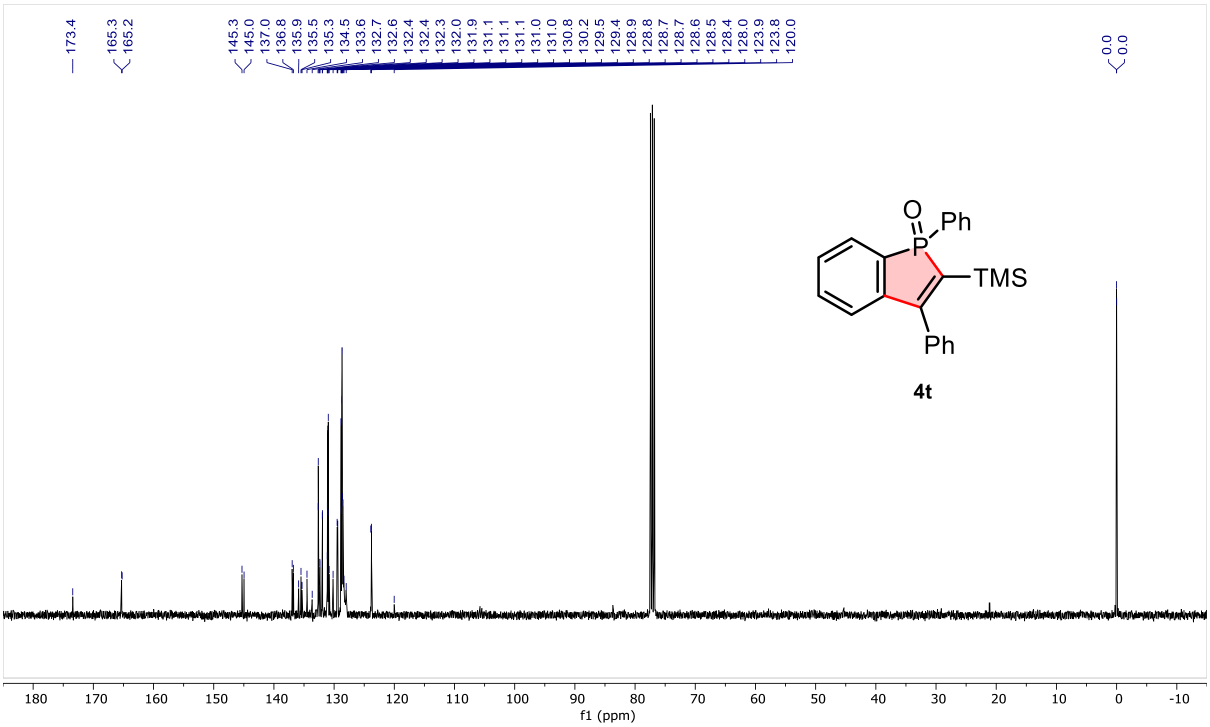

^{31}P NMR (162 MHz, $CDCl_3$) of compound **4t**

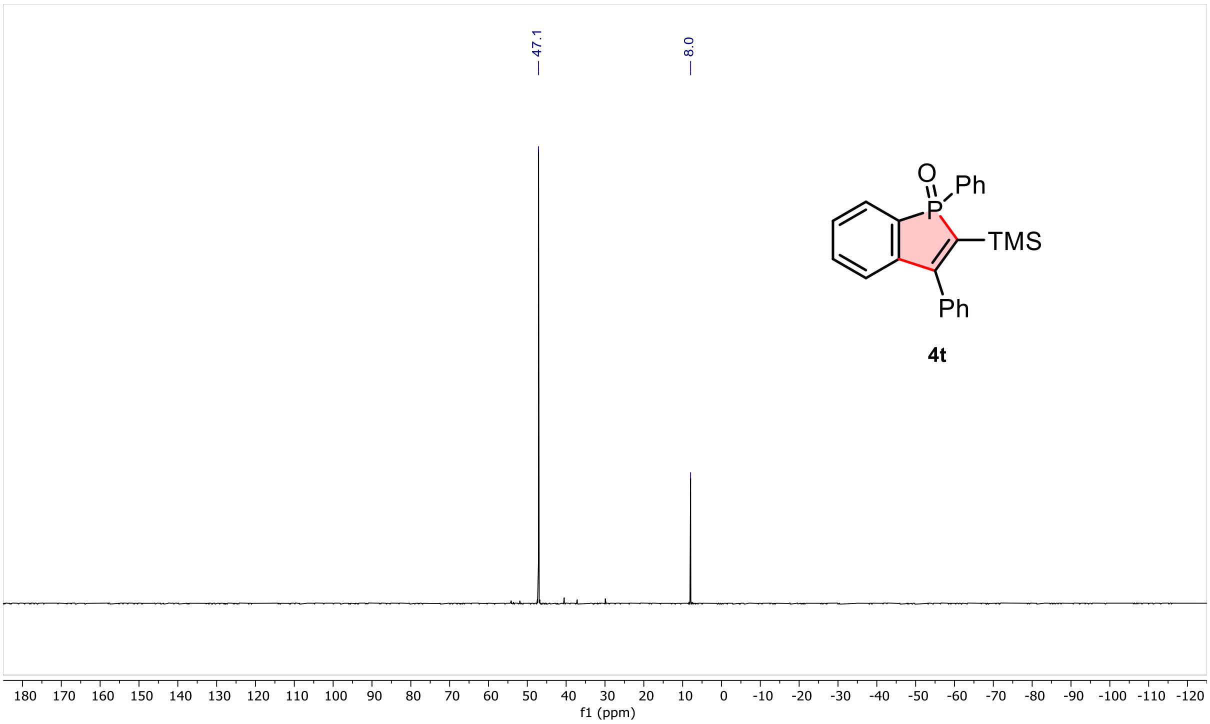

^{1}H NMR (400 MHz, $CDCl_3$) of compound **4u**

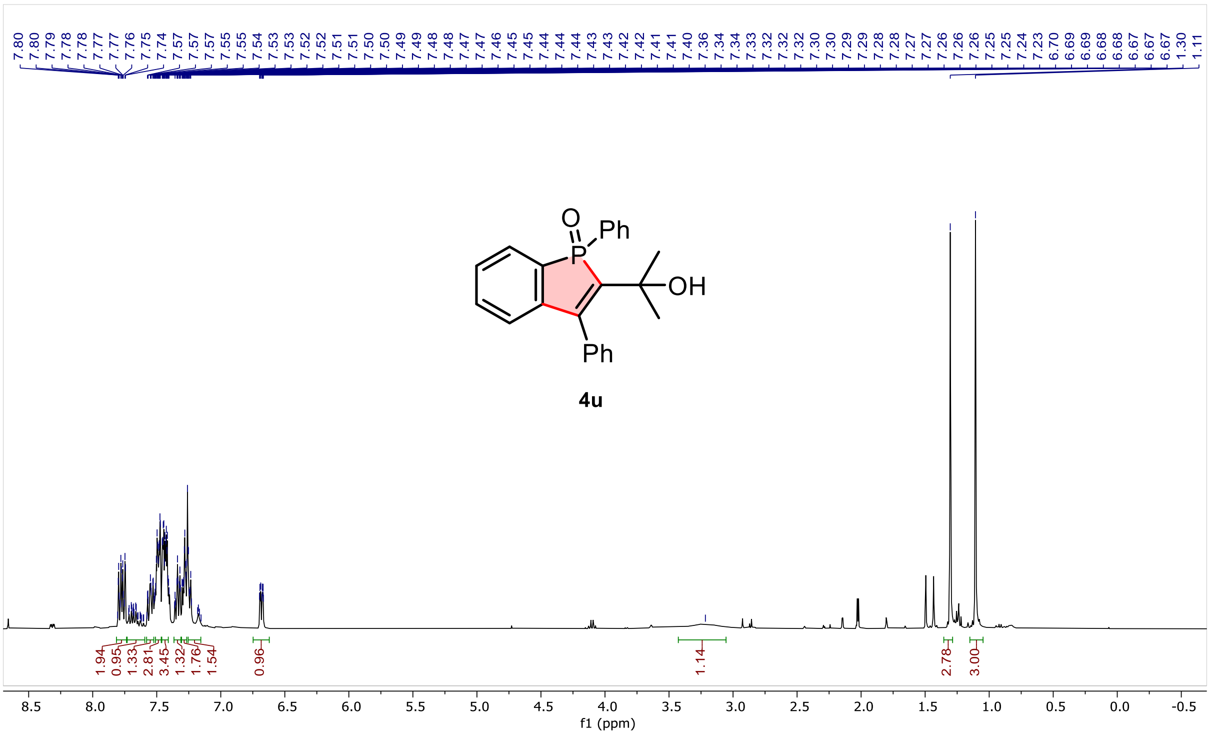

^{13}C NMR (101 MHz, $CDCl_3$) of compound **4u**

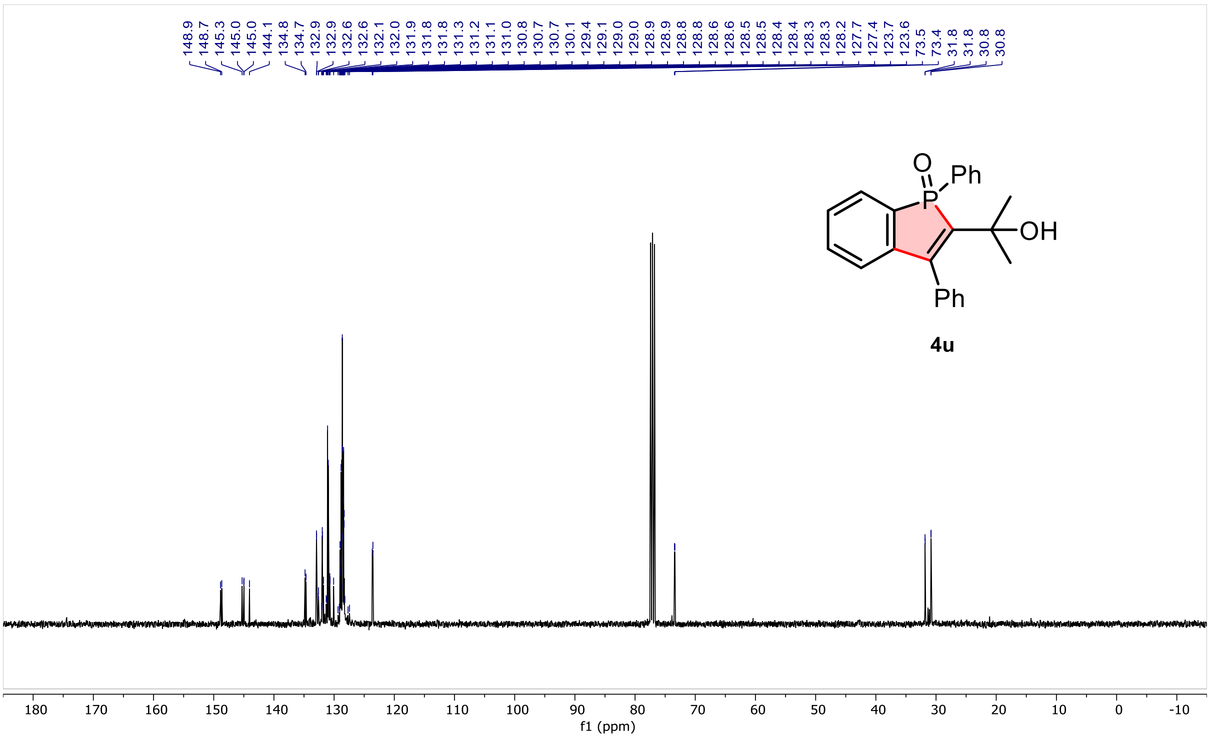

^{31}P NMR (162 MHz, $CDCl_3$) of compound **4u**

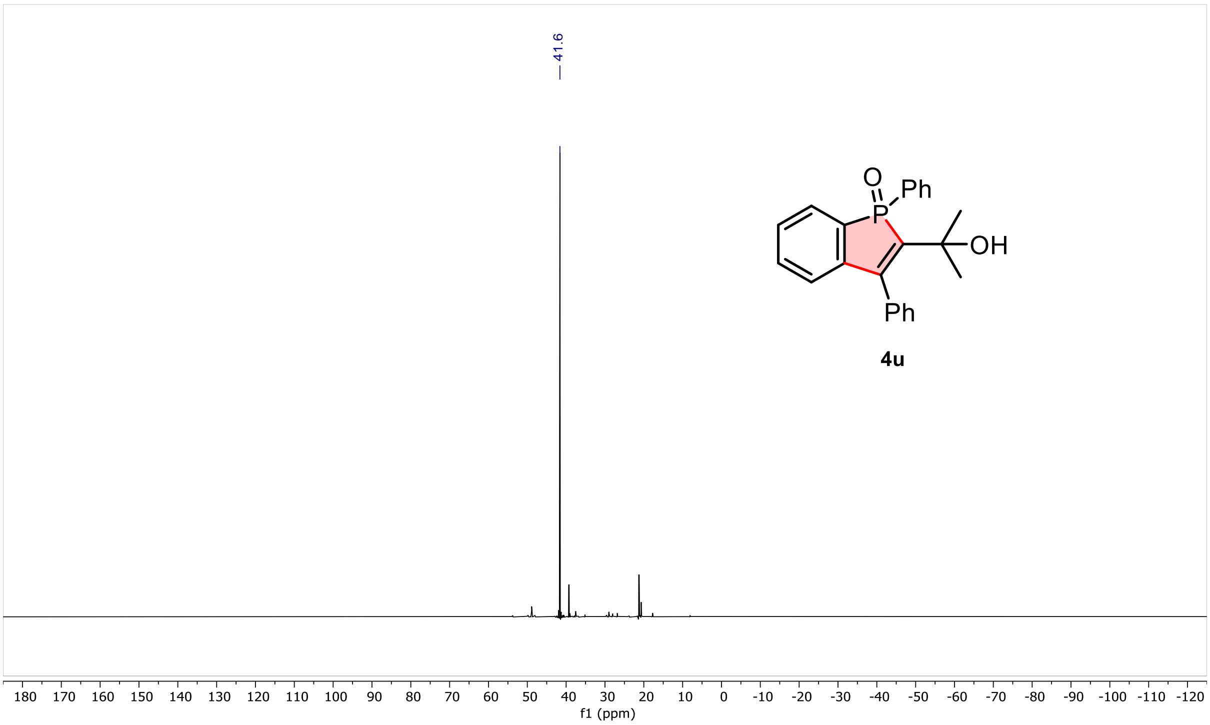

^{1}H NMR (400 MHz, $CDCl_3$) of compound **4v**

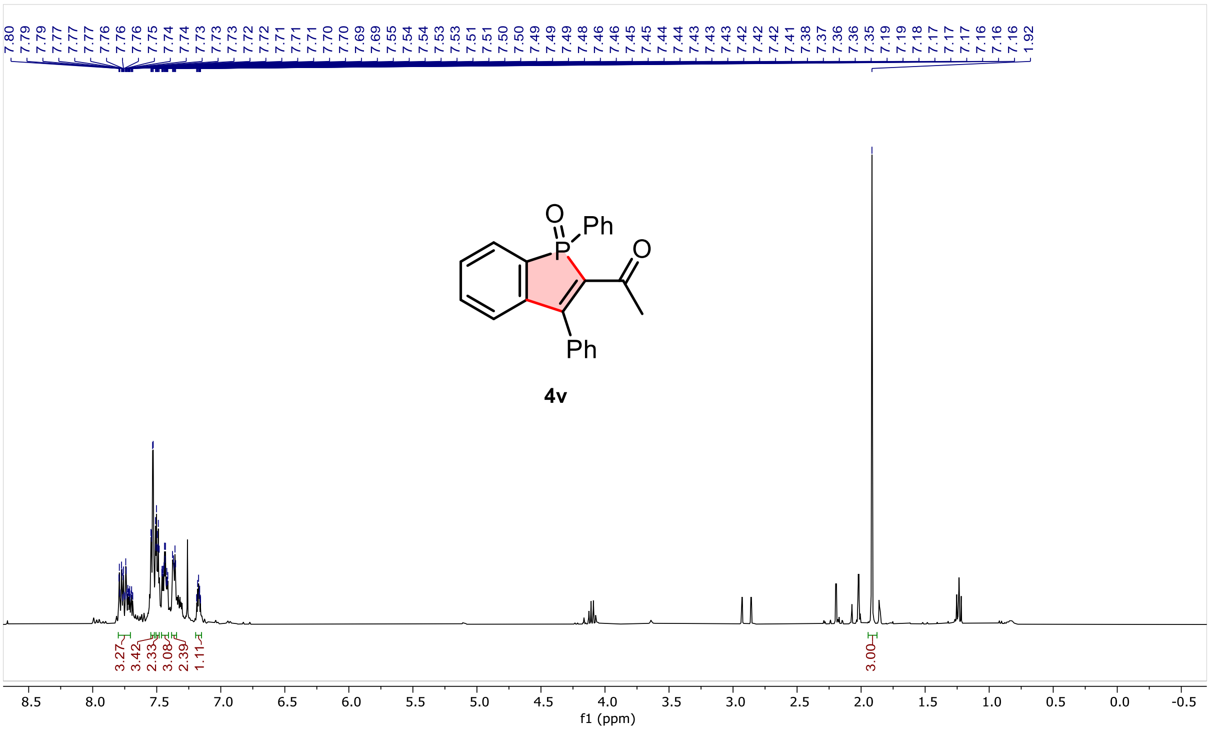

^{13}C NMR (101 MHz, $CDCl_3$) of compound **4v**

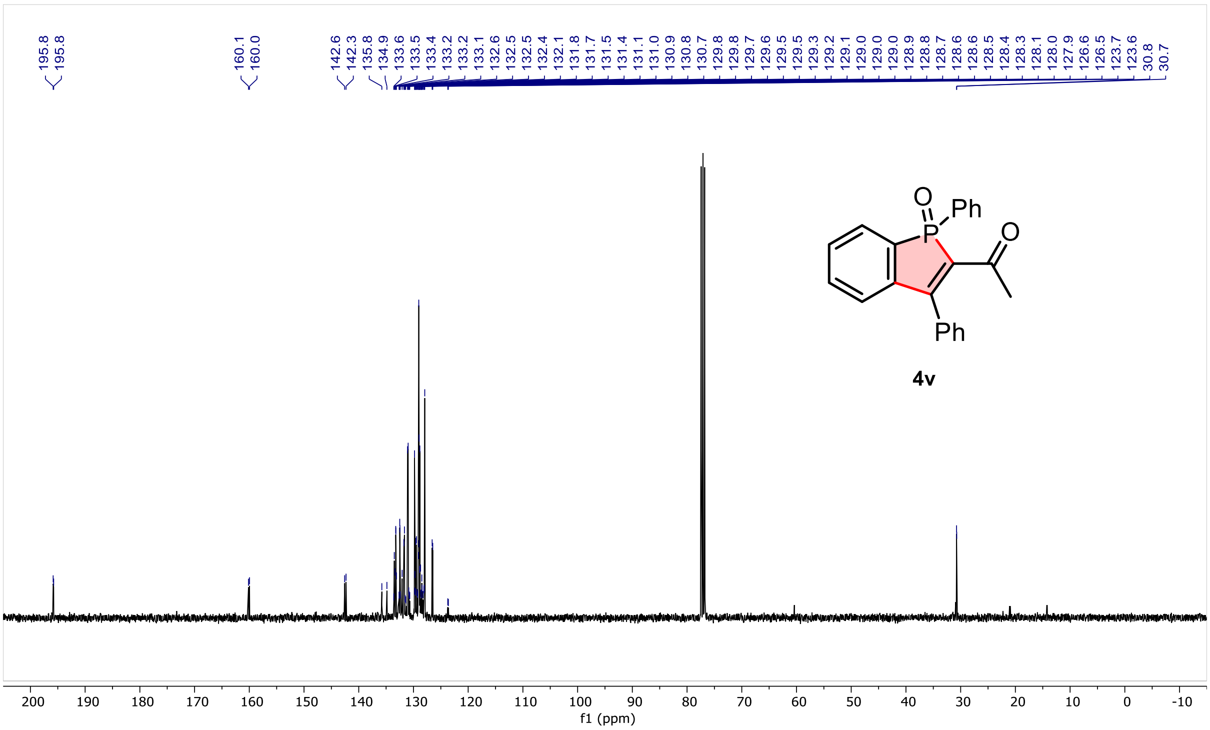

^{31}P NMR (162 MHz, $CDCl_3$) of compound **4v**

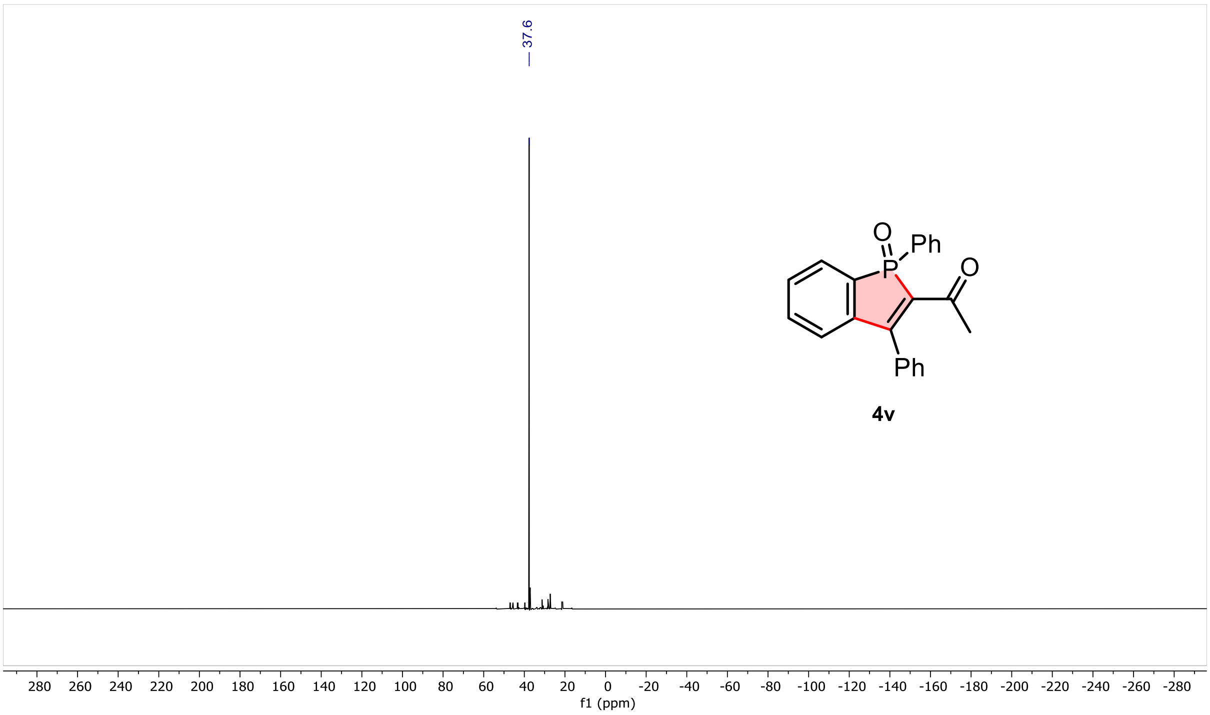

Supplement: Supplementary file 1 — cs5c01983_si_001.pdf [file cs5c01983_si_001.pdf]
